# Functional evolution and rewiring of the UVR8–BES1/BIM1 module underpin the refinement of UV-B responses during plant terrestrialization

**DOI:** 10.1016/j.xplc.2026.101842

**Published:** 2026-04-03

**Authors:** Chengjuan Cao, Runjie Diao, Mengru Zhao, Qiuting Ji, Jingwen Wang, Zilong Xu, Wenhui Xie, Yujun Zhou, Zhenhua Zhang, Bojian Zhong

**Affiliations:** State Key Laboratory of Microbial Technology, College of Life Sciences, Ministry of Education Key Laboratory of NSLSCS, Nanjing Normal University, Nanjing 210023, China

**Keywords:** UVR8–BES1/BIM1 module, UV-B signaling, plant terrestrialization, functional evolution, regulatory networks

## Abstract

The UVR8–BES1/BIM1-mediated crosstalk between UV-B and brassinosteroid (BR) signaling orchestrates transcriptional reprogramming and thereby coordinates BR-mediated growth and UV-B responses in flowering plants. However, when the UVR8–BES1/BIM1 module originated and how this transcriptional regulatory network evolved in plants remain largely unknown. Here, we traced the evolutionary trajectory of the UVR8–BES1/BIM1 module using a structure-guided approach that integrates homology modeling and structural alignment across major plant lineages. By integrating protein interaction modeling, transcriptome profiling, and genome-wide binding analyses, we elucidated the functional evolution of the UVR8–BES1/BIM1 module driven by structural innovations and genetic co-option. Our results reveal that UVR8 and BIM1 orthologs originated in the last common ancestor (LCA) of chlorophytes and maintained a conserved interaction in green plants, whereas BES1 orthologs emerged in the LCA of streptophyte algae and acquired the capacity to interact with UVR8 in vascular plants. BIM1 served as a core UV-B-responsive transcription factor in the LCA of green plants. By contrast, BES1 initially participated in UV-B signaling through a BIM1-dependent mechanism in the LCA of land plants and later evolved to function as a dominant integrator within UV-B–BR crosstalk in angiosperms. The expansion of the BES1 regulatory network and its binding specificity largely parallels the elaboration of UV-B transcriptional programs during land plant evolution. Our study thus demonstrates that the functional evolution of the UVR8–BES1/BIM1 module enables the stepwise integration of UV-B and BR signaling in green plants, advancing our understanding of how plants have wired hormonal and environmental signals to adapt to terrestrial habitats.

## Teaser

The UVR8–BES1/BIM1 module plays a critical role in coordinating UV-B and brassinosteroid signaling to regulate growth in flowering plants. Using a structure-guided phylogenetic approach, this study traces of the evolutionary trajectory of this module and characterizes of its stepwise assembly and the progressive refinement of downstream networks during terrestrialization. This evolutionary process enables plants to balance growth and UV-B stress responses, facilitating their successful establishment in terrestrial habitats.

## Introduction

The colonization of land by plants, which occurred approximately 480 million years ago, fundamentally altered the light environments they inhabit (Maberly, 2014; Morris et al., 2018; Furst-Jansen et al., 2020). UV-B wavelengths (280–315 nm) can damage DNA molecules and also induce plant photomorphogenesis and UV-B acclimation responses (Reyero-Saavedra et al., 2023). Although algae typically experience low doses of UV-B, which are attenuated by water, they have developed a UV-B signaling pathway that is mediated by the photoreceptor UV resistance locus 8 (UVR8) and induces UV-B acclimation (Rozema et al., 2002; Fernandez et al., 2016; Tilbrook et al., 2016). By contrast, land plants must confront highly dynamic UV-B radiation and have evolved sophisticated mechanisms to integrate UV-B signaling with other signaling pathways and achieve UV-B acclimation (Zhang et al., 2022a). Given the broad effects of UV-B on plants, it is crucial to understand the evolution of UV-B signaling and its integration with plant development and adaptation through the differential expression of numerous genes.

UVR8 serves as the UV-B photoreceptor in green plants and is functionally conserved from green algae to flowering plants (Podolec et al., 2021). UV-B-activated UVR8 signaling occurs mainly in the nucleus: (1) in the absence of UV-B, UVR8 forms an inactive homodimer; and (2) upon UV-B exposure, UVR8 uses tryptophan residues as chromophores to sense UV-B radiation, triggering a conformational change from an inactive homodimer to an active monomer. The active monomers interact with key downstream regulators to transmit UV-B signals, thereby modulating gene expression, evoking different physiological responses, and enhancing UV-B tolerance (Liang et al., 2019; O'Hara and Jenkins, 2012; Rizzini et al., 2011). Two different mechanisms of UVR8 signaling have been reported: (1) UVR8 interacts with the E3 ubiquitin ligase COP1 (constitutively photomorphogenic 1), thereby regulating COP1-targeted transcription factors (TFs) that in turn alter gene expression; and (2) UVR8 directly interacts with specific TFs, altering their binding to DNA and thus the transcription of their target genes (Oravecz et al., 2006; Hayes et al., 2014; Liang et al., 2018, 2019; Yang et al., 2018). The UVR8–COP1 pathway is evolutionarily conserved from green algae to flowering plants; however, it is unclear whether the direct action of UVR8 on specific TFs is functionally conserved (Han et al., 2019). BES1 (BRI1–EMS-suppressor 1) and BIM1 (BES1-interacting Myc-like 1) are representative TFs that directly interact with UVR8. In *Arabidopsis thaliana*, the UVR8–BES1/BIM1 module forms a key regulatory nexus linking exogenous UV-B signaling with endogenous brassinosteroid (BR) responses to balance growth–stress trade-offs (Liang et al., 2018, 2020). UV-B-activated UVR8 monomers directly interact with the non-phosphorylated forms of BES1 and BIM1, inhibiting their DNA-binding activity. This downregulates the expression of growth-promoting genes, suppressing cell elongation and promoting photomorphogenesis (Liang et al., 2018). Notably, BES1 also contributes to UV-B acclimation by promoting flavonoid accumulation in a UVR8-independent manner (Liang et al., 2020; Shi and Liu, 2021). Sequence-based homology searches indicated that *UVR8* and *BIM1* orthologs originated in the last common ancestor (LCA) of chlorophytes, whereas *BES1* orthologs emerged in the LCA of streptophyte algae (Delaux et al., 2019; Zhang et al., 2022a). However, the origin and functional evolution of the UVR8–BES1/BIM1 module in mediating UV-B signaling across green plant lineages is poorly understood.

The regulatory capacity of the UVR8–BES1/BIM1 module depends on the structural organization and intrinsic DNA-binding properties of BES1 and BIM1. AtBIM1 (AT5G08130) belongs to the typical basic helix–loop–helix (bHLH) TF family, and the His/Lys/Arg(i−4), Glu(i), Arg(i+3), and Arg(i+4) residues (H/K/RxxxExxRR motif) in the bHLH domain collectively determine its binding specificity toward G-box elements (CACGTG). AtBES1 (AT1G19350) exhibits a bHLH-like architecture characterized by a β-hairpin immediately following the first α-helix, an intervening loop, and a truncated second α-helix (Yin et al., 2005; Ezer et al., 2017; Nosaki et al., 2018). This distinctive structural configuration induces more pronounced conformational changes in both the major and minor grooves of bound DNA, thereby relaxing sequence constraints and conferring on AtBES1 a broader capacity for *cis*-element recognition (Nosaki et al., 2022). AtBES1 can bind not only G-box but also E-box motifs (CANNTG) and the BR-response element (BRRE, CGTG^C^/_T_G) (Yin et al., 2005; Yu et al., 2011). Moreover, heterodimer assembly between BES1 and typical bHLH TFs may enable a key Glu (E) residue to bind into the major groove of the E-box motif by making use of the tilt-angle variation of the DNA recognition helix with a conformational change in dimerization regions, coordinating the crosstalk between phytohormone and environmental signaling (Oh et al., 2012; Nosaki et al., 2018). Thus, exploring the evolutionary trajectories of BES1 and BIM1 across major green plant lineages is essential for understanding the evolutionary plasticity of UV-B signaling.

In this study, we used AlphaFold2-based structural modeling to systematically investigate the origin and functional evolution of the UVR8–BES1/BIM1 module in green plants. By integrating structure-guided phylogenies, protein–protein interaction (PPI) assays, and comparative omics analyses, we revealed how UV-B and BR signaling pathways evolved to interconnect during land plant evolution. Our findings provide new insights into the multi-signal integration of light and hormone signaling networks and highlight the emergence of the UVR8–BES1/BIM1 module as a key innovation that enabled plants to adapt to changing light environments on land.

## Results

### Evolution of UVR8, BES1, and BIM1 revealed by structure-guided phylogenies

**Figure 1:**
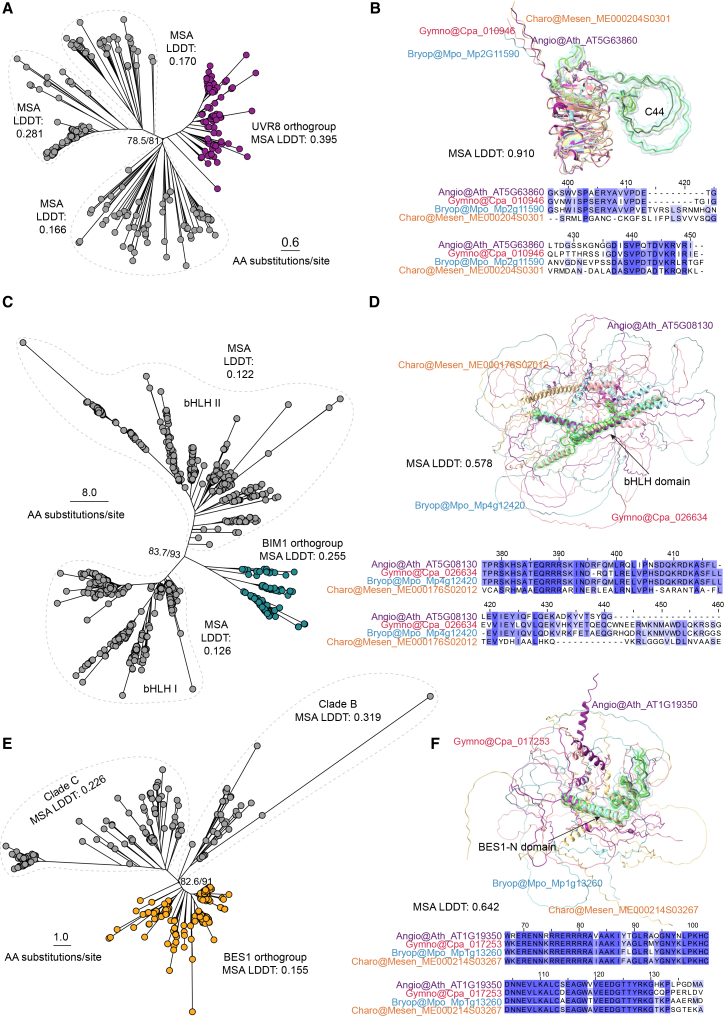
Evolutionary history of UVR8, BES1, and BIM1 revealed by structure-guided phylogeny. **(A)** Phylogenetic tree of UVR8 orthologs. The tree was generated by IQ-TREE 2 using the WAG+F+R8 model with 1000 ultrafast bootstraps (UFBS). The UVR8 orthogroup is indicated with purple circles. **(B)** Structural alignment of representative UVR8 orthologs. Sequence alignment of the C44 region of UVR8 is shown. **(C)** Phylogenetic tree of BIM1 orthologs. The tree was generated by IQ-TREE 2 using the JTT+F+R10 model with 1000 UFBS. The BIM1 orthogroup is indicated with cyan circles. **(D)** Structural alignment of representative BIM1 orthologs. Sequence alignment of the bHLH domain is shown. **(E)** Phylogenetic tree of BES1 orthologs. The tree was generated by IQ-TREE 2 using the JTT+F+R7 model with 1000 UFBS. The BES1 orthogroup is indicated with yellow circles. **(F)** Structural alignment of representative BES1 orthologs. Sequence alignment of the N-terminal region is shown. Multiple sequence alignment LDDT scores quantify local atomic distance deviations across multiple sequence alignments and are used to assess structural alignment quality.

We used a structure-guided phylogenetic framework to characterize the evolutionary trajectories of the UVR8–BES1/BIM1 module in green plants, integrating deep learning-based protein structure prediction with structural alignment (Kim et al., 2025). The structure-guided phylogenies identified UVR8, BIM1, and BES1 orthologs, with orthologous groups exhibiting higher pairwise structural similarity than paralogous groups (Figure 1 and Supplemental Figures 1–3 and 7). The results provided further support for the notion that *UVR8* and *BIM1* orthologs originated in the LCA of chlorophytes and that the *BES1* ortholog emerged in the LCA of streptophyte algae, a scenario largely congruent with tree topologies produced by the sequence-based approach (Zhang et al., 2022a; Figure 1 and Supplemental Figures 4–6). Structure-guided analyses markedly improved the resolution of ortholog/paralog discrimination, particularly in ancient and highly divergent clades (Figure 1). For example, two UVR8-like sequences from the green algae *Chloroidium sp*. (Chsp_0000611-CF150.5) and *Ulva mutabilis* (Ulvmu_UM015_0123.1) were misclassified as UVR8 orthologs on the basis of sequence similarity but were excluded by structure-guided analyses. Inspection of their structures revealed that Chsp_0000611-CF150.5 exhibits structural features corresponding to only 3 of the 7 blades of the 7-bladed β-propeller fold (the core structure of canonical UVR8), and Ulvmu_UM015_0123.1 has a C-terminal extension of approximately 320 amino acids located outside this conserved fold (Figure 1A and Supplemental Figure 8A). These findings demonstrate the limitations of sequence-based phylogeny in resolving deeply divergent orthologs and the enhanced accuracy of structure-guided approaches for ortholog assignment.

A well-supported monophyletic BIM1 orthogroup was present in the structure-guided phylogeny (Figure 1B). Noting that BIM1 harbors more intrinsically disordered regions (IDRs), we quantified the proportion of disordered residues across all analyzed sequences and found that it was significantly higher in the BIM1 orthogroup than in the other two major bHLH clades (Supplemental Figures 8B and 8C). To evaluate the phylogenetic contribution of these regions, we constructed a phylogenetic tree with IDRs excluded. Although the overall topology remained largely congruent, the inclusion of IDRs provided substantially higher bootstrap support for the BIM1 clade and was essential for accurate phylogenetic placement of algal lineages (Supplemental Figure 9). For BES1 orthologs, structure-guided phylogenies resolved three major clades (A, B, and C), consistent with sequence-based classifications (Furuya et al., 2024; Figure 1C and Supplemental Figure 6). Comparative analysis of the BES1-N domain (the DNA-binding domain) revealed that clade C exhibits distinct structural features in the DNA-binding region relative to clades A and B, particularly in the orientation of the two α-helices (Supplemental Figure 10A and 10B). This divergence resulted from a conserved RERRRR motif in clades A and B, whereas an RERxRR variant occurred in clade C. This structural divergence between different clades of BES1 orthologs was observed in both liverworts (*Marchantia polymorpha*) and monocots (*Oryza sativa*) (Supplemental Figures 10A and 10B). We found that the presence of RERRRR or RERxRR differentially shapes the electrostatic potential of the BES1-N DNA-binding surface and underlies clade-specific DNA-binding specificities (Supplemental Figures 10C and 10D). Ancestral sequence reconstruction further indicated that the divergence of the BES1-N domain originated in the LCA of the BES1 ortholog (Supplemental Figure 10E), supporting an early splitting event that established the distinct DNA-binding specificities of the BES1 clades (Nosaki et al., 2022).

### Stepwise establishment of protein–protein interactions among UVR8, BES1, and BIM1 in green plants

**Figure 2:**
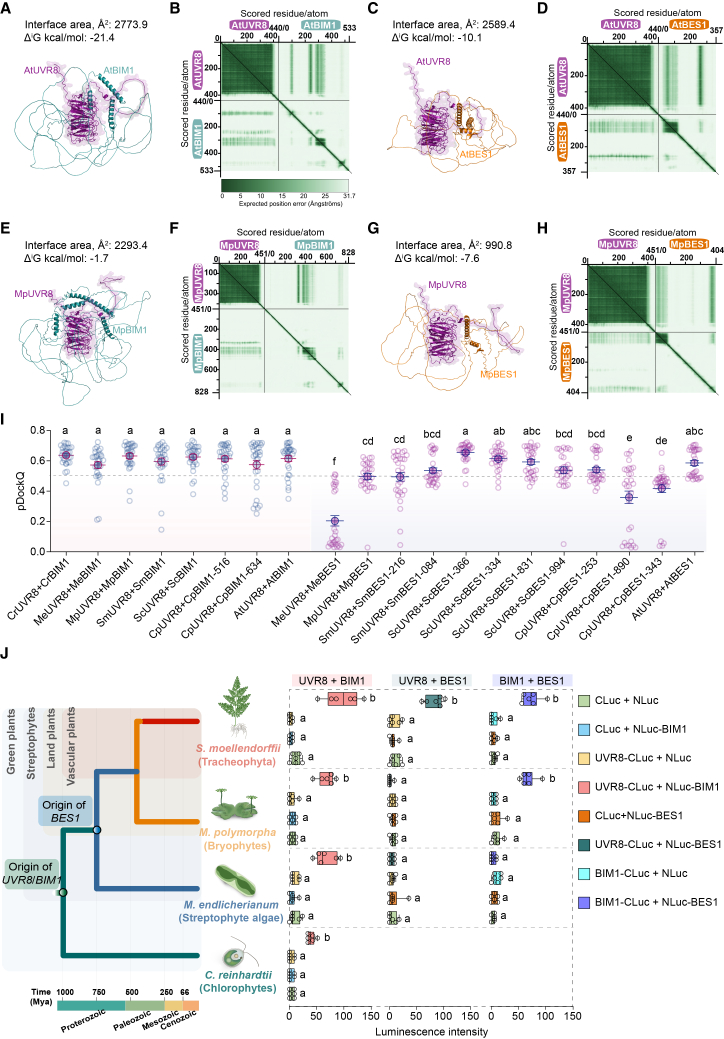
Stepwise establishment of protein–protein interactions among UVR8, BES1, and BIM1 orthologs across green plants. **(A–H**) Examples of predicted structures and predicted alignment error (PAE) heatmaps for UVR8–BIM1 and UVR8–BES1 dimers in *A*. *thaliana***(A–D)** and *M*. *polymorpha***(E–H)**. Dark green regions in the interaction quadrants of PAE denote low expected position error, indicative of protein–protein interactions. Δ^i^G indicates the solvation free energy gain upon formation of the interface (in kcal mol⁻¹); lower Δ^i^G values and larger interface areas indicate a higher likelihood of physical interaction. (**I**) pDockQ scores of UVR8–BIM1 and UVR8–BES1 across species. Each dot represents one prediction replicate. pDockQ ≥ 0.5 indicates high-confidence interaction predictions. (**J**) Luciferase complementation assays confirming interactions among UVR8, BES1, and BIM1 orthologs from *C*. *reinhardtii*, *M*. *endlicherianum*, *M*. *polymorpha*, and *S*. *moellendorffii*. nLUC and cLUC refer to the N- and C-terminal fragments of luciferase, respectively. Data are presented as means ± SD (*n* = 6). Phylogenetic relationships and divergence time estimates of major plant lineages are based on Bowman (2022). Different letters indicate significant differences (Student’s *t*-test, *p* < 0.05).

We next investigated the potential for direct physical interactions among components of the UVR8–BES1/BIM1 module in green plants. Using SpeedPPI (Bryant et al., 2022; Bryant and Noé, 2023), we evaluated the predicted docking quality (pDockQ scores) and local structure confidence (predicted Local Distance Difference Test, pLDDT scores) of UVR8–BES1 and UVR8–BIM1 complexes in representative species from green algae to land plants. In *A*. *thaliana*, both the AtUVR8–AtBES1 and AtUVR8–AtBIM1 complexes exhibited low interchain predicted alignment errors (PAEs) and high pDockQ scores (average 0.588 and 0.614, respectively). Given that pDockQ values above 0.5 are indicative of high-confidence PPIs, these results supported the formation of stable AtUVR8–AtBES1 and AtUVR8–AtBIM1 complexes, consistent with previous experimental results (Liang et al., 2018; Figures 2A–2D). Predicted protein structures from other plants exhibited high structural confidence (pLDDT ≥ 70) (Figure 2E–2I and Supplemental Figures 11A and 11B), enabling the evaluation of UVR8–BIM1 interactions. These interactions appeared to be conserved from the green alga *Chlamydomonas reinhardtii* to angiosperms, as evidenced by high pDockQ scores (Figures 2E, 2F, and 2I). Analyses of evolutionary rate covariation (ERC) also supported the functional association and coevolution of UVR8 and BIM1 throughout the evolution of green plants (Supplemental Figure 12). By contrast, the UVR8–BES1 complex exhibited low pDockQ scores in the streptophyte alga *Mesotaenium endlicherianum* (average pDockQ = 0.18) and the bryophyte *M*. *polymorpha* (average pDockQ = 0.49), indicating limited potential for stable interactions (Figures 2G–2I and Supplemental Figures 11C and 11D). In vascular plants, including the lycophyte *Selaginella moellendorffii* (average pDockQ = 0.53), the fern *Salvinia cucullata* (average pDockQ = 0.64), and seed plants (*Cycas panzhihuaensis*, average pDockQ = 0.56; *A*. *thaliana* 0.58), UVR8–BES1 complexes showed markedly enhanced interaction signals (Figure 2I; Supplemental Figures 11C and 11D). These findings suggest that the capacity for direct UVR8–BES1 interactions is likely greater in vascular plants than in bryophytes.

Because the extensive IDRs in both the BES1 and BIM1 proteins might obscure interface predictions, we sought to experimentally validate the observed PPI patterns. Luciferase complementation imaging (LCI) assays in *Nicotiana benthamiana* confirmed that BIM1 orthologs from green algae to angiosperms specifically interacted with UVR8 (Figure 2J), supporting conservation of the UVR8–BIM1 complex. By contrast, MpBES1 from *M*. *polymorpha* did not directly interact with MpUVR8 but was able to interact with MpBIM1 (Figure 2J). In the lycophyte *S*. *moellendorffii*, direct SmUVR8–SmBES1 interactions were detected, suggesting that UVR8 acquired the ability to directly modulate BES1 function independently of BIM1 (Figure 2J). These results support an evolutionary model in which BES1 initially participated in UV-B signaling indirectly through BIM1-mediated bridging (UVR8–BIM1–BES1) and subsequently gained the ability to directly interact with UVR8 (UVR8–BES1) during the evolution of vascular plants.

### Distinct patterns of UV-B-induced transcriptional regulation between green algae and land plants

**Figure 3:**
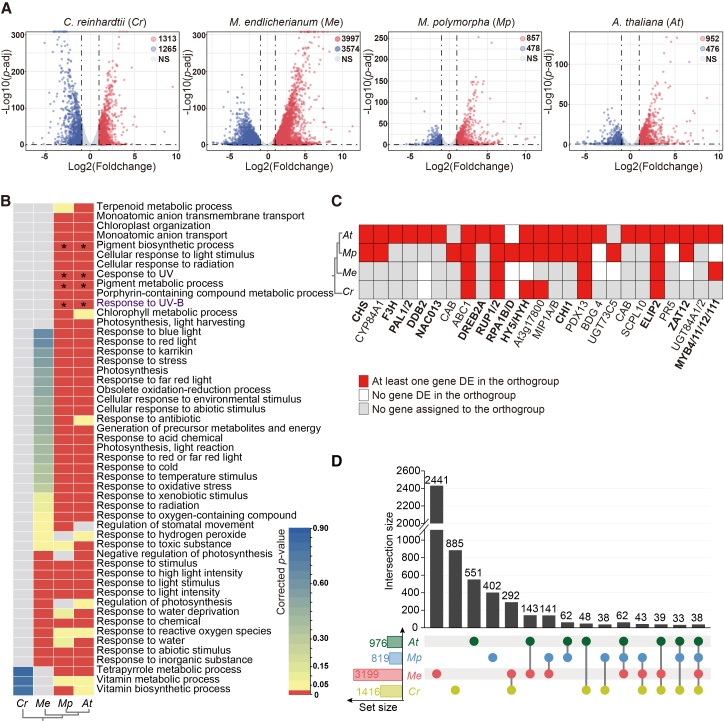
Transcriptomic comparisons of UV-B-responsive genes across representative species of green plants. **(A)** Volcano plots showing transcriptional responses to UV-B treatment in *C*. *reinhardtii*, *M*. *endlicherianum*, *M*. *polymorpha*, and *A*. *thaliana*. *p* adj. < 0.05 and |log_2_FC| ≥ 1 were used as significance thresholds. Blue dots represent significantly downregulated genes, red dots represent significantly upregulated genes, and gray dots represent genes whose expression did not change significantly. **(B)** GO enrichment analysis of UV-B-responsive DEGs, with red indicating significantly enriched terms (*p* adj. < 0.05). Asterisks indicate GO terms associated with UV-B responses. **(C)** Distribution of orthologous genes from UV-B-responsive terms across species. **(D)** UpSet plot showing the overlap of orthologous genes identified from UV-B-responsive DEGs across the four species (vertical bars) and the total number of genes per species (horizontal bars).

To investigate the evolution of expression patterns in response to UV-B, we performed comparative transcriptome analyses of four representative green plants: *C*. *reinhardtii* (Tilbrook et al., 2016), *M*. *endlicherianum*, *M*. *polymorpha*, and *A*. *thaliana* (Tavridou et al., 2020). UV-B treatment induced widespread transcriptional reprogramming in all four species, but the extent and direction of gene expression changes varied among the species (Figure 3 and Supplemental Figures 14–16A). The number of UV-B-responsive genes was highest in *M*. *endlicherianum* (3997 upregulated and 3574 downregulated), followed by *C*. *reinhardtii* (1313 upregulated and 1265 downregulated), *A*. *thaliana* (952 upregulated and 476 downregulated), and *M*. *polymorpha* (857 upregulated and 478 downregulated) (Figure 3A and Supplemental Table 1). In *C*. *reinhardtii*, similar numbers of genes were up- and downregulated in response to UV-B. In *M*. *endlicherianum*, although comparable numbers of genes were up- and downregulated, the upregulated genes tended to exhibit greater significance than the downregulated genes (Figure 3A). This trend resembled the transcriptional activation bias observed in land plants, in which upregulated genes evidently outnumber downregulated genes by approximately twofold, as seen in both *M*. *polymorpha* and *A*. *thaliana*. Given that *M*. *endlicherianum* represents the phylogenetically closest streptophyte algal lineage to land plants, this pattern may reflect an intermediate evolutionary state of UV-B-responsive transcriptional regulation between algae and land plants (Goldbecker and de Vries, 2025; Hess et al., 2022; Figure 3A). Gene Ontology (GO) analysis of these differentially expressed genes (DEGs) identified significantly enriched GO terms, including response to UV-B, response to UV, pigment biosynthetic process, and pigment metabolic process, in both *M. polymorpha* and *A. thaliana* (these terms were not enriched in algal DEGs) (Figure 3B, Supplemental Figure 17, and Supplemental Table 2). Further examination of genes associated with UV-B-responsive GO terms revealed that *PDX1.3* (pyridoxine biosynthesis 1.3), which is involved in vitamin B6 biosynthesis, and *ELIP2* (early light-inducible protein 2) were conserved across all four species, suggesting that they may serve as an ancestral stress response mechanism. By contrast, flavonoid biosynthesis genes, specifically *CHS* (chalcone synthase) and *CHI1* (chalcone isomerase 1), were robustly induced in both *M*. *polymorpha* and *A*. *thaliana* (Figure 3C). Reverse transcription quantitative PCR (RT–qPCR) assays also confirmed the strong UV-B-induced activation of *MpERF11*, *MpCHS*, and *MpPAL* (phenylalanine ammonia-lyase) in *Marchantia* (Supplemental Figure 16B). Originating in the LCA of land plants, these genes appear to have been recruited into the UV-B signaling network to provide critical compounds for UV protection and have been conserved (Tanaka et al., 2008; de Vries et al., 2021; Davies et al., 2024). Orthogroup clustering revealed a comparable set of UV-B-responsive orthologs in *M*. *polymorpha* (819) and *A*. *thaliana* (976) (Figure 3D and Supplemental Table 3). Pairwise comparisons between land plants revealed a convergence degree >0.2 for UV-B-responsive gene orthologs, which was significantly higher than that of the alga–land plant pairs (Supplemental Figure 18). These results highlight distinct patterns of transcriptional reprogramming in response to UV-B between green algae and land plants.

### Evolutionary expansion of UV-B regulatory networks

**Figure 4:**
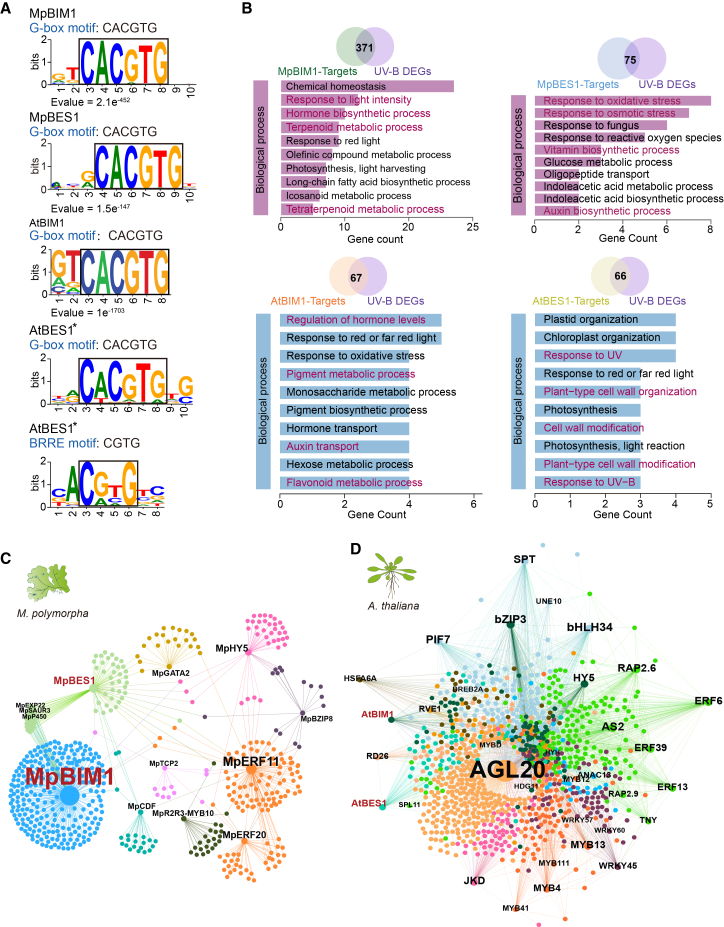
Diversification of *cis*-regulatory control of BES1/BIM1 in the UV-B response. **(A)** Top enriched DNA motifs in BIM1- and BES1-binding regions. AtBES1 data were obtained from a previous study by Yu et al. **(B)** GO enrichment analysis of overlapping genes between BIM1/BES1 targets and UV-B-responsive DEGs. Terms potentially associated with UV-B responses are highlighted in purple. **(C and D)** High-confidence gene regulatory networks inferred from UV-B-responsive DEGs in *M*. *polymorpha***(C)** and *A*. *thaliana***(D)**. Different colors denote different modules.

Given that BIM1 and BES1 were identified as core UV-B-responsive factors, we performed DNA affinity purification sequencing (DAP-seq) analyses of MpBES1, MpBIM1, and AtBIM1 for further comparison. Motif analysis revealed that the canonical G-box motif (CACGTG), a conserved bHLH *cis*-element (Toledo-Ortiz et al., 2003), was significantly enriched in the binding regions of MpBES1, MpBIM1, and AtBIM1, consistent with observations for AtBES1 (Figure 4A). However, the BRRE (CGTG^T^/_C_G), a hallmark of AtBES1 targets (Yu et al., 2011), was not significantly enriched in MpBES1-bound regions (Supplemental Table 4). We next filtered peaks within the 2-kb upstream regions of annotated genes and identified 1133 target genes (TGs) for MpBES1, 3690 TGs for MpBIM1, and 963 TGs for AtBIM1 (Supplemental Figure 19 and Supplemental Table 5). By comparing these TGs with UV-B-responsive gene expression profiles, we quantified the regulatory contribution of each TF. MpBIM1 exhibited the most extensive regulatory engagement, with 10.05% (371/3690) of its TGs responding to UV-B signaling. The corresponding percentages for MpBES1, AtBIM1, and AtBES1 were lower: 6.62% (75/1133), 6.96% (67/963), and 5.72% (66/1154), respectively (Figure 4B). GO enrichment analysis highlighted the conserved regulatory role of BIM1: TGs of both MpBIM1 and AtBIM1 were significantly enriched in “terpenoid metabolism” and “hormone biosynthesis/transport.” By contrast, the function of BES1 appeared to have diverged: the TGs of MpBES1 were primarily associated with general stress responses (e.g., vitamin and carbohydrate metabolism), whereas the TGs of AtBES1 were specifically enriched in “response to UV-B” and “cell wall modification” (Figure 4B).

To gain a holistic view of the UV-B transcriptional regulatory landscape, we constructed high-confidence gene regulatory networks (GRNs) using the UV-B-responsive DEGs. By incorporating the identified TGs into regulatory prediction models, we found that the *M*. *polymorpha* GRN (comprising 10 TFs and 867 edges) was relatively dispersed, with MpBIM1 emerging as a primary hub that directly regulated a large number of targets (Figure 4C and Supplemental Table 6). By contrast, the *A*. *thaliana* GRN (comprising 34 TFs and 3338 edges) had evolved into a highly dense and complex topology. Although AtBIM1 and AtBES1 remained integral components, they were part of a larger regulatory ensemble, with the MADS-box TF AGL20 emerging as a dominant hub (Figure 4D and Supplemental Table 6). Next, we expanded the comparative analysis and constructed predictive GRNs across four species (*C*. *reinhardtii*, *M*. *endlicherianum*, *M*. *polymorpha*, and *A*. *thaliana*) without restricting the TFs to DEGs. The results showed that HY5 and its orthologs were present in all four species, establishing HY5 as an ancient and conserved component of UV-B signaling (Supplemental Figure 20). The GRNs (from *C*. *reinhardtii* and *M*. *endlicherianum* to *M*. *polymorpha* and *A*. *thaliana*) evolved from simple network topologies (comprising only a few TFs, such as ERF and C2H2) to sophisticated, hierarchically layered architectures that incorporated diverse TF families (e.g., DOF, BPC, LBD, GATA, and MYB) (Rieseberg et al., 2025; Supplemental Figure 20).

### Diversified *cis*-regulatory control of BES1/BIM1 in mediation of the UV-B response

**Figure 5:**
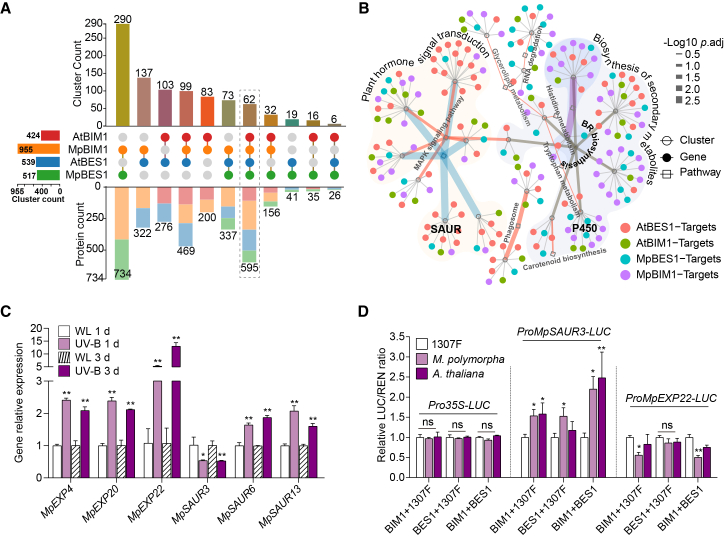
Conservation of BES1/BIM1 regulatory targets across species. **(A)** UpSet plot showing the overlap of DAP-seq targets among MpBIM1, MpBES1, AtBIM1, and AtBES1. Vertical and horizontal bars represent intersection sizes and total orthogroups per TF, respectively. **(B)** KEGG pathway network of shared orthogroups targeted by MpBES1, MpBIM1, AtBIM1, and AtBES1. **(C)** Relative expression levels of *SAUR*/*expansin* family genes under white light (WL) and WL + UV-B treatments in *M. polymorpha*. Data are presented as means ± SD (*n* = 3). **(D)** Dual-luciferase reporter (DLR) assays testing the transcriptional regulation of selected target promoters by BES1 and BIM1. Data are presented as means ± SD (*n* = 6). *p* values were calculated using two-tailed Student’s *t* test. ∗*p* < 0.05; ∗∗*p* < 0.01.

To explore the conservation and divergence of downstream regulatory targets, we compared BES1 and BIM1 TGs between *M*. *polymorpha* and *A*. *thaliana*. We observed numerous overlapping TGs between BIM1 and BES1 within each species (MpBIM1 and MpBES1: 290 orthogroups [OGs], including 734 TGs; AtBIM1 and AtBES1: 103 OGs, including 276 TGs), indicating strong functional interdependence between these two factors (Figure 5A and Supplemental Figure 21). Interestingly, MpBIM1 shared substantially more orthologous TGs with AtBES1 (137 OGs) than with AtBIM1 (83 OGs) (Figure 5A and Supplemental Table 7), indicating a greater regulatory resemblance between MpBIM1 and AtBES1. Moreover, these conserved target clusters consisted predominantly of expanded gene families, including several gene families associated with axillary meristems, auxin, and cell expansion: *RAX3*, *CYP450*, *SAUR*, and *expansin* gene families (Supplemental Table 8; Zeng et al., 2024). Kyoto Encyclopedia of Genes and Genomes (KEGG) analysis of the 62 OGs shared by MpBIM1, MpBES1, AtBIM1, and AtBES1 revealed significantly enriched pathways: plant hormone signal transduction, biosynthesis of secondary metabolites, and BR biosynthesis (Figure 5B and Supplemental Table 9). RT–qPCR analysis showed that several members of these gene families were significantly differentially expressed in response to UV-B treatment. For example, *MpSAUR1*, and *MpSAUR3* were downregulated, whereas *MpSAUR6*, *MpEXP4*, *MpEXP20*, and *MpEXP22* were upregulated (Figure 5C and Supplemental Figure 22). Dual-luciferase reporter (DLR) assays demonstrated that MpBIM1 transcriptionally activated the *MpSAUR3* promoter while repressing the *MpEXP22* promoter in *N*. *benthamiana*. Co-expression with MpBES1 significantly enhanced *MpSAUR3* activation, whereas repression of *MpEXP22* was unaffected (Figure 5D). AtBIM1 could similarly activate *MpSAUR3*, and co-expression with AtBES1 further potentiated this activation. By contrast, the regulation of *MpEXP22* appeared to be specific to MpBIM1, as it was not effectively regulated by AtBIM1 or AtBES1 (Figure 5D). These analyses suggest that MpBIM1, rather than MpBES1, may perform regulatory functions analogous to those of AtBES1 in early-diverging land plants.

## Discussion

During plant terrestrialization, the emergence of core components of UV-B signaling pathways in green algae (chlorophytes and streptophyte algae) facilitated adaptation to the variable UV-B radiation in subaerial environments (Zhang et al., 2022b). The UVR8–BES1/BIM1 signaling module integrates environmental light signals and endogenous phytohormones to coordinate plant growth and development (Liang et al., 2018). In this study, we combined structure-guided phylogeny, comparative transcriptomics, DAP-seq analyses, and experimental validation to uncover the structural innovations and regulatory mechanisms underlying the functional evolution of the UVR8–BES1/BIM1 signaling module in green plants.

Both structure-guided and sequence-based phylogenetic analyses have distinct advantages and limitations in the detection of deep homology. For UVR8, structure-guided homology searches outperformed the sequence-based approach, as the extra domains and incomplete β-propeller folds of this protein often introduced ambiguity when its evolutionary relationships were assessed using sequence-based phylogeny (Figure 1A and Supplemental Figures 1, 3, and 4A). This observation is consistent with a recent report on viral evolution, in which the deep divergence of glycoproteins across the Flaviviridae was resolved using structural information (Mifsud et al., 2024). The BES1 and BIM1 TFs presented challenges due to their extensive IDRs and the inherently low confidence of protein structure predictions in non-model plants. Although the conserved DNA-binding domain of BES1 enabled partial reconstruction of its evolutionary relationships, structure-guided phylogenies provided limited resolution for deep homology of BES1 (Figure 1C and Supplemental Figures 3 and 6). The enrichment of IDRs in BIM1 orthologs limited the ability of structure-guided phylogeny to resolve fine-grained clades (Figure 1B and Supplemental Figure 2). Indeed, algorithms such as FoldMason rely on local structural alignments of ordered regions, which inherently limits their ability to capture evolutionary signals within disordered domains (Gilchrist et al., 2026). Compared with phylogenies based exclusively on functional domains, those that incorporated full-length structural models showed substantially higher bootstrap support values, indicating that structural context improves phylogenetic robustness (Supplemental Figure 9). These results emphasize the necessity of integrating both structural and sequence information to achieve accurate phylogenetic inference (Puente-Lelievre et al., 2025). Our results demonstrated two different cases of structure-guided approaches for deep orthology searches, providing a scalable framework for future large-scale phylogenetic analyses of photoreceptors and TFs in plants.

Both *C*. *reinhardtii* and *M*. *endlicherianum* exhibit broad transcriptional and physiological responses to UV-B signals (Kunz et al., 2026; Tilbrook et al., 2016; Figure 3). Green algae also accumulate photoprotective compounds, including phenolics, mycosporine-like amino acids, and mucilage, which function as “sunscreens” to attenuate UV-B stress (Karsten and Holzinger, 2014; Holzinger et al., 2018; Zhang et al., 2020; Kunz et al., 2024). These findings indicate that the UVR8-mediated transcriptional regulation chassis is deeply rooted in the green lineage. Our protein interaction analyses demonstrated that the UVR8–BIM1 interaction was already established in green algae and has remained highly conserved throughout plant evolution (Figures 1 and 2). Thus, we propose that BIM1 served as one of the early core TFs (such as WRKY3 and HY5) in UV-B signaling. The BES1 ortholog emerged in the LCA of streptophyte algae, but it was unable to interact directly with UVR8 (Figures 1 and 2). In *M*. *polymorpha*, the ancestral UVR8–BIM1 module expanded by recruiting BES1 into the signaling complex through a physical interaction with BIM1. This expansion likely served as an adaptive strategy for terrestrial environments, ensuring a more integrated regulatory network to deal with intense UV-B radiation. During the course of land plant evolution, BES1 acquired a key regulatory motif, the 14-3-3 binding motif, in the LCA of lycophytes (Diao et al., 2023; Supplemental Figure 6). The 14-3-3 binding motif (RxxxSxP) of AtBES1, with a conserved serine (S) residue that serves as a BIN2 phosphorylation site, is critical for the AtUVR8–AtBES1 interaction and BR signal transduction (Gampala et al., 2007; Guo et al., 2024). Correspondingly, we found that the origin of direct UVR8–BES1 interaction coincided with the evolutionary node (lycophytes) at which the preliminary BR signaling pathway arose (Cheon et al., 2013; Ferreira-Guerra et al., 2020). Thus, we inferred that a complex BR-integrated UV-B signaling response evolved at least in the LCA of vascular plants, enhancing the complexity and responsiveness of their UV-B signaling.

The functional diversification of TFs has been a key driver for the expansion of UV-B signaling networks. In *M*. *polymorpha*, both MpBIM1 and MpBES1 bind to the canonical G-box motif (CACGTG), a *cis*-regulatory element recognized by bHLH TFs (Ezer et al., 2017). However, BRREs were not enriched in MpBES1 binding peaks (Figure 4A). This finding is consistent with the reported insensitivity of *M*. *polymorpha* to BR treatment and indicates that canonical BR signaling is not fully established in liverworts (Mecchia et al., 2021). In *A*. *thaliana*, AtBES1 has expanded its DNA-binding specificity and evolved the ability to bind the BRRE motif (CGTG^T^/_C_G), reflecting its dual role in BR and UV-B signaling pathways (Yu et al., 2011; Liang et al., 2018). Structural comparison between AtBES1 and bHLH TFs suggested that the versatility of AtBES1 is enabled by the unique conformation of the BES1-N domain, which permits recognition of both canonical and non-canonical elements (Nosaki et al., 2018). Cross-species transcriptome and DAP-seq comparisons indicated that MpBIM1 acts as the main UV-B-responsive TF in *M*. *polymorpha*, whereas MpBES1, as an auxiliary factor, functions by interacting with MpBIM1 (Figures 3 and 4). This interpretation was also supported by PPI predictions and experimental analyses, which showed that indirect connections between UVR8 and BES1 in bryophytes were established through BIM1 (Figure 2). MpBES1 has been shown to regulate cell division and differentiation in *Marchantia*, reflecting its ancient role in the regulation of plant growth (Mecchia et al., 2021). In angiosperms, BES1 has evolved from a BIM1-dependent co-regulatory component into a dominant regulator of the UV-B response (Liang et al., 2018). By acquiring UVR8-binding capacity and increasing its BR-responsive targets, BES1 gained the ability to coordinate growth and stress signaling in land plants (Liang et al., 2018, 2020). This transition likely involved the co-option of its ancestral growth-related roles into the UV-B signaling network, including the regulation of cell elongation, plant growth, and flavonoid biosynthesis.

**Figure 6:**
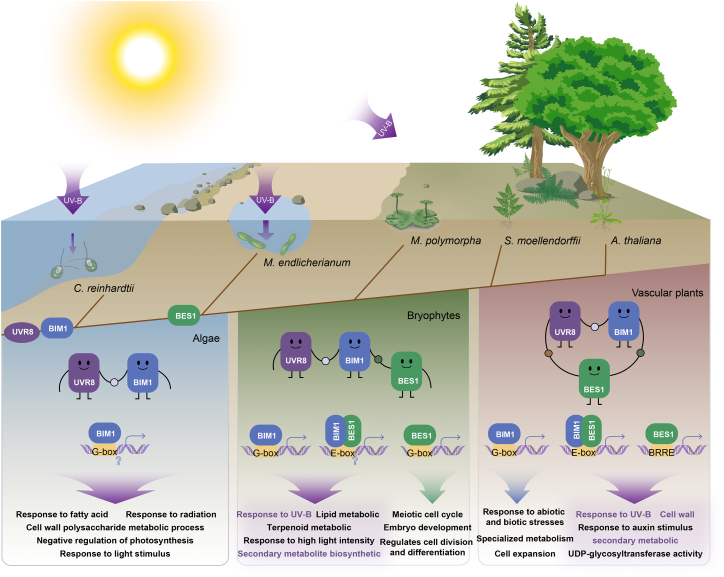
Proposed evolutionary trajectories of the UVR8–BES1/BIM1 signaling module in green plants. The UV-B photoreceptor UVR8 and the transcription factor BIM1 both originated in the ancestor of green plants, whereas BES1 emerged later in streptophytes. The UVR8–BIM1 interaction emerged in green algae, forming an ancient UV-B signaling module. The BIM1–BES1 interaction emerged in liverworts, representing a significant step in the evolution of regulatory complexity. The UVR8–BES1 interaction emerged in lycophytes, enabling integration of UV-B and BR signaling. In green algae and liverworts, BIM1 is one of the core transcription factors in UV-B responses. After expansion and functional innovation of BES1, it became the dominant regulator in vascular plants. Unknown binding motifs are shown with question marks. The schematic is adapted from Martin and Allen (2018) and De Vries and Archibald (2018).

Through multi-layered comparisons across species, we revealed that the UVR8–BES1/BIM1 module evolved through domain acquisition and transcriptional rewiring to meet the complex demands of terrestrial plants. The stepwise assembly of modular signaling components, coupled with a shift from shared to specialized regulatory roles, is a key mechanism underlying the adaptive evolution of UV-B-responsive regulatory networks in green plants (Figure 6). Our study provides a framework for understanding how light signaling networks evolved in green plants, shedding light on the adaptive strategies that confer plasticity and resilience in fluctuating terrestrial environments.

## Methods

### Structure-guided ortholog identification and phylogenetic analyses

The genomes and gene annotations for 99 plant species, covering all major plant lineages, were obtained from public databases, including Phytozome, NCBI, CNCB (China National Center for Bioinformation), FernBase, and published sources (Supplemental Table 10). Sequence-based phylogenetic analyses of *UVR8*, *BES1*, and *BIM1* were performed following the pipeline described by Zhang et al. (2022a, 2022b). In brief, candidate sequences were identified using DIAMOND BLASTp (Buchfink et al., 2021) against *A*. *thaliana* proteins with an e-value cutoff of 1e−5 and validated using InterProScan v5.24 (Jones et al., 2014). Filtered protein sequences were aligned using MAFFT v7.490 (Katoh and Standley, 2013), trimmed with trimAl v1.3 (Capella-Gutierrez et al., 2009), and used to construct maximum-likelihood phylogenetic trees with IQ-TREE v2.4.0 (Minh et al., 2020). ModelFinder (Kalyaanamoorthy et al., 2017) was used to select the best-fit model, and 1000 ultrafast bootstrap replicates were performed.

For structure-guided phylogenetic analyses, all filtered protein sequences were subjected to protein structure prediction using AlphaFold2 (Bryant et al., 2022). For each protein, the model with the highest rank score was retained as the representative structure. Multiple structure alignments were generated using FoldMason v4-dd3c235 (Gilchrist et al., 2026; default parameters), which encodes structures as 3D interaction (3Di) + amino acid (aa) sequences and evaluates alignment quality using LDDT scores in a reference-free manner. The resulting structural alignments were mapped back onto the amino acid sequences, producing structure-informed multiple sequence alignments (MSAs) in FASTA format suitable for direct use in downstream phylogenetic analyses. Protein structures were visualized using PyMOL v3 (Schrödinger, LLC).

### Protein-protein interaction (PPI) prediction and assays

The SpeedPPI pipeline, based on FoldDock and AlphaFold2, was used to predict the structures of the UVR8–BIM1, UVR8–BES1, and BIM1–BES1 dimers (Bryant et al., 2022; Bryant and Noé, 2023). All predictions were performed using all-vs.-all mode with default parameters. Predicted models with pLDDT scores below 70 were excluded, and the pDockQ scores of the remaining models were used to evaluate the likelihood of the PPIs.

LCI assays were performed to validate the PPIs of the UVR8–BIM1, UVR8–BES1, and BIM1–BES1 dimers, as described previously (Chen et al., 2008). In brief, full-length coding sequences (CDSs) of *UVR8*, *BES1*, and *BIM1* from *C*. *reinhardtii*, *M*. *endlicherianum*, *M*. *polymorpha*, and *S*. *moellendorffii* were cloned and inserted into the modified binary vectors pCAMBIA1300-NLuc and pCAMBIA1300-CLuc. *Agrobacterium tumefaciens* strain GV3101 harboring the NLuc- and -CLuc constructs was infiltrated into the abaxial side of 4-week-old *N*. *benthamiana* leaves. After 48 h, leaves were sprayed with 1 mM D-luciferin (40901ES01; Yeasen, Shanghai, China) containing 0.01% Triton X-100, and luminescence images were captured using a chemiluminescence imaging system (Tanon 4100). Luciferase activity was quantified using a dual-luciferase reporter assay kit (DL101; Vazyme, Nanjing, China) according to the manufacturer’s instructions.

### ERC analyses of the UVR8–BIM1 gene family

ERC was analyzed using the CovER pipeline in PhyKIT v1.11.12 (Steenwyk et al., 2021). For the UVR8–BIM1 pair, gene trees were pruned to retain shared taxa, and branch lengths were re-estimated using the fixed topology of the species tree. Pearson’s correlation coefficient (*R*) between branch lengths was calculated, with *R* > 0 and an adjusted *p* value (*p*adj) < 0.05 considered indicative of significant coevolution.

### Ancestral sequence reconstruction of the BES1 gene family

The sequence-based BES1 phylogenetic tree and corresponding multiple sequence alignment were used as inputs for ancestral sequence reconstruction, which was performed using CodeML in PAMLX under the empirical+F model (Xu and Yang, 2013).

### Plant materials and growth conditions

*Mesotaenium endlicherianum* (SAG 12.97) was cultivated in SFM+NH4 medium prepared according to the protocol provided by the Central Collection of Algal Cultures (CCAC), University of Duisburg–Essen. Algae were maintained at 20°C under a 16-h (∼650 μW cm^−2^)/8-h light/dark cycle in a growth chamber. *Marchantia polymorpha* L. accession Takaragaike-1 (Tak-1, male) was cultured on half-strength Gamborg’s B5 medium with 1% (w/v) agar at 22°C under a 16-h (∼1000 μW cm^−2^)/8-h light/dark cycle in an incubator (TCC-20, Wuhan Ruihua Appliance Limited Company, China) (Liu et al., 2024).

For UV-B acclimation, 20-day-old algal cells and 10-day-old gemmalings were exposed to narrowband UV-B (70 μW cm^−2^; Philips TL20W/01RS) supplemented with white light at intensities of 650 μW cm^−2^ and 1000 μW cm^−2^, respectively. For UV-B stress, cells were exposed to broadband UV-B (200 μW cm^−2^; Philips TL20W/12RS) combined with 650 μW cm^−2^ white light (Tilbrook et al., 2016). Control samples were maintained under corresponding white-light conditions. All treatments were performed with three biological replicates.

### RNA-seq and RT–qPCR analyses

Total RNA was extracted from *M*. *endlicherianum* and *M*. *polymorpha* samples collected 3 h after UV-B treatment (70 μW cm^−2^) and from untreated controls. Three biological replicates were prepared for each treatment. RNA sequencing (RNA-seq) libraries were constructed and sequenced by Novogene Bioinformatics Technology Co., Ltd. (Beijing, China). Paired-end sequencing (150 bp) was performed using the Illumina NovaSeq 6000 platform. RNA-seq data analysis, including quality control, read alignment, quantification, normalization, and differential expression analysis, was performed as described previously (Diao et al., 2023). The reference genome assemblies and gene annotations for *M*. *endlicherianum* (v2) and *M*. *polymorpha* (v7.1) were used for read alignment and downstream analyses (Cheng et al., 2019; Dadras et al., 2023; Tanizawa et al., 2025). Significant DEGs were defined using a threshold of |log_2_ fold change (FC)| ≥ 1 and *p* adj. ≤ 0.05. The RNA-seq data for *C. reinhardtii* and *A*. *thaliana* were obtained from published studies and reanalyzed in this work (Tilbrook et al., 2016; Tavridou et al., 2020). Orthologous genes across species were identified and clustered using OrthoFinder v2.5.4 (Emms and Kelly, 2019). UV-B-responsive orthologs were analyzed to assess the conservation of transcriptional responses across species. The degree of conservation was quantified using the “convergence degree,” calculated as |A∩B|/min(|A|, |B|), where |A| and |B| represent the numbers of DEGs in species A and B, respectively (Wu et al., 2022). Volcano plots and GO enrichment analyses were performed using custom R scripts and relevant packages.

To confirm the expression patterns of UV-B-responsive DEGs, total RNA extraction, cDNA synthesis, qPCR, and relative expression analysis were performed as described previously (Livak and Schmittgen, 2001). Gene-specific primers used for RT–qPCR are listed in Supplemental Table 11. *MpACT7* was used as the internal reference gene.

### Analysis of gene regulatory networks

UV-B-responsive GRNs based on UV-B-responsive DEGs were reconstructed for *C*. *reinhardtii*, *M*. *endlicherianum*, *M*. *polymorpha*, and *A*. *thaliana*. High-confidence regulatory pairs were obtained from PlantRegMap (Tian et al., 2020), including both experimentally validated interactions and motif-based predictions. These pairs were filtered to ensure that both the TFs and their TGs were present in the UV-B DEG dataset. For motif-based predictions, the promoter region of each DEG, defined as the 1000-bp sequence upstream of its transcription start site, was scanned for TF binding motifs using FIMO (Find Individual Motif Occurrences) (Grant et al., 2011). Motif occurrences with *p* adj. < 0.05 were considered to indicate putative TF–target interactions and were incorporated into the regulatory network. All GRNs were visualized and analyzed using Gephi (Bastian et al., 2009).

### DNA affinity purification sequencing (DAP-seq) analyses

DAP-seq assays were conducted by Gene Denovo Biotechnology Co., Ltd. (Guangzhou, China) following a modified protocol based on Bartlett et al. (2017). In brief, genomic DNA was extracted from *M*. *polymorpha* and *A*. *thaliana* and used to prepare sequencing libraries with the NEBNext DNA Library Prep Master Mix Set for Illumina (E6040S; NEB, Beijing, China). The CDSs of *MpBIM1*, *MpBES1*, and *AtBIM1* were cloned into expression vectors carrying a Halo-tag, and recombinant proteins were expressed *in vitro* using the TNT SP6 High-Yield Wheat Germ Protein Expression System (L3260; Promega, Beijing, China). After incubation with the DNA library, protein–DNA complexes were captured using magnetic beads, and nonspecific fragments were removed through multiple washing steps. The enriched DNA was purified and subjected to paired-end sequencing (150 bp) on the Illumina HiSeq 4000 platform. Sequencing reads were quality filtered and mapped to the *M*. *polymorpha* and *A*. *thaliana* reference genomes using Bowtie2 v2.2.5 (Langmead and Salzberg, 2012), allowing up to two mismatches. Peak calling was performed with MACS2 v2.2.7.1 (Feng et al., 2012), and peak-associated genes were annotated with ChIPseeker (Yu et al., 2015). Motif enrichment analysis was performed using MEME (Multiple Expectation maximizations for Motif Elicitation) and DREME (Discriminative Regular Expression Motif Elicitation) in the MEME Suite (Bailey et al., 2015; https://meme-suite.org/meme/). GO enrichment analysis was performed using custom R scripts and relevant packages.

Chromatin immunoprecipitation sequencing data for AtBES1 were obtained from Yu et al. Cross-species comparison of BIM1 and BES1 TG sets between *M*. *polymorpha* and *A*. *thaliana* was performed using OrthoVenn3 (Sun et al., 2023). KEGG pathway analysis was conducted primarily on the basis of the *Arabidopsis* dataset, and the enrichment network was generated using cirFunMap (Bu et al., 2021). Subsequently, genes from each *M*. *polymorpha* cluster were mapped to their *A*. *thaliana* orthologs using an R script, followed by visualization of the corresponding KEGG networks.

### Dual-luciferase reporter (DLR) assay

To examine the regulatory activity of MpBIM1 and MpBES1 on their downstream target promoters, the CDSs of *MpBIM1* and *MpBES1* were cloned into the pCAMBIA1307-FLAG vector as effectors. Promoter regions (∼1.5 kb upstream) of the TGs were inserted into the pGreenII 0800-LUC vector to generate reporter constructs. Effector and reporter plasmids were co-infiltrated into tobacco leaves using *A*. *tumefaciens*-mediated transient expression as described previously (Cao et al., 2024). Firefly and *Renilla* luciferase activities were measured using a DLR assay kit (DL101; Vazyme, Nanjing, China). Firefly luciferase activity was normalized to *Renilla* luciferase activity, and relative LUC/REN ratios were calculated. Each assay was performed with three biological replicates and was repeated independently at least twice.

## Data and code availability

The raw RNA-seq and DAP-seq data have been deposited in the Genome Sequence Archive (Genomics, Proteomics, and Bioinformatics, 2021) at the National Genomics Data Center (Nucleic Acids Research, 2025) under BioProject no. PRJCA043849. The sequence alignment data and phylogenetic tree files have been deposited in Figshare (https://figshare.com/s/19af86adfe1c5a923c81).

## Funding

This work was supported by the 10.13039/501100001809National Natural Science Foundation of China (W2511024 and 32370228) (B.Z.), the 10.13039/501100001809National Natural Science Foundation of China (32470232 and 32100178) (Z.Z.), the 10.13039/501100004608Natural Science Foundation of Jiangsu Province (BK20250004), the Collaborative Innovation Center for Modern Crop Production co-sponsored by Province and Ministry (B.Z.), and the 10.13039/501100012246Priority Academic Program Development of Jiangsu Higher Education Institutions (B.Z.).

## Acknowledgments

We thank Hongtao Liu for kindly providing the *AtBES1-FLAG* plasmid. The authors declare no competing interests.

## Author contributions

B.Z. and Z.Z. conceived and designed the study. C.C. performed all experiments and analyses unless otherwise specified, including structural modeling and phylogenetic analysis, most molecular experiments, and DAP-seq analyses. R.D. contributed to RNA-seq analyses. M.Z. and J.W. assisted with protein interaction assays. Q.J. performed the UV-B adaptation experiments in *M*. *endlicherianum*. Z.X. supported the AlphaFold2 modeling. W.X. and Y.Z. performed the UV-B adaptation experiments in *M*. *polymorpha*. Z.Z. and C.C. drafted the manuscript. B.Z. revised and edited the manuscript. All authors read and approved the final paper.

## Supplemental information

Document S1. Supplemental Figures 1–22

Supplemental Table 1. UV-B-responsive DEGs

Supplemental Table 2. GO enrichment analysis of UV-B-responsive DEGs

Supplemental Table 3. Cross-species orthogroups of UV-B-responsive genes

Supplemental Table 4. Enriched motifs identified in DAP-seq binding peaks of MpBIM1 and MpBES1

Supplemental Table 5. DAP-seq binding peaks identified for MpBIM1, MpBES1, and AtBIM1

Supplemental Table 6. Transcriptional regulatory network underlying UV-B response

Supplemental Table 7. GO enrichment analysis of BIM1 and BES1 target genes and their overlap with UV-B-responsive DEGs

Supplemental Table 8. Conserved orthogroups of AtBES1, AtBIM1, MpBES1, and MpBIM1 target genes

Supplemental Table 9. KEGG pathway enrichment network of conserved targets of AtBES1, AtBIM1, MpBES1, and MpBIM1

Supplemental Table 10. Species list for phylogenomic analysis

Supplemental Table 11. List of primers used in this study

Document S2. Article plus supplemental information

## References

[bib1] Bailey T.L., Johnson J., Grant C.E., Noble W.S. (2015). The MEME Suite. Nucleic Acids Res..

[bib84] Bartlett A., O’Malley R.C., Huang S.C., Galli M., Nery J.R., Gallavotti A., Ecker J.R. (2017). Mapping genome-wide transcription-factor binding sites using DAP-seq. Nat. Protoc.

[bib2] Bastian M., Heymann S., Jacomy M. (2009). Gephi: An Open Source Software for Exploring and Manipulating Networks. Proceedings of the International AAAI Conference on Web and Social Media.

[bib3] Bowman J.L.J.N.P. (2022). The origin of a land flora. Nat. Plants.

[bib4] Bryant P., Noé F. (2023). Rapid protein-protein interaction network creation from multiple sequence alignments with Deep Learning. bioRxiv.

[bib5] Bryant P., Pozzati G., Elofsson A. (2022). Improved prediction of protein-protein interactions using AlphaFold2. Nat. Commun..

[bib6] Bu D., Luo H., Huo P., Wang Z., Zhang S., He Z., Wu Y., Zhao L., Liu J., Guo J. (2021). KOBAS-i: intelligent prioritization and exploratory visualization of biological functions for gene enrichment analysis. Nucleic Acids Res..

[bib7] Buchfink B., Reuter K., Drost H.G. (2021). Sensitive protein alignments at tree-of-life scale using DIAMOND. Nat. Methods.

[bib8] Cao C., Guo S., Deng P., Yang S., Xu J., Hu T., Hu Z., Chen D., Zhang H., Navea I.P. (2024). The BEL1-like homeodomain protein OsBLH4 regulates rice plant height, grain number, and heading date by repressing the expression of OsGA2ox1. Plant J..

[bib9] Capella-Gutiérrez S., Silla-Martínez J.M., Gabaldón T. (2009). trimAl: a tool for automated alignment trimming in large-scale phylogenetic analyses. Bioinformatics.

[bib10] Chen H., Zou Y., Shang Y., Lin H., Wang Y., Cai R., Tang X., Zhou J.M. (2008). Firefly luciferase complementation imaging assay for protein-protein interactions in plants. Plant Physiol..

[bib11] Cheng S., Xian W., Fu Y., Marin B., Keller J., Wu T., Sun W., Li X., Xu Y., Zhang Y. (2019). Genomes of Subaerial Zygnematophyceae Provide Insights into Land Plant Evolution. Cell.

[bib12] Cheon J., Fujioka S., Dilkes B.P., Choe S. (2013). Brassinosteroids regulate plant growth through distinct signaling pathways in Selaginella and Arabidopsis. PLoS One.

[bib13] Dadras A., Fürst-Jansen J.M.R., Darienko T., Krone D., Scholz P., Sun S., Herrfurth C., Rieseberg T.P., Irisarri I., Steinkamp R. (2023). Environmental gradients reveal stress hubs pre-dating plant terrestrialization. Nat. Plants.

[bib14] Davies K.M., Andre C.M., Kulshrestha S., Zhou Y., Schwinn K.E., Albert N.W., Chagné D., van Klink J.W., Landi M., Bowman J.L. (2024). The evolution of flavonoid biosynthesis. Philos. Trans. R. Soc. Lond. B Biol. Sci..

[bib15] de Vries J., Archibald J.M. (2018). Plant evolution: landmarks on the path to terrestrial life. New Phytol..

[bib16] de Vries S., Fürst-Jansen J.M.R., Irisarri I., Dhabalia Ashok A., Ischebeck T., Feussner K., Abreu I.N., Petersen M., Feussner I., de Vries J. (2021). The evolution of the phenylpropanoid pathway entailed pronounced radiations and divergences of enzyme families. Plant J..

[bib17] Delaux P.M., Hetherington A.J., Coudert Y., Delwiche C., Dunand C., Gould S., Kenrick P., Li F.W., Philippe H., Rensing S.A. (2019). Reconstructing trait evolution in plant evo-devo studies. Curr. Biol..

[bib18] Diao R., Zhao M., Liu Y., Zhang Z., Zhong B. (2023). The advantages of crosstalk during the evolution of the BZR1-ARF6-PIF4 (BAP) module. J. Integr. Plant Biol..

[bib19] Emms D.M., Kelly S. (2019). OrthoFinder: phylogenetic orthology inference for comparative genomics. Genome Biol..

[bib20] Ezer D., Shepherd S.J.K., Brestovitsky A., Dickinson P., Cortijo S., Charoensawan V., Box M.S., Biswas S., Jaeger K.E., Wigge P.A. (2017). The G-Box Transcriptional Regulatory Code in Arabidopsis. Plant Physiol..

[bib21] Feng J., Liu T., Qin B., Zhang Y., Liu X.S. (2012). Identifying ChIP-seq enrichment using MACS. Nat. Protoc..

[bib22] Fernández M.B., Tossi V., Lamattina L., Cassia R. (2016). A Comprehensive Phylogeny Reveals Functional Conservation of the UV-B Photoreceptor UVR8 from Green Algae to Higher Plants. Front. Plant Sci..

[bib23] Ferreira-Guerra M., Marquès-Bueno M., Mora-García S., Caño-Delgado A.I. (2020). Delving into the evolutionary origin of steroid sensing in plants. Curr. Opin. Plant Biol..

[bib24] Fürst-Jansen J.M.R., de Vries S., de Vries J. (2020). Evo-physio: on stress responses and the earliest land plants. J. Exp. Bot..

[bib25] Furuya T., Saegusa N., Yamaoka S., Tomoita Y., Minamino N., Niwa M., Inoue K., Yamamoto C., Motomura K., Shimadzu S. (2024). A non-canonical BZR/BES transcription factor regulates the development of haploid reproductive organs in Marchantia polymorpha. Nat. Plants.

[bib26] Gampala S.S., Kim T.W., He J.X., Tang W., Deng Z., Bai M.Y., Guan S., Lalonde S., Sun Y., Gendron J.M. (2007). An essential role for 14-3-3 proteins in brassinosteroid signal transduction in Arabidopsis. Dev. Cell.

[bib27] Gilchrist C.L.M., Mirdita M., Steinegger M. (2026). Multiple protein structure alignment at scale with FoldMason. Science.

[bib28] Goldbecker E.S., de Vries J. (2025). Systems Biology of Streptophyte Cell Evolution. Annu. Rev. Plant Biol..

[bib29] Grant C.E., Bailey T.L., Noble W.S. (2011). FIMO: scanning for occurrences of a given motif. Bioinformatics.

[bib30] Guo B., Kim E.J., Zhu Y., Wang K., Russinova E. (2024). Shaping Brassinosteroid Signaling through Scaffold Proteins. Plant Cell Physiol..

[bib31] Han X., Chang X., Zhang Z., Chen H., He H., Zhong B., Deng X.W. (2019). Origin and Evolution of Core Components Responsible for Monitoring Light Environment Changes during Plant Terrestrialization. Mol. Plant.

[bib32] Hayes S., Velanis C.N., Jenkins G.I., Franklin K.A. (2014). UV-B detected by the UVR8 photoreceptor antagonizes auxin signaling and plant shade avoidance. Proc. Natl. Acad. Sci. USA.

[bib33] Hess S., Williams S.K., Busch A., Irisarri I., Delwiche C.F., de Vries S., Darienko T., Roger A.J., Archibald J.M., Buschmann H. (2022). A phylogenomically informed five-order system for the closest relatives of land plants. Curr. Biol..

[bib34] Holzinger A., Albert A., Aigner S., Uhl J., Schmitt-Kopplin P., Trumhová K., Pichrtová M. (2018). Arctic, Antarctic, and temperate green algae Zygnema spp. under UV-B stress: vegetative cells perform better than pre-akinetes. Protoplasma.

[bib35] Jones P., Binns D., Chang H.Y., Fraser M., Li W., McAnulla C., McWilliam H., Maslen J., Mitchell A., Nuka G. (2014). InterProScan 5: genome-scale protein function classification. Bioinformatics.

[bib36] Kalyaanamoorthy S., Minh B.Q., Wong T.K.F., von Haeseler A., Jermiin L.S. (2017). ModelFinder: fast model selection for accurate phylogenetic estimates. Nat. Methods.

[bib37] Karsten U., Holzinger A. (2014). Green algae in alpine biological soil crust communities: acclimation strategies against ultraviolet radiation and dehydration. Biodivers. Conserv..

[bib38] Katoh K., Standley D.M. (2013). MAFFT multiple sequence alignment software version 7: improvements in performance and usability. Mol. Biol. Evol..

[bib39] Kim D., Park S., Steinegger M. (2025). Unicore Enables Scalable and Accurate Phylogenetic Reconstruction with Structural Core Genes. Genome Biol. Evol..

[bib40] Kunz C.F., de Vries S., de Vries J. (2024). Plant terrestrialization: an environmental pull on the evolution of multi-sourced streptophyte phenolics. Philos. Trans. R. Soc. Lond. B Biol. Sci..

[bib41] Kunz C.F., Abreu I.N., Darienko T., Fürst-Jansen J., Feussner K., Feussner I., Lorenz M., de Vries J. (2026). Chemodiverse cell systems responses to UV in an algal sister of land plants. bioRxiv.

[bib42] Langmead B., Salzberg S.L. (2012). Fast gapped-read alignment with Bowtie 2. Nat. Methods.

[bib43] Liang T., Yang Y., Liu H. (2019). Signal transduction mediated by the plant UV-B photoreceptor UVR8. New Phytol..

[bib44] Liang T., Mei S., Shi C., Yang Y., Peng Y., Ma L., Wang F., Li X., Huang X., Yin Y., Liu H. (2018). UVR8 Interacts with BES1 and BIM1 to Regulate Transcription and Photomorphogenesis in Arabidopsis. Dev. Cell.

[bib45] Liang T., Shi C., Peng Y., Tan H., Xin P., Yang Y., Wang F., Li X., Chu J., Huang J. (2020). Brassinosteroid-Activated BRI1-EMS-SUPPRESSOR 1 Inhibits Flavonoid Biosynthesis and Coordinates Growth and UV-B Stress Responses in Plants. Plant Cell.

[bib46] Liu W., Yang Z., Cai G., Li B., Liu S., Willemsen V., Xu L. (2024). MpANT regulates meristem development in Marchantia polymorpha. Cell Rep..

[bib47] Livak K.J., Schmittgen T.D. (2001). Analysis of relative gene expression data using real-time quantitative PCR and the 2(-Delta Delta C(T)) Method. Methods.

[bib48] Maberly S.C. (2014). The fitness of the environments of air and water for photosynthesis, growth, reproduction and dispersal of photoautotrophs: An evolutionary and biogeochemical perspective. Aquat. Bot..

[bib49] Martin W.F., Allen J.F. (2018). An Algal Greening of Land. Cell.

[bib50] Mecchia M.A., García-Hourquet M., Lozano-Elena F., Planas-Riverola A., Blasco-Escamez D., Marquès-Bueno M., Mora-García S., Caño-Delgado A.I. (2021). The BES1/BZR1-family transcription factor MpBES1 regulates cell division and differentiation in Marchantia polymorpha. Curr. Biol..

[bib51] Mifsud J.C.O., Lytras S., Oliver M.R., Toon K., Costa V.A., Holmes E.C., Grove J. (2024). Mapping glycoprotein structure reveals Flaviviridae evolutionary history. Nature.

[bib52] Minh B.Q., Schmidt H.A., Chernomor O., Schrempf D., Woodhams M.D., von Haeseler A., Lanfear R. (2020). IQ-TREE 2: New Models and Efficient Methods for Phylogenetic Inference in the Genomic Era. Mol. Biol. Evol..

[bib53] Morris J.L., Puttick M.N., Clark J.W., Edwards D., Kenrick P., Pressel S., Wellman C.H., Yang Z., Schneider H., Donoghue P.C.J. (2018). The timescale of early land plant evolution. Proc. Natl. Acad. Sci. USA.

[bib54] Nosaki S., Miyakawa T., Xu Y., Nakamura A., Hirabayashi K., Asami T., Nakano T., Tanokura M. (2018). Structural basis for brassinosteroid response by BIL1/BZR1. Nat. Plants.

[bib55] Nosaki S., Mitsuda N., Sakamoto S., Kusubayashi K., Yamagami A., Xu Y., Bui T.B.C., Terada T., Miura K., Nakano T. (2022). Brassinosteroid-induced gene repression requires specific and tight promoter binding of BIL1/BZR1 via DNA shape readout. Nat. Plants.

[bib56] O'Hara A., Jenkins G.I. (2012). In vivo function of tryptophans in the Arabidopsis UV-B photoreceptor UVR8. Plant Cell.

[bib57] Oh E., Zhu J.Y., Wang Z.Y. (2012). Interaction between BZR1 and PIF4 integrates brassinosteroid and environmental responses. Nat. Cell Biol..

[bib58] Oravecz A., Baumann A., Máté Z., Brzezinska A., Molinier J., Oakeley E.J., Adám E., Schäfer E., Nagy F., Ulm R. (2006). CONSTITUTIVELY PHOTOMORPHOGENIC1 is required for the UV-B response in Arabidopsis. Plant Cell.

[bib59] Podolec R., Demarsy E., Ulm R. (2021). Perception and Signaling of Ultraviolet-B Radiation in Plants. Annu. Rev. Plant Biol..

[bib60] Puente-Lelievre C., Malik A., Douglas J. (2025). Protein Structural Phylogenetics. Genome Biol. Evol..

[bib61] Reyero-Saavedra M.d.R., Sánchez Correa M.d.S., Estrella Parra E.A., Espinosa González A.M., Nolasco Ontiveros E., Ortiz-Montiel J.G., Campos Contreras J.E., López Urrutia E., Avila Acevedo J.G., Benítez Flores J.d.C., Oliveira M.T., Fernandes Silva A.A.A. (2023). Abiotic Stress in Plants - Adaptations to Climate Change.

[bib62] Rieseberg T.P., Dadras A., Darienko T., Post S., Herrfurth C., Fürst-Jansen J.M.R., Hohnhorst N., Petroll R., Rensing S.A., Pröschold T. (2025). Time-resolved oxidative signal convergence across the algae-embryophyte divide. Nat. Commun..

[bib63] Rizzini L., Favory J.-J., Cloix C., Faggionato D., O’Hara A., Kaiserli E., Baumeister R., Schäfer E., Nagy F., Jenkins G.I., Ulm R. (2011). Perception of UV-B by the Arabidopsis UVR8 protein. Science.

[bib64] Rozema J., Björn L.O., Bornman J.F., Gaberščik A., Häder D.P., Trošt T., Germ M., Klisch M., Gröniger A., Sinha R.P. (2002). The role of UV-B radiation in aquatic and terrestrial ecosystems—an experimental and functional analysis of the evolution of UV-absorbing compounds. J. Photochem. Photobiol., B.

[bib65] Shi C., Liu H. (2021). How plants protect themselves from ultraviolet-B radiation stress. Plant Physiol..

[bib66] Steenwyk J.L., Buida T.J., Labella A.L., Li Y., Shen X.X., Rokas A. (2021). PhyKIT: a broadly applicable UNIX shell toolkit for processing and analyzing phylogenomic data. Bioinformatics.

[bib67] Sun J., Lu F., Luo Y., Bie L., Xu L., Wang Y. (2023). OrthoVenn3: an integrated platform for exploring and visualizing orthologous data across genomes. Nucleic Acids Res..

[bib68] Tanaka Y., Sasaki N., Ohmiya A. (2008). Biosynthesis of plant pigments: anthocyanins, betalains and carotenoids. Plant J..

[bib69] Tanizawa Y., Mochizuki T., Yagura M., Sakamoto M., Fujisawa T., Kawamura S., Shimokawa E., Yamaoka S., Nishihama R., Bowman J.L. (2025). MarpolBase: Genome database for Marchantia polymorpha featuring high quality reference genome sequences. Plant Cell Physiol..

[bib70] Tavridou E., Pireyre M., Ulm R. (2020). Degradation of the transcription factors PIF4 and PIF5 under UV-B promotes UVR8-mediated inhibition of hypocotyl growth in Arabidopsis. Plant J..

[bib71] Tian F., Yang D.C., Meng Y.Q., Jin J., Gao G. (2020). PlantRegMap: charting functional regulatory maps in plants. Nucleic Acids Res..

[bib72] Tilbrook K., Dubois M., Crocco C.D., Yin R., Chappuis R., Allorent G., Schmid-Siegert E., Goldschmidt-Clermont M., Ulm R. (2016). UV-B Perception and Acclimation in Chlamydomonas reinhardtii. Plant Cell.

[bib73] Toledo-Ortiz G., Huq E., Quail P.H. (2003). The Arabidopsis basic/helix-loop-helix transcription factor family. Plant Cell.

[bib74] Wu T.Y., Hoh K.L., Boonyaves K., Krishnamoorthi S., Urano D. (2022). Diversification of heat shock transcription factors expanded thermal stress responses during early plant evolution. Plant Cell.

[bib75] Xu B., Yang Z. (2013). PAMLX: a graphical user interface for PAML. Mol. Biol. Evol..

[bib76] Yang Y., Liang T., Zhang L., Shao K., Gu X., Shang R., Shi N., Li X., Zhang P., Liu H. (2018). UVR8 interacts with WRKY36 to regulate HY5 transcription and hypocotyl elongation in Arabidopsis. Nat. Plants.

[bib77] Yin Y., Vafeados D., Tao Y., Yoshida S., Asami T., Chory J. (2005). A new class of transcription factors mediates brassinosteroid-regulated gene expression in Arabidopsis. Cell.

[bib78] Yu G., Wang L.G., He Q.Y. (2015). ChIPseeker: an R/Bioconductor package for ChIP peak annotation, comparison and visualization. Bioinformatics.

[bib79] Yu X., Li L., Zola J., Aluru M., Ye H., Foudree A., Guo H., Anderson S., Aluru S., Liu P. (2011). A brassinosteroid transcriptional network revealed by genome-wide identification of BESI target genes in Arabidopsis thaliana. Plant J..

[bib80] Zeng H.Y., Deng S., Jin C., Shang Z., Chang L., Wang J., Han X., Wang A., Jin D., Wang Y. (2024). Origin and evolution of auxin-mediated acid growth. Proc. Natl. Acad. Sci. USA.

[bib81] Zhang Z., Xu C., Zhang S., Shi C., Cheng H., Liu H., Zhong B. (2022). Origin and adaptive evolution of UV RESISTANCE LOCUS 8-mediated signaling during plant terrestrialization. Plant Physiol..

[bib82] Zhang Z., Ma X., Liu Y., Yang L., Shi X., Wang H., Diao R., Zhong B. (2022). Origin and evolution of green plants in the light of key evolutionary events. J. Integr. Plant Biol..

[bib83] Zhang Z.H., Chang X., Su D.Y., Yao R., Liu X.D., Zhu H., Liu G.X., Zhong B.J. (2020). Comprehensive transcriptome analyses of twoOocystisalgae provide insights into the adaptation to Qinghai–Tibet Plateau. J. Syst. Evol..

