# Supplementary material for: Functional evolution and rewiring of the UVR8–BES1/BIM1 module underpin the refinement of UV-B responses during plant terrestrialization
Source: Plant Commun. 2026 Apr 3;7(6):101842. doi: 10.1016/j.xplc.2026.101842 (PMC13261680; doi:10.1016/j.xplc.2026.101842)
Supplement: Document S1. Supplemental Figures 1–22 [file mmc1.pdf]

Plant Communications, Volume *7*

Supplemental information

Functional evolution and rewiring of the UVR8–BES1/BIM1 module underpin the refinement of UV-B responses during plant terrestrialization

Chengjuan Cao, Runjie Diao, Mengru Zhao, Qiuting Ji, Jingwen Wang, Zilong Xu, Wenhui Xie, Yujun Zhou, Zhenhua Zhang, and Bojian Zhong

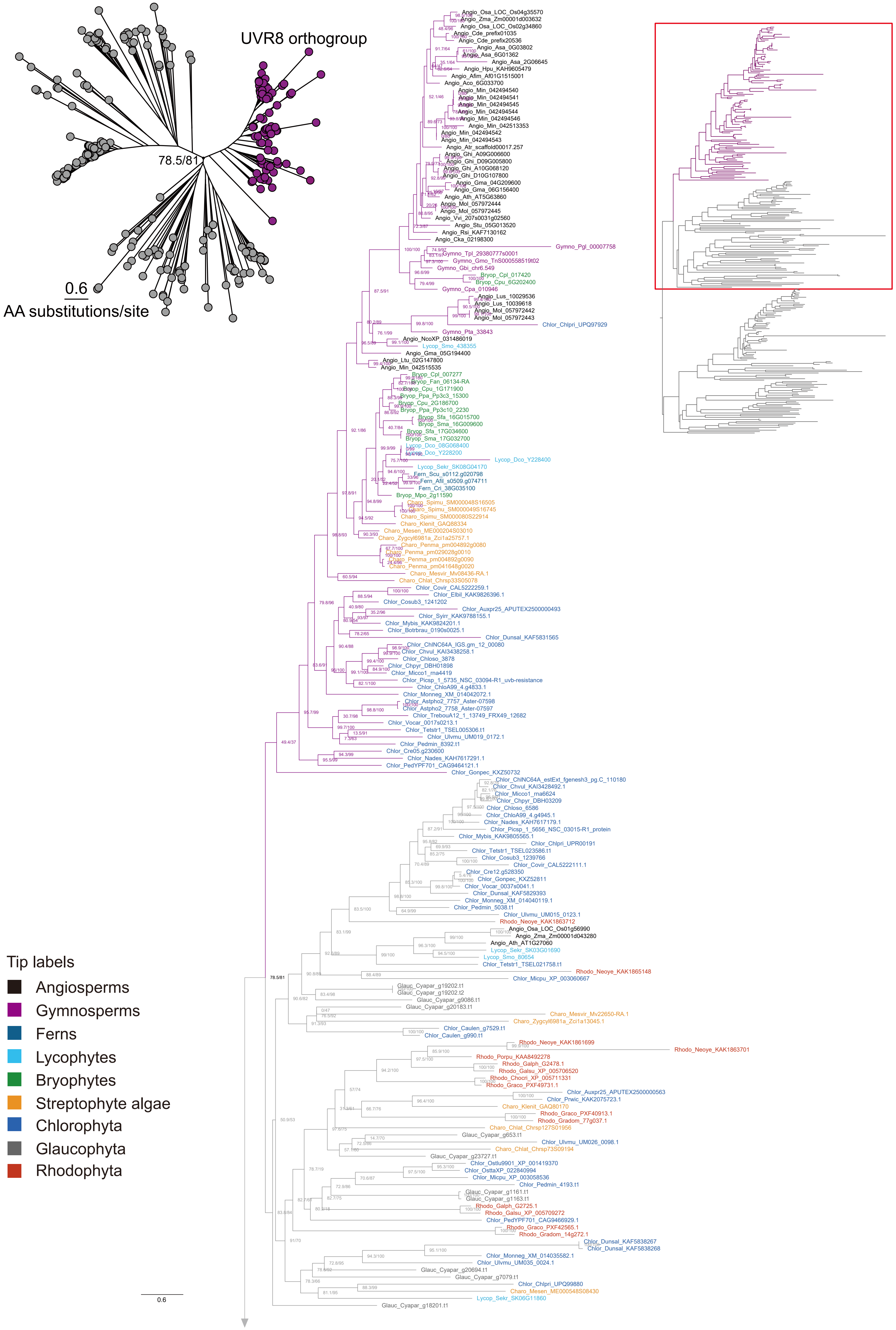

UVR8 orthogroup
78.5/81
0.6
AA substitutions/site
Tip labels
Angiosperms
Gymnosperms
Ferns
Lycophytes
Bryophytes
Streptophyte algae
Chlorophyta
Glaucophyta
Rhodophyta
Angio_Osa_LOC_Os04g35570
Angio_Zma_Zm00001d003632
Angio_Osa_LOC_Os02g34860
Angio_Cde_prefix01035
Angio_Cde_prefix20536
Angio_Asa_0G03802
Angio_Asa_6G01362
Angio_Asa_2G06645
Angio_Hpu_KAH9605479
Angio_Afim_Af01G1515001
Angio_Aco_6G033700
Angio_Min_042494540
Angio_Min_042494541
Angio_Min_042494545
Angio_Min_042494544
Angio_Min_042494546
Angio_Min_042513353
Angio_Min_042494542
Angio_Min_042494543
Angio_Atr_scaffold00017.257
Angio_Ghi_A09G006600
Angio_Ghi_D09G005800
Angio_Ghi_A10G068120
Angio_Ghi_D10G107800
Angio_Gma_04G209600
Angio_Gma_06G156400
Angio_Ath_AT5G63860
Angio_Mol_057972444
Angio_Mol_057972445
Angio_Vvi_207s0031g02560
Angio_Stu_05G013520
Angio_Rsi_KAF7130162
Angio_Cka_02198300
Gymno_Pgl_00007758
Gymno_Tpl_29380777s0001
Gymno_Gmo_TnS000558519t02
Gymno_Gbi_chr6.549
Bryop_Cpl_017420
Bryop_Cpu_6G202400
Gymno_Cpa_010946
Angio_Lus_10029536
Angio_Lus_10039618
Angio_Mol_057972442
Angio_Mol_057972443
Chlor_Chlpri_UPQ97929
Gymno_Pta_33843
Angio_NcoXP_031486019
Lycop_Smo_438355
Angio_Gma_05G194400
Angio_Ltu_02G147800
Angio_Min_042515535
Bryop_Cpl_007277
Bryop_Fan_06134-RA
Bryop_Cpu_1G171900
Bryop_Ppa_Pp3c3_15300
Bryop_Cpu_2G186700
Bryop_Ppa_Pp3c10_2230
Bryop_Sfa_16G015700
Bryop_Sma_16G009600
Bryop_Sfa_17G034600
Bryop_Sma_17G032700
Lycop_Dco_08G068400
Lycop_Dco_Y228200
Lycop_Dco_Y228400
Lycop_Sekr_SK08G04170
Fern_Scu_s0112.g020798
Fern_Afil_s0509.g074711
Fern_Cri_38G035100
Bryop_Mpo_2g11590
Charo_Spimu_SM000048S16505
Charo_Spimu_SM000049S16745
Charo_Spimu_SM000080S22914
Charo_Klenit_GAQ88334
Charo_Mesen_ME000204S03010
Charo_Zygcyl6981a_Zci1a25757.1
Charo_Penma_pm004892g0080
Charo_Penma_pm029028g0010
Charo_Penma_pm004892g0090
Charo_Penma_pm041648g0020
Charo_Mesvir_Mv08436-RA.1
Charo_Chlat_Chrsp33S05078
Chlor_Covir_CAL5222259.1
Chlor_Elbil_KAK9826396.1
Chlor_Cosub3_1241202
Chlor_Auxpr25_APUTEX2500000493
Chlor_Syirr_KAK9788155.1
Chlor_Mybis_KAK9824201.1
Chlor_Botrbrau_0190s0025.1
Chlor_Dunsal_KAF5831565
Chlor_ChlNC64A_IGS.gm_12_00080
Chlor_Chvul_KAI3438258.1
Chlor_Chloso_3878
Chlor_Chpyr_DBH01898
Chlor_Micco1_rna4419
Chlor_Picsp_1_5735_NSC_03094-R1_uvb-resistance
Chlor_ChloA99_4.g4833.1
Chlor_Monneg_XM_014042072.1
Chlor_Astpho2_7757_Aster-07598
Chlor_Astpho2_7758_Aster-07597
Chlor_TrebouA12_1_13749_FRX49_12682
Chlor_Vocar_0017s0213.1
Chlor_Tetstr1_TSEL005306.t1
Chlor_Ulvmu_UM019_0172.1
Chlor_Pedmin_8392.t1
Chlor_Cre05.g230600
Chlor_Nades_KAH7617291.1
Chlor_PedYPF701_CAG9464121.1
Chlor_Gonpec_KXZ50732
Chlor_ChlNC64A_estExt_fgenesh3_pg.C_110180
Chlor_Chvul_KAI3428492.1
Chlor_Micco1_rna6624
Chlor_Chpyr_DBH03209
Chlor_Chloso_6586
Chlor_ChloA99_4.g4945.1
Chlor_Nades_KAH7617179.1
Chlor_Picsp_1_5656_NSC_03015-R1_protein
Chlor_Mybis_KAK9805565.1
Chlor_Chlpri_UPR00191
Chlor_Tetstr1_TSEL023586.t1
Chlor_Cosub3_1239766
Chlor_Covir_CAL5222111.1
Chlor_Cre12.g528350
Chlor_Gonpec_KXZ52811
Chlor_Vocar_0037s0041.1
Chlor_Dunsal_KAF5829393
Chlor_Monneg_XM_014040119.1
Chlor_Pedmin_5038.t1
Chlor_Ulvmu_UM015_0123.1
Rhodo_Neoye_KAK1863712
Angio_Osa_LOC_Os01g56990
Angio_Zma_Zm00001d043280
Angio_Ath_AT1G27060
Lycop_Sekr_SK03G01690
Lycop_Smo_80654
Chlor_Tetstr1_TSEL021758.t1
Rhodo_Neoye_KAK1865148
Chlor_Micpu_XP_003060667
Glauc_Cyapar_g19202.t1
Glauc_Cyapar_g19202.t2
Glauc_Cyapar_g9086.t1
Glauc_Cyapar_g20183.t1
Charo_Mesvir_Mv22650-RA.1
Charo_Zygcyl6981a_Zci1a13045.1
Chlor_Caulen_g7529.t1
Chlor_Caulen_g990.t1
Rhodo_Neoye_KAK1861699
Rhodo_Neoye_KAK1863701
Rhodo_Porpu_KAA8492278
Rhodo_Galph_G2478.1
Rhodo_Galsu_XP_005706520
Rhodo_Chocri_XP_005711331
Rhodo_Graco_PXF49731.1
Chlor_Auxpr25_APUTEX2500000563
Chlor_Prwic_KAK2075723.1
Charo_Klenit_GAQ80170
Rhodo_Graco_PXF40913.1
Rhodo_Gradom_77g037.1
Charo_Chlat_Chrsp127S01956
Glauc_Cyapar_g653.t1
Chlor_Ulvmu_UM026_0098.1
Charo_Chlat_Chrsp73S09194
Glauc_Cyapar_g23727.t1
Chlor_Ostlu9901_XP_001419370
Chlor_OsttaXP_022840994
Chlor_Micpu_XP_003058536
Chlor_Pedmin_4193.t1
Glauc_Cyapar_g1161.t1
Glauc_Cyapar_g1163.t1
Rhodo_Galph_G2725.1
Rhodo_Galsu_XP_005709272
Chlor_PedYPF701_CAG9466929.1
Rhodo_Graco_PXF42565.1
Rhodo_Gradom_14g272.1
Chlor_Dunsal_KAF5838267
Chlor_Dunsal_KAF5838268
Chlor_Monneg_XM_014035582.1
Chlor_Ulvmu_UM035_0024.1
Glauc_Cyapar_g20694.t1
Glauc_Cyapar_g7079.t1
Chlor_Chlpri_UPQ99880
Charo_Mesen_ME000548S08430
Lycop_Sekr_SK06G11860
Glauc_Cyapar_g18201.t1
0.6

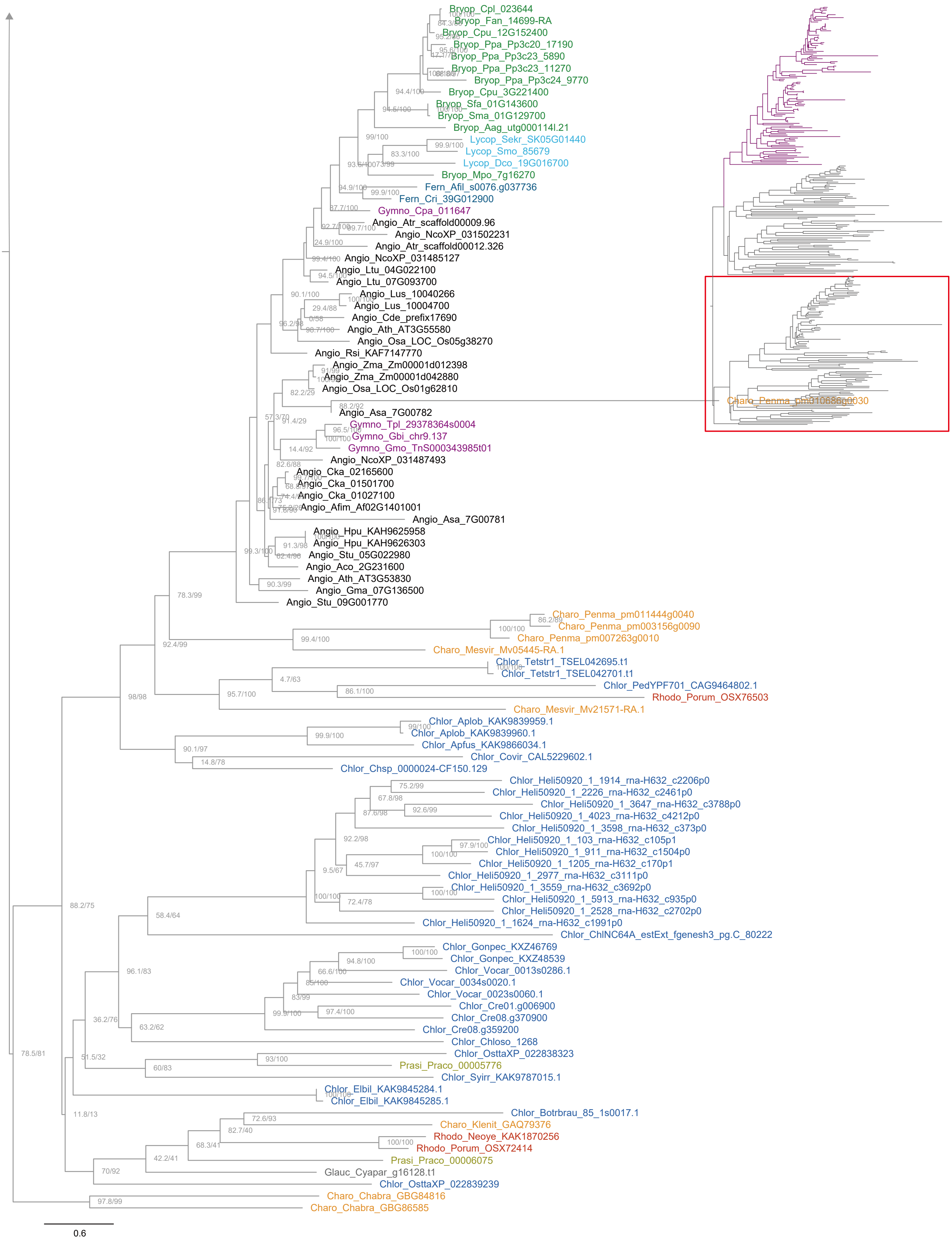

Supplementary Figure 1. Structure-guided phylogenetic analysis of UVR8. The phylogenetic tree was constructed using IQ-TREE v2.1.4 based on the best-fitting model (WAG+F+R8) with 1,000 bootstrap replicates. Purple branches represent the UVR8 clade, and the colors of tip labels represent different plant lineages.

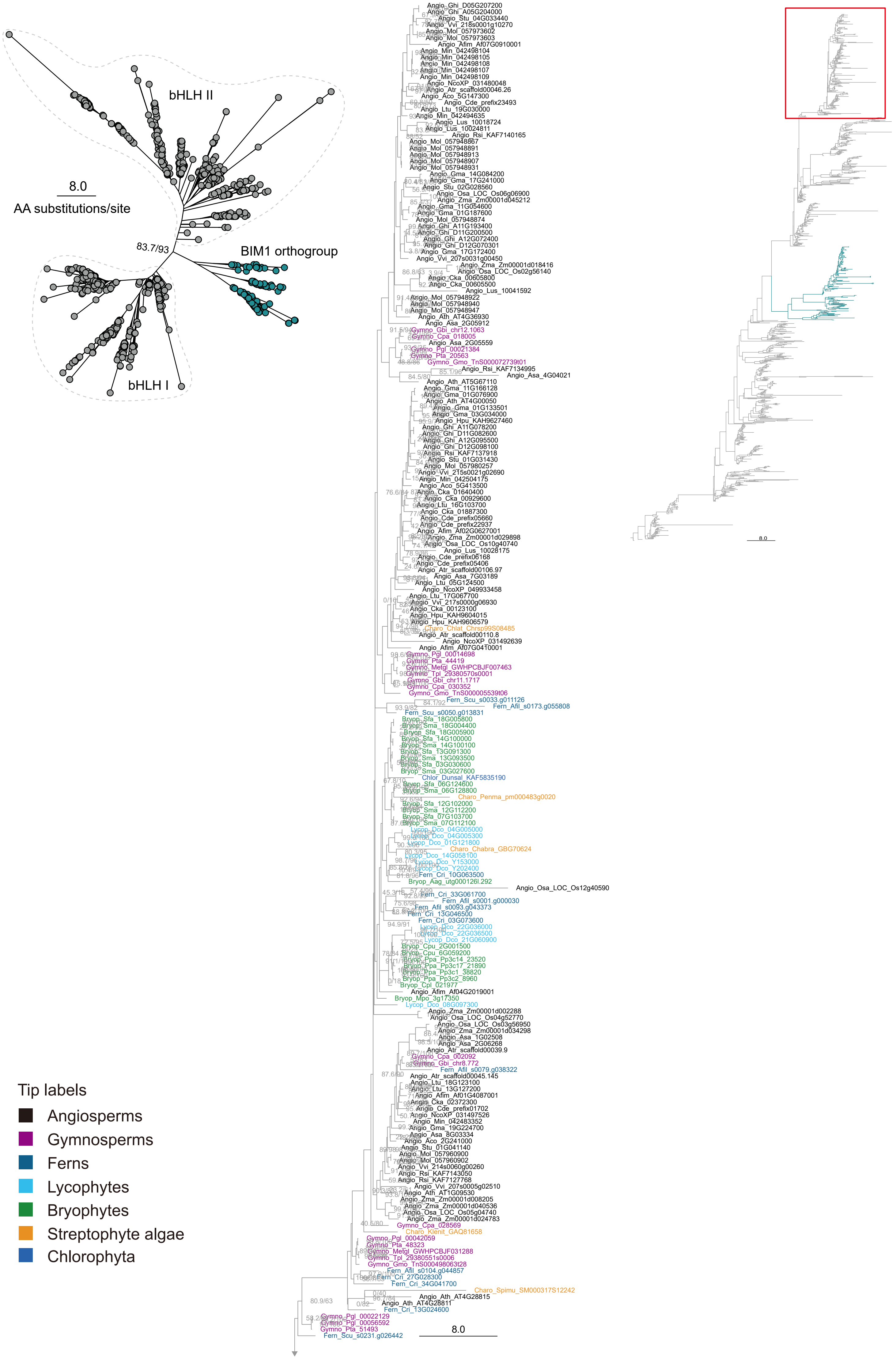

bHLH II
8.0
AA substitutions/site
83.7/93
BIM1 orthogroup
bHLH I
8.0
Tip labels
Angiosperms
Gymnosperms
Ferns
Lycophytes
Bryophytes
Streptophyte algae
Chlorophyta

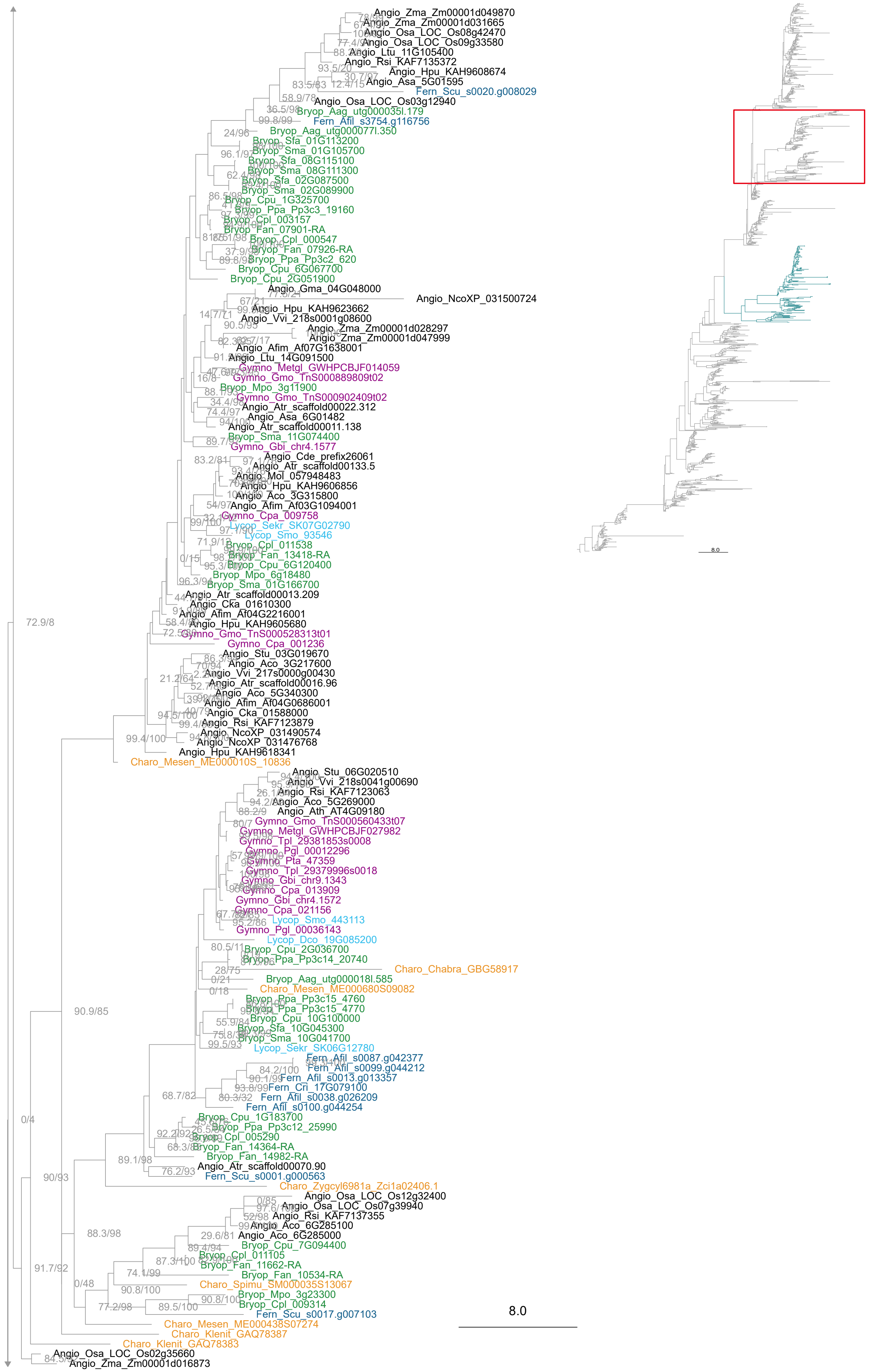
Angio_Zma_Zm00001d049870
Angio_Zma_Zm00001d031665
Angio_Osa_LOC_Os08g42470
Angio_Osa_LOC_Os09g33580
Angio_Ltu_11G105400
Angio_Rsi_KAF7135372
Angio_Hpu_KAH9608674
Angio_Asa_5G01595
Fern_Scu_s0020.g008029
Angio_Osa_LOC_Os03g12940
Bryop_Aag_utg000035l.179
Fern_Afil_s3754.g116756
Bryop_Aag_utg000077l.350
Bryop_Sfa_01G113200
Bryop_Sma_01G105700
Bryop_Sfa_08G115100
Bryop_Sma_08G111300
Bryop_Sfa_02G087500
Bryop_Sma_02G089900
Bryop_Cpu_1G325700
Bryop_Ppa_Pp3c3_19160
Bryop_Cpl_003157
Bryop_Fan_07901-RA
Bryop_Cpl_000547
Bryop_Fan_07926-RA
Bryop_Ppa_Pp3c2_620
Bryop_Cpu_6G067700
Bryop_Cpu_2G051900
Angio_Gma_04G048000
Angio_NcoXP_031500724
Angio_Hpu_KAH9623662
Angio_Vvi_218s0001g08600
Angio_Zma_Zm00001d028297
Angio_Zma_Zm00001d047999
Angio_Afim_Af07G1638001
Angio_Ltu_14G091500
Gymno_Metgl_GWHPCBJF014059
Gymno_Gmo_TnS000889809t02
Bryop_Mpo_3g11900
Gymno_Gmo_TnS000902409t02
Angio_Atr_scaffold00022.312
Angio_Asa_6G01482
Angio_Atr_scaffold00011.138
Bryop_Sma_11G074400
Gymno_Gbi_chr4.1577
Angio_Cde_prefix26061
Angio_Atr_scaffold00133.5
Angio_Mol_057948483
Angio_Hpu_KAH9606856
Angio_Aco_3G315800
Angio_Afim_Af03G1094001
Gymno_Cpa_009758
Lycop_Sekr_SK07G02790
Lycop_Smo_93546
Bryop_Cpl_011538
Bryop_Fan_13418-RA
Bryop_Cpu_6G120400
Bryop_Mpo_6g18480
Bryop_Sma_01G166700
Angio_Atr_scaffold00013.209
Angio_Cka_01610300
Angio_Afim_Af04G2216001
Angio_Hpu_KAH9605680
Gymno_Gmo_TnS000528313t01
Gymno_Cpa_001236
Angio_Stu_03G019670
Angio_Aco_3G217600
Angio_Vvi_217s0000g00430
Angio_Atr_scaffold00016.96
Angio_Aco_5G340300
Angio_Afim_Af04G0686001
Angio_Cka_01588000
Angio_Rsi_KAF7123879
Angio_NcoXP_031490574
Angio_NcoXP_031476768
Angio_Hpu_KAH9618341
Charo_Mesen_ME000010S_10836
Angio_Stu_06G020510
Angio_Vvi_218s0041g00690
Angio_Rsi_KAF7123063
Angio_Aco_5G269000
Angio_Ath_AT4G09180
Gymno_Gmo_TnS000560433t07
Gymno_Metgl_GWHPCBJF027982
Gymno_Tpl_29381853s0008
Gymno_Pgl_00012296
Gymno_Pta_47359
Gymno_Tpl_29379996s0018
Gymno_Gbi_chr9.1343
Gymno_Cpa_013909
Gymno_Gbi_chr4.1572
Gymno_Cpa_021156
Lycop_Smo_443113
Gymno_Pgl_00036143
Lycop_Dco_19G085200
Bryop_Cpu_2G036700
Bryop_Ppa_Pp3c14_20740
Charo_Chabra_GBG58917
Bryop_Aag_utg000018l.585
Charo_Mesen_ME000680S09082
Bryop_Ppa_Pp3c15_4760
Bryop_Ppa_Pp3c15_4770
Bryop_Cpu_10G100000
Bryop_Sfa_10G045300
Bryop_Sma_10G041700
Lycop_Sekr_SK06G12780
Fern_Afil_s0087.g042377
Fern_Afil_s0099.g044212
Fern_Afil_s0013.g013357
Fern_Cri_17G079100
Fern_Afil_s0038.g026209
Fern_Afil_s0100.g044254
Bryop_Cpu_1G183700
Bryop_Ppa_Pp3c12_25990
Bryop_Cpl_005290
Bryop_Fan_14364-RA
Bryop_Fan_14982-RA
Angio_Atr_scaffold00070.90
Fern_Scu_s0001.g000563
Charo_Zygcyl6981a_Zci1a02406.1
Angio_Osa_LOC_Os12g32400
Angio_Osa_LOC_Os07g39940
Angio_Rsi_KAF7137355
Angio_Aco_6G285100
Angio_Aco_6G285000
Bryop_Cpu_7G094400
Bryop_Cpl_011105
Bryop_Fan_11662-RA
Bryop_Fan_10534-RA
Charo_Spimu_SM000035S13067
Bryop_Mpo_3g23300
Bryop_Cpl_009314
Fern_Scu_s0017.g007103
Charo_Mesen_ME000438S07274
Charo_Klenit_GAQ78387
Charo_Klenit_GAQ78383
Angio_Osa_LOC_Os02g35660
Angio_Zma_Zm00001d016873
72.9/8
99.4/100
90.9/85
0/4
68.7/82
89.1/98
90/93
88.3/98
91.7/92
0/48
8.0

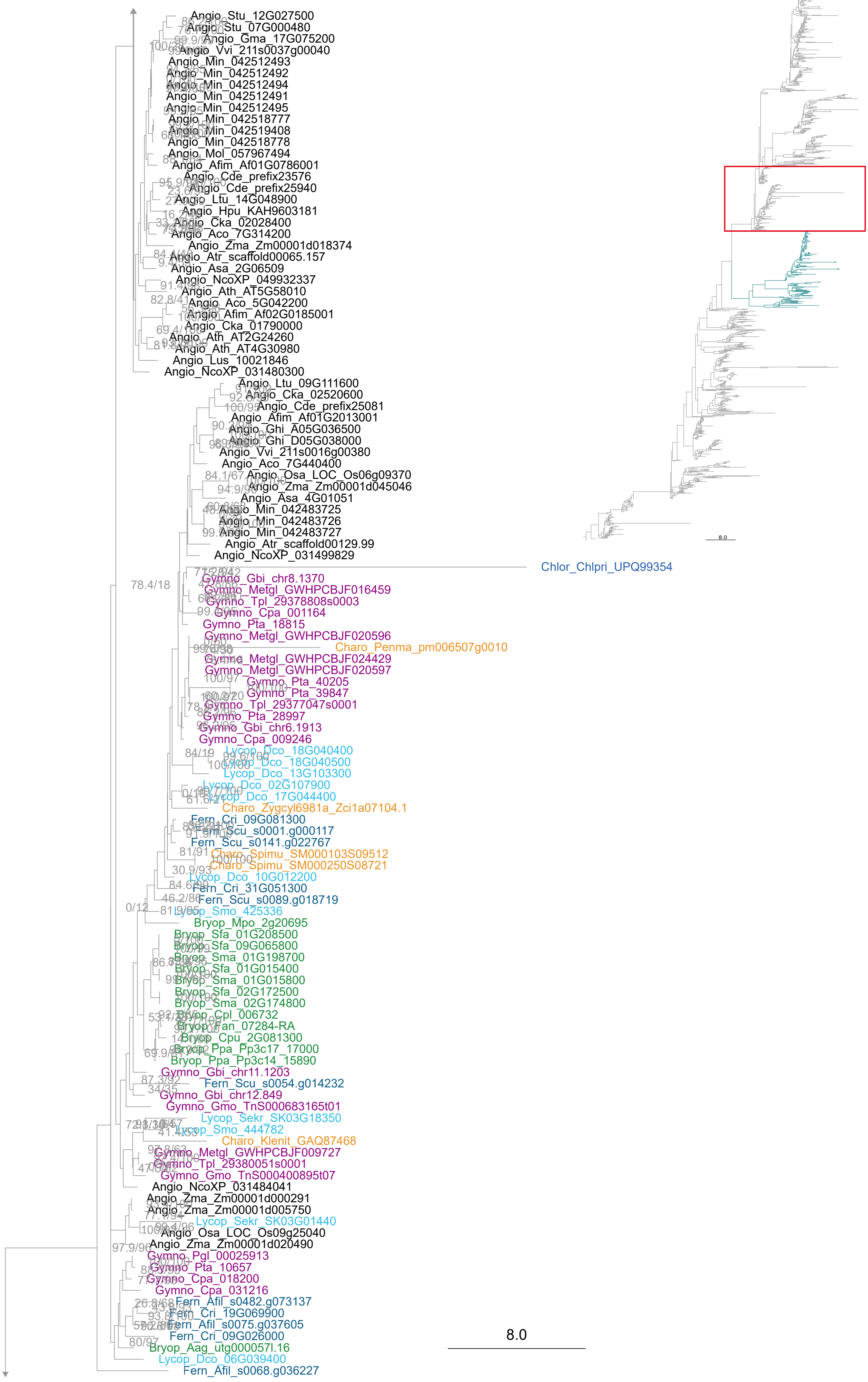
Angio_Stu_12G027500
Angio_Stu_07G000480
Angio_Gma_17G075200
Angio_Vvi_211s0037g00040
Angio_Min_042512493
Angio_Min_042512492
Angio_Min_042512494
Angio_Min_042512491
Angio_Min_042512495
Angio_Min_042518777
Angio_Min_042519408
Angio_Min_042518778
Angio_Mol_057967494
Angio_Afim_Af01G0786001
Angio_Cde_prefix23576
Angio_Cde_prefix25940
Angio_Ltu_14G048900
Angio_Hpu_KAH9603181
Angio_Cka_02028400
Angio_Aco_7G314200
Angio_Zma_Zm00001d018374
Angio_Atr_scaffold00065.157
Angio_Asa_2G06509
Angio_NcoXP_049932337
Angio_Ath_AT5G58010
Angio_Aco_5G042200
Angio_Afim_Af02G0185001
Angio_Cka_01790000
Angio_Ath_AT2G24260
Angio_Ath_AT4G30980
Angio_Lus_10021846
Angio_NcoXP_031480300
Angio_Ltu_09G111600
Angio_Cka_02520600
Angio_Cde_prefix25081
Angio_Afim_Af01G2013001
Angio_Ghi_A05G036500
Angio_Ghi_D05G038000
Angio_Vvi_211s0016g00380
Angio_Aco_7G440400
Angio_Osa_LOC_Os06g09370
Angio_Zma_Zm00001d045046
Angio_Asa_4G01051
Angio_Min_042483725
Angio_Min_042483726
Angio_Min_042483727
Angio_Atr_scaffold00129.99
Angio_NcoXP_031499829
Chlor_Chlpri_UPQ99354
78.4/18
Gymno_Gbi_chr8.1370
Gymno_Metgl_GWHPCBJF016459
Gymno_Tpl_29378808s0003
Gymno_Cpa_001164
Gymno_Pta_18815
Gymno_Metgl_GWHPCBJF020596
Charo_Penma_pm006507g0010
Gymno_Metgl_GWHPCBJF024429
Gymno_Metgl_GWHPCBJF020597
Gymno_Pta_40205
Gymno_Pta_39847
Gymno_Tpl_29377047s0001
Gymno_Pta_28997
Gymno_Gbi_chr6.1913
Gymno_Cpa_009246
Lycop_Dco_18G040400
Lycop_Dco_18G040500
Lycop_Dco_13G103300
Lycop_Dco_02G107900
Lycop_Dco_17G044400
Charo_Zygcyl6981a_Zci1a07104.1
Fern_Cri_09G081300
Fern_Scu_s0001.g000117
Fern_Scu_s0141.g022767
Charo_Spimu_SM000103S09512
Charo_Spimu_SM000250S08721
Lycop_Dco_10G012200
Fern_Cri_31G051300
Fern_Scu_s0089.g018719
0/12
Lycop_Smo_425336
Bryop_Mpo_2g20695
Bryop_Sfa_01G208500
Bryop_Sfa_09G065800
Bryop_Sma_01G198700
Bryop_Sfa_01G015400
Bryop_Sma_01G015800
Bryop_Sfa_02G172500
Bryop_Sma_02G174800
Bryop_Cpl_006732
Bryop_Fan_07284-RA
Bryop_Cpu_2G081300
Bryop_Ppa_Pp3c17_17000
Bryop_Ppa_Pp3c14_15890
Gymno_Gbi_chr11.1203
Fern_Scu_s0054.g014232
Gymno_Gbi_chr12.849
Gymno_Gmo_TnS000683165t01
Lycop_Sekr_SK03G18350
Lycop_Smo_444782
Charo_Klenit_GAQ87468
Gymno_Metgl_GWHPCBJF009727
Gymno_Tpl_29380051s0001
Gymno_Gmo_TnS000400895t07
Angio_NcoXP_031484041
Angio_Zma_Zm00001d000291
Angio_Zma_Zm00001d005750
Lycop_Sekr_SK03G01440
Angio_Osa_LOC_Os09g25040
Angio_Zma_Zm00001d020490
Gymno_Pgl_00025913
Gymno_Pta_10657
Gymno_Cpa_018200
Gymno_Cpa_031216
Fern_Afil_s0482.g073137
Fern_Cri_19G069900
Fern_Afil_s0075.g037605
Fern_Cri_09G026000
Bryop_Aag_utg000057l.16
Lycop_Dco_06G039400
Fern_Afil_s0068.g036227
8.0
8.0

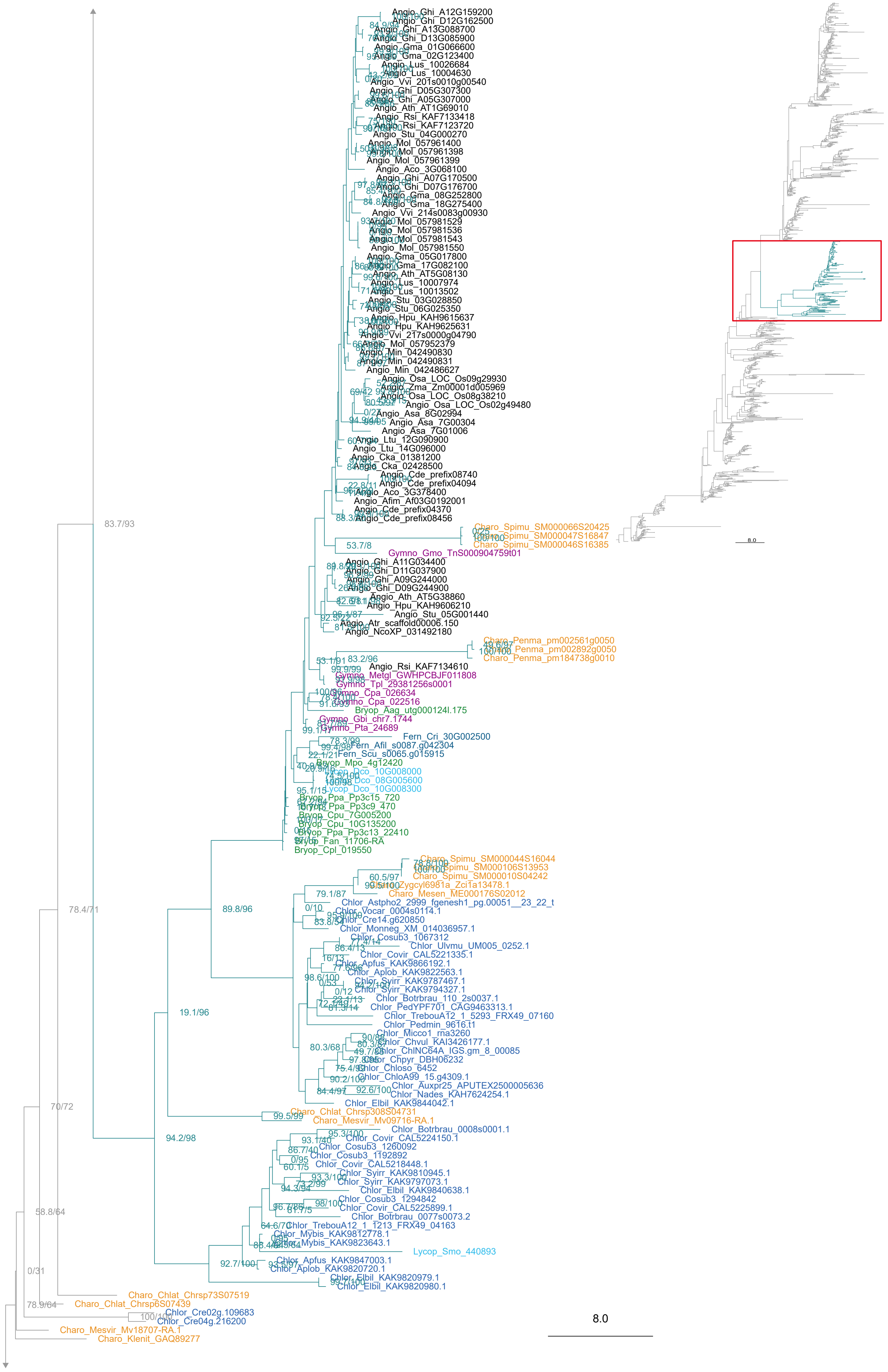

Angio_Ghi_A12G159200
Angio_Ghi_D12G162500
Angio_Ghi_A13G088700
Angio_Ghi_D13G085900
Angio_Gma_01G066600
Angio_Gma_02G123400
Angio_Lus_10026684
Angio_Lus_10004630
Angio_Vvi_201s0010g00540
Angio_Ghi_D05G307300
Angio_Ghi_A05G307000
Angio_Ath_AT1G69010
Angio_Rsi_KAF7133418
Angio_Rsi_KAF7123720
Angio_Stu_04G000270
Angio_Mol_057961400
Angio_Mol_057961398
Angio_Mol_057961399
Angio_Aco_3G068100
Angio_Ghi_A07G170500
Angio_Ghi_D07G176700
Angio_Gma_08G252800
Angio_Gma_18G275400
Angio_Vvi_214s0083g00930
Angio_Mol_057981529
Angio_Mol_057981536
Angio_Mol_057981543
Angio_Mol_057981550
Angio_Gma_05G017800
Angio_Gma_17G082100
Angio_Ath_AT5G08130
Angio_Lus_10007974
Angio_Lus_10013502
Angio_Stu_03G028850
Angio_Stu_06G025350
Angio_Hpu_KAH9615637
Angio_Hpu_KAH9625631
Angio_Vvi_217s0000g04790
Angio_Mol_057952379
Angio_Min_042490830
Angio_Min_042490831
Angio_Min_042486627
Angio_Osa_LOC_Os09g29930
Angio_Zma_Zm00001d005969
Angio_Osa_LOC_Os08g38210
Angio_Osa_LOC_Os02g49480
Angio_Asa_8G02994
Angio_Asa_7G00304
Angio_Asa_7G01006
Angio_Ltu_12G090900
Angio_Ltu_14G096000
Angio_Cka_01381200
Angio_Cka_02428500
Angio_Cde_prefix08740
Angio_Cde_prefix04094
Angio_Aco_3G378400
Angio_Afim_Af03G0192001
Angio_Cde_prefix04370
Angio_Cde_prefix08456
Charo_Spimu_SM000066S20425
Charo_Spimu_SM000047S16847
Charo_Spimu_SM000046S16385
53.7/8
Gymno_Gmo_TnS000904759t01
Angio_Ghi_A11G034400
Angio_Ghi_D11G037900
Angio_Ghi_A09G244000
Angio_Ghi_D09G244900
Angio_Ath_AT5G38860
Angio_Hpu_KAH9606210
Angio_Stu_05G001440
Angio_Atr_scaffold00006.150
Angio_NcoXP_031492180
Charo_Penma_pm002561g0050
Charo_Penma_pm002892g0050
Charo_Penma_pm184738g0010
83.2/96
Angio_Rsi_KAF7134610
Gymno_Metgl_GWHPCBJF011808
Gymno_Tpl_29381256s0001
Gymno_Cpa_026634
Gymno_Cpa_022516
Bryop_Aag_utg000124l.175
Gymno_Gbi_chr7.1744
Gymno_Pta_24689
Fern_Cri_30G002500
Fern_Afil_s0087.g042304
Fern_Scu_s0065.g015915
Bryop_Mpo_4g12420
Lycop_Dco_10G008000
Lycop_Dco_08G005600
Lycop_Dco_10G008300
Bryop_Ppa_Pp3c15_720
Bryop_Ppa_Pp3c9_470
Bryop_Cpu_7G005200
Bryop_Cpu_10G135200
Bryop_Ppa_Pp3c13_22410
Bryop_Fan_11706-RA
Bryop_Cpl_019550
83.7/93
Charo_Spimu_SM000044S16044
Charo_Spimu_SM000106S13953
Charo_Spimu_SM000010S04242
Charo_Zygcyl6981a_Zci1a13478.1
Charo_Mesen_ME000176S02012
Chlor_Astpho2_2999_fgenesh1_pg.00051__23_22_t
Chlor_Vocar_0004s0114.1
Chlor_Cre14.g620850
Chlor_Monneg_XM_014036957.1
Chlor_Cosub3_1067312
Chlor_Ulvmu_UM005_0252.1
Chlor_Covir_CAL5221335.1
Chlor_Apfus_KAK9866192.1
Chlor_Aplob_KAK9822563.1
Chlor_Syirr_KAK9787467.1
Chlor_Syirr_KAK9794327.1
Chlor_Botrbrau_110_2s0037.1
Chlor_PedYPF701_CAG9463313.1
Chlor_TrebouA12_1_5293_FRX49_07160
Chlor_Pedmin_9616.t1
Chlor_Micco1_rna3260
Chlor_Chvul_KAI3426177.1
Chlor_ChlNC64A_IGS.gm_8_00085
Chlor_Chpyr_DBH06232
Chlor_Chloso_6452
Chlor_ChloA99_15.g4309.1
Chlor_Auxpr25_APUTEX2500005636
Chlor_Nades_KAH7624254.1
Chlor_Elbil_KAK9844042.1
78.4/71
89.8/96
19.1/96
Charo_Chlat_Chrsp308S04731
Charo_Mesvir_Mv09716-RA.1
70/72
94.2/98
Chlor_Botrbrau_0008s0001.1
Chlor_Covir_CAL5224150.1
Chlor_Cosub3_1260092
Chlor_Cosub3_1192892
Chlor_Covir_CAL5218448.1
Chlor_Syirr_KAK9810945.1
Chlor_Syirr_KAK9797073.1
Chlor_Elbil_KAK9840638.1
Chlor_Cosub3_1294842
Chlor_Covir_CAL5225899.1
Chlor_Botrbrau_0077s0073.2
Chlor_TrebouA12_1_1213_FRX49_04163
Chlor_Mybis_KAK9812778.1
Chlor_Mybis_KAK9823643.1
Lycop_Smo_440893
Chlor_Apfus_KAK9847003.1
Chlor_Aplob_KAK9820720.1
Chlor_Elbil_KAK9820979.1
Chlor_Elbil_KAK9820980.1
58.8/64
0/31
Charo_Chlat_Chrsp73S07519
78.9/64
Charo_Chlat_Chrsp6S07439
Chlor_Cre02g.109683
Chlor_Cre04g.216200
Charo_Mesvir_Mv18707-RA.1
Charo_Klenit_GAQ89277
8.0

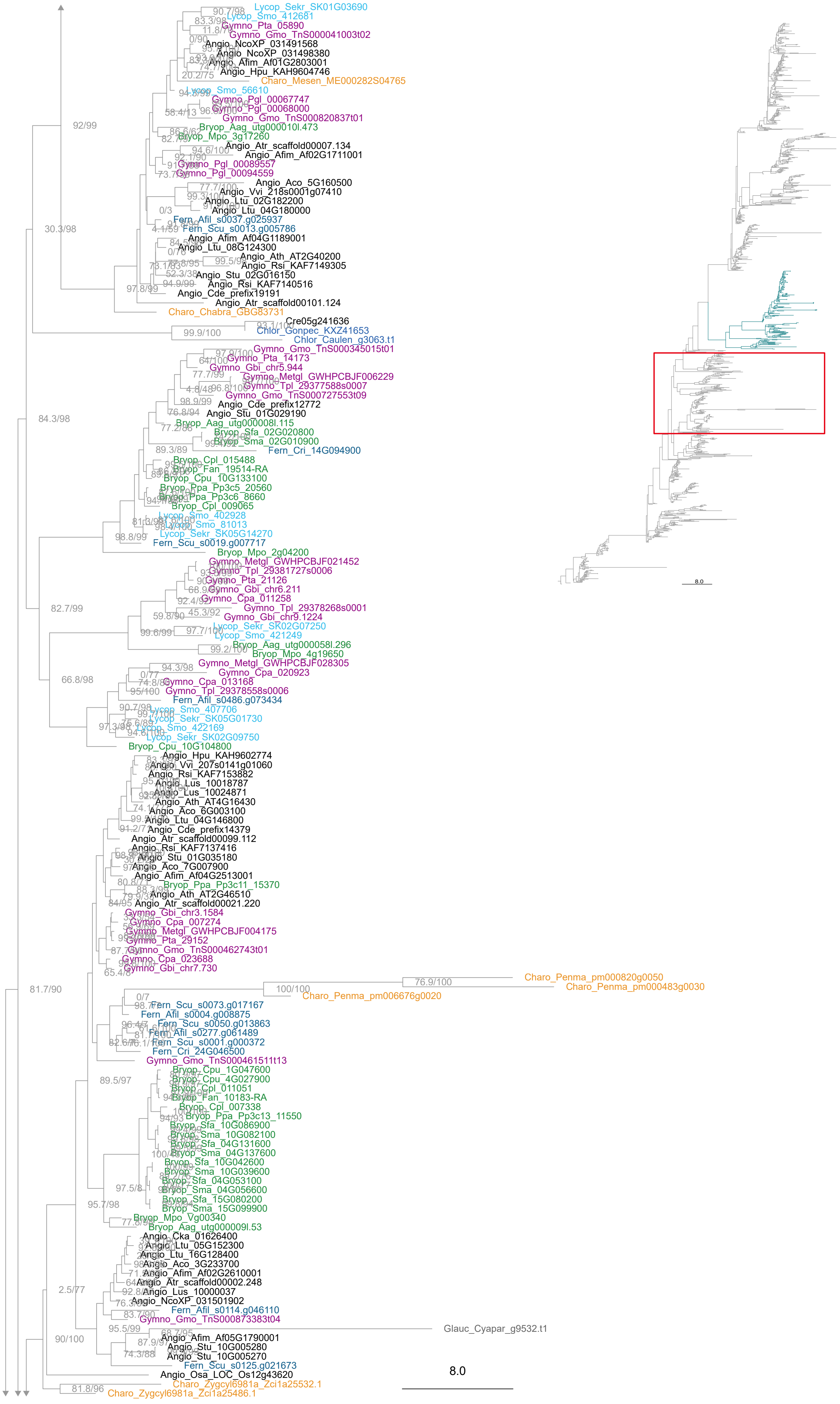

Lycop_Sekr_SK01G03690
Lycop_Smo_412681
Gymno_Pta_05890
Gymno_Gmo_TnS000041003t02
Angio_NcoXP_031491568
Angio_NcoXP_031498380
Angio_Afim_Af01G2803001
Angio_Hpu_KAH9604746
Charo_Mesen_ME000282S04765
Lycop_Smo_56610
Gymno_Pgl_00067747
Gymno_Pgl_00068000
Gymno_Gmo_TnS000820837t01
Bryop_Aag_utg000010l.473
Bryop_Mpo_3g17260
Angio_Atr_scaffold00007.134
Angio_Afim_Af02G1711001
Gymno_Pgl_00089557
Gymno_Pgl_00094559
Angio_Aco_5G160500
Angio_Vvi_218s0001g07410
Angio_Ltu_02G182200
Angio_Ltu_04G180000
Fern_Afil_s0037.g025937
Fern_Scu_s0013.g005786
Angio_Afim_Af04G1189001
Angio_Ltu_08G124300
Angio_Ath_AT2G40200
Angio_Rsi_KAF7149305
Angio_Stu_02G016150
Angio_Rsi_KAF7140516
Angio_Cde_prefix19191
Angio_Atr_scaffold00101.124
Charo_Chabra_GBG83731
Cre05g241636
Chlor_Gonpec_KXZ41653
Chlor_Caulen_g3063.t1
Gymno_Gmo_TnS000345015t01
Gymno_Pta_14173
Gymno_Gbi_chr5.944
Gymno_Metgl_GWHPCBJF006229
Gymno_Tpl_29377588s0007
Gymno_Gmo_TnS000727553t09
Angio_Cde_prefix12772
Angio_Stu_01G029190
Bryop_Aag_utg000008l.115
Bryop_Sfa_02G020800
Bryop_Sma_02G010900
Fern_Cri_14G094900
Bryop_Cpl_015488
Bryop_Fan_19514-RA
Bryop_Cpu_10G133100
Bryop_Ppa_Pp3c5_20560
Bryop_Ppa_Pp3c6_8660
Bryop_Cpl_009065
Lycop_Smo_402928
Lycop_Smo_81013
Lycop_Sekr_SK05G14270
Fern_Scu_s0019.g007717
Bryop_Mpo_2g04200
Gymno_Metgl_GWHPCBJF021452
Gymno_Tpl_29381727s0006
Gymno_Pta_21126
Gymno_Gbi_chr6.211
Gymno_Cpa_011258
Gymno_Tpl_29378268s0001
Gymno_Gbi_chr9.1224
Lycop_Sekr_SK02G07250
Lycop_Smo_421249
Bryop_Aag_utg000058l.296
Bryop_Mpo_4g19650
Gymno_Metgl_GWHPCBJF028305
Gymno_Cpa_020923
Gymno_Cpa_013168
Gymno_Tpl_29378558s0006
Fern_Afil_s0486.g073434
Lycop_Smo_407706
Lycop_Sekr_SK05G01730
Lycop_Smo_422169
Lycop_Sekr_SK02G09750
Bryop_Cpu_10G104800
Angio_Hpu_KAH9602774
Angio_Vvi_207s0141g01060
Angio_Rsi_KAF7153882
Angio_Lus_10018787
Angio_Lus_10024871
Angio_Ath_AT4G16430
Angio_Aco_6G003100
Angio_Ltu_04G146800
Angio_Cde_prefix14379
Angio_Atr_scaffold00099.112
Angio_Rsi_KAF7137416
Angio_Stu_01G035180
Angio_Aco_7G007900
Angio_Afim_Af04G2513001
Bryop_Ppa_Pp3c11_15370
Angio_Ath_AT2G46510
Angio_Atr_scaffold00021.220
Gymno_Gbi_chr3.1584
Gymno_Cpa_007274
Gymno_Metgl_GWHPCBJF004175
Gymno_Pta_29152
Gymno_Gmo_TnS000462743t01
Gymno_Cpa_023688
Gymno_Gbi_chr7.730
Charo_Penma_pm000820g0050
Charo_Penma_pm000483g0030
Charo_Penma_pm006676g0020
Fern_Scu_s0073.g017167
Fern_Afil_s0004.g008875
Fern_Scu_s0050.g013863
Fern_Afil_s0277.g061489
Fern_Scu_s0001.g000372
Fern_Cri_24G046500
Gymno_Gmo_TnS000461511t13
Bryop_Cpu_1G047600
Bryop_Cpu_4G027900
Bryop_Cpl_011051
Bryop_Fan_10183-RA
Bryop_Cpl_007338
Bryop_Ppa_Pp3c13_11550
Bryop_Sfa_10G086900
Bryop_Sma_10G082100
Bryop_Sfa_04G131600
Bryop_Sma_04G137600
Bryop_Sfa_10G042600
Bryop_Sma_10G039600
Bryop_Sfa_04G053100
Bryop_Sma_04G056600
Bryop_Sfa_15G080200
Bryop_Sma_15G099900
Bryop_Mpo_Vg00340
Bryop_Aag_utg000009l.53
Angio_Cka_01626400
Angio_Ltu_05G152300
Angio_Ltu_16G128400
Angio_Aco_3G233700
Angio_Afim_Af02G2610001
Angio_Atr_scaffold00002.248
Angio_Lus_10000037
Angio_NcoXP_031501902
Fern_Afil_s0114.g046110
Gymno_Gmo_TnS000873383t04
Glauc_Cyapar_g9532.t1
Angio_Afim_Af05G1790001
Angio_Stu_10G005280
Angio_Stu_10G005270
Fern_Scu_s0125.g021673
Angio_Osa_LOC_Os12g43620
Charo_Zygcyl6981a_Zci1a25532.1
Charo_Zygcyl6981a_Zci1a25486.1
92/99
30.3/98
84.3/98
82.7/99
66.8/98
81.7/90
89.5/97
95.7/98
2.5/77
90/100
81.8/96
8.0

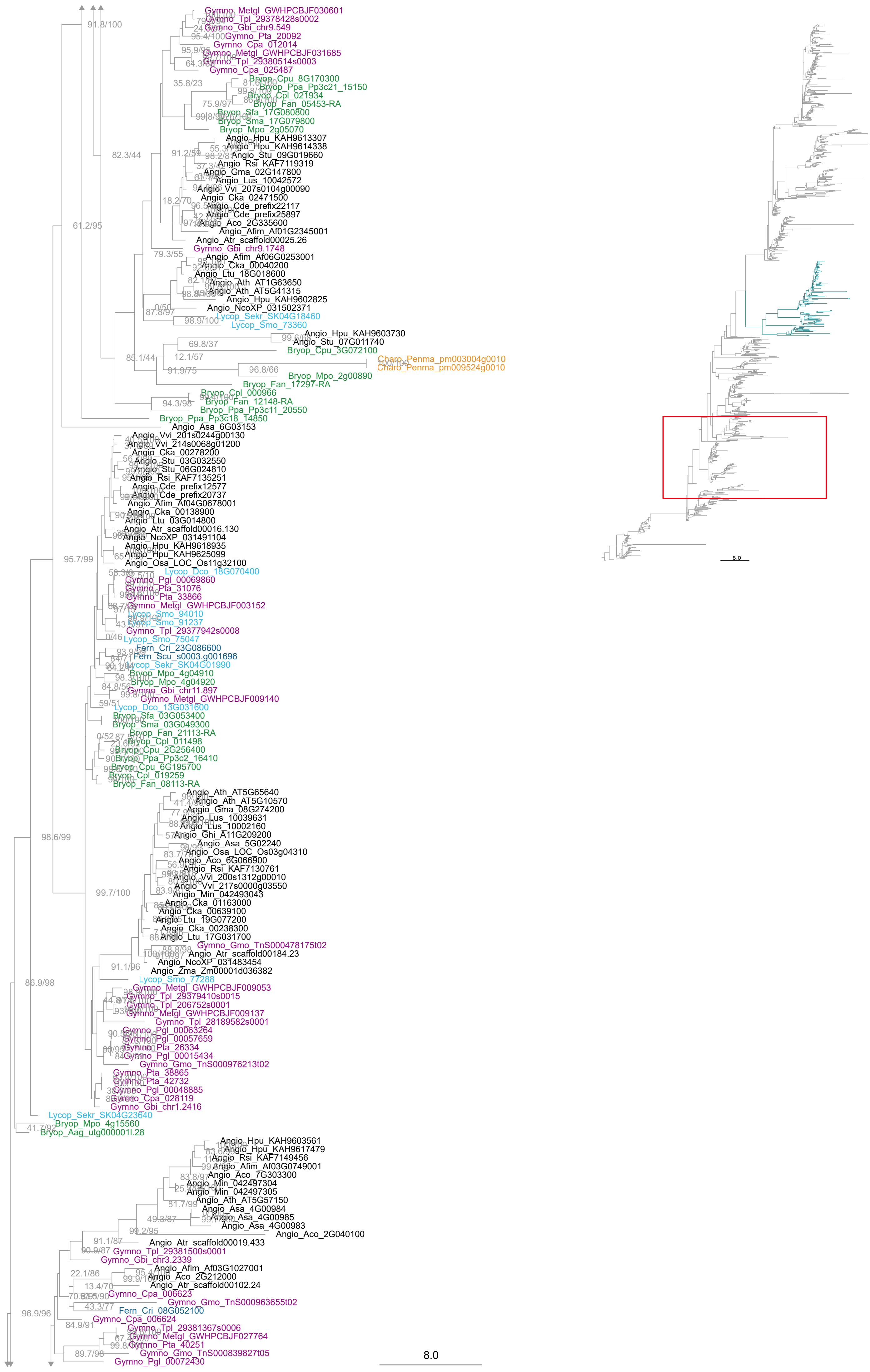

Gymno_Metgl_GWHPCBJF030601
Gymno_Tpl_29378428s0002
Gymno_Gbi_chr9.549
Gymno_Pta_20092
Gymno_Cpa_012014
Gymno_Metgl_GWHPCBJF031685
Gymno_Tpl_29380514s0003
Gymno_Cpa_025487
Bryop_Cpu_8G170300
Bryop_Ppa_Pp3c21_15150
Bryop_Cpl_021934
Bryop_Fan_05453-RA
Bryop_Sfa_17G080800
Bryop_Sma_17G079800
Bryop_Mpo_2g05070
Angio_Hpu_KAH9613307
Angio_Hpu_KAH9614338
Angio_Stu_09G019660
Angio_Rsi_KAF7119319
Angio_Gma_02G147800
Angio_Lus_10042572
Angio_Vvi_207s0104g00090
Angio_Cka_02471500
Angio_Cde_prefix22117
Angio_Cde_prefix25897
Angio_Aco_2G335600
Angio_Afim_Af01G2345001
Angio_Atr_scaffold00025.26
Gymno_Gbi_chr9.1748
Angio_Afim_Af06G0253001
Angio_Cka_00040200
Angio_Ltu_18G018600
Angio_Ath_AT1G63650
Angio_Ath_AT5G41315
Angio_Hpu_KAH9602825
Angio_NcoXP_031502371
Lycop_Sekr_SK04G18460
Lycop_Smo_73360
Angio_Hpu_KAH9603730
Angio_Stu_07G011740
Bryop_Cpu_3G072100
Charo_Penma_pm003004g0010
Charo_Penma_pm009524g0010
Bryop_Mpo_2g00890
Bryop_Fan_17297-RA
Bryop_Cpl_000966
Bryop_Fan_12148-RA
Bryop_Ppa_Pp3c11_20550
Bryop_Ppa_Pp3c18_14850
Angio_Asa_6G03153
Angio_Vvi_201s0244g00130
Angio_Vvi_214s0068g01200
Angio_Cka_00278200
Angio_Stu_03G032550
Angio_Stu_06G024810
Angio_Rsi_KAF7135251
Angio_Cde_prefix12577
Angio_Cde_prefix20737
Angio_Afim_Af04G0678001
Angio_Cka_00138900
Angio_Ltu_03G014800
Angio_Atr_scaffold00016.130
Angio_NcoXP_031491104
Angio_Hpu_KAH9618935
Angio_Hpu_KAH9625099
Angio_Osa_LOC_Os11g32100
Lycop_Dco_18G070400
Gymno_Pgl_00069860
Gymno_Pta_31076
Gymno_Pta_33866
Gymno_Metgl_GWHPCBJF003152
Lycop_Smo_94010
Lycop_Smo_91237
Gymno_Tpl_29377942s0008
Lycop_Smo_75047
Fern_Cri_23G086600
Fern_Scu_s0003.g001696
Lycop_Sekr_SK04G01990
Bryop_Mpo_4g04910
Bryop_Mpo_4g04920
Gymno_Gbi_chr11.897
Gymno_Metgl_GWHPCBJF009140
Lycop_Dco_13G031600
Bryop_Sfa_03G053400
Bryop_Sma_03G049300
Bryop_Fan_21113-RA
Bryop_Cpl_011498
Bryop_Cpu_2G256400
Bryop_Ppa_Pp3c2_16410
Bryop_Cpu_6G195700
Bryop_Cpl_019259
Bryop_Fan_08113-RA
Angio_Ath_AT5G65640
Angio_Ath_AT5G10570
Angio_Gma_08G274200
Angio_Lus_10039631
Angio_Lus_10002160
Angio_Ghi_A11G209200
Angio_Asa_5G02240
Angio_Osa_LOC_Os03g04310
Angio_Aco_6G066900
Angio_Rsi_KAF7130761
Angio_Vvi_200s1312g00010
Angio_Vvi_217s0000g03550
Angio_Min_042493043
Angio_Cka_01163000
Angio_Cka_00639100
Angio_Ltu_19G077200
Angio_Cka_00238300
Angio_Ltu_17G031700
Gymno_Gmo_TnS000478175t02
Angio_Atr_scaffold00184.23
Angio_NcoXP_031483454
Angio_Zma_Zm00001d036382
Lycop_Smo_77288
Gymno_Metgl_GWHPCBJF009053
Gymno_Tpl_29379410s0015
Gymno_Tpl_206752s0001
Gymno_Metgl_GWHPCBJF009137
Gymno_Tpl_28189582s0001
Gymno_Pgl_00063264
Gymno_Pgl_00057659
Gymno_Pta_26334
Gymno_Pgl_00015434
Gymno_Gmo_TnS000976213t02
Gymno_Pta_38865
Gymno_Pta_42732
Gymno_Pgl_00048885
Gymno_Cpa_028119
Gymno_Gbi_chr1.2416
Lycop_Sekr_SK04G23640
Bryop_Mpo_4g15560
Bryop_Aag_utg000001l.28
Angio_Hpu_KAH9603561
Angio_Hpu_KAH9617479
Angio_Rsi_KAF7149456
Angio_Afim_Af03G0749001
Angio_Aco_7G303300
Angio_Min_042497304
Angio_Min_042497305
Angio_Ath_AT5G57150
Angio_Asa_4G00984
Angio_Asa_4G00985
Angio_Asa_4G00983
Angio_Aco_2G040100
Angio_Atr_scaffold00019.433
Gymno_Tpl_29381500s0001
Gymno_Gbi_chr3.2339
Angio_Afim_Af03G1027001
Angio_Aco_2G212000
Angio_Atr_scaffold00102.24
Gymno_Cpa_006623
Gymno_Gmo_TnS000963655t02
Fern_Cri_08G052100
Gymno_Cpa_006624
Gymno_Tpl_29381367s0006
Gymno_Metgl_GWHPCBJF027764
Gymno_Pta_40251
Gymno_Gmo_TnS000839827t05
Gymno_Pgl_00072430
91.8/100
82.3/44
61.2/95
35.8/23
75.9/97
91.2/59
18.2/70
79.3/55
87.8/97
98.9/100
69.8/37
85.1/44
12.1/57
91.9/75
96.8/66
94.3/98
95.7/99
98.6/99
99.7/100
91.1/96
86.9/98
41.7/92
81.7/99
49.3/87
99.2/95
91.1/87
90.9/87
22.1/86
13.4/70
43.3/77
96.9/96
84.9/91
89.7/98
8.0

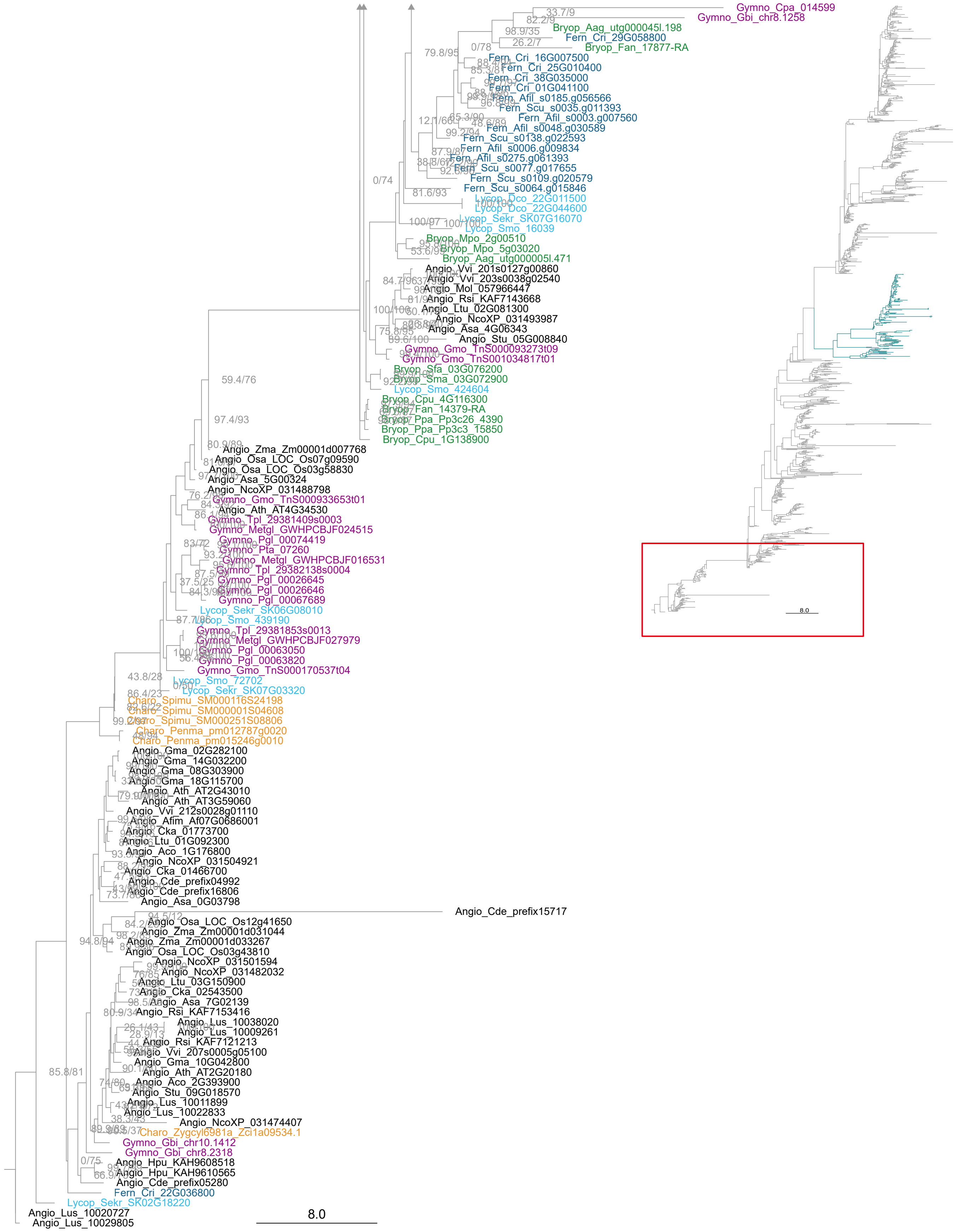

Supplementary Figure 2. Structure-guided phylogenetic analysis of BIM1. The phylogenetic tree was constructed using IQ-TREE v2.1.4 based on the best-fitting model (JTT+F+R10) with 1,000 bootstrap replicates. Green branches represent the BIM1 clade, and the colors of tip labels represent different plant lineages.

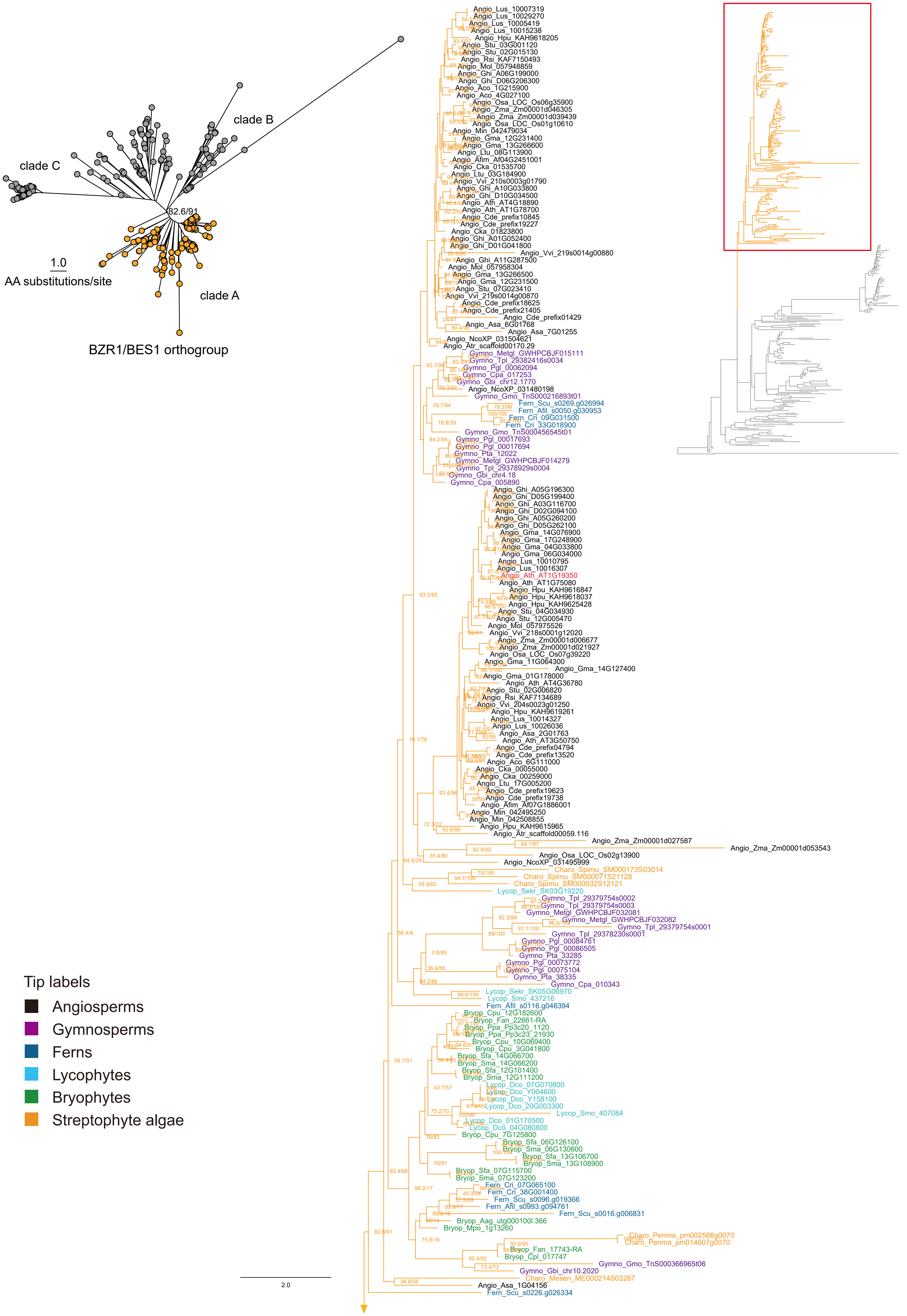

clade B
clade C
82.6/91
1.0
AA substitutions/site
clade A
BZR1/BES1 orthogroup
Angio_Lus_10007319
Angio_Lus_10029270
Angio_Lus_10005419
Angio_Lus_10015238
Angio_Hpu_KAH9618205
Angio_Stu_03G001120
Angio_Stu_02G015130
Angio_Rsi_KAF7150493
Angio_Mol_057948859
Angio_Ghi_A06G199000
Angio_Ghi_D06G206300
Angio_Aco_1G215900
Angio_Aco_4G027100
Angio_Osa_LOC_Os06g35900
Angio_Zma_Zm00001d046305
Angio_Zma_Zm00001d039439
Angio_Osa_LOC_Os01g10610
Angio_Min_042479034
Angio_Gma_12G231400
Angio_Gma_13G266600
Angio_Ltu_08G113900
Angio_Afim_Af04G2451001
Angio_Cka_01535700
Angio_Ltu_03G184900
Angio_Vvi_210s0003g01790
Angio_Ghi_A10G033800
Angio_Ghi_D10G034500
Angio_Ath_AT4G18890
Angio_Ath_AT1G78700
Angio_Cde_prefix10845
Angio_Cde_prefix19227
Angio_Cka_01823800
Angio_Ghi_A01G052400
Angio_Ghi_D01G041800
Angio_Vvi_219s0014g00880
Angio_Ghi_A11G287500
Angio_Mol_057958304
Angio_Gma_13G266500
Angio_Gma_12G231500
Angio_Stu_07G023410
Angio_Vvi_219s0014g00870
Angio_Cde_prefix18625
Angio_Cde_prefix21405
Angio_Cde_prefix01429
Angio_Asa_6G01768
Angio_Asa_7G01255
Angio_NcoXP_031504621
Angio_Atr_scaffold00170.29
Gymno_Metgl_GWHPCBJF015111
Gymno_Tpl_29382416s0034
Gymno_Pgl_00062094
Gymno_Cpa_017253
Gymno_Gbi_chr12.1770
Angio_NcoXP_031480198
Gymno_Gmo_TnS000216893t01
Fern_Scu_s0269.g026994
Fern_Afil_s0050.g030953
Fern_Cri_09G031500
Fern_Cri_33G018900
Gymno_Gmo_TnS000456545t01
Gymno_Pgl_00017693
Gymno_Pgl_00017694
Gymno_Pta_12022
Gymno_Metgl_GWHPCBJF014279
Gymno_Tpl_29378929s0004
Gymno_Gbi_chr4.18
Gymno_Cpa_005890
Angio_Ghi_A05G196300
Angio_Ghi_D05G199400
Angio_Ghi_A03G116700
Angio_Ghi_D02G094100
Angio_Ghi_A05G260200
Angio_Ghi_D05G262100
Angio_Gma_14G076900
Angio_Gma_17G248900
Angio_Gma_04G033800
Angio_Gma_06G034000
Angio_Lus_10010795
Angio_Lus_10016307
Angio_Ath_AT1G19350
Angio_Ath_AT1G75080
Angio_Hpu_KAH9616847
Angio_Hpu_KAH9618037
Angio_Hpu_KAH9625428
Angio_Stu_04G034930
Angio_Stu_12G005470
Angio_Mol_057975526
Angio_Vvi_218s0001g12020
Angio_Zma_Zm00001d006677
Angio_Zma_Zm00001d021927
Angio_Osa_LOC_Os07g39220
Angio_Gma_11G064300
Angio_Gma_14G127400
Angio_Gma_01G178000
Angio_Ath_AT4G36780
Angio_Stu_02G006820
Angio_Rsi_KAF7134689
Angio_Vvi_204s0023g01250
Angio_Hpu_KAH9619261
Angio_Lus_10014327
Angio_Lus_10026036
Angio_Asa_2G01763
Angio_Ath_AT3G50750
Angio_Cde_prefix04794
Angio_Cde_prefix13520
Angio_Aco_6G111000
Angio_Cka_00055000
Angio_Cka_00259000
Angio_Ltu_17G005200
Angio_Cde_prefix19623
Angio_Cde_prefix19738
Angio_Afim_Af07G1886001
Angio_Min_042495250
Angio_Min_042508855
Angio_Hpu_KAH9615965
Angio_Atr_scaffold00059.116
Angio_Zma_Zm00001d027587
Angio_Zma_Zm00001d053543
Angio_Osa_LOC_Os02g13900
Angio_NcoXP_031495999
Charo_Spimu_SM000173S03014
Charo_Spimu_SM000071S21128
Charo_Spimu_SM000032S12121
Lycop_Sekr_SK03G19220
Gymno_Tpl_29379754s0002
Gymno_Tpl_29379754s0003
Gymno_Metgl_GWHPCBJF032081
Gymno_Metgl_GWHPCBJF032082
Gymno_Tpl_29379754s0001
Gymno_Tpl_29378230s0001
Gymno_Pgl_00084761
Gymno_Pgl_00086505
Gymno_Pta_33285
Gymno_Pgl_00073772
Gymno_Pgl_00075104
Gymno_Pta_38335
Gymno_Cpa_010343
Lycop_Sekr_SK05G06970
Lycop_Smo_437216
Fern_Afil_s0116.g046394
Bryop_Cpu_12G182600
Bryop_Fan_22661-RA
Bryop_Ppa_Pp3c20_1120
Bryop_Ppa_Pp3c23_21930
Bryop_Cpu_10G069400
Bryop_Cpu_3G041800
Bryop_Sfa_14G066700
Bryop_Sma_14G066200
Bryop_Sfa_12G101400
Bryop_Sma_12G111200
Lycop_Dco_07G070800
Lycop_Dco_Y004600
Lycop_Dco_Y158100
Lycop_Dco_20G003300
Lycop_Smo_407084
Lycop_Dco_01G170500
Lycop_Dco_04G080800
Bryop_Cpu_7G125800
Bryop_Sfa_06G126100
Bryop_Sma_06G130600
Bryop_Sfa_13G106700
Bryop_Sma_13G108900
Bryop_Sfa_07G115700
Bryop_Sma_07G123200
Fern_Cri_07G065100
Fern_Cri_38G001400
Fern_Scu_s0096.g019366
Fern_Afil_s0993.g094761
Fern_Scu_s0016.g006831
Bryop_Aag_utg000100l.366
Bryop_Mpo_1g13260
Charo_Penma_pm002566g0070
Charo_Penma_pm014607g0070
Bryop_Fan_17743-RA
Bryop_Cpl_017747
Gymno_Gmo_TnS000366965t06
Gymno_Gbi_chr10.2020
Charo_Mesen_ME000214S03267
Angio_Asa_1G04156
Fern_Scu_s0226.g026334
2.0
Tip labels
Angiosperms
Gymnosperms
Ferns
Lycophytes
Bryophytes
Streptophyte algae

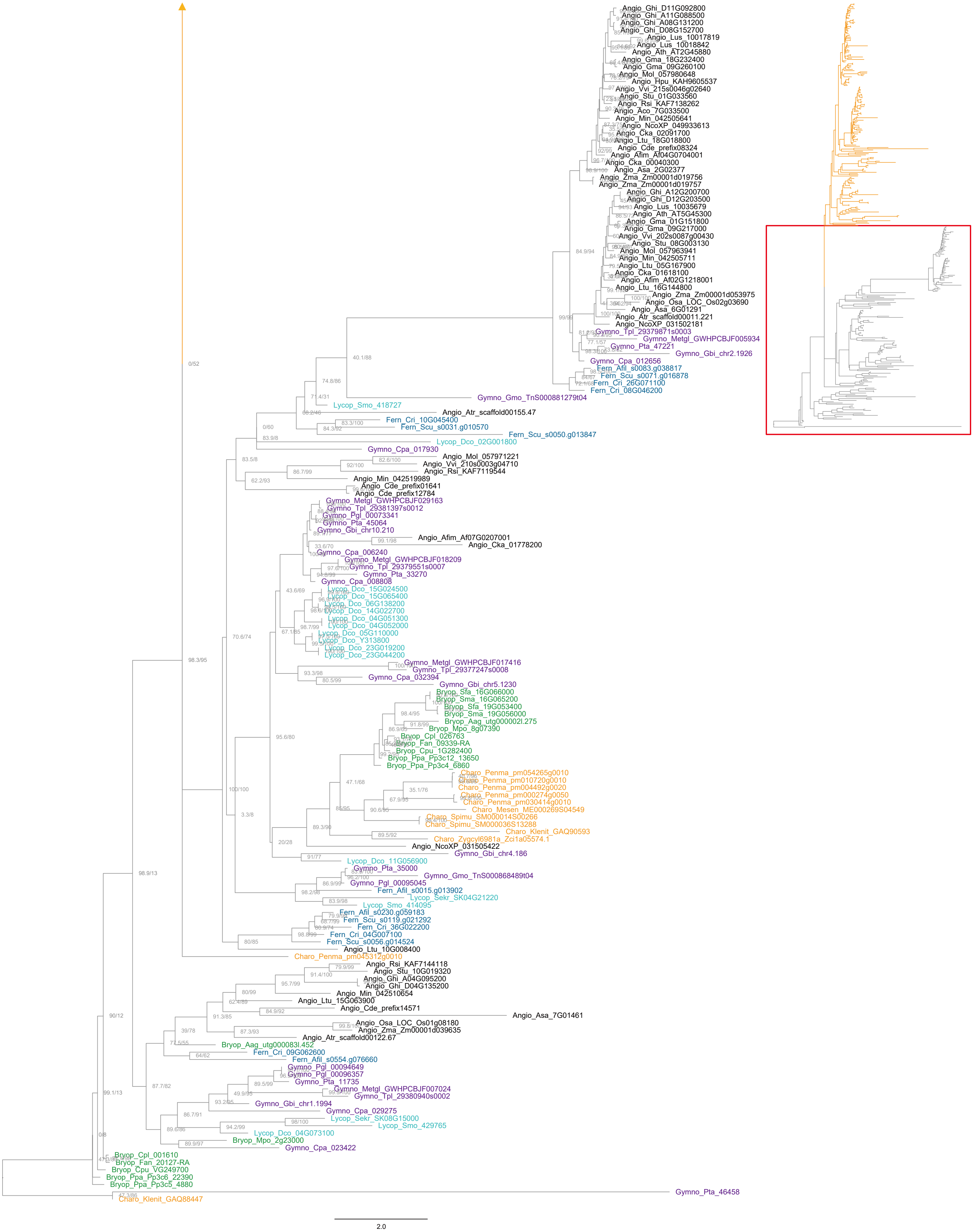

Supplementary Figure 3. Structure-guided phylogenetic analysis of BES1. The phylogenetic tree was constructed using IQ-TREE v2.1.4 based on the best-fitting model (JTT+F+R7) with 1,000 bootstrap replicates. Orange branches represent the BES1 clade, and the colors of tip labels represent different plant lineages.

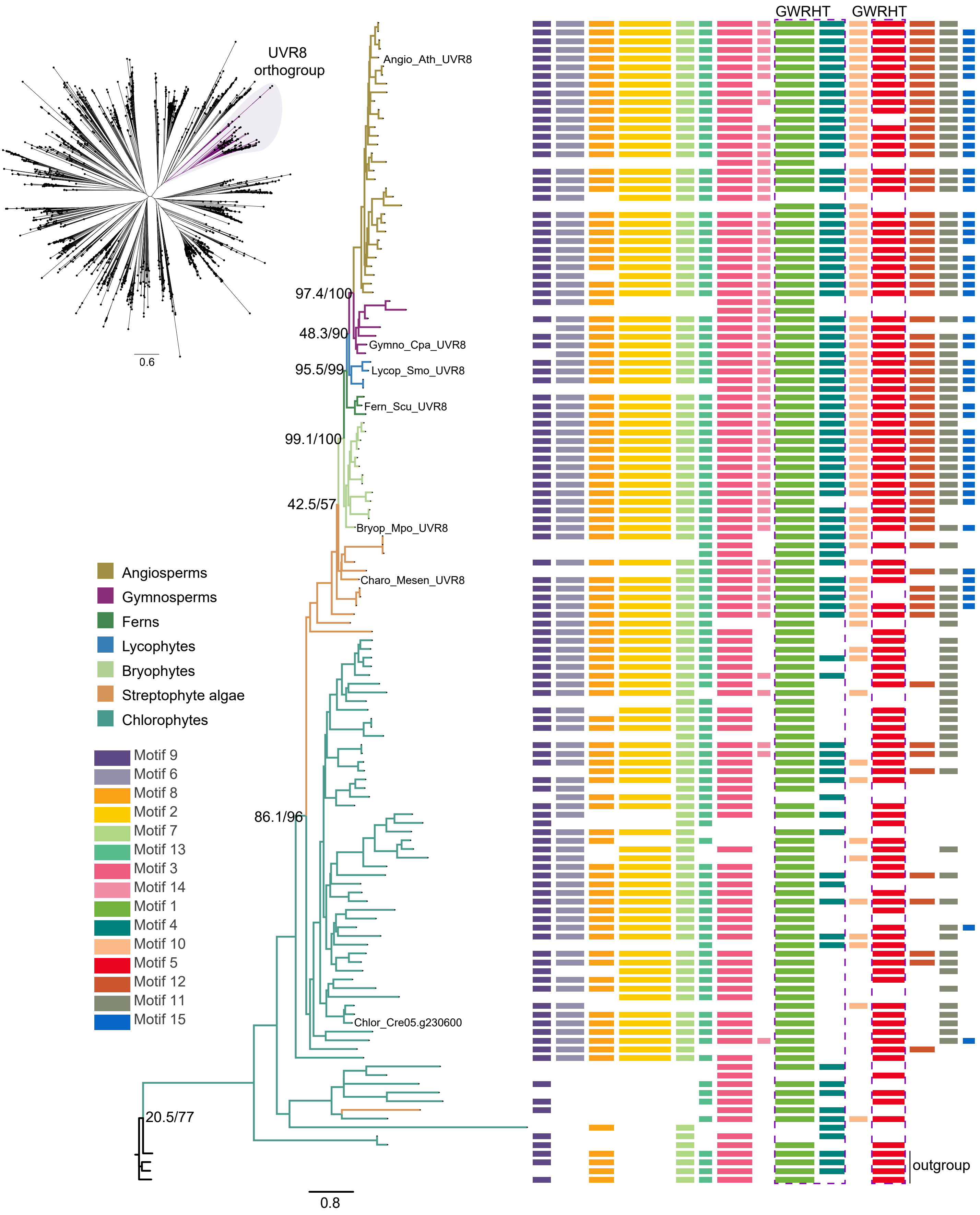

Supplementary Figure 4. Phylogenetic tree and domain architecture of *UVR8* orthologs. Maximum-likelihood phylogeny based on amino acid sequence alignments, with the LG+I+R7 model and 1,000 ultrafast bootstraps. The closest paralogs (black branches) were used to root the tree. Scale bar: amino acid substitutions per site. MEME-identified motifs are shown to the right as colored boxes; the dashed box marks the WGRHT-containing motif critical for UV-B perception. Motif sequences are listed in Supplementary Table 10.

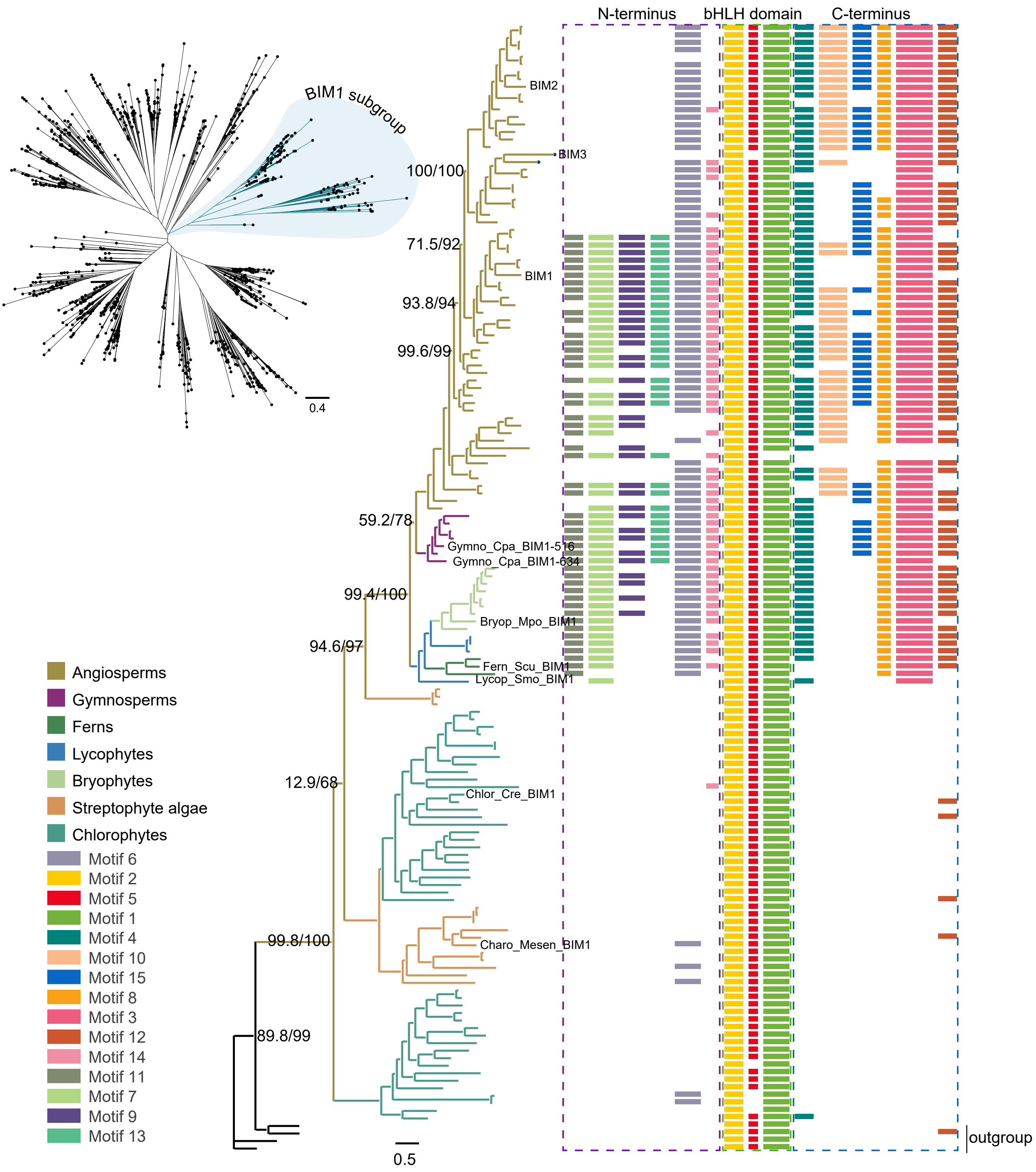

Supplementary Figure 5. Phylogenetic tree and domain architecture of *BIM1* orthologs. Maximum-likelihood phylogeny based on amino acid sequence alignments, with the JTT+F+R5 model and 1,000 ultrafast bootstraps. The closest paralogs (black branches) were used to root the tree. Scale bar: amino acid substitutions per site. MEME-identified motifs are shown to the right as colored boxes; the dashed box marks the N-terminal region, the bHLH domain, and the C-terminal region. Motif sequences are listed in Supplementary Table 10.

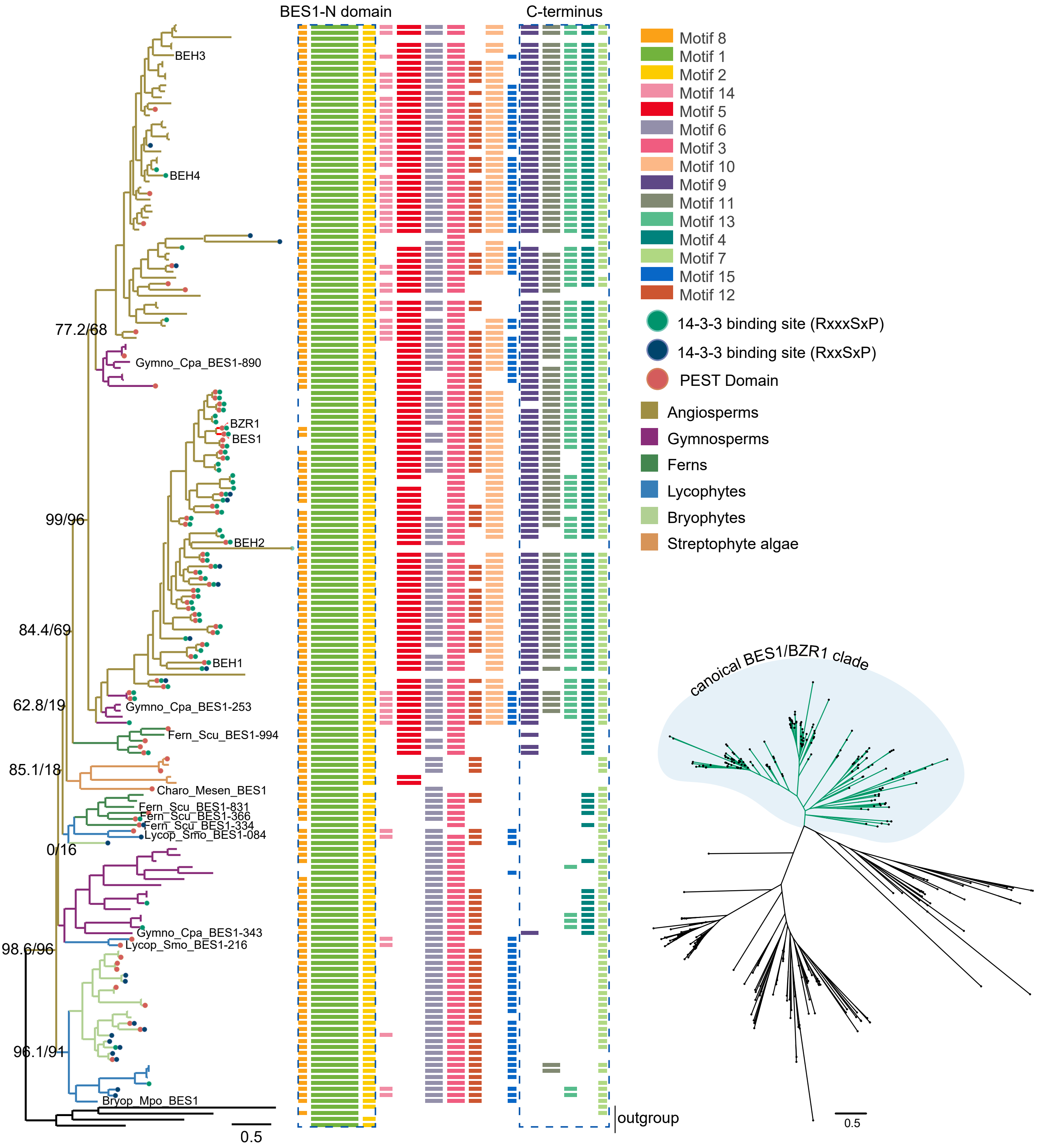

Supplementary Figure 6. Phylogenetic tree and domain architecture of *BES1* orthologs. Maximum-likelihood phylogeny based on amino acid sequence alignments, with the JTT+I+R7 model and 1,000 ultrafast bootstraps. The closest paralogs (black branches) were used to root the tree. Scale bar: amino acid substitutions per site. MEME-identified motifs are shown to the right as colored boxes; the dashed box marks the BES1 N-terminal domain and the C-terminal region. Motif sequences are listed in Supplementary Table 10.

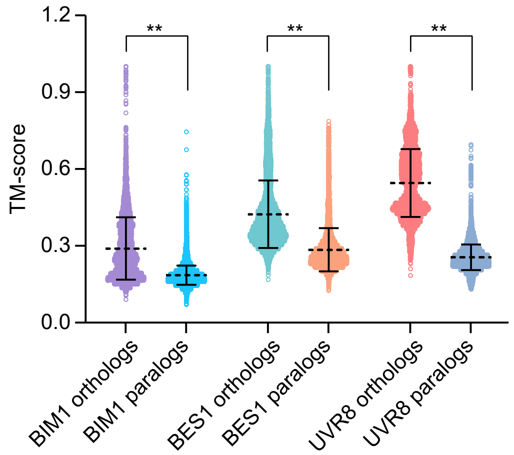

Supplementary Figure 7. Pairwise TM-scores between orthologs and paralogs. Pairwise protein structural alignments were performed using Foldseek (Van Kempen et al., 2024). p values were calculated using two-tailed Student's t-tests. $^{**}p < 0.01$.

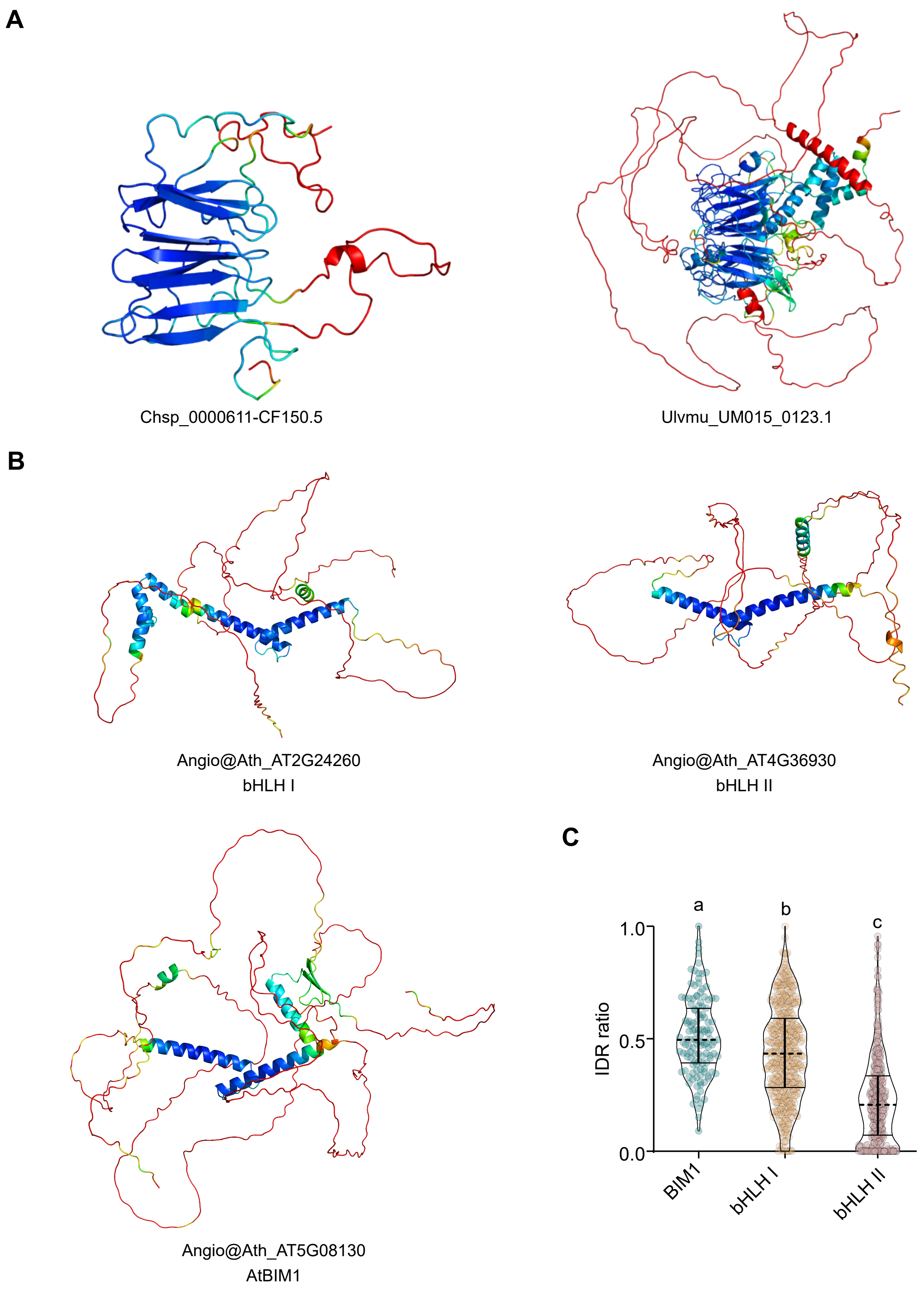

Supplementary Figure 8. Structural divergence between orthologs and paralogs of UVR8- and BIM1-like proteins. A, Example of structural divergence in UVR8 paralogs: Chsp_0000611-CF150.5 lacks full β-propeller architecture and Ulvmu_UM015_0123.1 carries an extra C-terminal domain beyond the canonical UVR8 core. **B**, Example of structural divergence in AtBIM1 and its paralogs. AtBIM1 harbors more intrinsically disordered regions than its paralogs. **C**, Ratio of intrinsically disordered region (IDR) length to total protein length for each sequence in the bHLH subfamily.

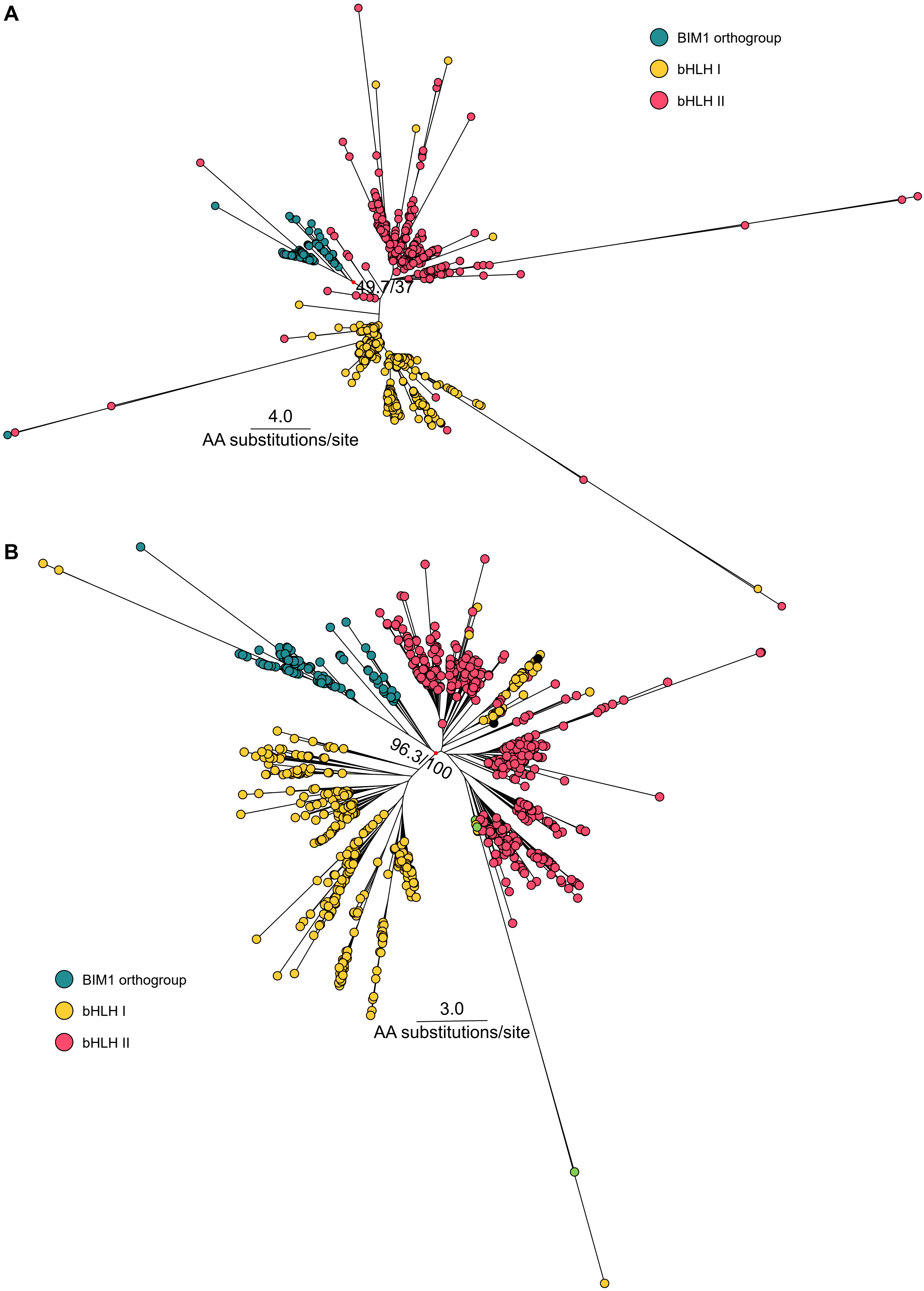

Supplementary Figure 9. Impact of IDRs on BIM1 phylogenetic reconstruction. A, Structure-guided unrooted tree of BIM1 constructed with a trimming threshold of 0.5 (-gt 0.5). **B,** Structure-guided unrooted tree of BIM1 constructed without trimming. Solid green circles at the tips denote the BIM1 clade, while red and yellow circles represent bHLH II and bHLH I, respectively.

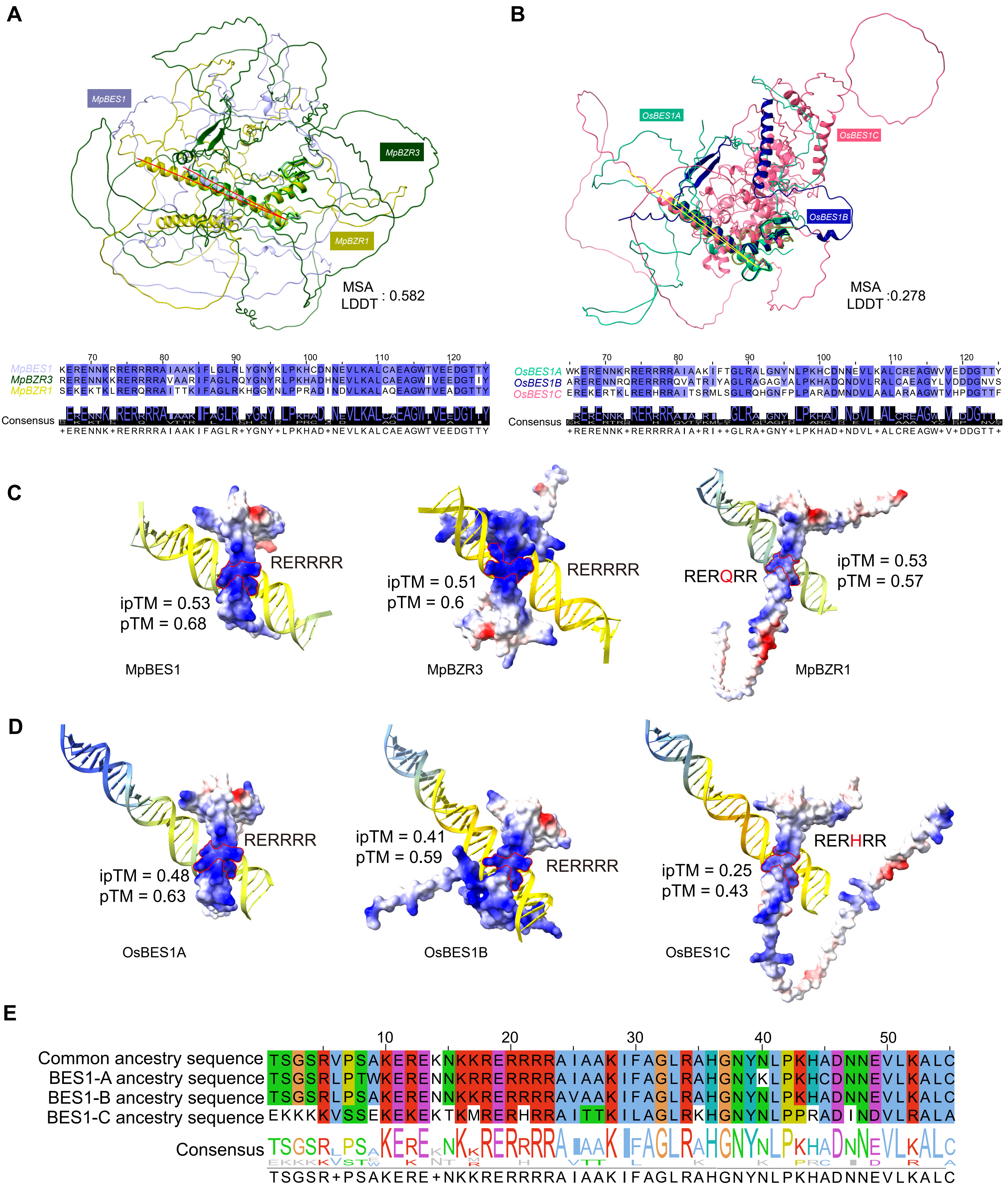

Supplementary Figure 10. Structural diversification of BES1 subfamilies in land plants. A,B, Structure alignment of BES1-A, BES1-B, and BES1-C subfamily members from *M. polymorpha* (A) and *O. sativa* (B). **C, D,** Electrostatic potential surfaces of molecular docking models showing the interaction between DNA and BES1 subfamily members from *M. polymorpha* (C) and *O. sativa* (D). The interface predicted template modeling (ipTM) and predicted template modeling (pTM) scores are indicated. In the electrostatic potential maps, deep blue indicates positively charged regions. **E**, Ancestral sequence reconstruction of the BES1-N domain.

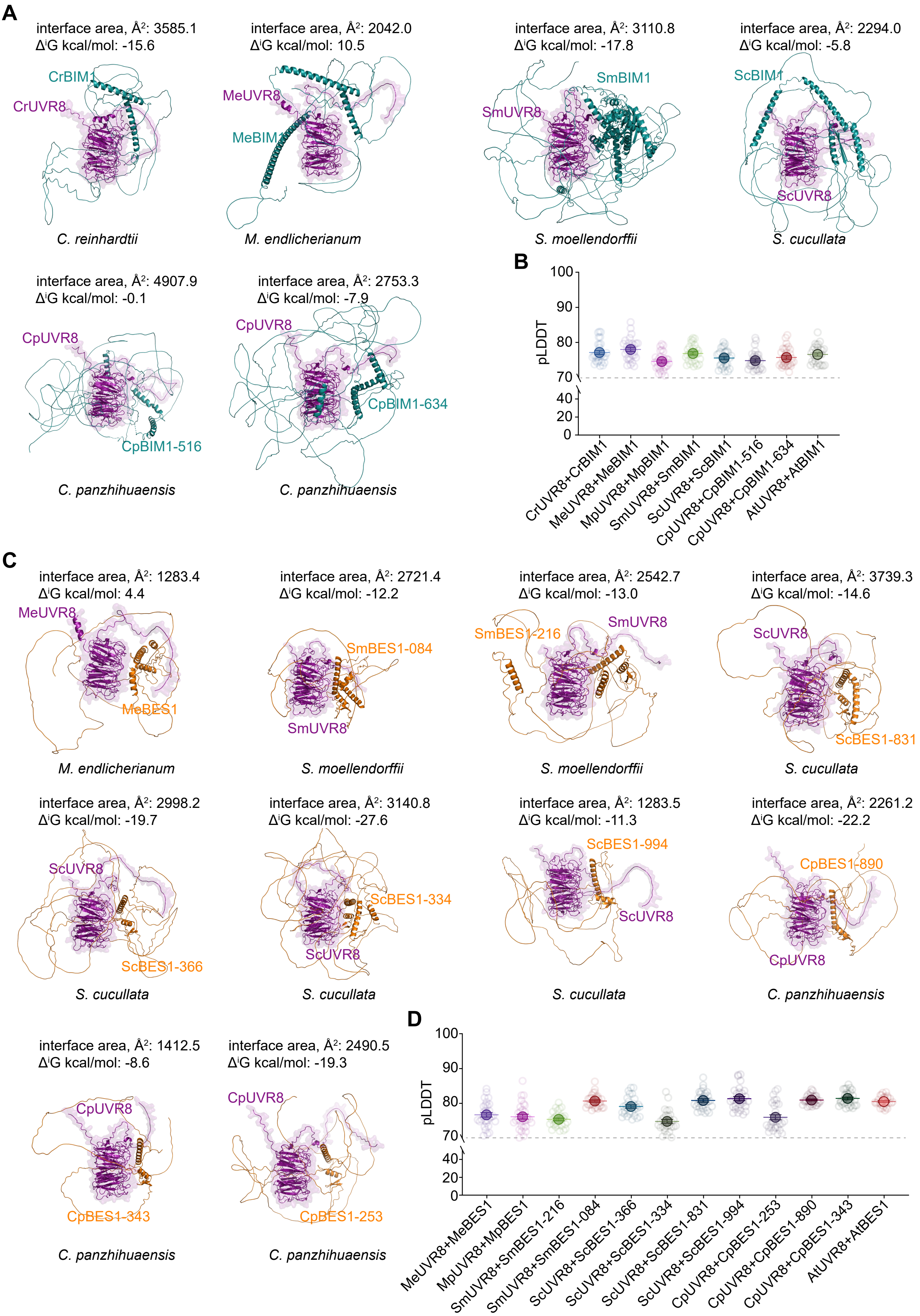
A
interface area, Å^2: 3585.1
Δ^{i}G kcal/mol: -15.6
CrBIM1
CrUVR8
C. reinhardtii
interface area, Å^2: 2042.0
Δ^{i}G kcal/mol: 10.5
MeUVR8
MeBIM1
M. endlicherianum
interface area, Å^2: 3110.8
Δ^{i}G kcal/mol: -17.8
SmBIM1
SmUVR8
S. moellendorffii
interface area, Å^2: 2294.0
Δ^{i}G kcal/mol: -5.8
ScBIM1
ScUVR8
S. cucullata
interface area, Å^2: 4907.9
Δ^{i}G kcal/mol: -0.1
CpUVR8
CpBIM1-516
C. panzhihuaensis
interface area, Å^2: 2753.3
Δ^{i}G kcal/mol: -7.9
CpUVR8
CpBIM1-634
C. panzhihuaensis
B
pLDDT
100
90
80
70
40
20
0
CrUVR8+CrBIM1
MeUVR8+MeBIM1
MpUVR8+MpBIM1
SmUVR8+SmBIM1
ScUVR8+ScBIM1
CpUVR8+CpBIM1-516
CpUVR8+CpBIM1-634
AtUVR8+AtBIM1
C
interface area, Å^2: 1283.4
Δ^{i}G kcal/mol: 4.4
MeUVR8
MeBES1
M. endlicherianum
interface area, Å^2: 2721.4
Δ^{i}G kcal/mol: -12.2
SmBES1-084
SmUVR8
S. moellendorffii
interface area, Å^2: 2542.7
Δ^{i}G kcal/mol: -13.0
SmBES1-216
SmUVR8
S. moellendorffii
interface area, Å^2: 3739.3
Δ^{i}G kcal/mol: -14.6
ScUVR8
ScBES1-831
S. cucullata
interface area, Å^2: 2998.2
Δ^{i}G kcal/mol: -19.7
ScUVR8
ScBES1-366
S. cucullata
interface area, Å^2: 3140.8
Δ^{i}G kcal/mol: -27.6
ScBES1-334
ScUVR8
S. cucullata
interface area, Å^2: 1283.5
Δ^{i}G kcal/mol: -11.3
ScBES1-994
ScUVR8
S. cucullata
interface area, Å^2: 2261.2
Δ^{i}G kcal/mol: -22.2
CpBES1-890
CpUVR8
C. panzhihuaensis
interface area, Å^2: 1412.5
Δ^{i}G kcal/mol: -8.6
CpUVR8
CpBES1-343
C. panzhihuaensis
interface area, Å^2: 2490.5
Δ^{i}G kcal/mol: -19.3
CpUVR8
CpBES1-253
C. panzhihuaensis
D
pLDDT
100
90
80
70
40
20
0
MeUVR8+MeBES1
MpUVR8+MpBES1
SmUVR8+SmBES1-216
SmUVR8+SmBES1-084
ScUVR8+ScBES1-366
ScUVR8+ScBES1-334
ScUVR8+ScBES1-831
ScUVR8+ScBES1-994
CpUVR8+CpBES1-253
CpUVR8+CpBES1-890
CpUVR8+CpBES1-343
AtUVR8+AtBES1

Supplementary Figure 11. Predicted dimeric structures of UVR8-BIM1 and UVR8-BES1. A,**C**, Examples of predicted structures for UVR8-BIM1 (A) and UVR8-BES1 (C) dimers in representative species. $\Delta^{i}G$ indicates the solvation free energy gain upon formation of the interface (in kcal/M). Lower $\Delta^{i}G$ and larger interface areas indicates higher likelihood of physical interaction. **B**,**D**, pLDDT scores of UVR8-BIM1 (B) and UVR8-BES1 (D) dimer structures in representative species. Each dot represents one prediction repeat. pLDDT $\geq$ 70 denotes confident structures.

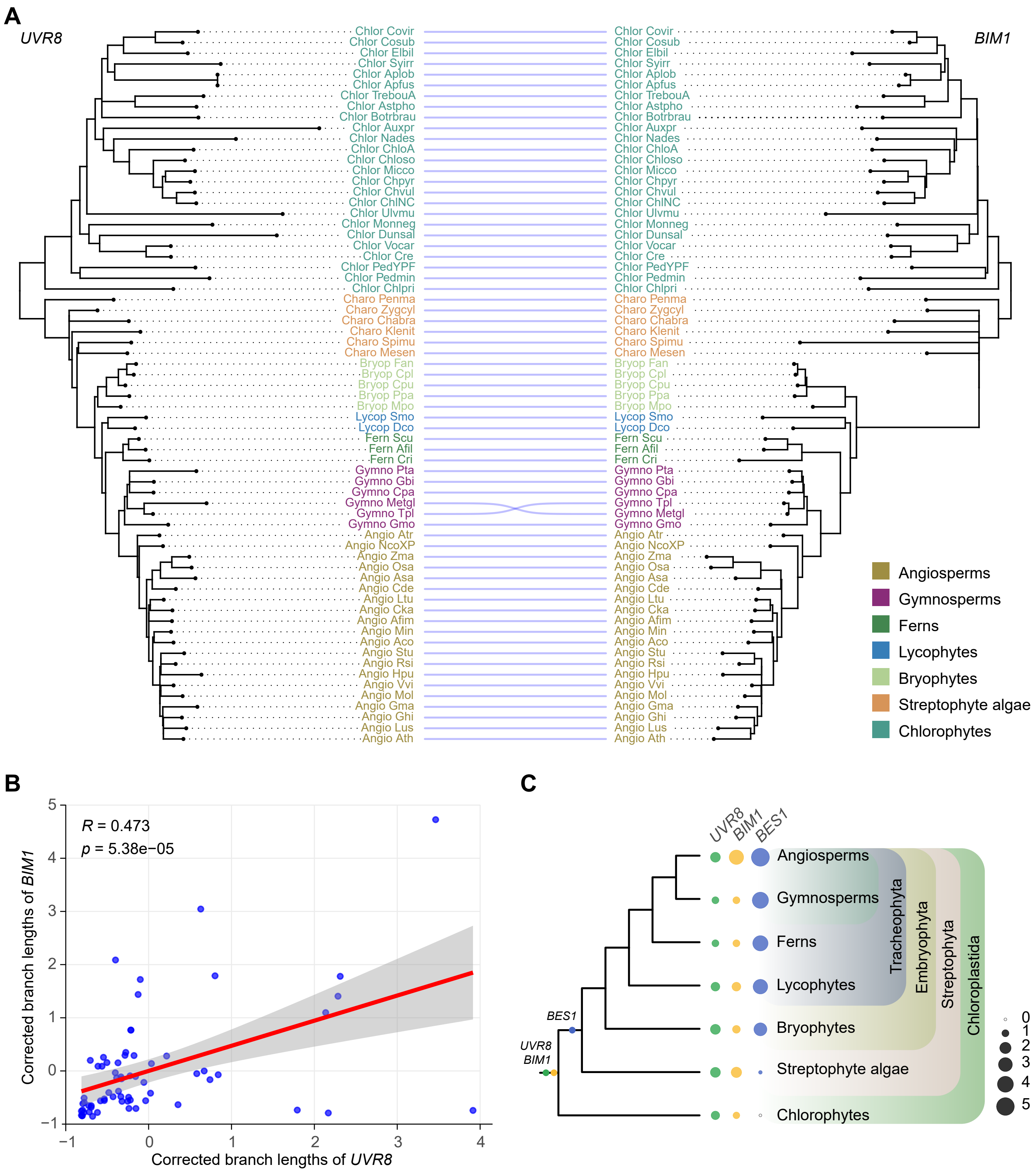

Supplementary Figure 12. Coordinated evolution of UVR8 and BIM1 in green plants. **A**, The mirror-tree of *UVR8* and *BIM1*. **B**, Evolutionary rate covariation (ERC) analysis between *UVR8* and *BIM1* was performed using the CovER function in PhyKIT. **C**, Copy number of *UVR8*, *BIM1*, and *BES1* orthologs in major green plant lineages.

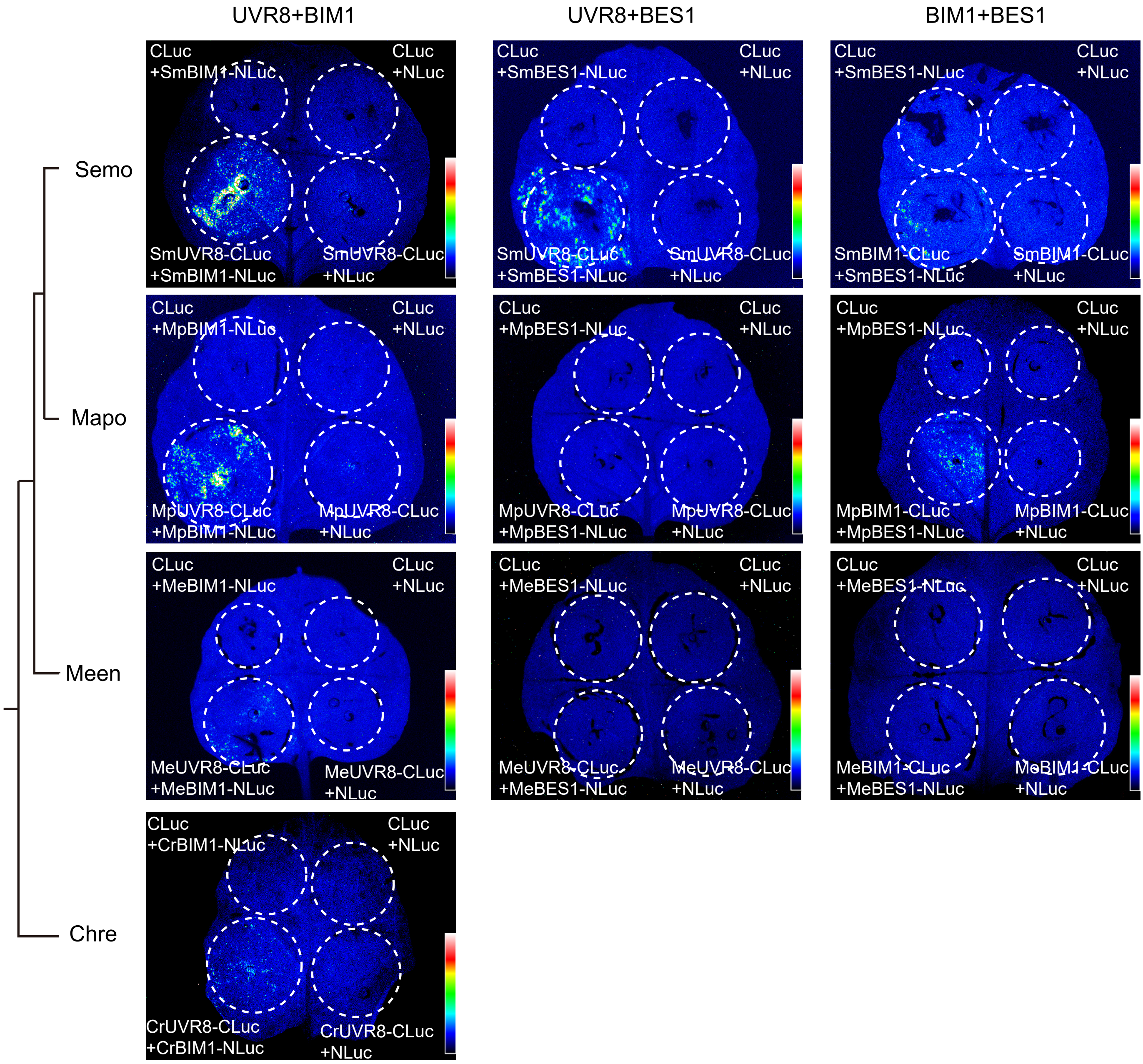

Supplementary Figure 13. Representative images of luciferase complementation in tobacco leaves. The pseudocolor scale indicates the luminescence intensity, with red and blue representing high and low signals, respectively. CLuc + NLuc-gene, CLuc-gene + NLuc, and CLuc + NLuc were used as negative controls.

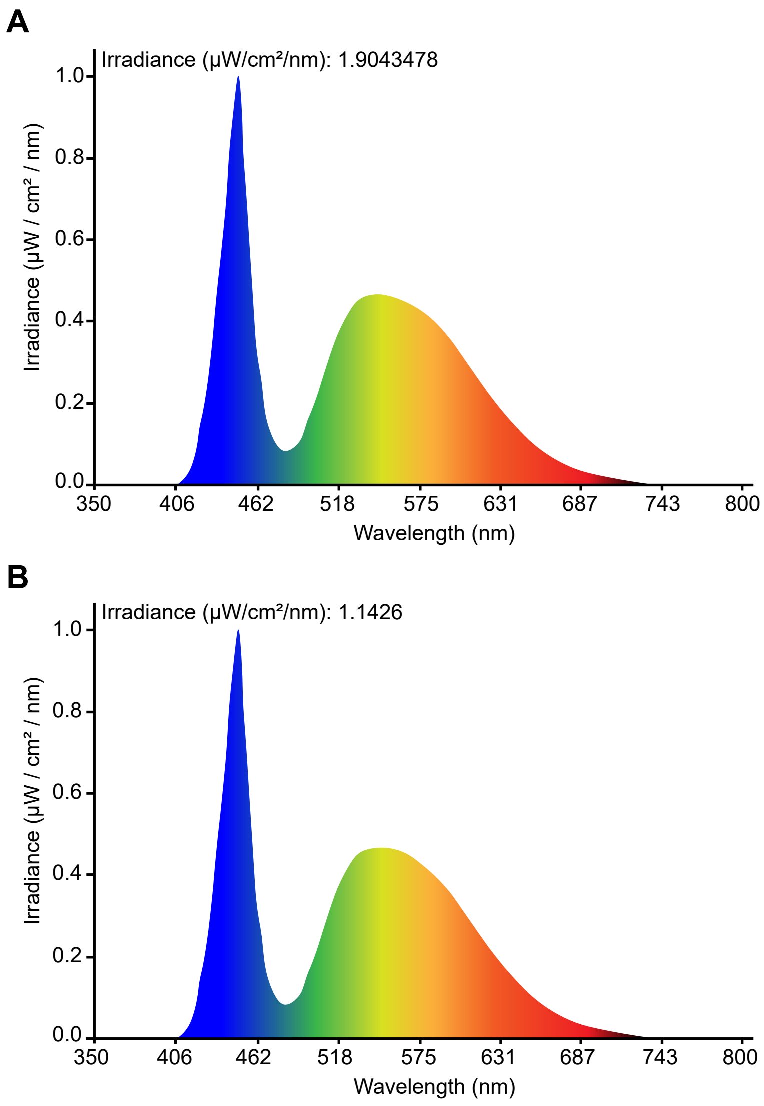

Supplementary Figure 14. Light spectra used for *M. endlicherianum* and *M. polymorpha* cultivation. A, B, Light spectra of the growth chambers used for *M. polymorpha* (A) and *M. endlicherianum* (B).

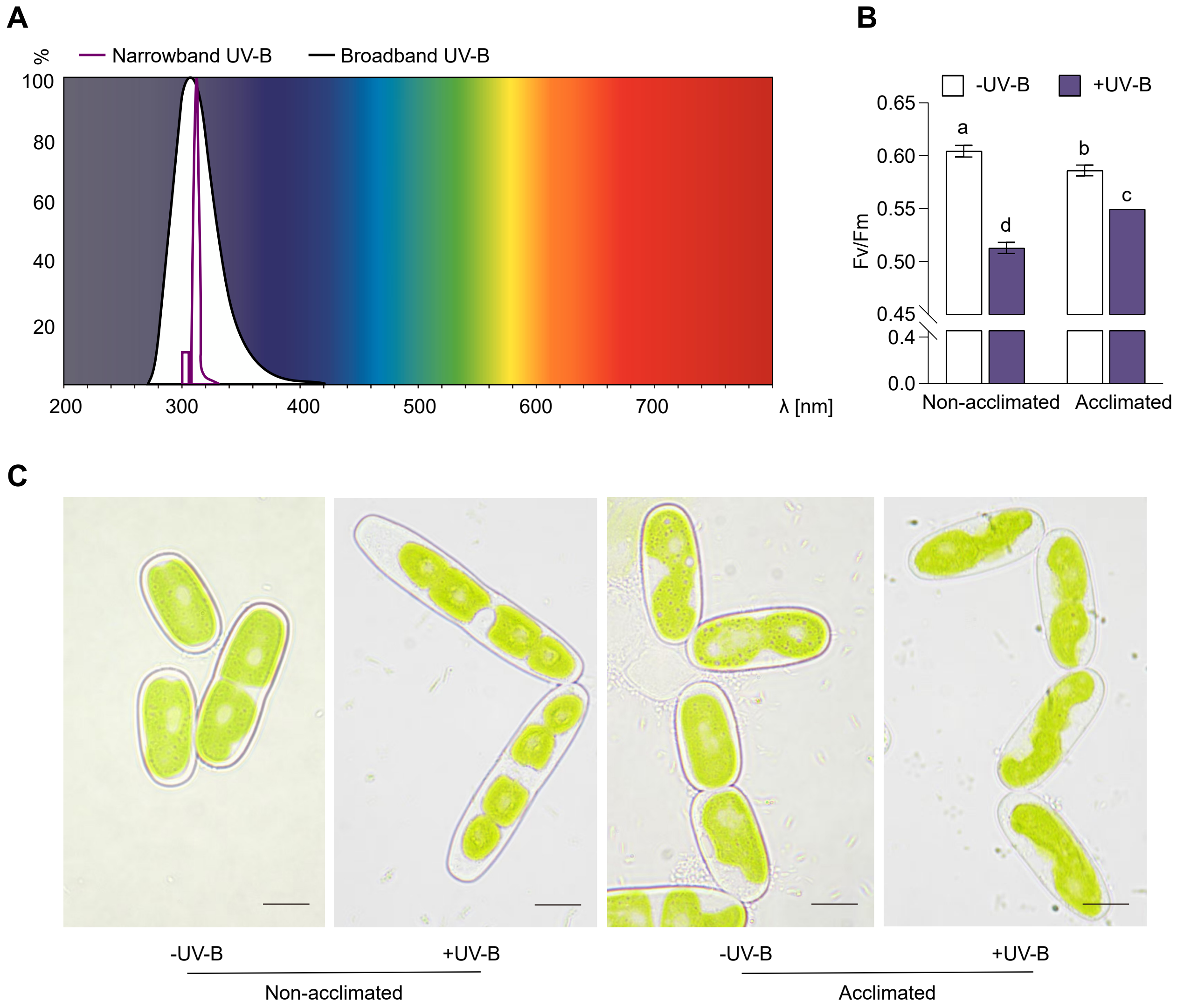

Supplementary Figure 15. Physiological and morphological responses of *M. endlicherianum* to narrowband and broadband UV-B. A, Spectral distribution of the narrowband and broadband UV-B light sources. **B,** Fv/Fm in *M. endlicherianum* under UV-B stress. Data were recorded after 3 h of broadband UV-B irradiation following 3 days of narrowband UV-B acclimation (or in non-acclimated controls). **C,** Representative images showing the cell morphology of *M. endlicherianum* under UV-B stress with or without prior narrowband UV-B acclimation. Scale bar, 100 nm.

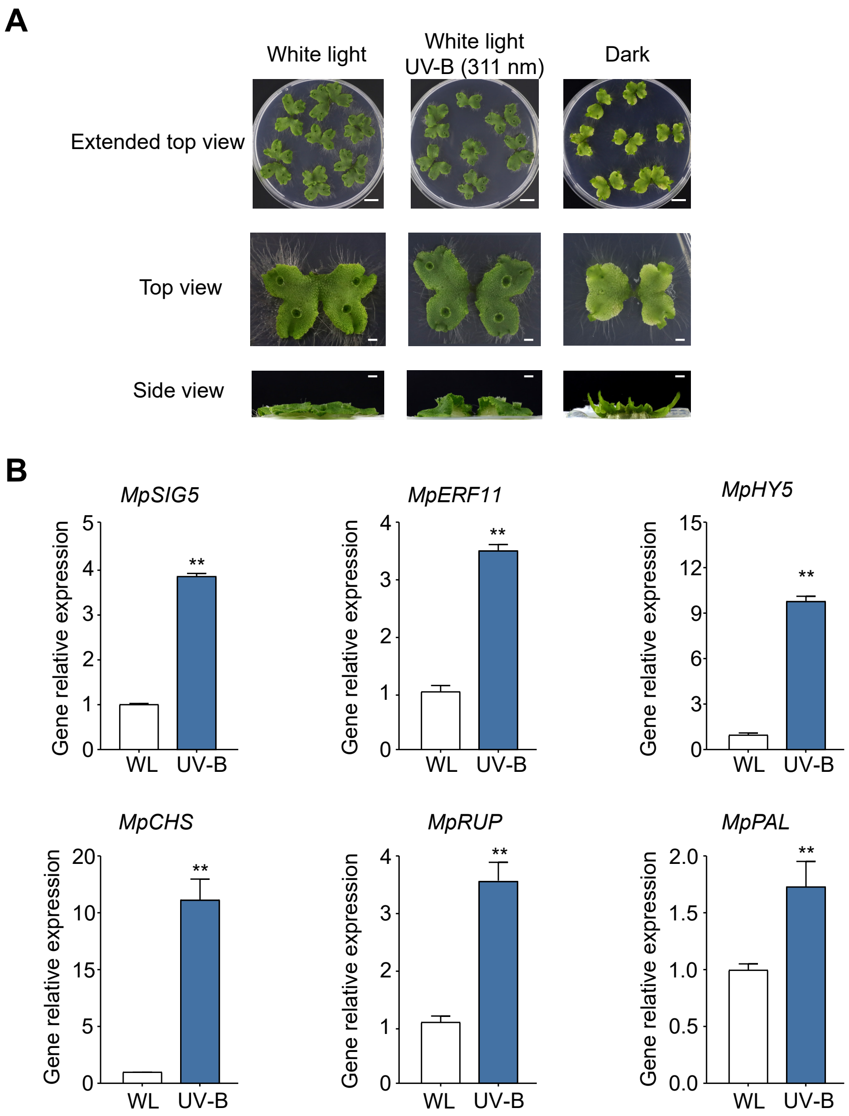

Supplementary Figure 16. UV-B-induced morphological and transcriptional responses in *M. polymorpha*. **A**, Morphology of 12-day-old *M. polymorpha* gemmalings (Tak-1, wild-type, male) after 7-day treatments under different light conditions. Scale bars: 1 cm (extended top view); 200 µm (top view and side view). **B**, Relative expression levels of UV-B-responsive genes under white light (WL) and WL + UV-B treatments in *M. polymorpha*. Data are presented as means ± SD (n = 3). ** denotes a statistically significant difference compared to control (Student's *t*-test, $p < 0.01$).

A

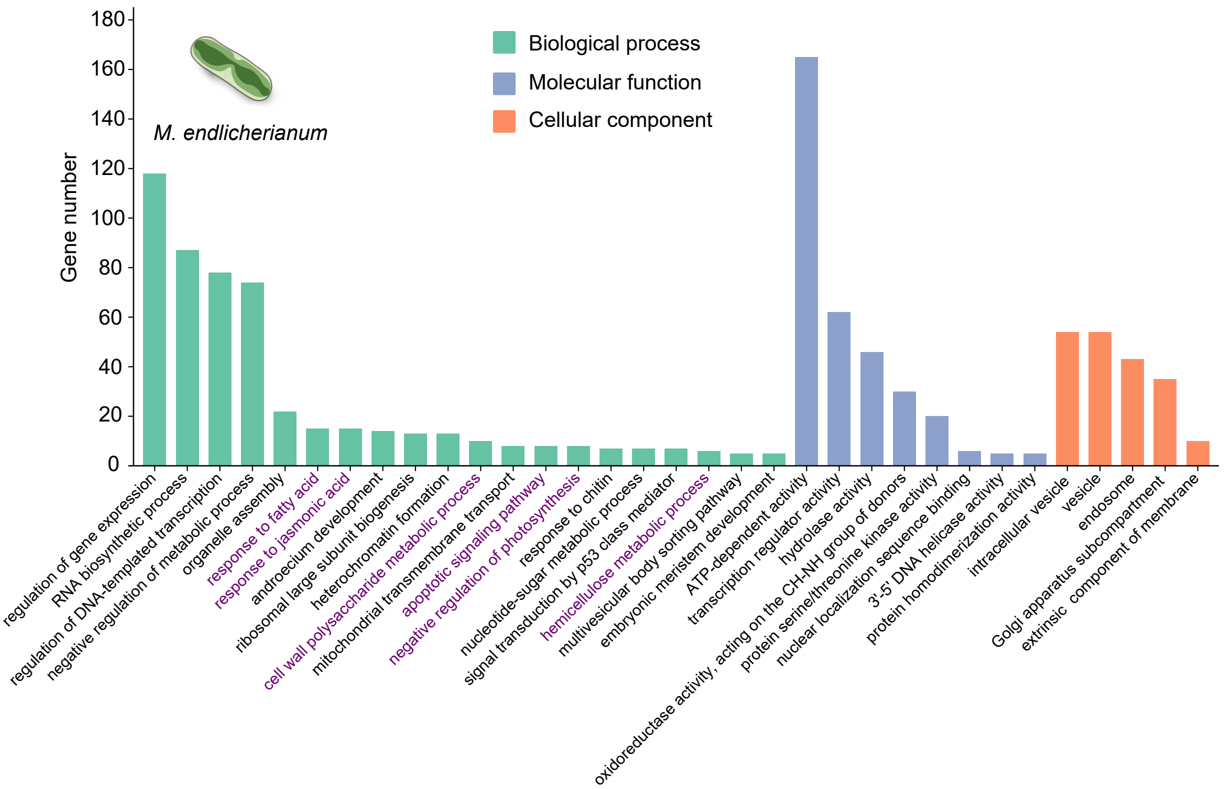

B

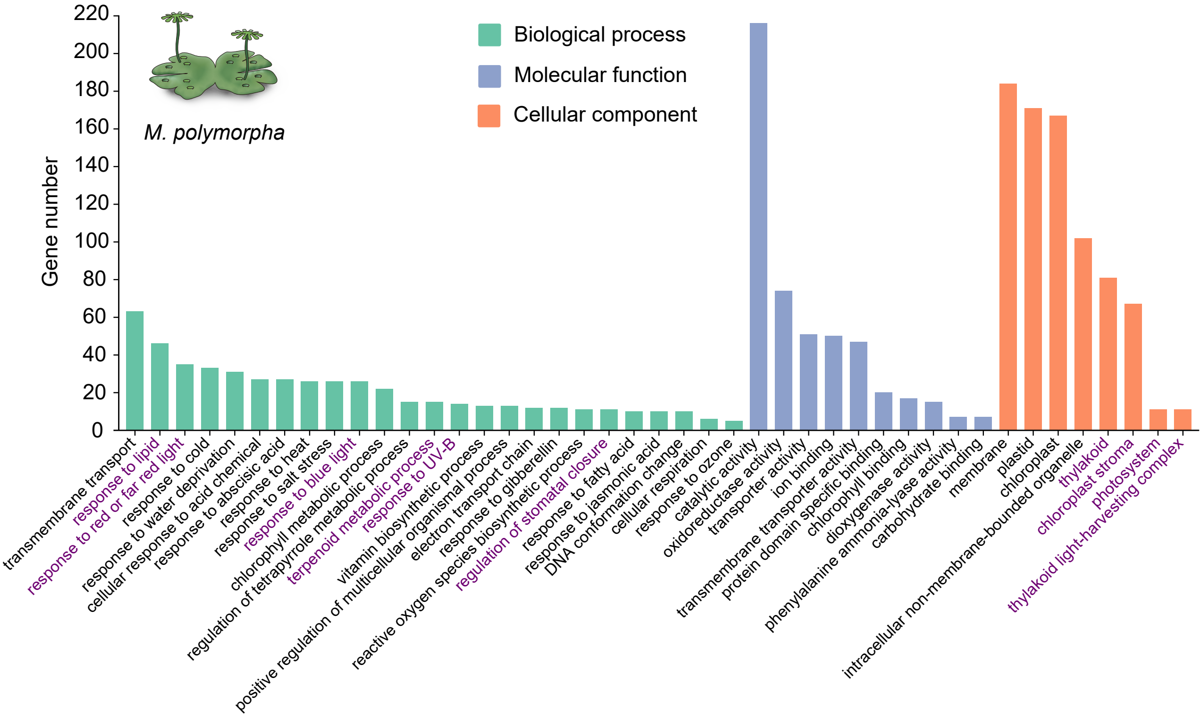

Supplementary Figure 17. GO enrichment analysis of UV-B-responsive DEGs in *M. endlicherianum* and *M. polymorpha*. **A**, GO enrichment of UV-B-responsive DEGs identified in *M. endlicherianum*. **B**, GO enrichment of UV-B-responsive DEGs identified in *M. polymorpha*.

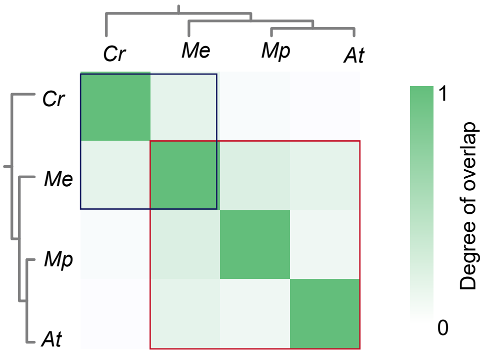

Supplementary Figure 18. Pairwise overlap of UV-B-responsive gene orthogroups across selected plant species. Heatmap color intensity reflects the degree of overlap in UV-B-responsive orthogroups (Green: high; white: low). Boxes highlight comparisons in which the proportion of shared orthogroups exceeds 60% (0.206), indicating relatively conserved UV-B-responsive transcriptional programs.

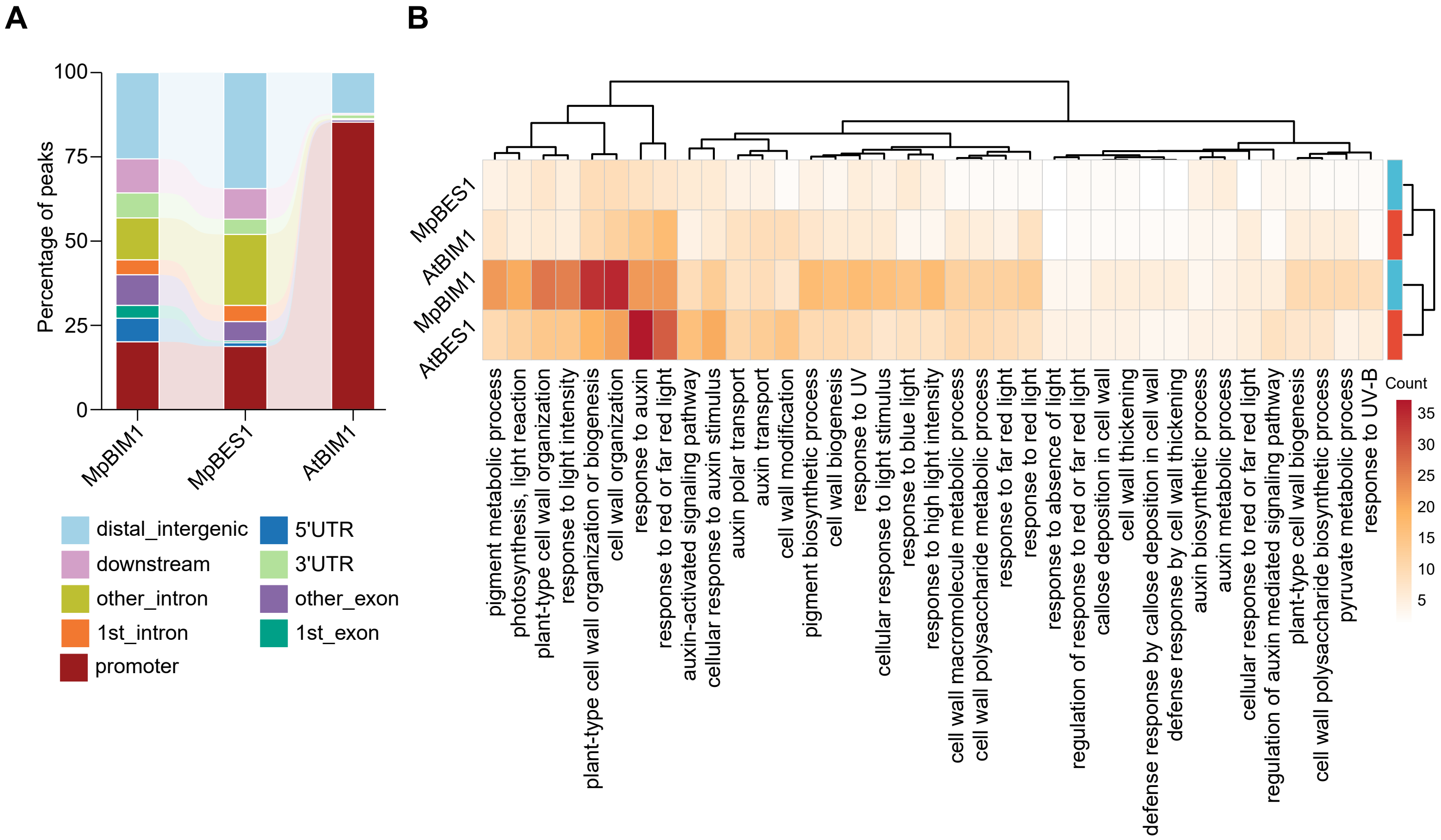

Supplementary Figure 19: Genome-wide binding sites distribution and functional annotation of BIM1 and BES1. A, Genomic distribution of MpBIM1, MpBES1, and AtBIM1 binding sites. **B,** GO enrichment analysis of putative target genes bound by BIM1 and BES1 in *M. polymorpha* and *A. thaliana*.

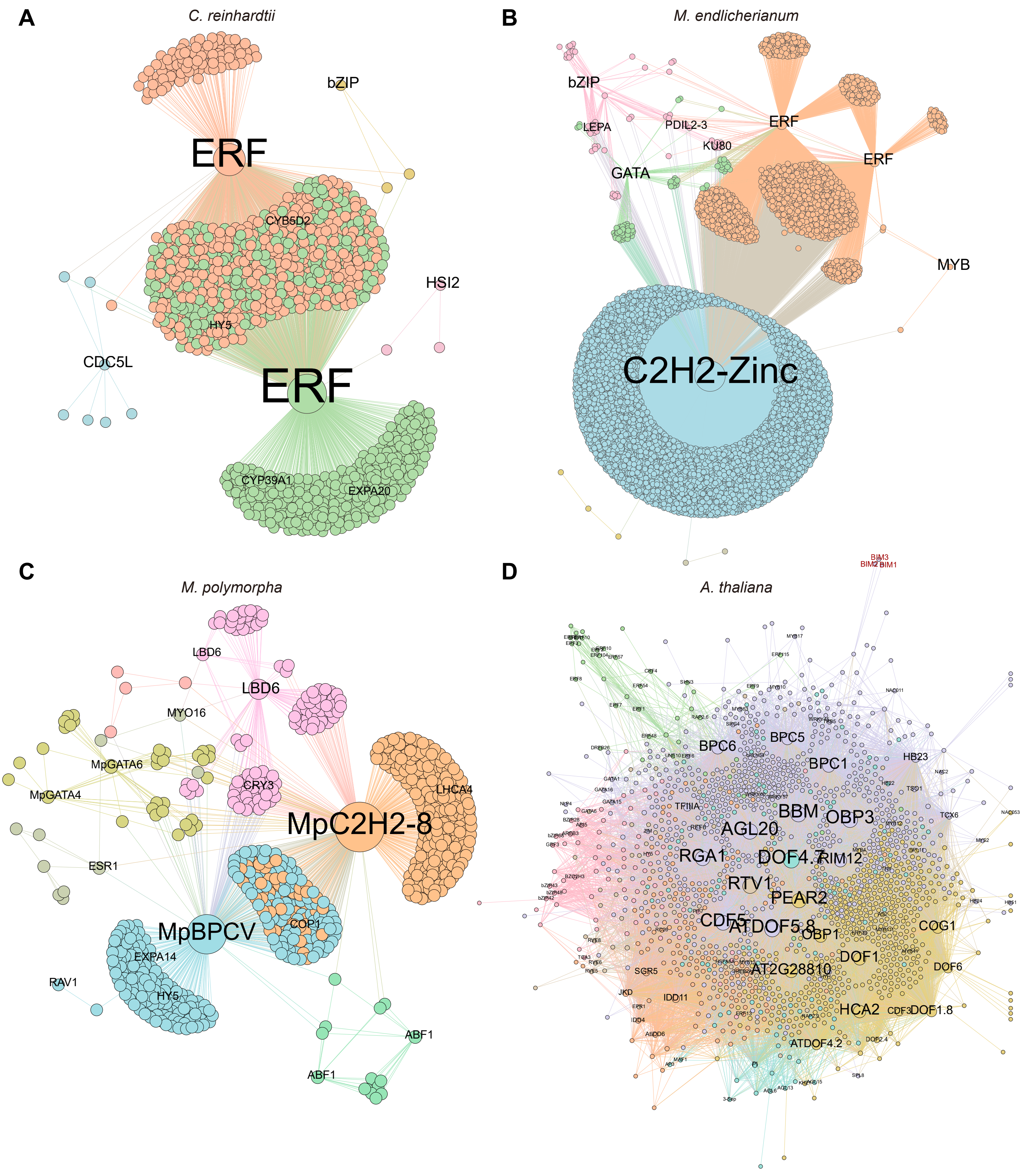

Supplementary Figure 20. Inferred UV-B gene regulatory networks in algae and land plants. A, *C. reinhardtii*. **B,** *M. endlicherianum*. **C,** *M. polymorpha*. **D,** *A. thaliana*.

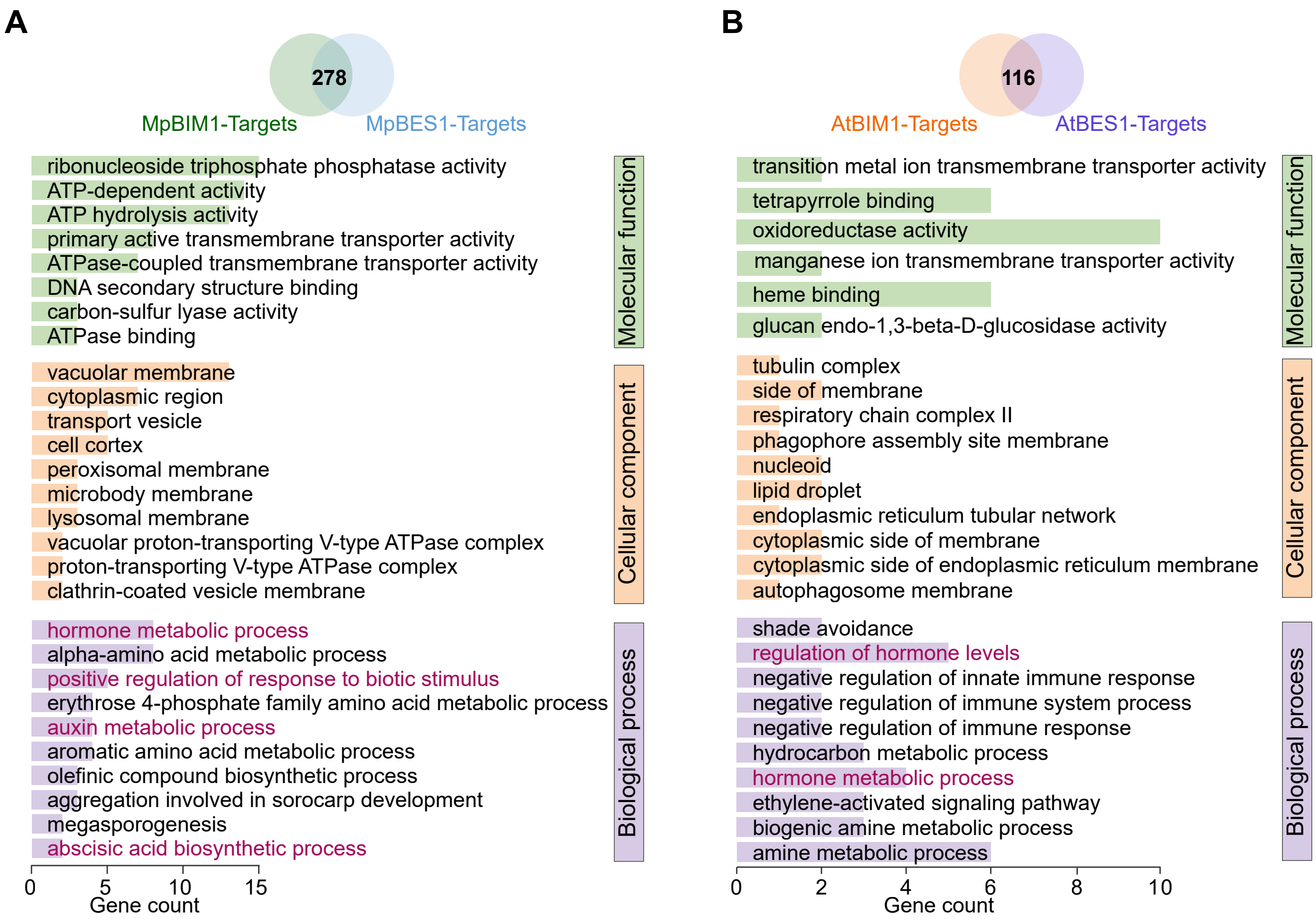

Supplementary Figure 21. Functional enrichment of BIM1/BES1 shared targets. A,B, GO enrichment analysis of the overlapping target genes shared by MpBIM1 and MpBES1 (A) and by AtBIM1 and AtBES1 (B).

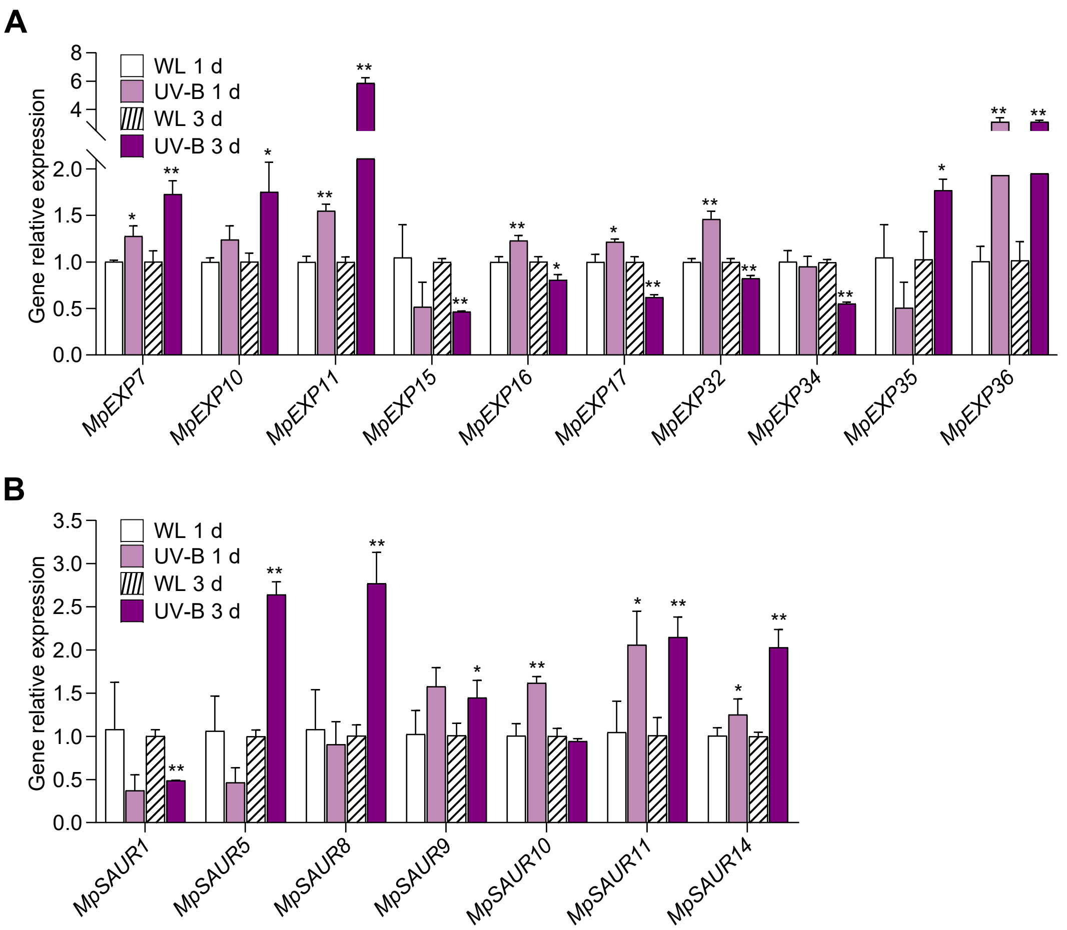

Supplementary Figure 22. Relative expression levels of *EXPANSIN* and *SAUR* gene families. A,**B**, Relative expression levels of *EXPANSIN* (A) and *SAUR* (B) genes under WL and WL + UV-B treatments in *M. polymorpha*. Data are presented as means ± SD (n = 3). *p* values were calculated using two-tailed Student's *t*-tests. *$p < 0.05$; **$p < 0.01$.
