# Supplementary material for: Functional evolution and rewiring of the UVR8–BES1/BIM1 module underpin the refinement of UV-B responses during plant terrestrialization
Source: Plant Commun. 2026 Apr 3;7(6):101842. doi: 10.1016/j.xplc.2026.101842 (PMC13261680; doi:10.1016/j.xplc.2026.101842)
Supplement: Document S2. Article plus supplemental information [file mmc13.pdf]

Plant Communications
Research article

CellPress
Partner Journal

Functional evolution and rewiring of the UVR8–BES1/BIM1 module underpin the refinement of UV-B responses during plant terrestrialization

Chengjuan Cao, Runjie Diao, Mengru Zhao, Qiuting Ji, Jingwen Wang, Zilong Xu, Wenhui Xie, Yujun Zhou, Zhenhua Zhang* and Bojian Zhong*

State Key Laboratory of Microbial Technology, College of Life Sciences, Ministry of Education Key Laboratory of NSLSCS, Nanjing Normal University, Nanjing 210023, China

***Correspondence: Zhenhua Zhang (zhzhang@njnu.edu.cn), Bojian Zhong (bjzhong@gmail.com)**

https://doi.org/10.1016/j.xplc.2026.101842

ABSTRACT

The UVR8–BES1/BIM1-mediated crosstalk between UV-B and brassinosteroid (BR) signaling orchestrates transcriptional reprogramming and thereby coordinates BR-mediated growth and UV-B responses in flowering plants. However, when the UVR8–BES1/BIM1 module originated and how this transcriptional regulatory network evolved in plants remain largely unknown. Here, we traced the evolutionary trajectory of the UVR8–BES1/BIM1 module using a structure-guided approach that integrates homology modeling and structural alignment across major plant lineages. By integrating protein interaction modeling, transcriptome profiling, and genome-wide binding analyses, we elucidated the functional evolution of the UVR8–BES1/BIM1 module driven by structural innovations and genetic co-option. Our results reveal that UVR8 and BIM1 orthologs originated in the last common ancestor (LCA) of chlorophytes and maintained a conserved interaction in green plants, whereas BES1 orthologs emerged in the LCA of streptophyte algae and acquired the capacity to interact with UVR8 in vascular plants. BIM1 served as a core UV-B-responsive transcription factor in the LCA of green plants. By contrast, BES1 initially participated in UV-B signaling through a BIM1-dependent mechanism in the LCA of land plants and later evolved to function as a dominant integrator within UV-B–BR crosstalk in angiosperms. The expansion of the BES1 regulatory network and its binding specificity largely parallels the elaboration of UV-B transcriptional programs during land plant evolution. Our study thus demonstrates that the functional evolution of the UVR8–BES1/BIM1 module enables the stepwise integration of UV-B and BR signaling in green plants, advancing our understanding of how plants have wired hormonal and environmental signals to adapt to terrestrial habitats.

Key words: UVR8–BES1/BIM1 module, UV-B signaling, plant terrestrialization, functional evolution, regulatory networks

Cao C., Diao R., Zhao M., Ji Q., Wang J., Xu Z., Xie W., Zhou Y., Zhang Z., and Zhong B. (2026). Functional evolution and rewiring of the UVR8–BES1/BIM1 module underpin the refinement of UV-B responses during plant terrestrialization. Plant Comm. **7**, 101842.

INTRODUCTION

The colonization of land by plants, which occurred approximately 480 million years ago, fundamentally altered the light environments they inhabit (Maberly, 2014; Morris et al., 2018; Furst-Jansen et al., 2020). UV-B wavelengths (280–315 nm) can damage DNA molecules and also induce plant photomorphogenesis and UV-B acclimation responses (Reyero-Saavedra et al., 2023). Although algae typically experience low doses of UV-B, which are attenuated by water, they have developed a UV-B signaling pathway that is mediated by the photoreceptor UV resistance locus 8 (UVR8) and induces UV-B acclimation (Rozema et al., 2002; Fernandez et al., 2016; Tilbrook et al., 2016). By contrast, land plants must confront highly dynamic UV-B radiation and have evolved sophisticated mechanisms to integrate UV-B signaling with other signaling pathways and achieve UV-B acclimation (Zhang et al., 2022a). Given the broad effects of UV-B on plants, it is crucial to understand the evolution of UV-B signaling and its integration with plant development and adaptation through the differential expression of numerous genes.

UVR8 serves as the UV-B photoreceptor in green plants and is functionally conserved from green algae to flowering plants

 © 2026 The Author(s). Published by Elsevier Inc. on behalf of CAS Center for Excellence in Molecular Plant Sciences, Chinese Academy of Sciences, and Chinese Society for Plant Biology. This is an open access article under the CC BY license (http://creativecommons.org/licenses/by/4.0/).

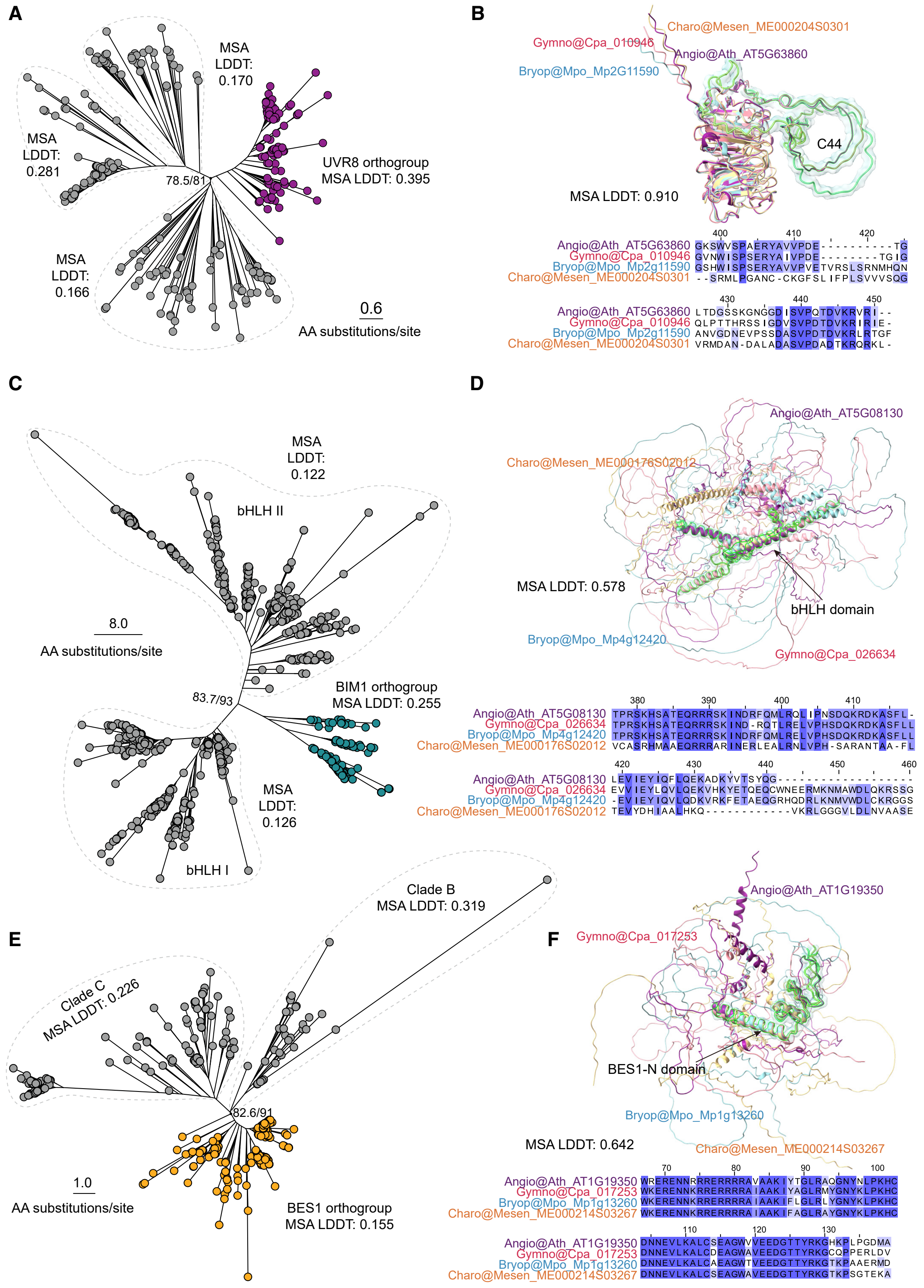

Figure 1. Evolutionary history of UVR8, BES1, and BIM1 revealed by structure-guided phylogeny.

(A) Phylogenetic tree of UVR8 orthologs. The tree was generated by IQ-TREE 2 using the WAG+F+R8 model with 1000 ultrafast bootstraps (UFBS). The UVR8 orthogroup is indicated with purple circles.

(B) Structural alignment of representative UVR8 orthologs. Sequence alignment of the C44 region of UVR8 is shown.

(legend continued on next page)

(Podolec et al., 2021). UV-B-activated UVR8 signaling occurs mainly in the nucleus: (1) in the absence of UV-B, UVR8 forms an inactive homodimer; and (2) upon UV-B exposure, UVR8 uses tryptophan residues as chromophores to sense UV-B radiation, triggering a conformational change from an inactive homodimer to an active monomer. The active monomers interact with key downstream regulators to transmit UV-B signals, thereby modulating gene expression, evoking different physiological responses, and enhancing UV-B tolerance (Liang et al., 2019; O'Hara and Jenkins, 2012; Rizzini et al., 2011). Two different mechanisms of UVR8 signaling have been reported: (1) UVR8 interacts with the E3 ubiquitin ligase COP1 (constitutively photomorphogenic 1), thereby regulating COP1-targeted transcription factors (TFs) that in turn alter gene expression; and (2) UVR8 directly interacts with specific TFs, altering their binding to DNA and thus the transcription of their target genes (Oravecz et al., 2006; Hayes et al., 2014; Liang et al., 2018, 2019; Yang et al., 2018). The UVR8–COP1 pathway is evolutionarily conserved from green algae to flowering plants; however, it is unclear whether the direct action of UVR8 on specific TFs is functionally conserved (Han et al., 2019). BES1 (BRI1–EMS-suppressor 1) and BIM1 (BES1-interacting Myc-like 1) are representative TFs that directly interact with UVR8. In *Arabidopsis thaliana*, the UVR8–BES1/BIM1 module forms a key regulatory nexus linking exogenous UV-B signaling with endogenous brassinosteroid (BR) responses to balance growth–stress trade-offs (Liang et al., 2018, 2020). UV-B-activated UVR8 monomers directly interact with the non-phosphorylated forms of BES1 and BIM1, inhibiting their DNA-binding activity. This downregulates the expression of growth-promoting genes, suppressing cell elongation and promoting photomorphogenesis (Liang et al., 2018). Notably, BES1 also contributes to UV-B acclimation by promoting flavonoid accumulation in a UVR8-independent manner (Liang et al., 2020; Shi and Liu, 2021). Sequence-based homology searches indicated that *UVR8* and *BIM1* orthologs originated in the last common ancestor (LCA) of chlorophytes, whereas *BES1* orthologs emerged in the LCA of streptophyte algae (Delaux et al., 2019; Zhang et al., 2022a). However, the origin and functional evolution of the UVR8–BES1/BIM1 module in mediating UV-B signaling across green plant lineages is poorly understood.

The regulatory capacity of the UVR8–BES1/BIM1 module depends on the structural organization and intrinsic DNA-binding properties of BES1 and BIM1. AtBIM1 (AT5G08130) belongs to the typical basic helix–loop–helix (bHLH) TF family, and the His/Lys/Arg(i$-$4), Glu(i), Arg(i+3), and Arg(i+4) residues (H/K/RxxxExxRR motif) in the bHLH domain collectively determine its binding specificity toward G-box elements (CACGTG). AtBES1 (AT1G19350) exhibits a bHLH-like architecture characterized by a β-hairpin immediately following the first α-helix, an intervening loop, and a truncated second α-helix (Yin et al., 2005; Ezer et al., 2017; Nosaki et al., 2018). This distinctive structural configuration induces more pronounced conformational changes in both the major and minor grooves of bound DNA, thereby relaxing sequence constraints and conferring on AtBES1 a broader capacity for *cis*-element recognition (Nosaki et al., 2022). AtBES1 can bind not only G-box but also E-box motifs (CANNTG) and the BR-response element (BRRE, CGTG$^{C}/_{T}$G) (Yin et al., 2005; Yu et al., 2011). Moreover, heterodimer assembly between BES1 and typical bHLH TFs may enable a key Glu (E) residue to bind into the major groove of the E-box motif by making use of the tilt-angle variation of the DNA recognition helix with a conformational change in dimerization regions, coordinating the crosstalk between phytohormone and environmental signaling (Oh et al., 2012; Nosaki et al., 2018). Thus, exploring the evolutionary trajectories of BES1 and BIM1 across major green plant lineages is essential for understanding the evolutionary plasticity of UV-B signaling.

In this study, we used AlphaFold2-based structural modeling to systematically investigate the origin and functional evolution of the UVR8–BES1/BIM1 module in green plants. By integrating structure-guided phylogenies, protein–protein interaction (PPI) assays, and comparative omics analyses, we revealed how UV-B and BR signaling pathways evolved to interconnect during land plant evolution. Our findings provide new insights into the multi-signal integration of light and hormone signaling networks and highlight the emergence of the UVR8–BES1/BIM1 module as a key innovation that enabled plants to adapt to changing light environments on land.

RESULTS

Evolution of UVR8, BES1, and BIM1 revealed by structure-guided phylogenies

We used a structure-guided phylogenetic framework to characterize the evolutionary trajectories of the UVR8–BES1/BIM1 module in green plants, integrating deep learning-based protein structure prediction with structural alignment (Kim et al., 2025). The structure-guided phylogenies identified UVR8, BIM1, and BES1 orthologs, with orthologous groups exhibiting higher pairwise structural similarity than paralogous groups (Figure 1 and Supplemental Figures 1–3 and 7). The results provided further support for the notion that *UVR8* and *BIM1* orthologs originated in the LCA of chlorophytes and that the *BES1* ortholog emerged in the LCA of streptophyte algae, a scenario largely congruent with tree topologies produced by the sequence-based approach (Zhang et al., 2022a; Figure 1 and Supplemental Figures 4–6). Structure-guided analyses markedly improved the resolution of ortholog/paralog discrimination, particularly in ancient and highly divergent clades (Figure 1). For example, two UVR8-like sequences from the green algae *Chloroidium sp*. (Chsp_0000611-CF150.5) and *Ulva mutabilis* (Ulvm-u_UM015_0123.1) were misclassified as UVR8 orthologs on the

(C) Phylogenetic tree of BIM1 orthologs. The tree was generated by IQ-TREE 2 using the JTT+F+R10 model with 1000 UFBS. The BIM1 orthogroup is indicated with cyan circles.

(D) Structural alignment of representative BIM1 orthologs. Sequence alignment of the bHLH domain is shown.

(E) Phylogenetic tree of BES1 orthologs. The tree was generated by IQ-TREE 2 using the JTT+F+R7 model with 1000 UFBS. The BES1 orthogroup is indicated with yellow circles.

(F) Structural alignment of representative BES1 orthologs. Sequence alignment of the N-terminal region is shown. Multiple sequence alignment LDDT scores quantify local atomic distance deviations across multiple sequence alignments and are used to assess structural alignment quality.

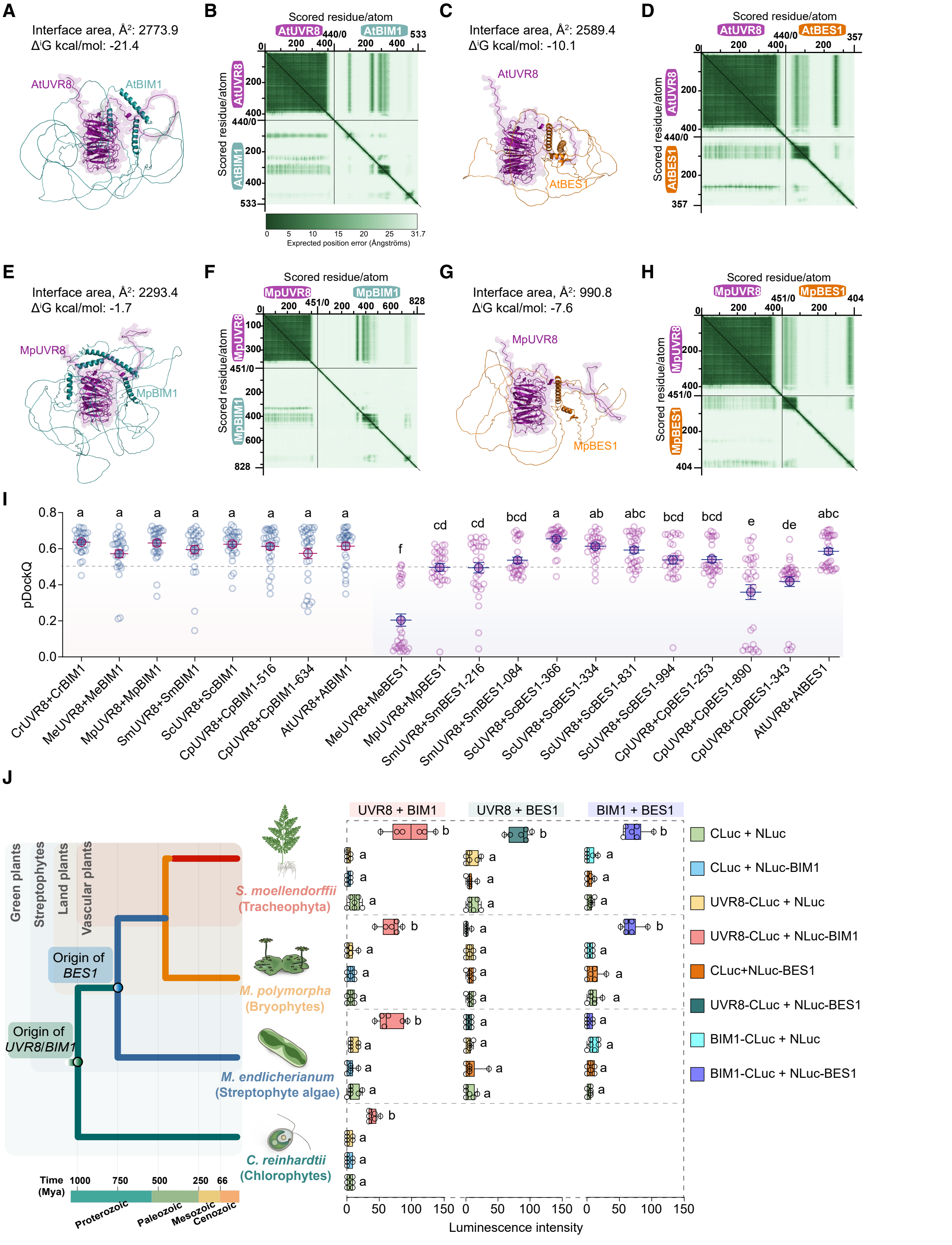

Figure 2. Stepwise establishment of protein–protein interactions among UVR8, BES1, and BIM1 orthologs across green plants.
(A–H) Examples of predicted structures and predicted alignment error (PAE) heatmaps for UVR8–BIM1 and UVR8–BES1 dimers in *A. thaliana* **(A–D)** and *M. polymorpha* **(E–H)**. Dark green regions in the interaction quadrants of PAE denote low expected position error, indicative of protein–protein

(legend continued on next page)

basis of sequence similarity but were excluded by structure-guided analyses. Inspection of their structures revealed that Chsp_0000611-CF150.5 exhibits structural features corresponding to only 3 of the 7 blades of the 7-bladed β-propeller fold (the core structure of canonical UVR8), and Ulvmu_UM015_0123.1 has a C-terminal extension of approximately 320 amino acids located outside this conserved fold (Figure 1A and Supplemental Figure 8A). These findings demonstrate the limitations of sequence-based phylogeny in resolving deeply divergent orthologs and the enhanced accuracy of structure-guided approaches for ortholog assignment.

A well-supported monophyletic BIM1 orthogroup was present in the structure-guided phylogeny (Figure 1B). Noting that BIM1 harbors more intrinsically disordered regions (IDRs), we quantified the proportion of disordered residues across all analyzed sequences and found that it was significantly higher in the BIM1 orthogroup than in the other two major bHLH clades (Supplemental Figures 8B and 8C). To evaluate the phylogenetic contribution of these regions, we constructed a phylogenetic tree with IDRs excluded. Although the overall topology remained largely congruent, the inclusion of IDRs provided substantially higher bootstrap support for the BIM1 clade and was essential for accurate phylogenetic placement of algal lineages (Supplemental Figure 9). For BES1 orthologs, structure-guided phylogenies resolved three major clades (A, B, and C), consistent with sequence-based classifications (Furuya et al., 2024; Figure 1C and Supplemental Figure 6). Comparative analysis of the BES1-N domain (the DNA-binding domain) revealed that clade C exhibits distinct structural features in the DNA-binding region relative to clades A and B, particularly in the orientation of the two α-helices (Supplemental Figure 10A and 10B). This divergence resulted from a conserved RERRRR motif in clades A and B, whereas an RERxRR variant occurred in clade C. This structural divergence between different clades of BES1 orthologs was observed in both liverworts (*Marchantia polymorpha*) and monocots (*Oryza sativa*) (Supplemental Figures 10A and 10B). We found that the presence of RERRRR or RERxRR differentially shapes the electrostatic potential of the BES1-N DNA-binding surface and underlies clade-specific DNA-binding specificities (Supplemental Figures 10C and 10D). Ancestral sequence reconstruction further indicated that the divergence of the BES1-N domain originated in the LCA of the BES1 ortholog (Supplemental Figure 10E), supporting an early splitting event that established the distinct DNA-binding specificities of the BES1 clades (Nosaki et al., 2022).

Stepwise establishment of protein–protein interactions among UVR8, BES1, and BIM1 in green plants

We next investigated the potential for direct physical interactions among components of the UVR8–BES1/BIM1 module in green plants. Using SpeedPPI (Bryant et al., 2022; Bryant and Noé, 2023), we evaluated the predicted docking quality (pDockQ scores) and local structure confidence (predicted Local Distance Difference Test, pLDDT scores) of UVR8–BES1 and UVR8–BIM1 complexes in representative species from green algae to land plants. In *A. thaliana*, both the AtUVR8–AtBES1 and AtUVR8–AtBIM1 complexes exhibited low interchain predicted alignment errors (PAEs) and high pDockQ scores (average 0.588 and 0.614, respectively). Given that pDockQ values above 0.5 are indicative of high-confidence PPIs, these results supported the formation of stable AtUVR8–AtBES1 and AtUVR8–AtBIM1 complexes, consistent with previous experimental results (Liang et al., 2018; Figures 2A–2D). Predicted protein structures from other plants exhibited high structural confidence (pLDDT $\geq$ 70) (Figure 2E–2I and Supplemental Figures 11A and 11B), enabling the evaluation of UVR8–BIM1 interactions. These interactions appeared to be conserved from the green alga *Chlamydomonas reinhardtii* to angiosperms, as evidenced by high pDockQ scores (Figures 2E, 2F, and 2I). Analyses of evolutionary rate covariation (ERC) also supported the functional association and coevolution of UVR8 and BIM1 throughout the evolution of green plants (Supplemental Figure 12). By contrast, the UVR8–BES1 complex exhibited low pDockQ scores in the streptophyte alga *Mesotaenium endlicherianum* (average pDockQ = 0.18) and the bryophyte *M. polymorpha* (average pDockQ = 0.49), indicating limited potential for stable interactions (Figures 2G–2I and Supplemental Figures 11C and 11D). In vascular plants, including the lycophyte *Selaginella moellendorffii* (average pDockQ = 0.53), the fern *Salvinia cucullata* (average pDockQ = 0.64), and seed plants (*Cycas panzhihuaensis*, average pDockQ = 0.56; *A. thaliana* 0.58), UVR8–BES1 complexes showed markedly enhanced interaction signals (Figure 2I; Supplemental Figures 11C and 11D). These findings suggest that the capacity for direct UVR8–BES1 interactions is likely greater in vascular plants than in bryophytes.

Because the extensive IDRs in both the BES1 and BIM1 proteins might obscure interface predictions, we sought to experimentally validate the observed PPI patterns. Luciferase complementation imaging (LCI) assays in *Nicotiana benthamiana* confirmed that BIM1 orthologs from green algae to angiosperms specifically interacted with UVR8 (Figure 2J), supporting conservation of the UVR8–BIM1 complex. By contrast, MpBES1 from *M. polymorpha* did not directly interact with MpUVR8 but was able to interact with MpBIM1 (Figure 2J). In the lycophyte *S. moellendorffii*, direct SmUVR8–SmBES1 interactions were detected, suggesting that UVR8 acquired the ability to directly modulate BES1 function independently of BIM1 (Figure 2J). These results support an evolutionary model in which BES1 initially participated in UV-B signaling indirectly through BIM1-mediated bridging (UVR8–BIM1–BES1) and subsequently gained

interactions. $\Delta^{i}G$ indicates the solvation free energy gain upon formation of the interface (in kcal mol^{-1}); lower $\Delta^{i}G$ values and larger interface areas indicate a higher likelihood of physical interaction.

(**I**) pDockQ scores of UVR8–BIM1 and UVR8–BES1 across species. Each dot represents one prediction replicate. pDockQ $\geq$ 0.5 indicates high-confidence interaction predictions.

(**J**) Luciferase complementation assays confirming interactions among UVR8, BES1, and BIM1 orthologs from *C. reinhardtii*, *M. endlicherianum*, *M. polymorpha*, and *S. moellendorffii*. nLUC and cLUC refer to the N- and C-terminal fragments of luciferase, respectively. Data are presented as means ± SD (n = 6). Phylogenetic relationships and divergence time estimates of major plant lineages are based on Bowman (2022). Different letters indicate significant differences (Student's t-test, $p < 0.05$).

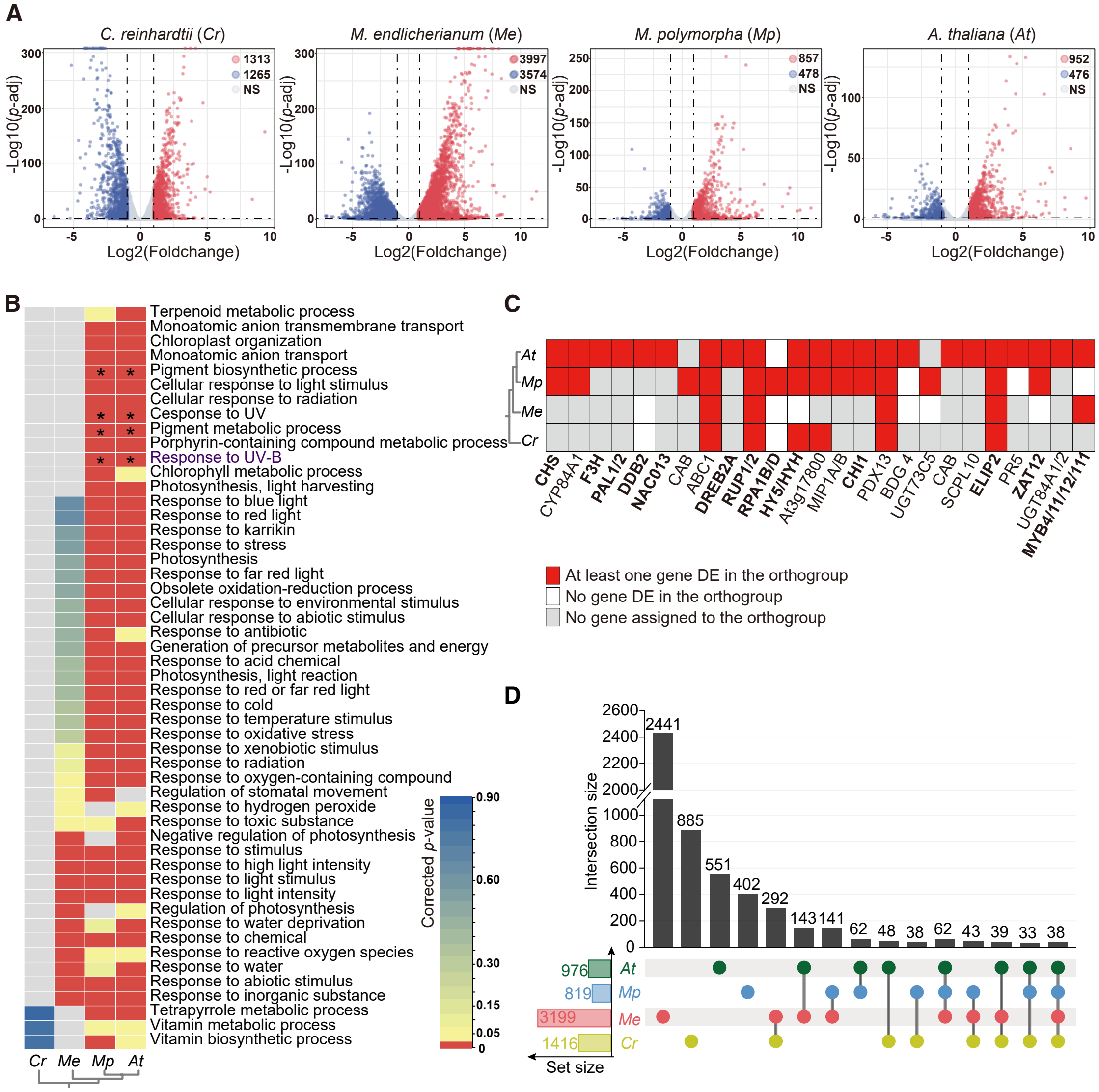

Figure 3. Transcriptomic comparisons of UV-B-responsive genes across representative species of green plants.

(A) Volcano plots showing transcriptional responses to UV-B treatment in *C. reinhardtii*, *M. endlicherianum*, *M. polymorpha*, and *A. thaliana*. *p* adj. < 0.05 and $|\log_2 FC| \geq 1$ were used as significance thresholds. Blue dots represent significantly downregulated genes, red dots represent significantly upregulated genes, and gray dots represent genes whose expression did not change significantly.

(B) GO enrichment analysis of UV-B-responsive DEGs, with red indicating significantly enriched terms (*p* adj. < 0.05). Asterisks indicate GO terms associated with UV-B responses.

(C) Distribution of orthologous genes from UV-B-responsive terms across species.

(D) UpSet plot showing the overlap of orthologous genes identified from UV-B-responsive DEGs across the four species (vertical bars) and the total number of genes per species (horizontal bars).

the ability to directly interact with UVR8 (UVR8–BES1) during the evolution of vascular plants.

Distinct patterns of UV-B-induced transcriptional regulation between green algae and land plants

To investigate the evolution of expression patterns in response to UV-B, we performed comparative transcriptome analyses of four representative green plants: *C. reinhardtii* (Tilbrook et al., 2016), *M. endlicherianum*, *M. polymorpha*, and *A. thaliana* (Tavridou et al., 2020). UV-B treatment induced widespread transcriptional reprogramming in all four species, but the extent and direction of gene expression changes varied among the species (Figure 3 and Supplemental Figures 14–16A). The number of UV-B-responsive genes was highest in *M. endlicherianum* (3997 upregulated and 3574 downregulated), followed by *C. reinhardtii* (1313 upregulated and 1265 downregulated), *A. thaliana* (952 upregulated and 476 downregulated), and *M. polymorpha* (857 upregulated and 478 downregulated) (Figure 3A and Supplemental Table 1). In *C. reinhardtii*, similar numbers of genes were up- and downregulated in response to UV-B. In *M. endlicherianum*, although comparable numbers of genes were up- and downregulated, the upregulated genes tended to exhibit greater significance than the downregulated genes (Figure 3A). This trend

resembled the transcriptional activation bias observed in land plants, in which upregulated genes evidently outnumber downregulated genes by approximately twofold, as seen in both *M. polymorpha* and *A. thaliana*. Given that *M. endlicherianum* represents the phylogenetically closest streptophyte algal lineage to land plants, this pattern may reflect an intermediate evolutionary state of UV-B-responsive transcriptional regulation between algae and land plants (Goldbecker and de Vries, 2025; Hess et al., 2022; Figure 3A). Gene Ontology (GO) analysis of these differentially expressed genes (DEGs) identified significantly enriched GO terms, including response to UV-B, response to UV, pigment biosynthetic process, and pigment metabolic process, in both *M. polymorpha* and *A. thaliana* (these terms were not enriched in algal DEGs) (Figure 3B, Supplemental Figure 17, and Supplemental Table 2). Further examination of genes associated with UV-B-responsive GO terms revealed that *PDX1.3* (pyridoxine biosynthesis 1.3), which is involved in vitamin B6 biosynthesis, and *ELIP2* (early light-inducible protein 2) were conserved across all four species, suggesting that they may serve as an ancestral stress response mechanism. By contrast, flavonoid biosynthesis genes, specifically *CHS* (chalcone synthase) and *CHI1* (chalcone isomerase 1), were robustly induced in both *M. polymorpha* and *A. thaliana* (Figure 3C). Reverse transcription quantitative PCR (RT–qPCR) assays also confirmed the strong UV-B-induced activation of *MpERF11*, *MpCHS*, and *MpPAL* (phenylalanine ammonia-lyase) in *Marchantia* (Supplemental Figure 16B). Originating in the LCA of land plants, these genes appear to have been recruited into the UV-B signaling network to provide critical compounds for UV protection and have been conserved (Tanaka et al., 2008; de Vries et al., 2021; Davies et al., 2024). Orthogroup clustering revealed a comparable set of UV-B-responsive orthologs in *M. polymorpha* (819) and *A. thaliana* (976) (Figure 3D and Supplemental Table 3). Pairwise comparisons between land plants revealed a convergence degree >0.2 for UV-B-responsive gene orthologs, which was significantly higher than that of the alga–land plant pairs (Supplemental Figure 18). These results highlight distinct patterns of transcriptional reprogramming in response to UV-B between green algae and land plants.

Evolutionary expansion of UV-B regulatory networks

Given that BIM1 and BES1 were identified as core UV-B-responsive factors, we performed DNA affinity purification sequencing (DAP-seq) analyses of MpBES1, MpBIM1, and AtBIM1 for further comparison. Motif analysis revealed that the canonical G-box motif (CACGTG), a conserved bHLH *cis*-element (Toledo-Ortiz et al., 2003), was significantly enriched in the binding regions of MpBES1, MpBIM1, and AtBIM1, consistent with observations for AtBES1 (Figure 4A). However, the BRRE (CGTG$^{T}/_{C}$G), a hallmark of AtBES1 targets (Yu et al., 2011), was not significantly enriched in MpBES1-bound regions (Supplemental Table 4). We next filtered peaks within the 2-kb upstream regions of annotated genes and identified 1133 target genes (TGs) for MpBES1, 3690 TGs for MpBIM1, and 963 TGs for AtBIM1 (Supplemental Figure 19 and Supplemental Table 5). By comparing these TGs with UV-B-responsive gene expression profiles, we quantified the regulatory contribution of each TF. MpBIM1 exhibited the most extensive regulatory engagement, with 10.05% (371/3690) of its TGs responding to UV-B signaling. The corresponding percentages for MpBES1, AtBIM1, and AtBES1 were lower: 6.62% (75/1133), 6.96% (67/963), and 5.72% (66/1154), respectively (Figure 4B). GO enrichment analysis highlighted the conserved regulatory role of BIM1: TGs of both MpBIM1 and AtBIM1 were significantly enriched in "terpenoid metabolism" and "hormone biosynthesis/transport." By contrast, the function of BES1 appeared to have diverged: the TGs of MpBES1 were primarily associated with general stress responses (e.g., vitamin and carbohydrate metabolism), whereas the TGs of AtBES1 were specifically enriched in "response to UV-B" and "cell wall modification" (Figure 4B).

To gain a holistic view of the UV-B transcriptional regulatory landscape, we constructed high-confidence gene regulatory networks (GRNs) using the UV-B-responsive DEGs. By incorporating the identified TGs into regulatory prediction models, we found that the *M. polymorpha* GRN (comprising 10 TFs and 867 edges) was relatively dispersed, with MpBIM1 emerging as a primary hub that directly regulated a large number of targets (Figure 4C and Supplemental Table 6). By contrast, the *A. thaliana* GRN (comprising 34 TFs and 3338 edges) had evolved into a highly dense and complex topology. Although AtBIM1 and AtBES1 remained integral components, they were part of a larger regulatory ensemble, with the MADS-box TF AGL20 emerging as a dominant hub (Figure 4D and Supplemental Table 6). Next, we expanded the comparative analysis and constructed predictive GRNs across four species (*C. reinhardtii*, *M. endlicherianum*, *M. polymorpha*, and *A. thaliana*) without restricting the TFs to DEGs. The results showed that HY5 and its orthologs were present in all four species, establishing HY5 as an ancient and conserved component of UV-B signaling (Supplemental Figure 20). The GRNs (from *C. reinhardtii* and *M. endlicherianum* to *M. polymorpha* and *A. thaliana*) evolved from simple network topologies (comprising only a few TFs, such as ERF and C2H2) to sophisticated, hierarchically layered architectures that incorporated diverse TF families (e.g., DOF, BPC, LBD, GATA, and MYB) (Rieseberg et al., 2025; Supplemental Figure 20).

Diversified *cis*-regulatory control of BES1/BIM1 in mediation of the UV-B response

To explore the conservation and divergence of downstream regulatory targets, we compared BES1 and BIM1 TGs between *M. polymorpha* and *A. thaliana*. We observed numerous overlapping TGs between BIM1 and BES1 within each species (MpBIM1 and MpBES1: 290 orthogroups [OGs], including 734 TGs; AtBIM1 and AtBES1: 103 OGs, including 276 TGs), indicating strong functional interdependence between these two factors (Figure 5A and Supplemental Figure 21). Interestingly, MpBIM1 shared substantially more orthologous TGs with AtBES1 (137 OGs) than with AtBIM1 (83 OGs) (Figure 5A and Supplemental Table 7), indicating a greater regulatory resemblance between MpBIM1 and AtBES1. Moreover, these conserved target clusters consisted predominantly of expanded gene families, including several gene families associated with axillary meristems, auxin, and cell expansion: *RAX3*, *CYP450*, *SAUR*, and *expansin* gene families (Supplemental Table 8; Zeng et al., 2024). Kyoto Encyclopedia of Genes and Genomes (KEGG) analysis of the 62 OGs shared by MpBIM1, MpBES1, AtBIM1, and AtBES1 revealed significantly enriched pathways: plant hormone signal transduction, biosynthesis of secondary metabolites, and BR biosynthesis (Figure 5B and Supplemental Table 9). RT–qPCR analysis showed

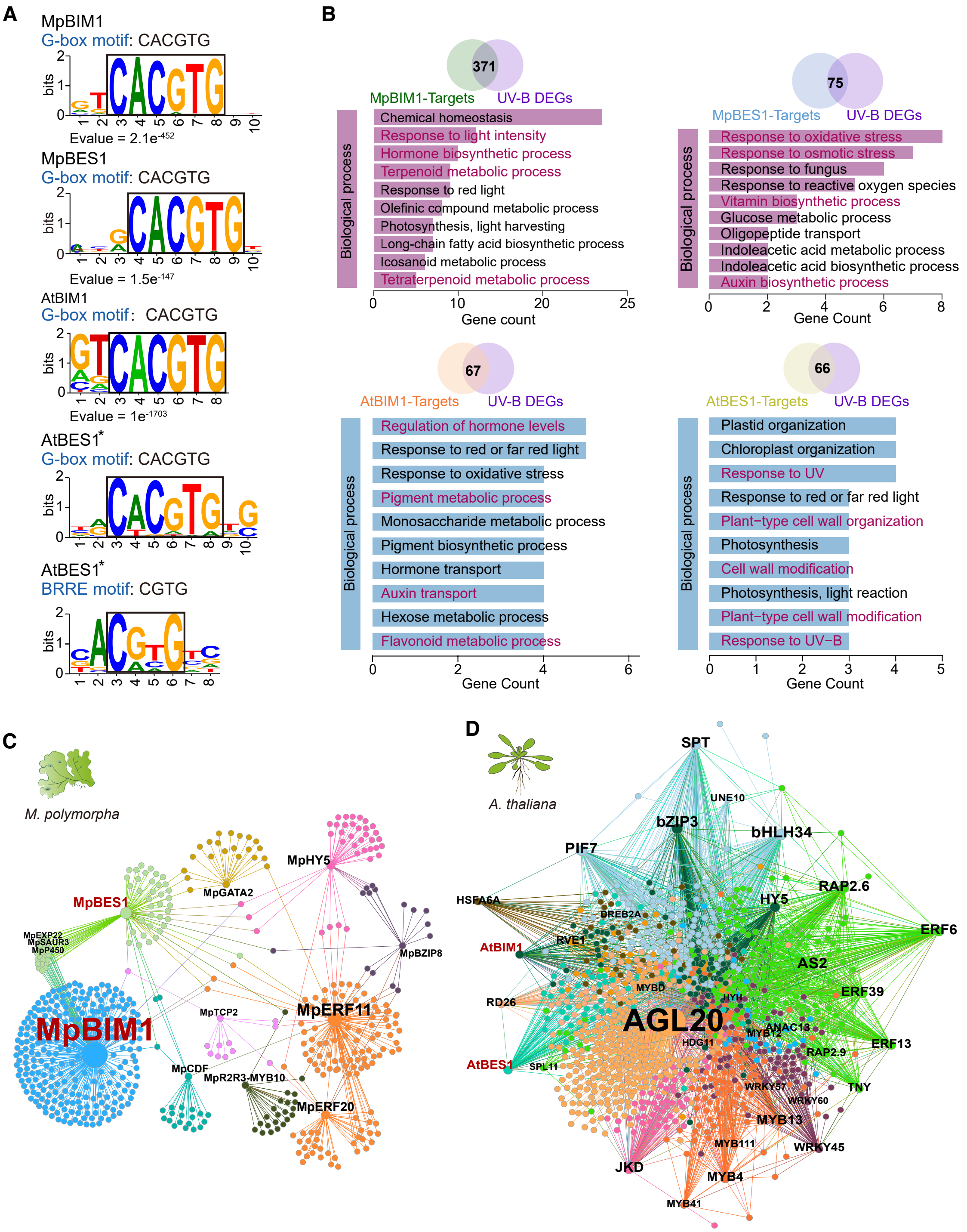

Figure 4. Diversification of *cis*-regulatory control of BES1/BIM1 in the UV-B response.

(A) Top enriched DNA motifs in BIM1- and BES1-binding regions. AtBES1 data were obtained from a previous study by Yu et al.

(B) GO enrichment analysis of overlapping genes between BIM1/BES1 targets and UV-B-responsive DEGs. Terms potentially associated with UV-B responses are highlighted in purple.

(C and D) High-confidence gene regulatory networks inferred from UV-B-responsive DEGs in *M. polymorpha* **(C)** and *A. thaliana* **(D)**. Different colors denote different modules.

that several members of these gene families were significantly differentially expressed in response to UV-B treatment. For example, *MpSAUR1*, and *MpSAUR3* were downregulated, whereas *MpSAUR6*, *MpEXP4*, *MpEXP20*, and *MpEXP22* were up-regulated (Figure 5C and Supplemental Figure 22). Dual-luciferase reporter (DLR) assays demonstrated that MpBIM1 transcriptionally activated the *MpSAUR3* promoter while repressing the *MpEXP22* promoter in *N. benthamiana*. Co-expression with

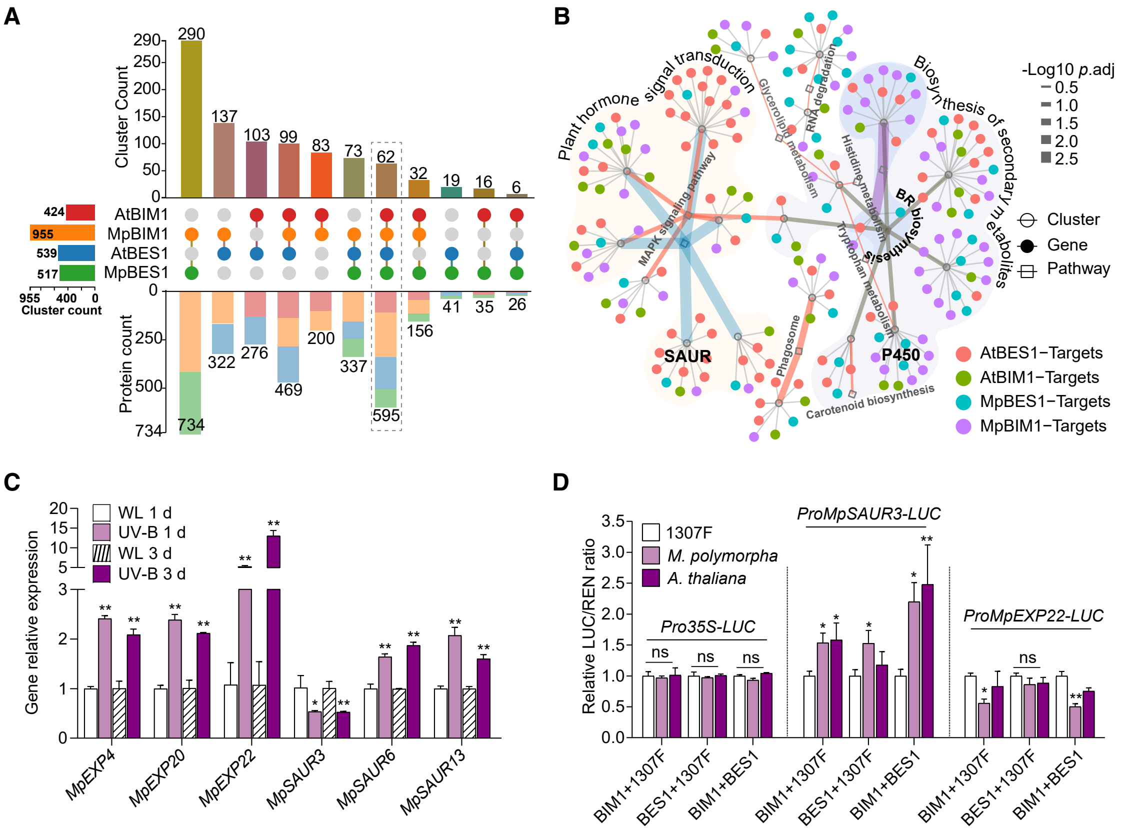

Figure 5. Conservation of BES1/BIM1 regulatory targets across species.

(A) UpSet plot showing the overlap of DAP-seq targets among MpBIM1, MpBES1, AtBIM1, and AtBES1. Vertical and horizontal bars represent intersection sizes and total orthogroups per TF, respectively.

(B) KEGG pathway network of shared orthogroups targeted by MpBES1, MpBIM1, AtBIM1, and AtBES1.

(C) Relative expression levels of *SAUR/expansin* family genes under white light (WL) and WL + UV-B treatments in *M. polymorpha*. Data are presented as means ± SD ($n = 3$).

(D) Dual-luciferase reporter (DLR) assays testing the transcriptional regulation of selected target promoters by BES1 and BIM1. Data are presented as means ± SD ($n = 6$). p values were calculated using two-tailed Student's t test. *$p < 0.05$; **$p < 0.01$.

MpBES1 significantly enhanced *MpSAUR3* activation, whereas repression of *MpEXP22* was unaffected (Figure 5D). AtBIM1 could similarly activate *MpSAUR3*, and co-expression with AtBES1 further potentiated this activation. By contrast, the regulation of *MpEXP22* appeared to be specific to MpBIM1, as it was not effectively regulated by AtBIM1 or AtBES1 (Figure 5D). These analyses suggest that MpBIM1, rather than MpBES1, may perform regulatory functions analogous to those of AtBES1 in early-diverging land plants.

DISCUSSION

During plant terrestrialization, the emergence of core components of UV-B signaling pathways in green algae (chlorophytes and streptophyte algae) facilitated adaptation to the variable UV-B radiation in subaerial environments (Zhang et al., 2022b). The UVR8–BES1/BIM1 signaling module integrates environmental light signals and endogenous phytohormones to coordinate plant growth and development (Liang et al., 2018). In this study, we combined structure-guided phylogeny, comparative transcriptomics, DAP-seq analyses, and experimental validation to uncover the structural innovations and regulatory mechanisms underlying the functional evolution of the UVR8–BES1/BIM1 signaling module in green plants.

Both structure-guided and sequence-based phylogenetic analyses have distinct advantages and limitations in the detection of deep homology. For UVR8, structure-guided homology searches outperformed the sequence-based approach, as the extra domains and incomplete β-propeller folds of this protein often introduced ambiguity when its evolutionary relationships were assessed using sequence-based phylogeny (Figure 1A and Supplemental Figures 1, 3, and 4A). This observation is consistent with a recent report on viral evolution, in which the deep divergence of glycoproteins across the Flaviviridae was resolved using structural information (Mifsud et al., 2024). The BES1 and BIM1 TFs presented challenges due to their extensive IDRs and the inherently low confidence of protein structure predictions in non-model plants. Although the conserved DNA-binding domain of BES1 enabled partial reconstruction of its evolutionary relationships, structure-guided phylogenies provided limited resolution for deep homology of BES1 (Figure 1C and Supplemental Figures 3 and 6). The enrichment of IDRs in BIM1 orthologs limited the ability of structure-guided

phylogeny to resolve fine-grained clades (Figure 1B and Supplemental Figure 2). Indeed, algorithms such as FoldMason rely on local structural alignments of ordered regions, which inherently limits their ability to capture evolutionary signals within disordered domains (Gilchrist et al., 2026). Compared with phylogenies based exclusively on functional domains, those that incorporated full-length structural models showed substantially higher bootstrap support values, indicating that structural context improves phylogenetic robustness (Supplemental Figure 9). These results emphasize the necessity of integrating both structural and sequence information to achieve accurate phylogenetic inference (Puente-Lelievre et al., 2025). Our results demonstrated two different cases of structure-guided approaches for deep orthology searches, providing a scalable framework for future large-scale phylogenetic analyses of photoreceptors and TFs in plants.

Both *C. reinhardtii* and *M. endlicherianum* exhibit broad transcriptional and physiological responses to UV-B signals (Kunz et al., 2026; Tilbrook et al., 2016; Figure 3). Green algae also accumulate photoprotective compounds, including phenolics, mycosporine-like amino acids, and mucilage, which function as "sunscreens" to attenuate UV-B stress (Karsten and Holzinger, 2014; Holzinger et al., 2018; Zhang et al., 2020; Kunz et al., 2024). These findings indicate that the UVR8-mediated transcriptional regulation chassis is deeply rooted in the green lineage. Our protein interaction analyses demonstrated that the UVR8–BIM1 interaction was already established in green algae and has remained highly conserved throughout plant evolution (Figures 1 and 2). Thus, we propose that BIM1 served as one of the early core TFs (such as WRKY3 and HY5) in UV-B signaling. The BES1 ortholog emerged in the LCA of streptophyte algae, but it was unable to interact directly with UVR8 (Figures 1 and 2). In *M. polymorpha*, the ancestral UVR8–BIM1 module expanded by recruiting BES1 into the signaling complex through a physical interaction with BIM1. This expansion likely served as an adaptive strategy for terrestrial environments, ensuring a more integrated regulatory network to deal with intense UV-B radiation. During the course of land plant evolution, BES1 acquired a key regulatory motif, the 14-3-3 binding motif, in the LCA of lycophytes (Diao et al., 2023; Supplemental Figure 6). The 14-3-3 binding motif (RxxxSxP) of AtBES1, with a conserved serine (S) residue that serves as a BIN2 phosphorylation site, is critical for the AtUVR8–AtBES1 interaction and BR signal transduction (Gampala et al., 2007; Guo et al., 2024). Correspondingly, we found that the origin of direct UVR8–BES1 interaction coincided with the evolutionary node (lycophytes) at which the preliminary BR signaling pathway arose (Cheon et al., 2013; Ferreira-Guerra et al., 2020). Thus, we inferred that a complex BR-integrated UV-B signaling response evolved at least in the LCA of vascular plants, enhancing the complexity and responsiveness of their UV-B signaling.

The functional diversification of TFs has been a key driver for the expansion of UV-B signaling networks. In *M. polymorpha*, both MpBIM1 and MpBES1 bind to the canonical G-box motif (CACGTG), a *cis*-regulatory element recognized by bHLH TFs (Ezer et al., 2017). However, BRREs were not enriched in MpBES1 binding peaks (Figure 4A). This finding is consistent with the reported insensitivity of *M. polymorpha* to BR treatment and indicates that canonical BR signaling is not fully established in liverworts (Mecchia et al., 2021). In *A. thaliana*, AtBES1 has expanded its DNA-binding specificity and evolved the ability to bind the BRRE motif (CGTG$^{T}/_{C}$G), reflecting its dual role in BR and UV-B signaling pathways (Yu et al., 2011; Liang et al., 2018). Structural comparison between AtBES1 and bHLH TFs suggested that the versatility of AtBES1 is enabled by the unique conformation of the BES1-N domain, which permits recognition of both canonical and non-canonical elements (Nosaki et al., 2018). Cross-species transcriptome and DAP-seq comparisons indicated that MpBIM1 acts as the main UV-B-responsive TF in *M. polymorpha*, whereas MpBES1, as an auxiliary factor, functions by interacting with MpBIM1 (Figures 3 and 4). This interpretation was also supported by PPI predictions and experimental analyses, which showed that indirect connections between UVR8 and BES1 in bryophytes were established through BIM1 (Figure 2). MpBES1 has been shown to regulate cell division and differentiation in *Marchantia*, reflecting its ancient role in the regulation of plant growth (Mecchia et al., 2021). In angiosperms, BES1 has evolved from a BIM1-dependent co-regulatory component into a dominant regulator of the UV-B response (Liang et al., 2018). By acquiring UVR8-binding capacity and increasing its BR-responsive targets, BES1 gained the ability to coordinate growth and stress signaling in land plants (Liang et al., 2018, 2020). This transition likely involved the co-option of its ancestral growth-related roles into the UV-B signaling network, including the regulation of cell elongation, plant growth, and flavonoid biosynthesis.

Through multi-layered comparisons across species, we revealed that the UVR8–BES1/BIM1 module evolved through domain acquisition and transcriptional rewiring to meet the complex demands of terrestrial plants. The stepwise assembly of modular signaling components, coupled with a shift from shared to specialized regulatory roles, is a key mechanism underlying the adaptive evolution of UV-B-responsive regulatory networks in green plants (Figure 6). Our study provides a framework for understanding how light signaling networks evolved in green plants, shedding light on the adaptive strategies that confer plasticity and resilience in fluctuating terrestrial environments.

METHODS

Structure-guided ortholog identification and phylogenetic analyses

The genomes and gene annotations for 99 plant species, covering all major plant lineages, were obtained from public databases, including Phytozome, NCBI, CNCB (China National Center for Bioinformation), FernBase, and published sources (Supplemental Table 10). Sequence-based phylogenetic analyses of *UVR8*, *BES1*, and *BIM1* were performed following the pipeline described by Zhang et al. (2022a, 2022b). In brief, candidate sequences were identified using DIAMOND BLASTp (Buchfink et al., 2021) against *A. thaliana* proteins with an e-value cutoff of 1e−5 and validated using InterProScan v5.24 (Jones et al., 2014). Filtered protein sequences were aligned using MAFFT v7.490 (Katoh and Standley, 2013), trimmed with trimAl v1.3 (Capella-Gutierrez et al., 2009), and used to construct maximum-likelihood phylogenetic trees with IQ-TREE v2.4.0 (Minh et al., 2020). ModelFinder (Kalyaanamoorthy et al., 2017) was used to select the best-fit model, and 1000 ultrafast bootstrap replicates were performed.

For structure-guided phylogenetic analyses, all filtered protein sequences were subjected to protein structure prediction using AlphaFold2 (Bryant

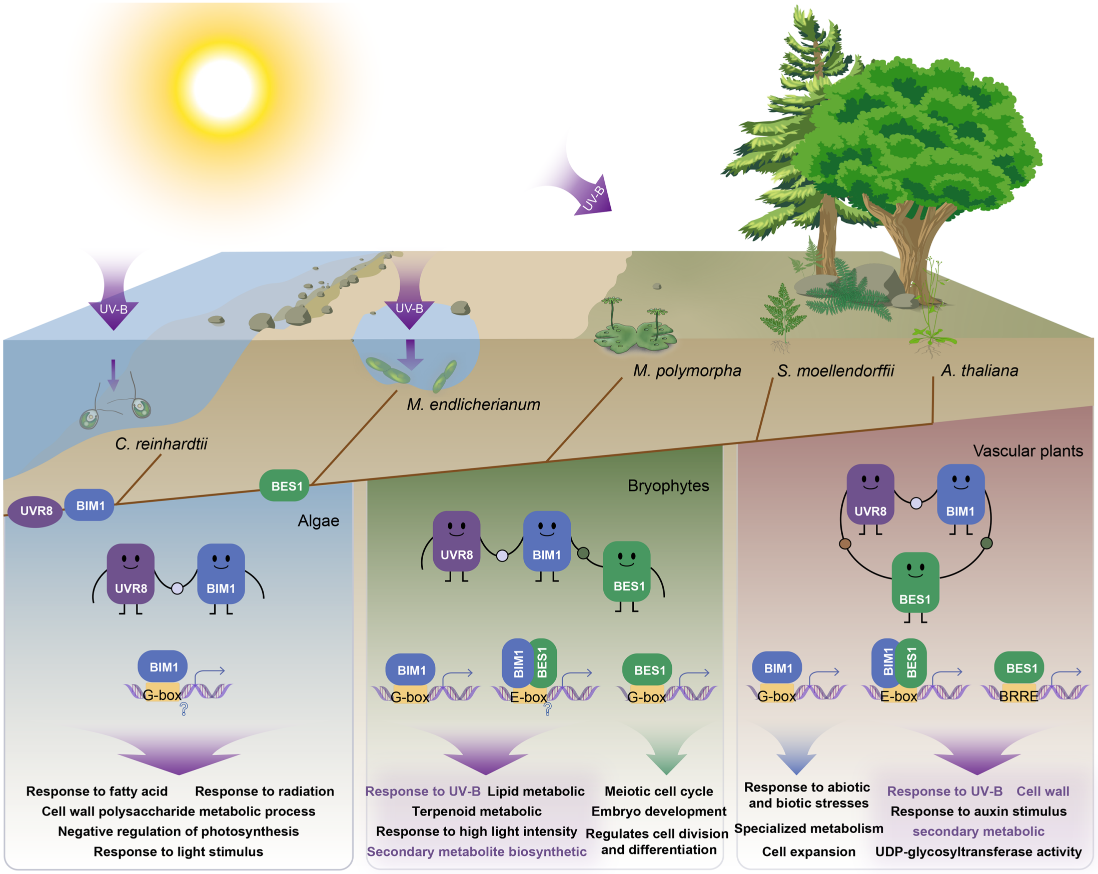

Figure 6. Proposed evolutionary trajectories of the UVR8–BES1/BIM1 signaling module in green plants.
The UV-B photoreceptor UVR8 and the transcription factor BIM1 both originated in the ancestor of green plants, whereas BES1 emerged later in streptophytes. The UVR8–BIM1 interaction emerged in green algae, forming an ancient UV-B signaling module. The BIM1–BES1 interaction emerged in liverworts, representing a significant step in the evolution of regulatory complexity. The UVR8–BES1 interaction emerged in lycophytes, enabling integration of UV-B and BR signaling. In green algae and liverworts, BIM1 is one of the core transcription factors in UV-B responses. After expansion and functional innovation of BES1, it became the dominant regulator in vascular plants. Unknown binding motifs are shown with question marks. The schematic is adapted from Martin and Allen (2018) and De Vries and Archibald (2018).

et al., 2022). For each protein, the model with the highest rank score was retained as the representative structure. Multiple structure alignments were generated using FoldMason v4-dd3c235 (Gilchrist et al., 2026; default parameters), which encodes structures as 3D interaction (3Di) + amino acid (aa) sequences and evaluates alignment quality using LDDT scores in a reference-free manner. The resulting structural alignments were mapped back onto the amino acid sequences, producing structure-informed multiple sequence alignments (MSAs) in FASTA format suitable for direct use in downstream phylogenetic analyses. Protein structures were visualized using PyMOL v3 (Schrödinger, LLC).

Protein-protein interaction (PPI) prediction and assays

The SpeedPPI pipeline, based on FoldDock and AlphaFold2, was used to predict the structures of the UVR8–BIM1, UVR8–BES1, and BIM1–BES1 dimers (Bryant et al., 2022; Bryant and Noé, 2023). All predictions were performed using all-vs.-all mode with default parameters. Predicted models with pLDDT scores below 70 were excluded, and the pDockQ scores of the remaining models were used to evaluate the likelihood of the PPIs.

LCI assays were performed to validate the PPIs of the UVR8–BIM1, UVR8–BES1, and BIM1–BES1 dimers, as described previously (Chen et al., 2008). In brief, full-length coding sequences (CDSs) of *UVR8*, *BES1*, and *BIM1* from *C. reinhardtii*, *M. endlicherianum*, *M. polymorpha*, and *S. moellendorffii* were cloned and inserted into the modified binary vectors pCAMBIA1300-NLuc and pCAMBIA1300-CLuc. *Agrobacterium tumefaciens* strain GV3101 harboring the NLuc- and -CLuc constructs was infiltrated into the abaxial side of 4-week-old *N. benthamiana* leaves. After 48 h, leaves were sprayed with 1 mM D-luciferin (40901ES01; Yeasen, Shanghai, China) containing 0.01% Triton X-100, and luminescence images were captured using a chemiluminescence imaging system (Tanon 4100). Luciferase activity was quantified using a dual-luciferase reporter assay kit (DL101; Vazyme, Nanjing, China) according to the manufacturer's instructions.

ERC analyses of the UVR8–BIM1 gene family

ERC was analyzed using the CovER pipeline in PhyKIT v1.11.12 (Steenwyk et al., 2021). For the UVR8–BIM1 pair, gene trees were pruned to retain shared taxa, and branch lengths were re-estimated using the fixed topology of the species tree. Pearson's correlation coefficient (R) between branch lengths was calculated, with $R > 0$ and an adjusted p value ($padj$) < 0.05 considered indicative of significant coevolution.

Ancestral sequence reconstruction of the BES1 gene family

The sequence-based BES1 phylogenetic tree and corresponding multiple sequence alignment were used as inputs for ancestral sequence

reconstruction, which was performed using CodeML in PAMLX under the empirical+F model (Xu and Yang, 2013).

Plant materials and growth conditions

Mesotaenium endlicherianum (SAG 12.97) was cultivated in SFM+NH4 medium prepared according to the protocol provided by the Central Collection of Algal Cultures (CCAC), University of Duisburg–Essen. Algae were maintained at 20°C under a 16-h (~650 μW cm^{-2})/8-h light/dark cycle in a growth chamber. *Marchantia polymorpha* L. accession Takaragaike-1 (Tak-1, male) was cultured on half-strength Gamborg's B5 medium with 1% (w/v) agar at 22°C under a 16-h (~1000 μW cm^{-2})/8-h light/dark cycle in an incubator (TCC-20, Wuhan Ruihua Appliance Limited Company, China) (Liu et al., 2024).

For UV-B acclimation, 20-day-old algal cells and 10-day-old gemmalings were exposed to narrowband UV-B (70 μW cm^{-2}; Philips TL20W/01RS) supplemented with white light at intensities of 650 μW cm^{-2} and 1000 μW cm^{-2}, respectively. For UV-B stress, cells were exposed to broadband UV-B (200 μW cm^{-2}; Philips TL20W/12RS) combined with 650 μW cm^{-2} white light (Tilbrook et al., 2016). Control samples were maintained under corresponding white-light conditions. All treatments were performed with three biological replicates.

RNA-seq and RT–qPCR analyses

Total RNA was extracted from *M. endlicherianum* and *M. polymorpha* samples collected 3 h after UV-B treatment (70 μW cm^{-2}) and from untreated controls. Three biological replicates were prepared for each treatment. RNA sequencing (RNA-seq) libraries were constructed and sequenced by Novogene Bioinformatics Technology Co., Ltd. (Beijing, China). Paired-end sequencing (150 bp) was performed using the Illumina NovaSeq 6000 platform. RNA-seq data analysis, including quality control, read alignment, quantification, normalization, and differential expression analysis, was performed as described previously (Diao et al., 2023). The reference genome assemblies and gene annotations for *M. endlicherianum* (v2) and *M. polymorpha* (v7.1) were used for read alignment and downstream analyses (Cheng et al., 2019; Dadras et al., 2023; Tanizawa et al., 2025). Significant DEGs were defined using a threshold of $|\log_2$ fold change (FC)$| \geq 1$ and p adj. ≤ 0.05. The RNA-seq data for *C. reinhardtii* and *A. thaliana* were obtained from published studies and reanalyzed in this work (Tilbrook et al., 2016; Tavridou et al., 2020). Orthologous genes across species were identified and clustered using OrthoFinder v2.5.4 (Emms and Kelly, 2019). UV-B-responsive orthologs were analyzed to assess the conservation of transcriptional responses across species. The degree of conservation was quantified using the "convergence degree," calculated as $|A \cap B|/\min(|A|, |B|)$, where $|A|$ and $|B|$ represent the numbers of DEGs in species A and B, respectively (Wu et al., 2022). Volcano plots and GO enrichment analyses were performed using custom R scripts and relevant packages.

To confirm the expression patterns of UV-B-responsive DEGs, total RNA extraction, cDNA synthesis, qPCR, and relative expression analysis were performed as described previously (Livak and Schmittgen, 2001). Gene-specific primers used for RT–qPCR are listed in Supplemental Table 11. *MpACT7* was used as the internal reference gene.

Analysis of gene regulatory networks

UV-B-responsive GRNs based on UV-B-responsive DEGs were reconstructed for *C. reinhardtii*, *M. endlicherianum*, *M. polymorpha*, and *A. thaliana*. High-confidence regulatory pairs were obtained from PlantRegMap (Tian et al., 2020), including both experimentally validated interactions and motif-based predictions. These pairs were filtered to ensure that both the TFs and their TGs were present in the UV-B DEG dataset. For motif-based predictions, the promoter region of each DEG, defined as the 1000-bp sequence upstream of its transcription start site, was scanned for TF binding motifs using FIMO (Find Individual Motif Occurrences) (Grant et al., 2011). Motif occurrences with p adj. < 0.05 were considered to indicate putative TF–target interactions and were incorporated into the regulatory network. All GRNs were visualized and analyzed using Gephi (Bastian et al., 2009).

DNA affinity purification sequencing (DAP-seq) analyses

DAP-seq assays were conducted by Gene Denovo Biotechnology Co., Ltd. (Guangzhou, China) following a modified protocol based on Bartlett et al. (2017). In brief, genomic DNA was extracted from *M. polymorpha* and *A. thaliana* and used to prepare sequencing libraries with the NEBNext DNA Library Prep Master Mix Set for Illumina (E6040S; NEB, Beijing, China). The CDSs of *MpBIM1*, *MpBES1*, and *AtBIM1* were cloned into expression vectors carrying a Halo-tag, and recombinant proteins were expressed *in vitro* using the TNT SP6 High-Yield Wheat Germ Protein Expression System (L3260; Promega, Beijing, China). After incubation with the DNA library, protein–DNA complexes were captured using magnetic beads, and nonspecific fragments were removed through multiple washing steps. The enriched DNA was purified and subjected to paired-end sequencing (150 bp) on the Illumina HiSeq 4000 platform. Sequencing reads were quality filtered and mapped to the *M. polymorpha* and *A. thaliana* reference genomes using Bowtie2 v2.2.5 (Langmead and Salzberg, 2012), allowing up to two mismatches. Peak calling was performed with MACS2 v2.2.7.1 (Feng et al., 2012), and peak-associated genes were annotated with ChIPseeker (Yu et al., 2015). Motif enrichment analysis was performed using MEME (Multiple Expectation maximizations for Motif Elicitation) and DREME (Discriminative Regular Expression Motif Elicitation) in the MEME Suite (Bailey et al., 2015; https://meme-suite.org/meme/). GO enrichment analysis was performed using custom R scripts and relevant packages.

Chromatin immunoprecipitation sequencing data for AtBES1 were obtained from Yu et al. Cross-species comparison of BIM1 and BES1 TG sets between *M. polymorpha* and *A. thaliana* was performed using OrthoVenn3 (Sun et al., 2023). KEGG pathway analysis was conducted primarily on the basis of the *Arabidopsis* dataset, and the enrichment network was generated using cirFunMap (Bu et al., 2021). Subsequently, genes from each *M. polymorpha* cluster were mapped to their *A. thaliana* orthologs using an R script, followed by visualization of the corresponding KEGG networks.

Dual-luciferase reporter (DLR) assay

To examine the regulatory activity of MpBIM1 and MpBES1 on their downstream target promoters, the CDSs of *MpBIM1* and *MpBES1* were cloned into the pCAMBIA1307-FLAG vector as effectors. Promoter regions (~1.5 kb upstream) of the TGs were inserted into the pGreenII 0800-LUC vector to generate reporter constructs. Effector and reporter plasmids were co-infiltrated into tobacco leaves using *A. tumefaciens*-mediated transient expression as described previously (Cao et al., 2024). Firefly and *Renilla* luciferase activities were measured using a DLR assay kit (DL101; Vazyme, Nanjing, China). Firefly luciferase activity was normalized to *Renilla* luciferase activity, and relative LUC/REN ratios were calculated. Each assay was performed with three biological replicates and was repeated independently at least twice.

DATA AND CODE AVAILABILITY

The raw RNA-seq and DAP-seq data have been deposited in the Genome Sequence Archive (Genomics, Proteomics, and Bioinformatics, 2021) at the National Genomics Data Center (Nucleic Acids Research, 2025) under BioProject no. PRJCA043849. The sequence alignment data and phylogenetic tree files have been deposited in Figshare (https://figshare.com/s/19af86adfe1c5a923c81).

FUNDING

This work was supported by the National Natural Science Foundation of China (W2511024 and 32370228) (B.Z.), the National Natural Science Foundation of China (32470232 and 32100178) (Z.Z.), the Natural Science Foundation of Jiangsu Province (BK20250004), the Collaborative

Innovation Center for Modern Crop Production co-sponsored by Province and Ministry (B.Z.), and the Priority Academic Program Development of Jiangsu Higher Education Institutions (B.Z.).

ACKNOWLEDGMENTS

We thank Hongtao Liu for kindly providing the *AtBES1-FLAG* plasmid. The authors declare no competing interests.

AUTHOR CONTRIBUTIONS

B.Z. and Z.Z. conceived and designed the study. C.C. performed all experiments and analyses unless otherwise specified, including structural modeling and phylogenetic analysis, most molecular experiments, and DAP-seq analyses. R.D. contributed to RNA-seq analyses. M.Z. and J. W. assisted with protein interaction assays. Q.J. performed the UV-B adaptation experiments in *M. endlicherianum*. Z.X. supported the AlphaFold2 modeling. W.X. and Y.Z. performed the UV-B adaptation experiments in *M. polymorpha*. Z.Z. and C.C. drafted the manuscript. B.Z. revised and edited the manuscript. All authors read and approved the final paper.

SUPPLEMENTAL INFORMATION

Supplemental information is available at *Plant Communications Online*.

Received: September 28, 2025
Revised: December 3, 2025
Accepted: April 1, 2026
Published: April 3, 2026

REFERENCES

Bailey, T.L., Johnson, J., Grant, C.E., and Noble, W.S. (2015). The MEME Suite. Nucleic Acids Res. **43**:W39–W49. https://doi.org/10.1093/nar/gkv416.

Bartlett, A., O'Malley, R.C., Huang, S.C., Galli, M., Nery, J.R., Gallavotti, A., Ecker, J.R., et al. (2017). Mapping genome-wide transcription-factor binding sites using DAP-seq. Nat. Protoc **12**:1659–1672. https://doi.org/10.1038/nprot.2017.055.

Bastian, M., Heymann, S., and Jacomy, M. (2009). Gephi: An Open Source Software for Exploring and Manipulating Networks. Proceedings of the International AAAI Conference on Web and Social Media **3**:361–362. https://doi.org/10.1609/icwsm.v3i1.13937.

Bowman, J.L.J.N.P. (2022). The origin of a land flora. Nat. Plants 8:1352–1369.

Bryant, P., and Noé, F. (2023). Rapid protein-protein interaction network creation from multiple sequence alignments with Deep Learning. Preprint at bioRxiv. https://doi.org/10.1101/2023.04.15.536993.

Bryant, P., Pozzati, G., and Elofsson, A. (2022). Improved prediction of protein-protein interactions using AlphaFold2. Nat. Commun. **13**:1265. https://doi.org/10.1038/s41467-022-28865-w.

Bu, D., Luo, H., Huo, P., Wang, Z., Zhang, S., He, Z., Wu, Y., Zhao, L., Liu, J., Guo, J., et al. (2021). KOBAS-i: intelligent prioritization and exploratory visualization of biological functions for gene enrichment analysis. Nucleic Acids Res. **49**:W317–W325. https://doi.org/10.1093/nar/gkab447.

Buchfink, B., Reuter, K., and Drost, H.G. (2021). Sensitive protein alignments at tree-of-life scale using DIAMOND. Nat. Methods **18**:366–368. https://doi.org/10.1038/s41592-021-01101-x.

Cao, C., Guo, S., Deng, P., Yang, S., Xu, J., Hu, T., Hu, Z., Chen, D., Zhang, H., Navea, I.P., et al. (2024). The BEL1-like homeodomain protein OsBLH4 regulates rice plant height, grain number, and heading date by repressing the expression of OsGA2ox1. Plant J. **119**:1369–1385. https://doi.org/10.1111/tpj.16857.

Capella-Gutiérrez, S., Silla-Martínez, J.M., and Gabaldón, T. (2009). trimAl: a tool for automated alignment trimming in large-scale phylogenetic analyses. Bioinformatics **25**:1972–1973. https://doi.org/10.1093/bioinformatics/btp348.

Chen, H., Zou, Y., Shang, Y., Lin, H., Wang, Y., Cai, R., Tang, X., and Zhou, J.M. (2008). Firefly luciferase complementation imaging assay for protein-protein interactions in plants. Plant Physiol. **146**:368–376. https://doi.org/10.1104/pp.107.111740.

Cheng, S., Xian, W., Fu, Y., Marin, B., Keller, J., Wu, T., Sun, W., Li, X., Xu, Y., Zhang, Y., et al. (2019). Genomes of Subaerial Zygnematophyceae Provide Insights into Land Plant Evolution. Cell **179**:1057–1067.e14. https://doi.org/10.1016/j.cell.2019.10.019.

Cheon, J., Fujioka, S., Dilkes, B.P., and Choe, S. (2013). Brassinosteroids regulate plant growth through distinct signaling pathways in Selaginella and Arabidopsis. PLoS One **8**:e81938. https://doi.org/10.1371/journal.pone.0081938.

Dadras, A., Fürst-Jansen, J.M.R., Darienko, T., Krone, D., Scholz, P., Sun, S., Herrfurth, C., Rieseberg, T.P., Irisarri, I., Steinkamp, R., et al. (2023). Environmental gradients reveal stress hubs pre-dating plant terrestrialization. Nat. Plants **9**:1419–1438. https://doi.org/10.1038/s41477-023-01491-0.

Davies, K.M., Andre, C.M., Kulshrestha, S., Zhou, Y., Schwinn, K.E., Albert, N.W., Chagné, D., van Klink, J.W., Landi, M., and Bowman, J.L. (2024). The evolution of flavonoid biosynthesis. Philos. Trans. R. Soc. Lond. B Biol. Sci. **379**:20230361. https://doi.org/10.1098/rstb.2023.0361.

de Vries, J., and Archibald, J.M. (2018). Plant evolution: landmarks on the path to terrestrial life. New Phytol. **217**:1428–1434. https://doi.org/10.1111/nph.14975.

de Vries, S., Fürst-Jansen, J.M.R., Irisarri, I., Dhabalia Ashok, A., Ischebeck, T., Feussner, K., Abreu, I.N., Petersen, M., Feussner, I., and de Vries, J. (2021). The evolution of the phenylpropanoid pathway entailed pronounced radiations and divergences of enzyme families. Plant J. **107**:975–1002. https://doi.org/10.1111/tpj.15387.

Delaux, P.M., Hetherington, A.J., Coudert, Y., Delwiche, C., Dunand, C., Gould, S., Kenrick, P., Li, F.W., Philippe, H., Rensing, S.A., et al. (2019). Reconstructing trait evolution in plant evo-devo studies. Curr. Biol. **29**:R1110–R1118. https://doi.org/10.1016/j.cub.2019.09.044.

Diao, R., Zhao, M., Liu, Y., Zhang, Z., and Zhong, B. (2023). The advantages of crosstalk during the evolution of the BZR1-ARF6-PIF4 (BAP) module. J. Integr. Plant Biol. **65**:2631–2644. https://doi.org/10.1111/jipb.13554.

Emms, D.M., and Kelly, S. (2019). OrthoFinder: phylogenetic orthology inference for comparative genomics. Genome Biol. **20**:238. https://doi.org/10.1186/s13059-019-1832-y.

Ezer, D., Shepherd, S.J.K., Brestovitsky, A., Dickinson, P., Cortijo, S., Charoensawan, V., Box, M.S., Biswas, S., Jaeger, K.E., and Wigge, P.A. (2017). The G-Box Transcriptional Regulatory Code in Arabidopsis. Plant Physiol. **175**:628–640. https://doi.org/10.1104/pp.17.01086.

Feng, J., Liu, T., Qin, B., Zhang, Y., and Liu, X.S. (2012). Identifying ChIP-seq enrichment using MACS. Nat. Protoc. **7**:1728–1740. https://doi.org/10.1038/nprot.2012.101.

Fernández, M.B., Tossi, V., Lamattina, L., and Cassia, R. (2016). A Comprehensive Phylogeny Reveals Functional Conservation of the UV-B Photoreceptor UVR8 from Green Algae to Higher Plants. Front. Plant Sci. **7**:1698. https://doi.org/10.3389/fpls.2016.01698.

Ferreira-Guerra, M., Marquès-Bueno, M., Mora-García, S., and Caño-Delgado, A.I. (2020). Delving into the evolutionary origin of steroid sensing in plants. Curr. Opin. Plant Biol. **57**:87–95. https://doi.org/10.1016/j.pbi.2020.06.005.

Fürst-Jansen, J.M.R., de Vries, S., and de Vries, J. (2020). Evo-physio: on stress responses and the earliest land plants. J. Exp. Bot. **71**:3254–3269. https://doi.org/10.1093/jxb/eraa007.

Furuya, T., Saegusa, N., Yamaoka, S., Tomoita, Y., Minamino, N., Niwa, M., Inoue, K., Yamamoto, C., Motomura, K., Shimadzu, S., et al. (2024). A non-canonical BZR/BES transcription factor regulates the development of haploid reproductive organs in Marchantia polymorpha. Nat. Plants **10**:785–797. https://doi.org/10.1038/s41477-024-01669-0.

Gampala, S.S., Kim, T.W., He, J.X., Tang, W., Deng, Z., Bai, M.Y., Guan, S., Lalonde, S., Sun, Y., Gendron, J.M., et al. (2007). An essential role for 14-3-3 proteins in brassinosteroid signal transduction in Arabidopsis. Dev. Cell **13**:177–189. https://doi.org/10.1016/j.devcel.2007.06.009.

Gilchrist, C.L.M., Mirdita, M., and Steinegger, M. (2026). Multiple protein structure alignment at scale with FoldMason. Science **391**:485–488. https://doi.org/10.1126/science.ads6733.

Goldbecker, E.S., and de Vries, J. (2025). Systems Biology of Streptophyte Cell Evolution. Annu. Rev. Plant Biol. **76**:493–522. https://doi.org/10.1146/annurev-arplant-083123-060254.

Grant, C.E., Bailey, T.L., and Noble, W.S. (2011). FIMO: scanning for occurrences of a given motif. Bioinformatics **27**:1017–1018. https://doi.org/10.1093/bioinformatics/btr064.

Guo, B., Kim, E.J., Zhu, Y., Wang, K., and Russinova, E. (2024). Shaping Brassinosteroid Signaling through Scaffold Proteins. Plant Cell Physiol. **65**:1608–1617. https://doi.org/10.1093/pcp/pcae040.

Han, X., Chang, X., Zhang, Z., Chen, H., He, H., Zhong, B., and Deng, X. W. (2019). Origin and Evolution of Core Components Responsible for Monitoring Light Environment Changes during Plant Terrestrialization. Mol. Plant **12**:847–862. https://doi.org/10.1016/j.molp.2019.04.006.

Hayes, S., Velanis, C.N., Jenkins, G.I., and Franklin, K.A. (2014). UV-B detected by the UVR8 photoreceptor antagonizes auxin signaling and plant shade avoidance. Proc. Natl. Acad. Sci. USA **111**:11894–11899. https://doi.org/10.1073/pnas.1403052111.

Hess, S., Williams, S.K., Busch, A., Irisarri, I., Delwiche, C.F., de Vries, S., Darienko, T., Roger, A.J., Archibald, J.M., Buschmann, H., et al. (2022). A phylogenomically informed five-order system for the closest relatives of land plants. Curr. Biol. **32**:4473–4482.e7. https://doi.org/10.1016/j.cub.2022.08.022.

Holzinger, A., Albert, A., Aigner, S., Uhl, J., Schmitt-Kopplin, P., Trumhová, K., and Pichrtová, M. (2018). Arctic, Antarctic, and temperate green algae Zygnema spp. under UV-B stress: vegetative cells perform better than pre-akinetes. Protoplasma **255**:1239–1252. https://doi.org/10.1007/s00709-018-1225-1.

Jones, P., Binns, D., Chang, H.Y., Fraser, M., Li, W., McAnulla, C., McWilliam, H., Maslen, J., Mitchell, A., Nuka, G., et al. (2014). InterProScan 5: genome-scale protein function classification. Bioinformatics **30**:1236–1240. https://doi.org/10.1093/bioinformatics/btu031.

Kalyaanamoorthy, S., Minh, B.Q., Wong, T.K.F., von Haeseler, A., and Jermiin, L.S. (2017). ModelFinder: fast model selection for accurate phylogenetic estimates. Nat. Methods **14**:587–589. https://doi.org/10.1038/nmeth.4285.

Karsten, U., and Holzinger, A. (2014). Green algae in alpine biological soil crust communities: acclimation strategies against ultraviolet radiation and dehydration. Biodivers. Conserv. **23**:1845–1858. https://doi.org/10.1007/s10531-014-0653-2.

Katoh, K., and Standley, D.M. (2013). MAFFT multiple sequence alignment software version 7: improvements in performance and usability. Mol. Biol. Evol. **30**:772–780. https://doi.org/10.1093/molbev/mst010.

Kim, D., Park, S., and Steinegger, M. (2025). Unicore Enables Scalable and Accurate Phylogenetic Reconstruction with Structural Core Genes. Genome Biol. Evol. **17**. evaf109–gbe/evaf109.

Kunz, C.F., de Vries, S., and de Vries, J. (2024). Plant terrestrialization: an environmental pull on the evolution of multi-sourced streptophyte phenolics. Philos. Trans. R. Soc. Lond. B Biol. Sci. **379**:20230358. https://doi.org/10.1098/rstb.2023.0358.

Kunz, C.F., Abreu, I.N., Darienko, T., Fürst-Jansen, J., Feussner, K., Feussner, I., Lorenz, M., and de Vries, J. (2026). Chemodiverse cell systems responses to UV in an algal sister of land plants. bioRxiv. https://doi.org/10.64898/2026.02.10.704744.

Langmead, B., and Salzberg, S.L. (2012). Fast gapped-read alignment with Bowtie 2. Nat. Methods **9**:357–359. https://doi.org/10.1038/nmeth.1923.

Liang, T., Yang, Y., and Liu, H. (2019). Signal transduction mediated by the plant UV-B photoreceptor UVR8. New Phytol. **221**:1247–1252. https://doi.org/10.1111/nph.15469.

Liang, T., Mei, S., Shi, C., Yang, Y., Peng, Y., Ma, L., Wang, F., Li, X., Huang, X., Yin, Y., and Liu, H. (2018). UVR8 Interacts with BES1 and BIM1 to Regulate Transcription and Photomorphogenesis in Arabidopsis. Dev. Cell **44**:512–523.e5. https://doi.org/10.1016/j.devcel.2017.12.028.

Liang, T., Shi, C., Peng, Y., Tan, H., Xin, P., Yang, Y., Wang, F., Li, X., Chu, J., Huang, J., et al. (2020). Brassinosteroid-Activated BRI1-EMS-SUPPRESSOR 1 Inhibits Flavonoid Biosynthesis and Coordinates Growth and UV-B Stress Responses in Plants. Plant Cell **32**:3224–3239. https://doi.org/10.1105/tpc.20.00048.

Liu, W., Yang, Z., Cai, G., Li, B., Liu, S., Willemsen, V., and Xu, L. (2024). MpANT regulates meristem development in Marchantia polymorpha. Cell Rep. **43**:114466. https://doi.org/10.1016/j.celrep.2024.114466.

Livak, K.J., and Schmittgen, T.D. (2001). Analysis of relative gene expression data using real-time quantitative PCR and the 2(-Delta Delta C(T)) Method. Methods **25**:402–408. https://doi.org/10.1006/meth.2001.1262.

Maberly, S.C. (2014). The fitness of the environments of air and water for photosynthesis, growth, reproduction and dispersal of photoautotrophs: An evolutionary and biogeochemical perspective. Aquat. Bot. **118**:4–13. https://doi.org/10.1016/j.aquabot.2014.06.014.

Martin, W.F., and Allen, J.F. (2018). An Algal Greening of Land. Cell **174**:256–258.

Mecchia, M.A., García-Hourquet, M., Lozano-Elena, F., Planas-Riverola, A., Blasco-Escamez, D., Marquès-Bueno, M., Mora-García, S., and Caño-Delgado, A.I. (2021). The BES1/BZR1-family transcription factor MpBES1 regulates cell division and differentiation in Marchantia polymorpha. Curr. Biol. **31**:4860–4869.e8. https://doi.org/10.1016/j.cub.2021.08.050.

Mifsud, J.C.O., Lytras, S., Oliver, M.R., Toon, K., Costa, V.A., Holmes, E.C., and Grove, J. (2024). Mapping glycoprotein structure reveals Flaviviridae evolutionary history. Nature **633**:695–703. https://doi.org/10.1038/s41586-024-07899-8.

Minh, B.Q., Schmidt, H.A., Chernomor, O., Schrempf, D., Woodhams, M.D., von Haeseler, A., and Lanfear, R. (2020). IQ-TREE 2: New Models and Efficient Methods for Phylogenetic Inference in the Genomic Era. Mol. Biol. Evol. **37**:1530–1534. https://doi.org/10.1093/molbev/msaa015.

Morris, J.L., Puttick, M.N., Clark, J.W., Edwards, D., Kenrick, P., Pressel, S., Wellman, C.H., Yang, Z., Schneider, H., and Donoghue, P.C.J. (2018). The timescale of early land plant evolution. Proc. Natl. Acad. Sci. USA **115**:E2274–E2283. https://doi.org/10.1073/pnas.1719588115.

Nosaki, S., Miyakawa, T., Xu, Y., Nakamura, A., Hirabayashi, K., Asami, T., Nakano, T., and Tanokura, M. (2018). Structural basis

for brassinosteroid response by BIL1/BZR1. Nat. Plants **4**:771–776. https://doi.org/10.1038/s41477-018-0255-1.

Nosaki, S., Mitsuda, N., Sakamoto, S., Kusubayashi, K., Yamagami, A., Xu, Y., Bui, T.B.C., Terada, T., Miura, K., Nakano, T., et al. (2022). Brassinosteroid-induced gene repression requires specific and tight promoter binding of BIL1/BZR1 via DNA shape readout. Nat. Plants **8**:1440–1452. https://doi.org/10.1038/s41477-022-01289-6.

O'Hara, A., and Jenkins, G.I. (2012). In vivo function of tryptophans in the Arabidopsis UV-B photoreceptor UVR8. Plant Cell **24**:3755–3766. https://doi.org/10.1105/tpc.112.101451.

Oh, E., Zhu, J.Y., and Wang, Z.Y. (2012). Interaction between BZR1 and PIF4 integrates brassinosteroid and environmental responses. Nat. Cell Biol. **14**:802–809. https://doi.org/10.1038/ncb2545.

Oravecz, A., Baumann, A., Máté, Z., Brzezinska, A., Molinier, J., Oakeley, E.J., Adám, E., Schäfer, E., Nagy, F., and Ulm, R. (2006). CONSTITUTIVELY PHOTOMORPHOGENIC1 is required for the UV-B response in Arabidopsis. Plant Cell **18**:1975–1990. https://doi.org/10.1105/tpc.105.040097.

Podolec, R., Demarsy, E., and Ulm, R. (2021). Perception and Signaling of Ultraviolet-B Radiation in Plants. Annu. Rev. Plant Biol. **72**:793–822. https://doi.org/10.1146/annurev-arplant-050718-095946.

Puente-Lelievre, C., Malik, A., and Douglas, J. (2025). Protein Structural Phylogenetics. Genome Biol. Evol. **17**:evaf139.

Reyero-Saavedra, M.d.R., Sánchez Correa, M.d.S., Estrella Parra, E.A., Espinosa González, A.M., Nolasco Ontiveros, E., Ortiz-Montiel, J.G., Campos Contreras, J.E., López Urrutia, E., Avila Acevedo, J.G., Benítez Flores, J.d.C., et al. (2023). Ultraviolet Radiation and Its Effects on Plants. In Abiotic Stress in Plants - Adaptations to Climate Change, M.T. Oliveira and A.A.A. Fernandes Silva, eds. (IntechOpen: London). https://doi.org/10.5772/intechopen.109474.

Rieseberg, T.P., Dadras, A., Darienko, T., Post, S., Herrfurth, C., Fürst-Jansen, J.M.R., Hohnhorst, N., Petroll, R., Rensing, S.A., Pröschold, T., et al. (2025). Time-resolved oxidative signal convergence across the algae-embryophyte divide. Nat. Commun. **16**:1780. https://doi.org/10.1038/s41467-025-56939-y.

Rizzini, L., Favory, J.-J., Cloix, C., Faggionato, D., O'Hara, A., Kaiserli, E., Baumeister, R., Schäfer, E., Nagy, F., Jenkins, G.I., and Ulm, R. (2011). Perception of UV-B by the Arabidopsis UVR8 protein. Science **332**:103–106. https://doi.org/10.1126/science.1200660.

Rozema, J., Björn, L.O., Bornman, J.F., Gaberščik, A., Häder, D.P., Trošt, T., Germ, M., Klisch, M., Gröniger, A., Sinha, R.P., et al. (2002). The role of UV-B radiation in aquatic and terrestrial ecosystems—an experimental and functional analysis of the evolution of UV-absorbing compounds. J. Photochem. Photobiol., B **66**:2–12. https://doi.org/10.1016/S1011-1344(01)00269-X.

Shi, C., and Liu, H. (2021). How plants protect themselves from ultraviolet-B radiation stress. Plant Physiol. **187**:1096–1103. https://doi.org/10.1093/plphys/kiab245.

Steenwyk, J.L., Buida, T.J., Labella, A.L., Li, Y., Shen, X.X., and Rokas, A. (2021). PhyKIT: a broadly applicable UNIX shell toolkit for processing and analyzing phylogenomic data. Bioinformatics **37**:2325–2331. https://doi.org/10.1093/bioinformatics/btab096.

Sun, J., Lu, F., Luo, Y., Bie, L., Xu, L., and Wang, Y. (2023). OrthoVenn3: an integrated platform for exploring and visualizing orthologous data across genomes. Nucleic Acids Res. **51**:W397–W403. https://doi.org/10.1093/nar/gkad313.

Tanaka, Y., Sasaki, N., and Ohmiya, A. (2008). Biosynthesis of plant pigments: anthocyanins, betalains and carotenoids. Plant J. **54**:733–749. https://doi.org/10.1111/j.1365-313X.2008.03447.x.

Tanizawa, Y., Mochizuki, T., Yagura, M., Sakamoto, M., Fujisawa, T., Kawamura, S., Shimokawa, E., Yamaoka, S., Nishihama, R., Bowman, J.L., et al. (2025). MarpolBase: Genome database for Marchantia polymorpha featuring high quality reference genome sequences. Plant Cell Physiol. pcaf159. https://doi.org/10.1093/pcp/pcaf159.

Tavridou, E., Pireyre, M., and Ulm, R. (2020). Degradation of the transcription factors PIF4 and PIF5 under UV-B promotes UVR8-mediated inhibition of hypocotyl growth in Arabidopsis. Plant J. **101**:507–517. https://doi.org/10.1111/tpj.14556.

Tian, F., Yang, D.C., Meng, Y.Q., Jin, J., and Gao, G. (2020). PlantRegMap: charting functional regulatory maps in plants. Nucleic Acids Res. **48**:D1104–D1113. https://doi.org/10.1093/nar/gkz1020.

Tilbrook, K., Dubois, M., Crocco, C.D., Yin, R., Chappuis, R., Allorent, G., Schmid-Siegert, E., Goldschmidt-Clermont, M., and Ulm, R. (2016). UV-B Perception and Acclimation in Chlamydomonas reinhardtii. Plant Cell **28**:966–983. https://doi.org/10.1105/tpc.15.00287.

Toledo-Ortiz, G., Huq, E., and Quail, P.H. (2003). The Arabidopsis basic/helix-loop-helix transcription factor family. Plant Cell **15**:1749–1770. https://doi.org/10.1105/tpc.013839.

Wu, T.Y., Hoh, K.L., Boonyaves, K., Krishnamoorthi, S., and Urano, D. (2022). Diversification of heat shock transcription factors expanded thermal stress responses during early plant evolution. Plant Cell **34**:3557–3576. https://doi.org/10.1093/plcell/koac204.

Xu, B., and Yang, Z. (2013). PAMLX: a graphical user interface for PAML. Mol. Biol. Evol. **30**:2723–2724. https://doi.org/10.1093/molbev/mst179.

Yang, Y., Liang, T., Zhang, L., Shao, K., Gu, X., Shang, R., Shi, N., Li, X., Zhang, P., and Liu, H. (2018). UVR8 interacts with WRKY36 to regulate HY5 transcription and hypocotyl elongation in Arabidopsis. Nat. Plants **4**:98–107. https://doi.org/10.1038/s41477-017-0099-0.

Yin, Y., Vafeados, D., Tao, Y., Yoshida, S., Asami, T., and Chory, J. (2005). A new class of transcription factors mediates brassinosteroid-regulated gene expression in Arabidopsis. Cell **120**:249–259. https://doi.org/10.1016/j.cell.2004.11.044.

Yu, G., Wang, L.G., and He, Q.Y. (2015). ChIPseeker: an R/Bioconductor package for ChIP peak annotation, comparison and visualization. Bioinformatics **31**:2382–2383. https://doi.org/10.1093/bioinformatics/btv145.

Yu, X., Li, L., Zola, J., Aluru, M., Ye, H., Foudree, A., Guo, H., Anderson, S., Aluru, S., Liu, P., et al. (2011). A brassinosteroid transcriptional network revealed by genome-wide identification of BESI target genes in Arabidopsis thaliana. Plant J. **65**:634–646. https://doi.org/10.1111/j.1365-313X.2010.04449.x.

Zeng, H.Y., Deng, S., Jin, C., Shang, Z., Chang, L., Wang, J., Han, X., Wang, A., Jin, D., Wang, Y., et al. (2024). Origin and evolution of auxin-mediated acid growth. Proc. Natl. Acad. Sci. USA **121**:e2412493121. https://doi.org/10.1073/pnas.2412493121.

Zhang, Z., Xu, C., Zhang, S., Shi, C., Cheng, H., Liu, H., and Zhong, B. (2022a). Origin and adaptive evolution of UV RESISTANCE LOCUS 8-mediated signaling during plant terrestrialization. Plant Physiol. **188**:332–346. https://doi.org/10.1093/plphys/kiab486.

Zhang, Z., Ma, X., Liu, Y., Yang, L., Shi, X., Wang, H., Diao, R., and Zhong, B. (2022b). Origin and evolution of green plants in the light of key evolutionary events. J. Integr. Plant Biol. **64**:516–535. https://doi.org/10.1111/jipb.13224.

Zhang, Z.H., Chang, X., Su, D.Y., Yao, R., Liu, X.D., Zhu, H., Liu, G.X., and Zhong, B.J. (2020). Comprehensive transcriptome analyses of twoOocystisalgae provide insights into the adaptation to Qinghai–Tibet Plateau. J. Syst. Evol. **59**:1209–1219. https://doi.org/10.1111/jse.12589.

Plant Communications, Volume *7*

Supplemental information

Functional evolution and rewiring of the UVR8–BES1/BIM1 module underpin the refinement of UV-B responses during plant terrestrialization

Chengjuan Cao, Runjie Diao, Mengru Zhao, Qiuting Ji, Jingwen Wang, Zilong Xu, Wenhui Xie, Yujun Zhou, Zhenhua Zhang, and Bojian Zhong

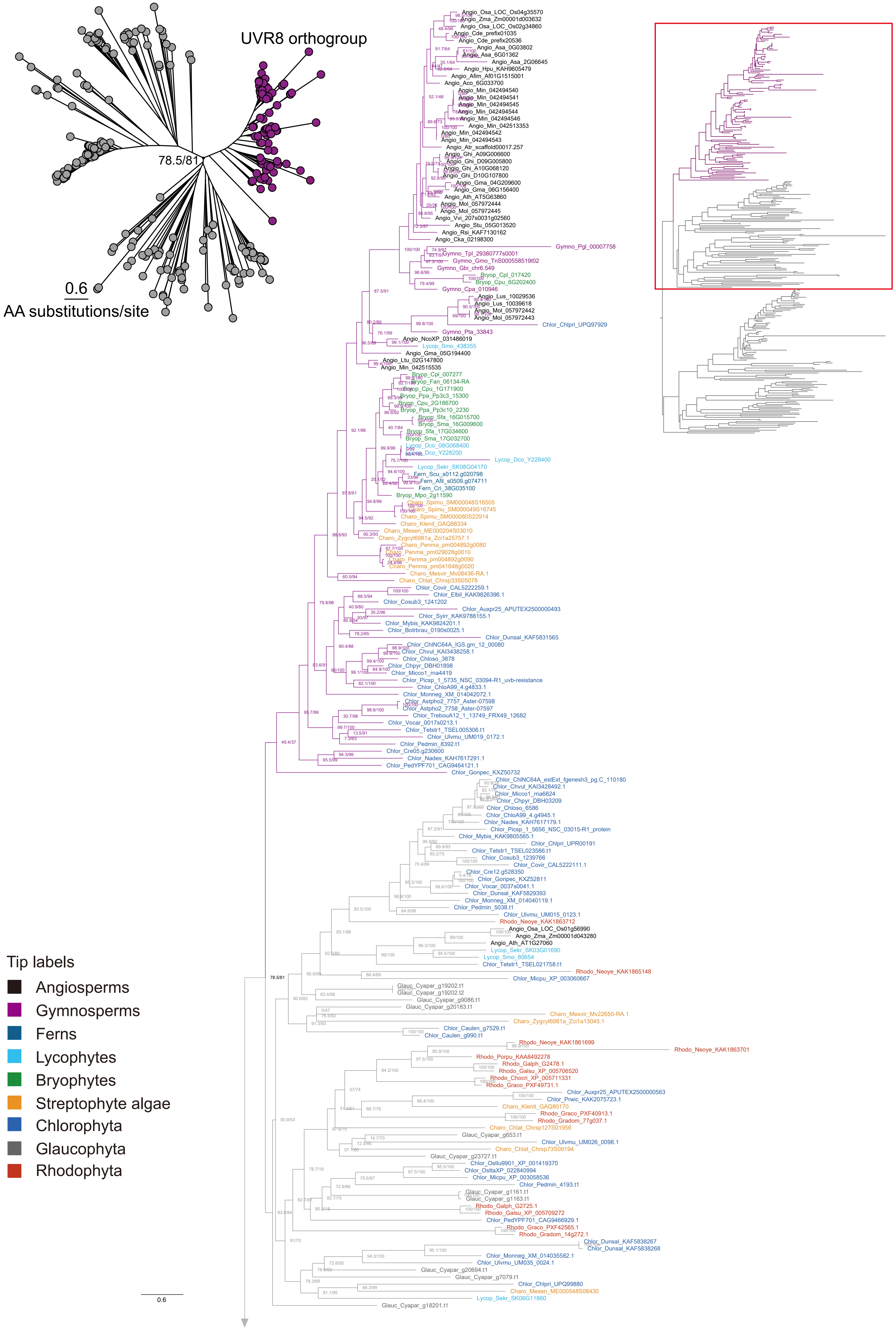
UVR8 orthogroup
78.5/81
0.6
AA substitutions/site
Angio_Osa_LOC_Os04g35570
Angio_Zma_Zm00001d003632
Angio_Osa_LOC_Os02g34860
Angio_Cde_prefix01035
Angio_Cde_prefix20536
Angio_Asa_0G03802
Angio_Asa_6G01362
Angio_Asa_2G06645
Angio_Hpu_KAH9605479
Angio_Afim_Af01G1515001
Angio_Aco_6G033700
Angio_Min_042494540
Angio_Min_042494541
Angio_Min_042494545
Angio_Min_042494544
Angio_Min_042494546
Angio_Min_042513353
Angio_Min_042494542
Angio_Min_042494543
Angio_Atr_scaffold00017.257
Angio_Ghi_A09G006600
Angio_Ghi_D09G005800
Angio_Ghi_A10G068120
Angio_Ghi_D10G107800
Angio_Gma_04G209600
Angio_Gma_06G156400
Angio_Ath_AT5G63860
Angio_Mol_057972444
Angio_Mol_057972445
Angio_Vvi_207s0031g02560
Angio_Stu_05G013520
Angio_Rsi_KAF7130162
Angio_Cka_02198300
Gymno_Pgl_00007758
Gymno_Tpl_29380777s0001
Gymno_Gmo_TnS000558519t02
Gymno_Gbi_chr6.549
Bryop_Cpl_017420
Bryop_Cpu_6G202400
Gymno_Cpa_010946
Angio_Lus_10029536
Angio_Lus_10039618
Angio_Mol_057972442
Angio_Mol_057972443
Chlor_Chlpri_UPQ97929
Gymno_Pta_33843
Angio_NcoXP_031486019
Lycop_Smo_438355
Angio_Gma_05G194400
Angio_Ltu_02G147800
Angio_Min_042515535
Bryop_Cpl_007277
Bryop_Fan_06134-RA
Bryop_Cpu_1G171900
Bryop_Ppa_Pp3c3_15300
Bryop_Cpu_2G186700
Bryop_Ppa_Pp3c10_2230
Bryop_Sfa_16G015700
Bryop_Sma_16G009600
Bryop_Sfa_17G034600
Bryop_Sma_17G032700
Lycop_Dco_08G068400
Lycop_Dco_Y228200
Lycop_Dco_Y228400
Lycop_Sekr_SK08G04170
Fern_Scu_s0112.g020798
Fern_Afil_s0509.g074711
Fern_Cri_38G035100
Bryop_Mpo_2g11590
Charo_Spimu_SM000048S16505
Charo_Spimu_SM000049S16745
Charo_Spimu_SM000080S22914
Charo_Klenit_GAQ88334
Charo_Mesen_ME000204S03010
Charo_Zygcyl6981a_Zci1a25757.1
Charo_Penma_pm004892g0080
Charo_Penma_pm029028g0010
Charo_Penma_pm004892g0090
Charo_Penma_pm041648g0020
Charo_Mesvir_Mv08436-RA.1
Charo_Chlat_Chrsp33S05078
Chlor_Covir_CAL5222259.1
Chlor_Elbil_KAK9826396.1
Chlor_Cosub3_1241202
Chlor_Auxpr25_APUTEX2500000493
Chlor_Syirr_KAK9788155.1
Chlor_Mybis_KAK9824201.1
Chlor_Botrbrau_0190s0025.1
Chlor_Dunsal_KAF5831565
Chlor_ChlNC64A_IGS.gm_12_00080
Chlor_Chvul_KAI3438258.1
Chlor_Chloso_3878
Chlor_Chpyr_DBH01898
Chlor_Micco1_rna4419
Chlor_Picsp_1_5735_NSC_03094-R1_uvb-resistance
Chlor_ChloA99_4.g4833.1
Chlor_Monneg_XM_014042072.1
Chlor_Astpho2_7757_Aster-07598
Chlor_Astpho2_7758_Aster-07597
Chlor_TrebouA12_1_13749_FRX49_12682
Chlor_Vocar_0017s0213.1
Chlor_Tetstr1_TSEL005306.t1
Chlor_Ulvmu_UM019_0172.1
Chlor_Pedmin_8392.t1
Chlor_Cre05.g230600
Chlor_Nades_KAH7617291.1
Chlor_PedYPF701_CAG9464121.1
Chlor_Gonpec_KXZ50732
Chlor_ChlNC64A_estExt_fgenesh3_pg.C_110180
Chlor_Chvul_KAI3428492.1
Chlor_Micco1_rna6624
Chlor_Chpyr_DBH03209
Chlor_Chloso_6586
Chlor_ChloA99_4.g4945.1
Chlor_Nades_KAH7617179.1
Chlor_Picsp_1_5656_NSC_03015-R1_protein
Chlor_Mybis_KAK9805565.1
Chlor_Chlpri_UPR00191
Chlor_Tetstr1_TSEL023586.t1
Chlor_Cosub3_1239766
Chlor_Covir_CAL5222111.1
Chlor_Cre12.g528350
Chlor_Gonpec_KXZ52811
Chlor_Vocar_0037s0041.1
Chlor_Dunsal_KAF5829393
Chlor_Monneg_XM_014040119.1
Chlor_Pedmin_5038.t1
Chlor_Ulvmu_UM015_0123.1
Rhodo_Neoye_KAK1863712
Angio_Osa_LOC_Os01g56990
Angio_Zma_Zm00001d043280
Angio_Ath_AT1G27060
Lycop_Sekr_SK03G01690
Lycop_Smo_80654
Chlor_Tetstr1_TSEL021758.t1
Rhodo_Neoye_KAK1865148
Chlor_Micpu_XP_003060667
Glauc_Cyapar_g19202.t1
Glauc_Cyapar_g19202.t2
Glauc_Cyapar_g9086.t1
Glauc_Cyapar_g20183.t1
Charo_Mesvir_Mv22650-RA.1
Charo_Zygcyl6981a_Zci1a13045.1
Chlor_Caulen_g7529.t1
Chlor_Caulen_g990.t1
Rhodo_Neoye_KAK1861699
Rhodo_Neoye_KAK1863701
Rhodo_Porpu_KAA8492278
Rhodo_Galph_G2478.1
Rhodo_Galsu_XP_005706520
Rhodo_Chocri_XP_005711331
Rhodo_Graco_PXF49731.1
Chlor_Auxpr25_APUTEX2500000563
Chlor_Prwic_KAK2075723.1
Charo_Klenit_GAQ80170
Rhodo_Graco_PXF40913.1
Rhodo_Gradom_77g037.1
Charo_Chlat_Chrsp127S01956
Glauc_Cyapar_g653.t1
Chlor_Ulvmu_UM026_0098.1
Charo_Chlat_Chrsp73S09194
Glauc_Cyapar_g23727.t1
Chlor_Ostlu9901_XP_001419370
Chlor_OsttaXP_022840994
Chlor_Micpu_XP_003058536
Chlor_Pedmin_4193.t1
Glauc_Cyapar_g1161.t1
Glauc_Cyapar_g1163.t1
Rhodo_Galph_G2725.1
Rhodo_Galsu_XP_005709272
Chlor_PedYPF701_CAG9466929.1
Rhodo_Graco_PXF42565.1
Rhodo_Gradom_14g272.1
Chlor_Dunsal_KAF5838267
Chlor_Dunsal_KAF5838268
Chlor_Monneg_XM_014035582.1
Chlor_Ulvmu_UM035_0024.1
Glauc_Cyapar_g20694.t1
Glauc_Cyapar_g7079.t1
Chlor_Chlpri_UPQ99880
Charo_Mesen_ME000548S08430
Lycop_Sekr_SK06G11860
Glauc_Cyapar_g18201.t1
Tip labels
Angiosperms
Gymnosperms
Ferns
Lycophytes
Bryophytes
Streptophyte algae
Chlorophyta
Glaucophyta
Rhodophyta
0.6

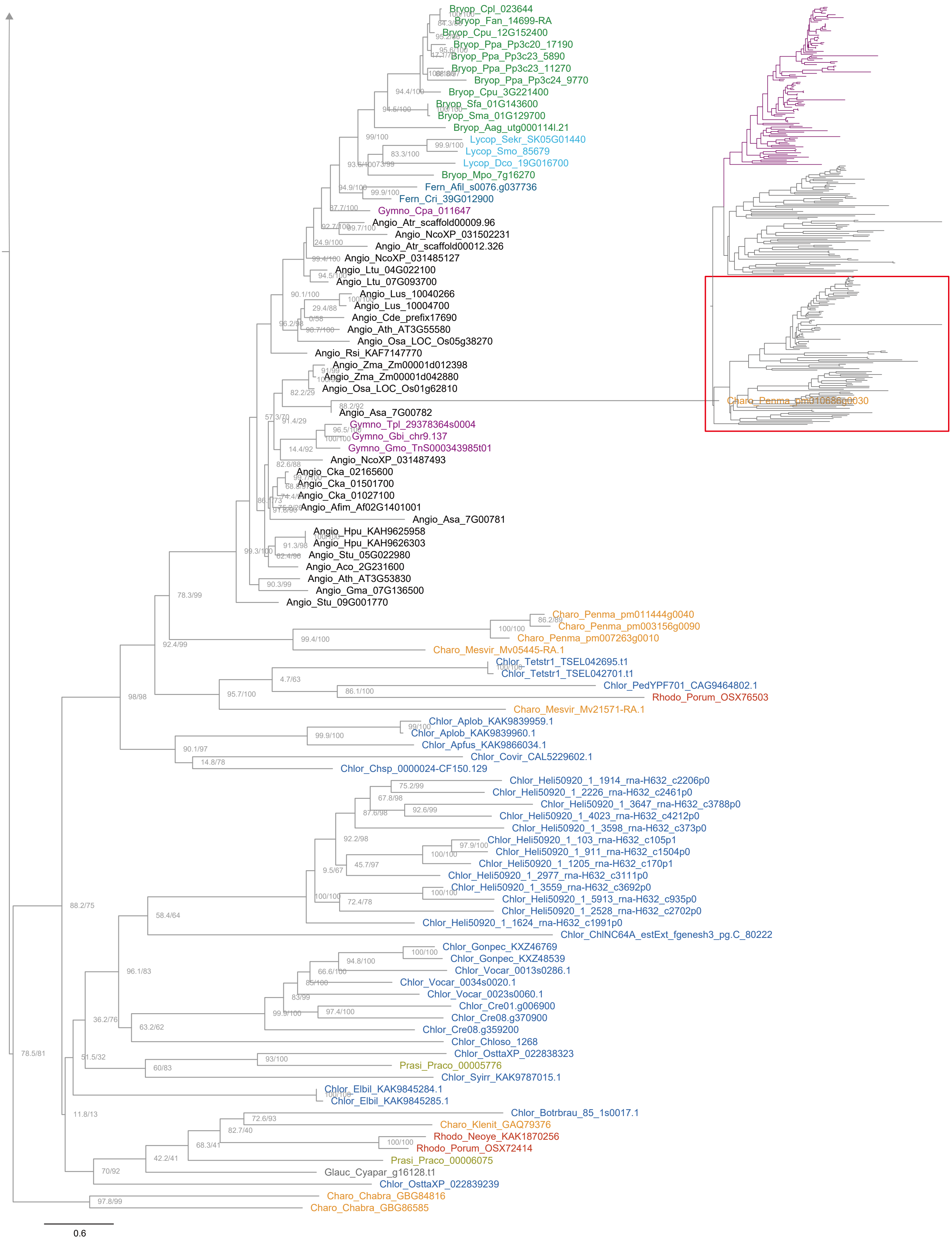

Supplementary Figure 1. Structure-guided phylogenetic analysis of UVR8. The phylogenetic tree was constructed using IQ-TREE v2.1.4 based on the best-fitting model (WAG+F+R8) with 1,000 bootstrap replicates. Purple branches represent the UVR8 clade, and the colors of tip labels represent different plant lineages.

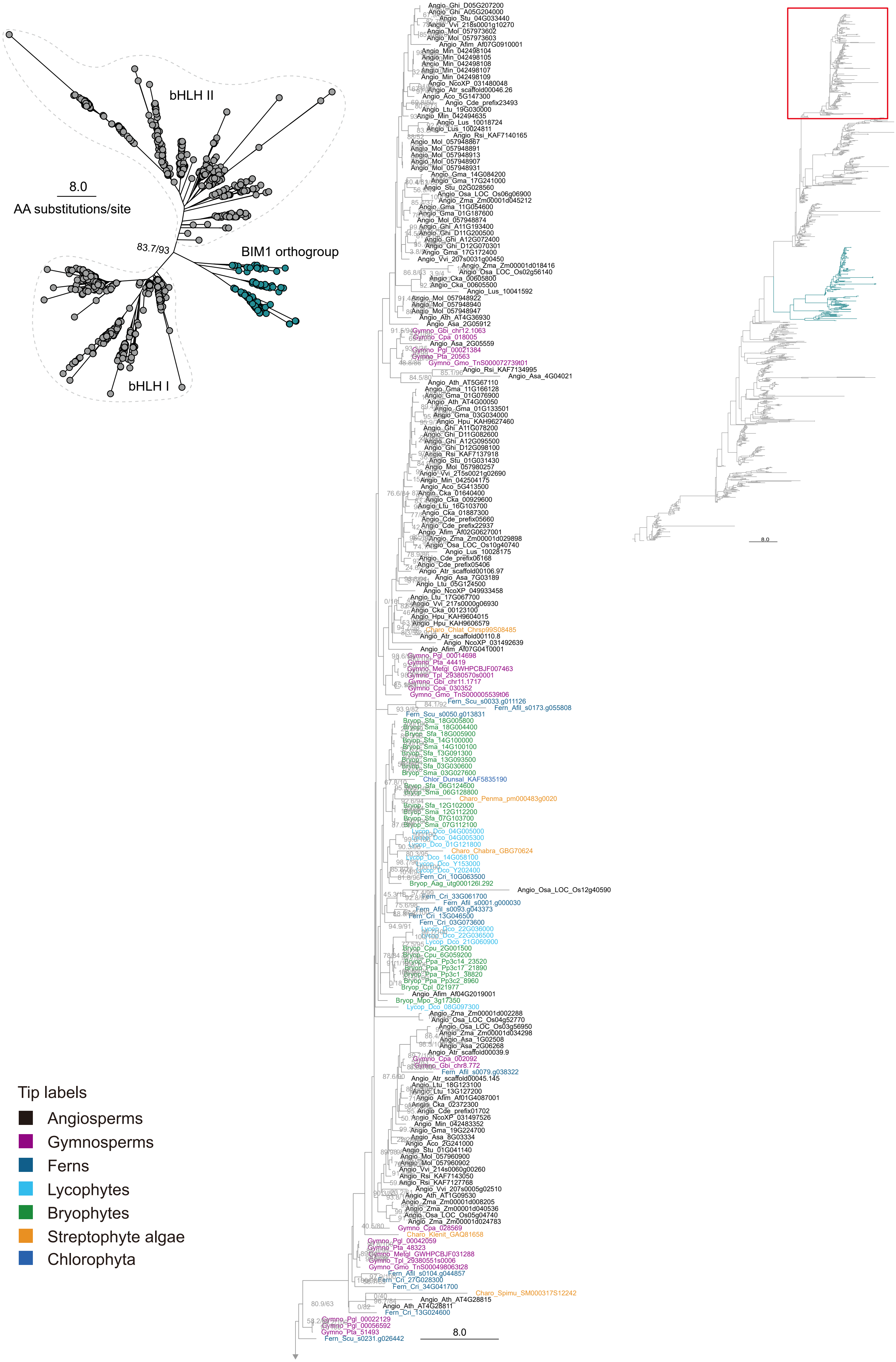

bHLH II
8.0
AA substitutions/site
83.7/93
BIM1 orthogroup
bHLH I
Tip labels
Angiosperms
Gymnosperms
Ferns
Lycophytes
Bryophytes
Streptophyte algae
Chlorophyta
Angio_Ghi_D05G207200
Angio_Ghi_A05G204000
Angio_Stu_04G033440
Angio_Vvi_218s0001g10270
Angio_Mol_057973602
Angio_Mol_057973603
Angio_Afim_Af07G0910001
Angio_Min_042498104
Angio_Min_042498105
Angio_Min_042498108
Angio_Min_042498107
Angio_Min_042498109
Angio_NcoXP_031480048
Angio_Atr_scaffold00046.26
Angio_Aco_5G147300
Angio_Cde_prefix23493
Angio_Ltu_19G030000
Angio_Min_042494635
Angio_Lus_10018724
Angio_Lus_10024811
Angio_Rsi_KAF7140165
Angio_Mol_057948867
Angio_Mol_057948891
Angio_Mol_057948913
Angio_Mol_057948907
Angio_Mol_057948931
Angio_Gma_14G084200
Angio_Gma_17G241000
Angio_Stu_02G028560
Angio_Osa_LOC_Os06g06900
Angio_Zma_Zm00001d045212
Angio_Gma_11G054600
Angio_Gma_01G187600
Angio_Mol_057948874
Angio_Ghi_A11G193400
Angio_Ghi_D11G200500
Angio_Ghi_A12G072400
Angio_Ghi_D12G070301
Angio_Gma_17G172400
Angio_Vvi_207s0031g00450
Angio_Zma_Zm00001d018416
Angio_Osa_LOC_Os02g56140
Angio_Cka_00605800
Angio_Cka_00605500
Angio_Lus_10041592
Angio_Mol_057948922
Angio_Mol_057948940
Angio_Mol_057948947
Angio_Ath_AT4G36930
Angio_Asa_2G05912
Gymno_Gbi_chr12.1063
Gymno_Cpa_018005
Angio_Asa_2G05559
Gymno_Pgl_00021384
Gymno_Pta_20563
Gymno_Gmo_TnS000072739t01
Angio_Rsi_KAF7134995
Angio_Asa_4G04021
Angio_Ath_AT5G67110
Angio_Gma_11G166128
Angio_Gma_01G076900
Angio_Ath_AT4G00050
Angio_Gma_01G133501
Angio_Gma_03G034000
Angio_Hpu_KAH9627460
Angio_Ghi_A11G078200
Angio_Ghi_D11G082600
Angio_Ghi_A12G095500
Angio_Ghi_D12G098100
Angio_Rsi_KAF7137918
Angio_Stu_01G031430
Angio_Mol_057980257
Angio_Vvi_215s0021g02690
Angio_Min_042504175
Angio_Aco_5G413500
Angio_Cka_01640400
Angio_Cka_00929600
Angio_Ltu_16G103700
Angio_Cka_01887300
Angio_Cde_prefix05660
Angio_Cde_prefix22937
Angio_Afim_Af02G0627001
Angio_Zma_Zm00001d029898
Angio_Osa_LOC_Os10g40740
Angio_Lus_10028175
Angio_Cde_prefix06168
Angio_Cde_prefix05406
Angio_Atr_scaffold00106.97
Angio_Asa_7G03189
Angio_Ltu_05G124500
Angio_NcoXP_049933458
Angio_Ltu_17G067700
Angio_Vvi_217s0000g06930
Angio_Cka_00123100
Angio_Hpu_KAH9604015
Angio_Hpu_KAH9606579
Charo_Chlat_Chrsp99S08485
Angio_Atr_scaffold00110.8
Angio_NcoXP_031492639
Angio_Afim_Af07G0410001
Gymno_Pgl_00014698
Gymno_Pta_44419
Gymno_Metgl_GWHPCBJF007463
Gymno_Tpl_29380570s0001
Gymno_Gbi_chr11.1717
Gymno_Cpa_030352
Gymno_Gmo_TnS000005539t06
Fern_Scu_s0033.g011126
Fern_Afil_s0173.g055808
Fern_Scu_s0050.g013831
Bryop_Sfa_18G005800
Bryop_Sma_18G004400
Bryop_Sfa_18G005900
Bryop_Sfa_14G100000
Bryop_Sma_14G100100
Bryop_Sfa_13G091300
Bryop_Sma_13G093500
Bryop_Sfa_03G030600
Bryop_Sma_03G027600
Chlor_Dunsal_KAF5835190
Bryop_Sfa_06G124600
Bryop_Sma_06G128800
Charo_Penma_pm000483g0020
Bryop_Sfa_12G102000
Bryop_Sma_12G112200
Bryop_Sfa_07G103700
Bryop_Sma_07G112100
Lycop_Dco_04G005000
Lycop_Dco_04G005300
Lycop_Dco_01G121800
Charo_Chabra_GBG70624
Lycop_Dco_14G058100
Lycop_Dco_Y153000
Lycop_Dco_Y202400
Fern_Cri_10G063500
Bryop_Aag_utg000126l.292
Angio_Osa_LOC_Os12g40590
Fern_Cri_33G061700
Fern_Afil_s0001.g000030
Fern_Afil_s0093.g043373
Fern_Cri_13G046500
Fern_Cri_03G073600
Lycop_Dco_22G036000
Lycop_Dco_22G036500
Lycop_Dco_21G060900
Bryop_Cpu_2G001500
Bryop_Cpu_6G059200
Bryop_Ppa_Pp3c14_23520
Bryop_Ppa_Pp3c17_21890
Bryop_Ppa_Pp3c1_38820
Bryop_Ppa_Pp3c2_8960
Bryop_Cpl_021977
Angio_Afim_Af04G2019001
Bryop_Mpo_3g17350
Lycop_Dco_08G097300
Angio_Zma_Zm00001d002288
Angio_Osa_LOC_Os04g52770
Angio_Osa_LOC_Os03g56950
Angio_Zma_Zm00001d034298
Angio_Asa_1G02508
Angio_Asa_2G06268
Angio_Atr_scaffold00039.9
Gymno_Cpa_002092
Gymno_Gbi_chr8.772
Fern_Afil_s0079.g038322
Angio_Atr_scaffold00045.145
Angio_Ltu_18G123100
Angio_Ltu_13G127200
Angio_Afim_Af01G4087001
Angio_Cka_02372300
Angio_Cde_prefix01702
Angio_NcoXP_031497526
Angio_Min_042483352
Angio_Gma_19G224700
Angio_Asa_8G03334
Angio_Aco_2G241000
Angio_Stu_01G041140
Angio_Mol_057960900
Angio_Mol_057960902
Angio_Vvi_214s0060g00260
Angio_Rsi_KAF7143050
Angio_Rsi_KAF7127768
Angio_Vvi_207s0005g02510
Angio_Ath_AT1G09530
Angio_Zma_Zm00001d008205
Angio_Zma_Zm00001d040536
Angio_Osa_LOC_Os05g04740
Angio_Zma_Zm00001d024783
Gymno_Cpa_028569
Charo_Klenit_GAQ81658
Gymno_Pgl_00042059
Gymno_Pta_48323
Gymno_Metgl_GWHPCBJF031288
Gymno_Tpl_29380551s0006
Gymno_Gmo_TnS000498063t28
Fern_Afil_s0104.g044857
Fern_Cri_27G028300
Fern_Cri_34G041700
Charo_Spimu_SM000317S12242
Angio_Ath_AT4G28815
Angio_Ath_AT4G28811
Fern_Cri_13G024600
Gymno_Pgl_00022129
Gymno_Pgl_00056592
Gymno_Pta_51493
Fern_Scu_s0231.g026442
8.0
8.0

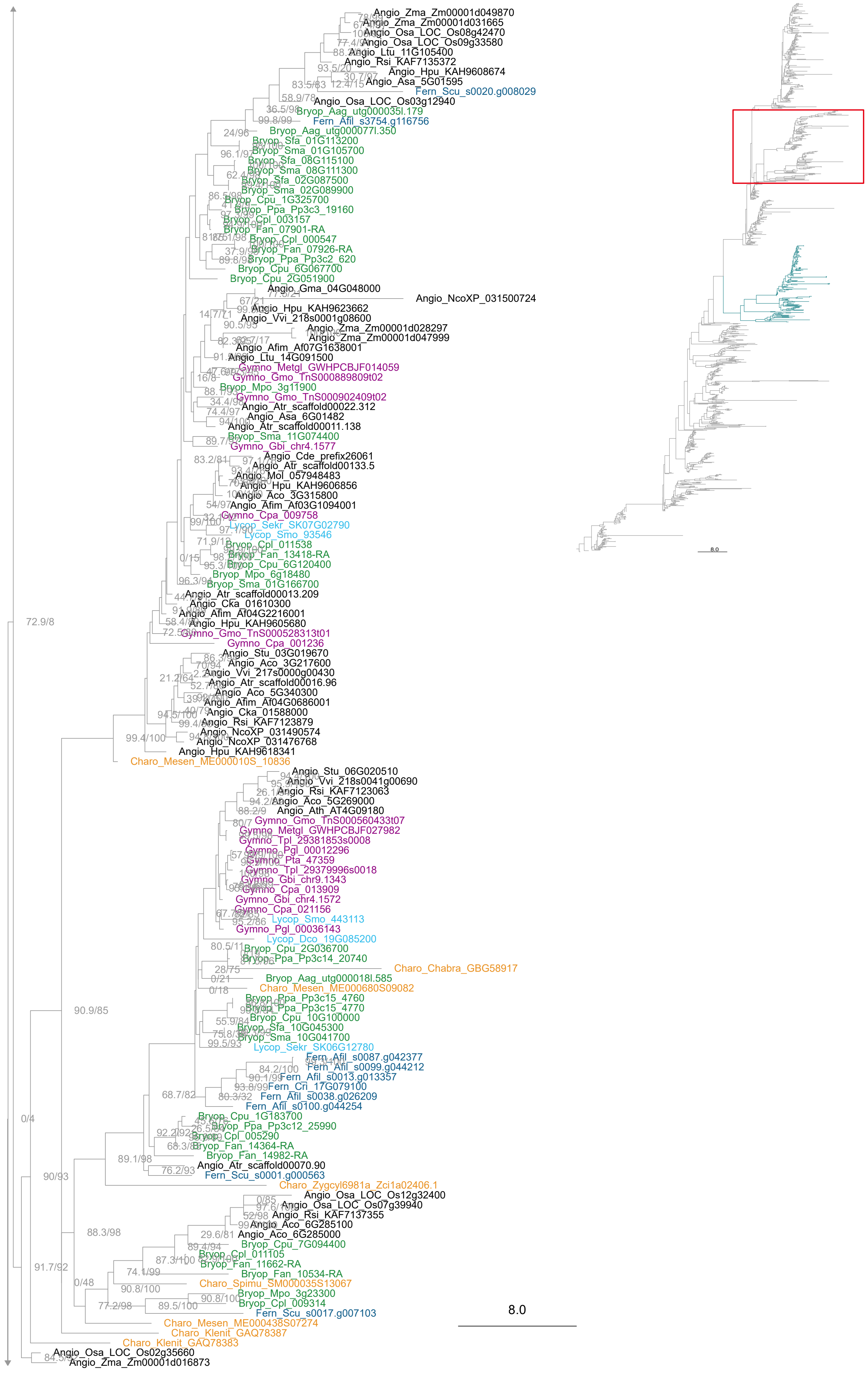

Angio_Zma_Zm00001d049870
Angio_Zma_Zm00001d031665
Angio_Osa_LOC_Os08g42470
Angio_Osa_LOC_Os09g33580
Angio_Ltu_11G105400
Angio_Rsi_KAF7135372
Angio_Hpu_KAH9608674
Angio_Asa_5G01595
Fern_Scu_s0020.g008029
Angio_Osa_LOC_Os03g12940
Bryop_Aag_utg000035l.179
Fern_Afil_s3754.g116756
Bryop_Aag_utg000077l.350
Bryop_Sfa_01G113200
Bryop_Sma_01G105700
Bryop_Sfa_08G115100
Bryop_Sma_08G111300
Bryop_Sfa_02G087500
Bryop_Sma_02G089900
Bryop_Cpu_1G325700
Bryop_Ppa_Pp3c3_19160
Bryop_Cpl_003157
Bryop_Fan_07901-RA
Bryop_Cpl_000547
Bryop_Fan_07926-RA
Bryop_Ppa_Pp3c2_620
Bryop_Cpu_6G067700
Bryop_Cpu_2G051900
Angio_Gma_04G048000
Angio_NcoXP_031500724
Angio_Hpu_KAH9623662
Angio_Vvi_218s0001g08600
Angio_Zma_Zm00001d028297
Angio_Zma_Zm00001d047999
Angio_Afim_Af07G1638001
Angio_Ltu_14G091500
Gymno_Metgl_GWHPCBJF014059
Gymno_Gmo_TnS000889809t02
Bryop_Mpo_3g11900
Gymno_Gmo_TnS000902409t02
Angio_Atr_scaffold00022.312
Angio_Asa_6G01482
Angio_Atr_scaffold00011.138
Bryop_Sma_11G074400
Gymno_Gbi_chr4.1577
Angio_Cde_prefix26061
Angio_Atr_scaffold00133.5
Angio_Mol_057948483
Angio_Hpu_KAH9606856
Angio_Aco_3G315800
Angio_Afim_Af03G1094001
Gymno_Cpa_009758
Lycop_Sekr_SK07G02790
Lycop_Smo_93546
Bryop_Cpl_011538
Bryop_Fan_13418-RA
Bryop_Cpu_6G120400
Bryop_Mpo_6g18480
Bryop_Sma_01G166700
Angio_Atr_scaffold00013.209
Angio_Cka_01610300
Angio_Afim_Af04G2216001
Angio_Hpu_KAH9605680
Gymno_Gmo_TnS000528313t01
Gymno_Cpa_001236
Angio_Stu_03G019670
Angio_Aco_3G217600
Angio_Vvi_217s0000g00430
Angio_Atr_scaffold00016.96
Angio_Aco_5G340300
Angio_Afim_Af04G0686001
Angio_Cka_01588000
Angio_Rsi_KAF7123879
Angio_NcoXP_031490574
Angio_NcoXP_031476768
Angio_Hpu_KAH9618341
Charo_Mesen_ME000010S_10836
Angio_Stu_06G020510
Angio_Vvi_218s0041g00690
Angio_Rsi_KAF7123063
Angio_Aco_5G269000
Angio_Ath_AT4G09180
Gymno_Gmo_TnS000560433t07
Gymno_Metgl_GWHPCBJF027982
Gymno_Tpl_29381853s0008
Gymno_Pgl_00012296
Gymno_Pta_47359
Gymno_Tpl_29379996s0018
Gymno_Gbi_chr9.1343
Gymno_Cpa_013909
Gymno_Gbi_chr4.1572
Gymno_Cpa_021156
Lycop_Smo_443113
Gymno_Pgl_00036143
Lycop_Dco_19G085200
Bryop_Cpu_2G036700
Bryop_Ppa_Pp3c14_20740
Charo_Chabra_GBG58917
Bryop_Aag_utg000018l.585
Charo_Mesen_ME000680S09082
Bryop_Ppa_Pp3c15_4760
Bryop_Ppa_Pp3c15_4770
Bryop_Cpu_10G100000
Bryop_Sfa_10G045300
Bryop_Sma_10G041700
Lycop_Sekr_SK06G12780
Fern_Afil_s0087.g042377
Fern_Afil_s0099.g044212
Fern_Afil_s0013.g013357
Fern_Cri_17G079100
Fern_Afil_s0038.g026209
Fern_Afil_s0100.g044254
Bryop_Cpu_1G183700
Bryop_Ppa_Pp3c12_25990
Bryop_Cpl_005290
Bryop_Fan_14364-RA
Bryop_Fan_14982-RA
Angio_Atr_scaffold00070.90
Fern_Scu_s0001.g000563
Charo_Zygcyl6981a_Zci1a02406.1
Angio_Osa_LOC_Os12g32400
Angio_Osa_LOC_Os07g39940
Angio_Rsi_KAF7137355
Angio_Aco_6G285100
Angio_Aco_6G285000
Bryop_Cpu_7G094400
Bryop_Cpl_011105
Bryop_Fan_11662-RA
Bryop_Fan_10534-RA
Charo_Spimu_SM000035S13067
Bryop_Mpo_3g23300
Bryop_Cpl_009314
Fern_Scu_s0017.g007103
Charo_Mesen_ME000438S07274
Charo_Klenit_GAQ78387
Charo_Klenit_GAQ78383
Angio_Osa_LOC_Os02g35660
Angio_Zma_Zm00001d016873
8.0

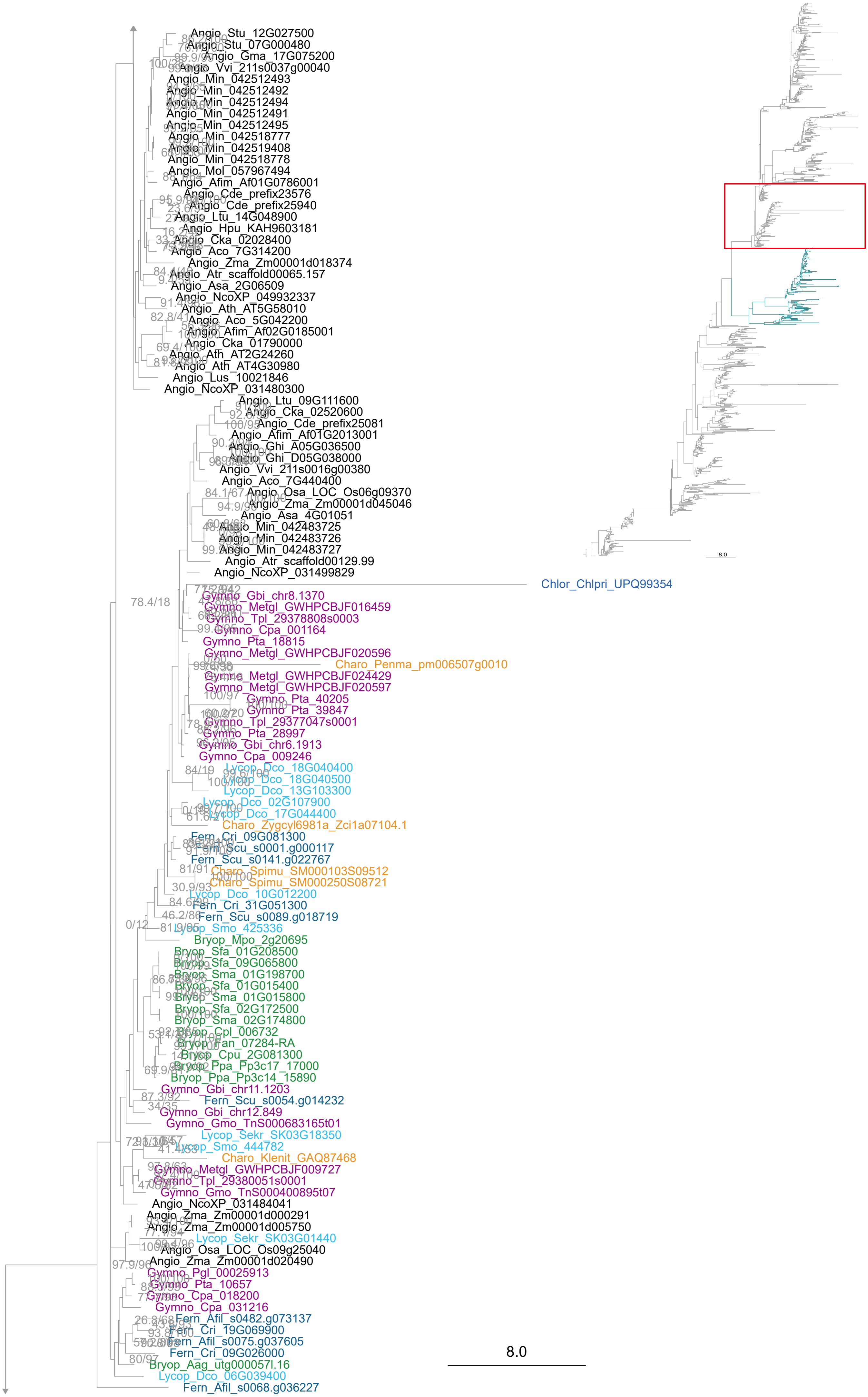

Angio_Stu_12G027500
Angio_Stu_07G000480
Angio_Gma_17G075200
Angio_Vvi_211s0037g00040
Angio_Min_042512493
Angio_Min_042512492
Angio_Min_042512494
Angio_Min_042512491
Angio_Min_042512495
Angio_Min_042518777
Angio_Min_042519408
Angio_Min_042518778
Angio_Mol_057967494
Angio_Afim_Af01G0786001
Angio_Cde_prefix23576
Angio_Cde_prefix25940
Angio_Ltu_14G048900
Angio_Hpu_KAH9603181
Angio_Cka_02028400
Angio_Aco_7G314200
Angio_Zma_Zm00001d018374
Angio_Atr_scaffold00065.157
Angio_Asa_2G06509
Angio_NcoXP_049932337
Angio_Ath_AT5G58010
Angio_Aco_5G042200
Angio_Afim_Af02G0185001
Angio_Cka_01790000
Angio_Ath_AT2G24260
Angio_Ath_AT4G30980
Angio_Lus_10021846
Angio_NcoXP_031480300
Angio_Ltu_09G111600
Angio_Cka_02520600
Angio_Cde_prefix25081
Angio_Afim_Af01G2013001
Angio_Ghi_A05G036500
Angio_Ghi_D05G038000
Angio_Vvi_211s0016g00380
Angio_Aco_7G440400
Angio_Osa_LOC_Os06g09370
Angio_Zma_Zm00001d045046
Angio_Asa_4G01051
Angio_Min_042483725
Angio_Min_042483726
Angio_Min_042483727
Angio_Atr_scaffold00129.99
Angio_NcoXP_031499829
Chlor_Chlpri_UPQ99354
78.4/18
Gymno_Gbi_chr8.1370
Gymno_Metgl_GWHPCBJF016459
Gymno_Tpl_29378808s0003
Gymno_Cpa_001164
Gymno_Pta_18815
Gymno_Metgl_GWHPCBJF020596
Charo_Penma_pm006507g0010
Gymno_Metgl_GWHPCBJF024429
Gymno_Metgl_GWHPCBJF020597
Gymno_Pta_40205
Gymno_Pta_39847
Gymno_Tpl_29377047s0001
Gymno_Pta_28997
Gymno_Gbi_chr6.1913
Gymno_Cpa_009246
Lycop_Dco_18G040400
Lycop_Dco_18G040500
Lycop_Dco_13G103300
Lycop_Dco_02G107900
Lycop_Dco_17G044400
Charo_Zygcyl6981a_Zci1a07104.1
Fern_Cri_09G081300
Fern_Scu_s0001.g000117
Fern_Scu_s0141.g022767
Charo_Spimu_SM000103S09512
Charo_Spimu_SM000250S08721
Lycop_Dco_10G012200
Fern_Cri_31G051300
Fern_Scu_s0089.g018719
0/12
Lycop_Smo_425336
Bryop_Mpo_2g20695
Bryop_Sfa_01G208500
Bryop_Sfa_09G065800
Bryop_Sma_01G198700
Bryop_Sfa_01G015400
Bryop_Sma_01G015800
Bryop_Sfa_02G172500
Bryop_Sma_02G174800
Bryop_Cpl_006732
Bryop_Fan_07284-RA
Bryop_Cpu_2G081300
Bryop_Ppa_Pp3c17_17000
Bryop_Ppa_Pp3c14_15890
Gymno_Gbi_chr11.1203
Fern_Scu_s0054.g014232
Gymno_Gbi_chr12.849
Gymno_Gmo_TnS000683165t01
Lycop_Sekr_SK03G18350
Lycop_Smo_444782
Charo_Klenit_GAQ87468
Gymno_Metgl_GWHPCBJF009727
Gymno_Tpl_29380051s0001
Gymno_Gmo_TnS000400895t07
Angio_NcoXP_031484041
Angio_Zma_Zm00001d000291
Angio_Zma_Zm00001d005750
Lycop_Sekr_SK03G01440
Angio_Osa_LOC_Os09g25040
Angio_Zma_Zm00001d020490
Gymno_Pgl_00025913
Gymno_Pta_10657
Gymno_Cpa_018200
Gymno_Cpa_031216
Fern_Afil_s0482.g073137
Fern_Cri_19G069900
Fern_Afil_s0075.g037605
Fern_Cri_09G026000
Bryop_Aag_utg000057l.16
Lycop_Dco_06G039400
Fern_Afil_s0068.g036227
8.0

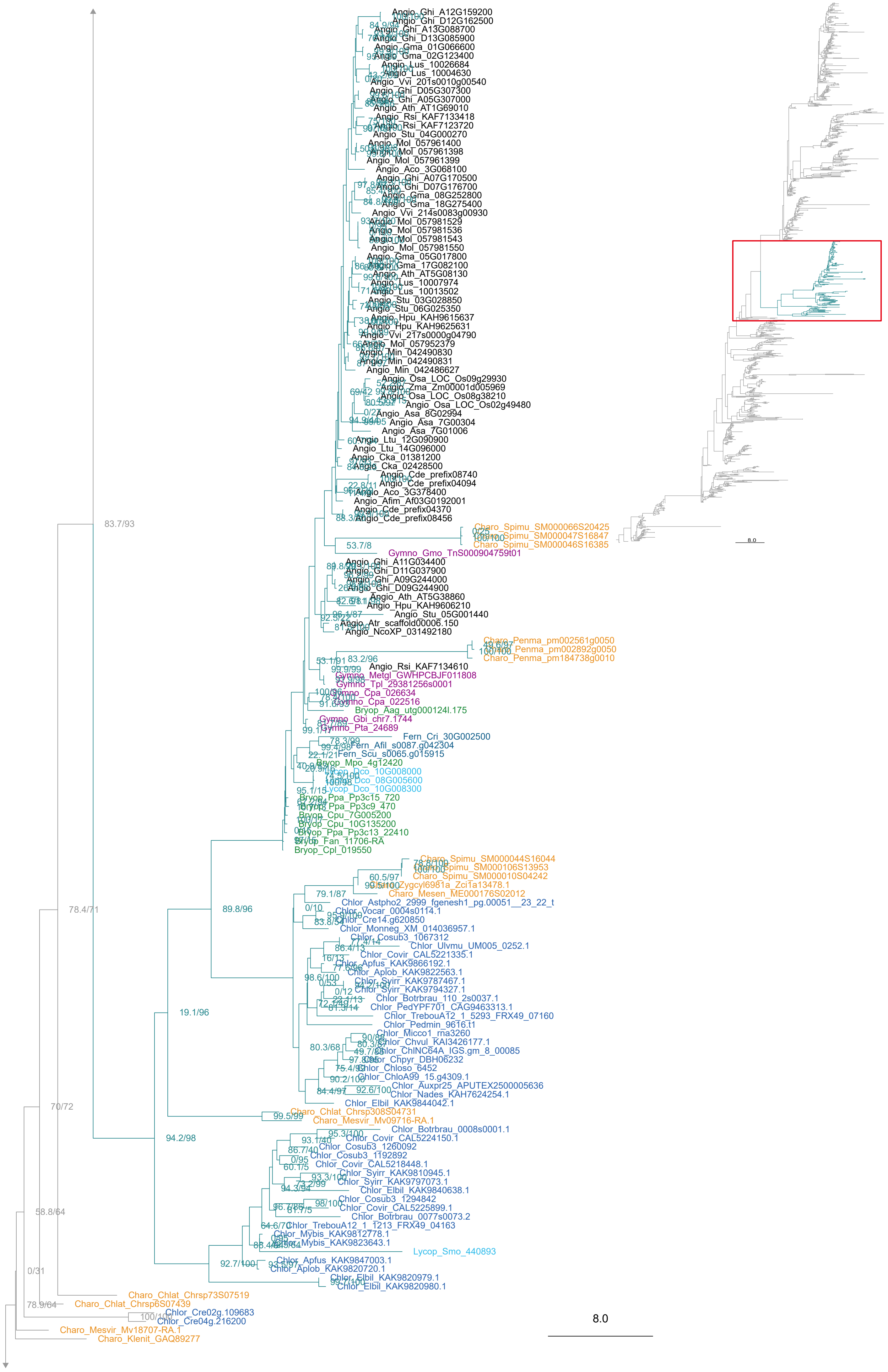

Angio_Ghi_A12G159200
Angio_Ghi_D12G162500
Angio_Ghi_A13G088700
Angio_Ghi_D13G085900
Angio_Gma_01G066600
Angio_Gma_02G123400
Angio_Lus_10026684
Angio_Lus_10004630
Angio_Vvi_201s0010g00540
Angio_Ghi_D05G307300
Angio_Ghi_A05G307000
Angio_Ath_AT1G69010
Angio_Rsi_KAF7133418
Angio_Rsi_KAF7123720
Angio_Stu_04G000270
Angio_Mol_057961400
Angio_Mol_057961398
Angio_Mol_057961399
Angio_Aco_3G068100
Angio_Ghi_A07G170500
Angio_Ghi_D07G176700
Angio_Gma_08G252800
Angio_Gma_18G275400
Angio_Vvi_214s0083g00930
Angio_Mol_057981529
Angio_Mol_057981536
Angio_Mol_057981543
Angio_Mol_057981550
Angio_Gma_05G017800
Angio_Gma_17G082100
Angio_Ath_AT5G08130
Angio_Lus_10007974
Angio_Lus_10013502
Angio_Stu_03G028850
Angio_Stu_06G025350
Angio_Hpu_KAH9615637
Angio_Hpu_KAH9625631
Angio_Vvi_217s0000g04790
Angio_Mol_057952379
Angio_Min_042490830
Angio_Min_042490831
Angio_Min_042486627
Angio_Osa_LOC_Os09g29930
Angio_Zma_Zm00001d005969
Angio_Osa_LOC_Os08g38210
Angio_Osa_LOC_Os02g49480
Angio_Asa_8G02994
Angio_Asa_7G00304
Angio_Asa_7G01006
Angio_Ltu_12G090900
Angio_Ltu_14G096000
Angio_Cka_01381200
Angio_Cka_02428500
Angio_Cde_prefix08740
Angio_Cde_prefix04094
Angio_Aco_3G378400
Angio_Afim_Af03G0192001
Angio_Cde_prefix04370
Angio_Cde_prefix08456
Charo_Spimu_SM000066S20425
Charo_Spimu_SM000047S16847
Charo_Spimu_SM000046S16385
53.7/8
Gymno_Gmo_TnS000904759t01
Angio_Ghi_A11G034400
Angio_Ghi_D11G037900
Angio_Ghi_A09G244000
Angio_Ghi_D09G244900
Angio_Ath_AT5G38860
Angio_Hpu_KAH9606210
Angio_Stu_05G001440
Angio_Atr_scaffold00006.150
Angio_NcoXP_031492180
Charo_Penma_pm002561g0050
Charo_Penma_pm002892g0050
Charo_Penma_pm184738g0010
83.2/96
Angio_Rsi_KAF7134610
Gymno_Metgl_GWHPCBJF011808
Gymno_Tpl_29381256s0001
Gymno_Cpa_026634
Gymno_Cpa_022516
Bryop_Aag_utg000124l.175
Gymno_Gbi_chr7.1744
Gymno_Pta_24689
Fern_Cri_30G002500
Fern_Afil_s0087.g042304
Fern_Scu_s0065.g015915
Bryop_Mpo_4g12420
Lycop_Dco_10G008000
Lycop_Dco_08G005600
Lycop_Dco_10G008300
Bryop_Ppa_Pp3c15_720
Bryop_Ppa_Pp3c9_470
Bryop_Cpu_7G005200
Bryop_Cpu_10G135200
Bryop_Ppa_Pp3c13_22410
Bryop_Fan_11706-RA
Bryop_Cpl_019550
Charo_Spimu_SM000044S16044
Charo_Spimu_SM000106S13953
Charo_Spimu_SM000010S04242
Charo_Zygcyl6981a_Zci1a13478.1
Charo_Mesen_ME000176S02012
Chlor_Astpho2_2999_fgenesh1_pg.00051__23_22_t
Chlor_Vocar_0004s0114.1
Chlor_Cre14.g620850
Chlor_Monneg_XM_014036957.1
Chlor_Cosub3_1067312
Chlor_Ulvmu_UM005_0252.1
Chlor_Covir_CAL5221335.1
Chlor_Apfus_KAK9866192.1
Chlor_Aplob_KAK9822563.1
Chlor_Syirr_KAK9787467.1
Chlor_Syirr_KAK9794327.1
Chlor_Botrbrau_110_2s0037.1
Chlor_PedYPF701_CAG9463313.1
Chlor_TrebouA12_1_5293_FRX49_07160
Chlor_Pedmin_9616.t1
Chlor_Micco1_rna3260
Chlor_Chvul_KAI3426177.1
Chlor_ChlNC64A_IGS.gm_8_00085
Chlor_Chpyr_DBH06232
Chlor_Chloso_6452
Chlor_ChloA99_15.g4309.1
Chlor_Auxpr25_APUTEX2500005636
Chlor_Nades_KAH7624254.1
Chlor_Elbil_KAK9844042.1
Charo_Chlat_Chrsp308S04731
Charo_Mesvir_Mv09716-RA.1
Chlor_Botrbrau_0008s0001.1
Chlor_Covir_CAL5224150.1
Chlor_Cosub3_1260092
Chlor_Cosub3_1192892
Chlor_Covir_CAL5218448.1
Chlor_Syirr_KAK9810945.1
Chlor_Syirr_KAK9797073.1
Chlor_Elbil_KAK9840638.1
Chlor_Cosub3_1294842
Chlor_Covir_CAL5225899.1
Chlor_Botrbrau_0077s0073.2
Chlor_TrebouA12_1_1213_FRX49_04163
Chlor_Mybis_KAK9812778.1
Chlor_Mybis_KAK9823643.1
Lycop_Smo_440893
Chlor_Apfus_KAK9847003.1
Chlor_Aplob_KAK9820720.1
Chlor_Elbil_KAK9820979.1
Chlor_Elbil_KAK9820980.1
Charo_Chlat_Chrsp73S07519
Charo_Chlat_Chrsp6S07439
Chlor_Cre02g.109683
Chlor_Cre04g.216200
Charo_Mesvir_Mv18707-RA.1
Charo_Klenit_GAQ89277
83.7/93
78.4/71
89.8/96
19.1/96
94.2/98
70/72
58.8/64
0/31
78.9/64
100/100
92.7/100
99.5/99
79.1/87
80.3/68
8.0

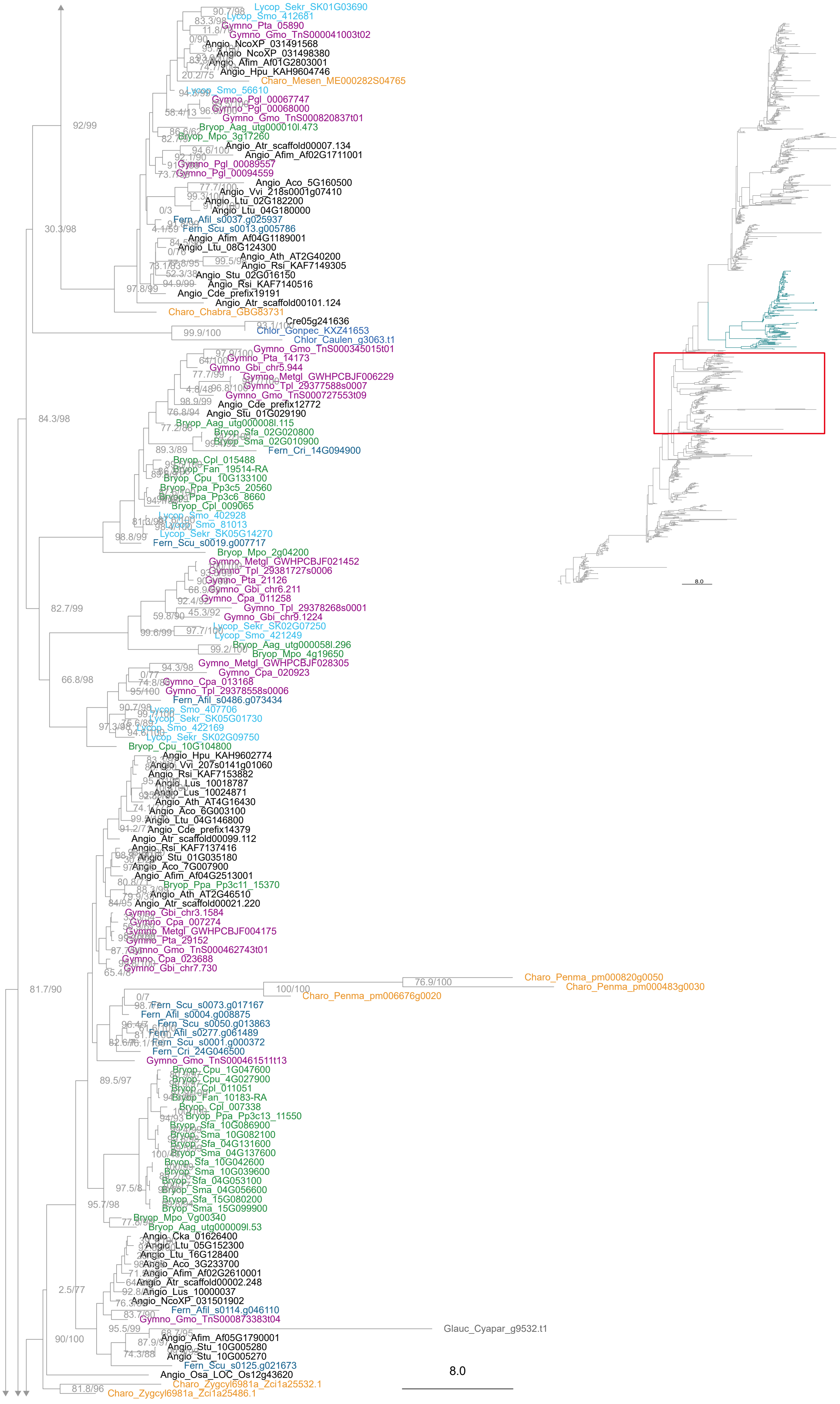
Lycop_Sekr_SK01G03690
Lycop_Smo_412681
Gymno_Pta_05890
Gymno_Gmo_TnS000041003t02
Angio_NcoXP_031491568
Angio_NcoXP_031498380
Angio_Afim_Af01G2803001
Angio_Hpu_KAH9604746
Charo_Mesen_ME000282S04765
Lycop_Smo_56610
Gymno_Pgl_00067747
Gymno_Pgl_00068000
Gymno_Gmo_TnS000820837t01
Bryop_Aag_utg000010l.473
Bryop_Mpo_3g17260
Angio_Atr_scaffold00007.134
Angio_Afim_Af02G1711001
Gymno_Pgl_00089557
Gymno_Pgl_00094559
Angio_Aco_5G160500
Angio_Vvi_218s0001g07410
Angio_Ltu_02G182200
Angio_Ltu_04G180000
Fern_Afil_s0037.g025937
Fern_Scu_s0013.g005786
Angio_Afim_Af04G1189001
Angio_Ltu_08G124300
Angio_Ath_AT2G40200
Angio_Rsi_KAF7149305
Angio_Stu_02G016150
Angio_Rsi_KAF7140516
Angio_Cde_prefix19191
Angio_Atr_scaffold00101.124
Charo_Chabra_GBG83731
Cre05g241636
Chlor_Gonpec_KXZ41653
Chlor_Caulen_g3063.t1
Gymno_Gmo_TnS000345015t01
Gymno_Pta_14173
Gymno_Gbi_chr5.944
Gymno_Metgl_GWHPCBJF006229
Gymno_Tpl_29377588s0007
Gymno_Gmo_TnS000727553t09
Angio_Cde_prefix12772
Angio_Stu_01G029190
Bryop_Aag_utg000008l.115
Bryop_Sfa_02G020800
Bryop_Sma_02G010900
Fern_Cri_14G094900
Bryop_Cpl_015488
Bryop_Fan_19514-RA
Bryop_Cpu_10G133100
Bryop_Ppa_Pp3c5_20560
Bryop_Ppa_Pp3c6_8660
Bryop_Cpl_009065
Lycop_Smo_402928
Lycop_Smo_81013
Lycop_Sekr_SK05G14270
Fern_Scu_s0019.g007717
Bryop_Mpo_2g04200
Gymno_Metgl_GWHPCBJF021452
Gymno_Tpl_29381727s0006
Gymno_Pta_21126
Gymno_Gbi_chr6.211
Gymno_Cpa_011258
Gymno_Tpl_29378268s0001
Gymno_Gbi_chr9.1224
Lycop_Sekr_SK02G07250
Lycop_Smo_421249
Bryop_Aag_utg000058l.296
Bryop_Mpo_4g19650
Gymno_Metgl_GWHPCBJF028305
Gymno_Cpa_020923
Gymno_Cpa_013168
Gymno_Tpl_29378558s0006
Fern_Afil_s0486.g073434
Lycop_Smo_407706
Lycop_Sekr_SK05G01730
Lycop_Smo_422169
Lycop_Sekr_SK02G09750
Bryop_Cpu_10G104800
Angio_Hpu_KAH9602774
Angio_Vvi_207s0141g01060
Angio_Rsi_KAF7153882
Angio_Lus_10018787
Angio_Lus_10024871
Angio_Ath_AT4G16430
Angio_Aco_6G003100
Angio_Ltu_04G146800
Angio_Cde_prefix14379
Angio_Atr_scaffold00099.112
Angio_Rsi_KAF7137416
Angio_Stu_01G035180
Angio_Aco_7G007900
Angio_Afim_Af04G2513001
Bryop_Ppa_Pp3c11_15370
Angio_Ath_AT2G46510
Angio_Atr_scaffold00021.220
Gymno_Gbi_chr3.1584
Gymno_Cpa_007274
Gymno_Metgl_GWHPCBJF004175
Gymno_Pta_29152
Gymno_Gmo_TnS000462743t01
Gymno_Cpa_023688
Gymno_Gbi_chr7.730
Charo_Penma_pm000820g0050
Charo_Penma_pm000483g0030
Charo_Penma_pm006676g0020
Fern_Scu_s0073.g017167
Fern_Afil_s0004.g008875
Fern_Scu_s0050.g013863
Fern_Afil_s0277.g061489
Fern_Scu_s0001.g000372
Fern_Cri_24G046500
Gymno_Gmo_TnS000461511t13
Bryop_Cpu_1G047600
Bryop_Cpu_4G027900
Bryop_Cpl_011051
Bryop_Fan_10183-RA
Bryop_Cpl_007338
Bryop_Ppa_Pp3c13_11550
Bryop_Sfa_10G086900
Bryop_Sma_10G082100
Bryop_Sfa_04G131600
Bryop_Sma_04G137600
Bryop_Sfa_10G042600
Bryop_Sma_10G039600
Bryop_Sfa_04G053100
Bryop_Sma_04G056600
Bryop_Sfa_15G080200
Bryop_Sma_15G099900
Bryop_Mpo_Vg00340
Bryop_Aag_utg000009l.53
Angio_Cka_01626400
Angio_Ltu_05G152300
Angio_Ltu_16G128400
Angio_Aco_3G233700
Angio_Afim_Af02G2610001
Angio_Atr_scaffold00002.248
Angio_Lus_10000037
Angio_NcoXP_031501902
Fern_Afil_s0114.g046110
Gymno_Gmo_TnS000873383t04
Glauc_Cyapar_g9532.t1
Angio_Afim_Af05G1790001
Angio_Stu_10G005280
Angio_Stu_10G005270
Fern_Scu_s0125.g021673
Angio_Osa_LOC_Os12g43620
Charo_Zygcyl6981a_Zci1a25532.1
Charo_Zygcyl6981a_Zci1a25486.1
92/99
30.3/98
84.3/98
82.7/99
66.8/98
81.7/90
89.5/97
95.7/98
2.5/77
90/100
99.9/100
100/100
76.9/100
81.8/96
8.0

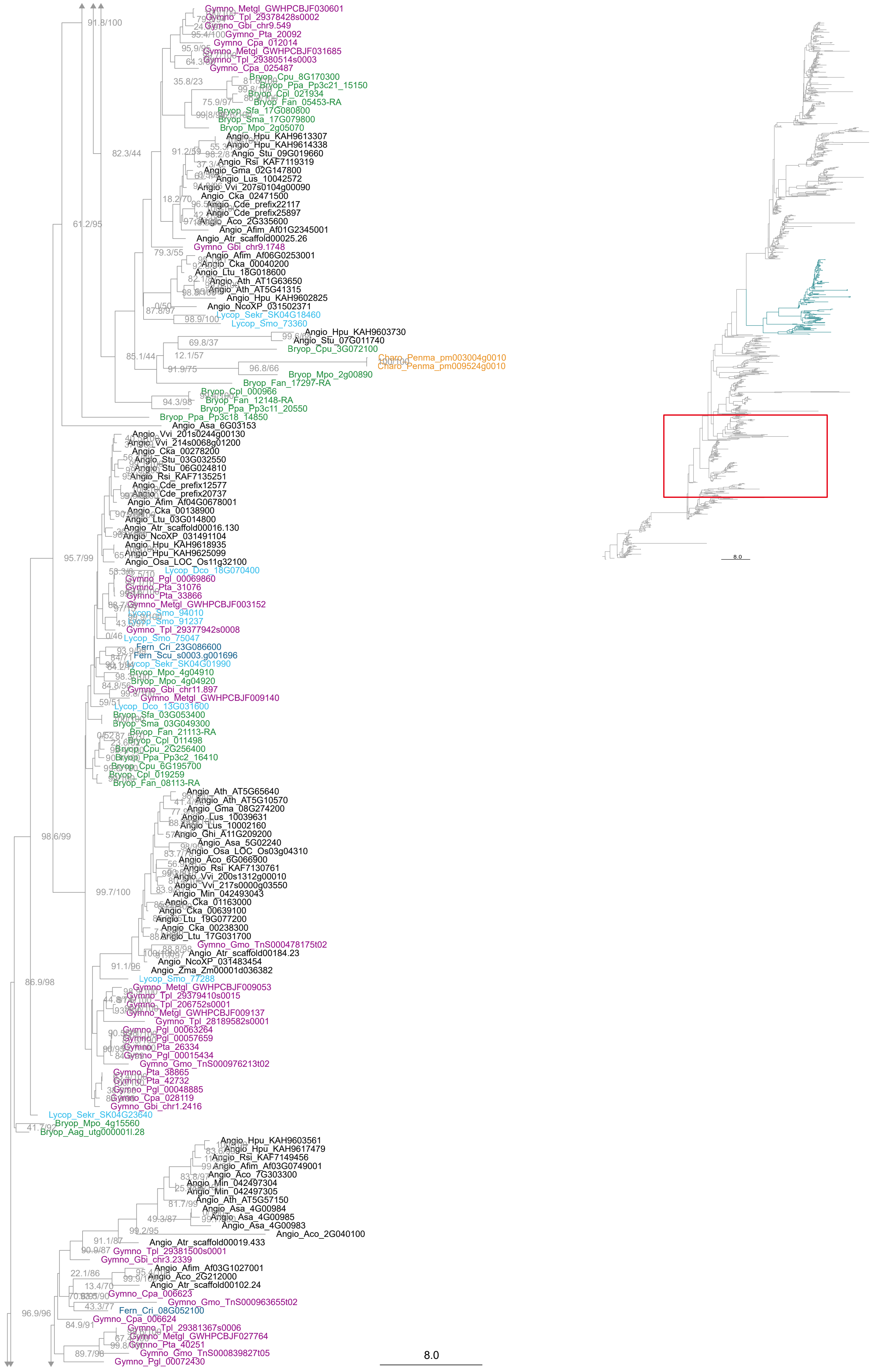
Gymno_Metgl_GWHPCBJF030601
Gymno_Tpl_29378428s0002
Gymno_Gbi_chr9.549
Gymno_Pta_20092
Gymno_Cpa_012014
Gymno_Metgl_GWHPCBJF031685
Gymno_Tpl_29380514s0003
Gymno_Cpa_025487
Bryop_Cpu_8G170300
Bryop_Ppa_Pp3c21_15150
Bryop_Cpl_021934
Bryop_Fan_05453-RA
Bryop_Sfa_17G080800
Bryop_Sma_17G079800
Bryop_Mpo_2g05070
Angio_Hpu_KAH9613307
Angio_Hpu_KAH9614338
Angio_Stu_09G019660
Angio_Rsi_KAF7119319
Angio_Gma_02G147800
Angio_Lus_10042572
Angio_Vvi_207s0104g00090
Angio_Cka_02471500
Angio_Cde_prefix22117
Angio_Cde_prefix25897
Angio_Aco_2G335600
Angio_Afim_Af01G2345001
Angio_Atr_scaffold00025.26
Gymno_Gbi_chr9.1748
Angio_Afim_Af06G0253001
Angio_Cka_00040200
Angio_Ltu_18G018600
Angio_Ath_AT1G63650
Angio_Ath_AT5G41315
Angio_Hpu_KAH9602825
Angio_NcoXP_031502371
Lycop_Sekr_SK04G18460
Lycop_Smo_73360
Angio_Hpu_KAH9603730
Angio_Stu_07G011740
Bryop_Cpu_3G072100
Charo_Penma_pm003004g0010
Charo_Penma_pm009524g0010
Bryop_Mpo_2g00890
Bryop_Fan_17297-RA
Bryop_Cpl_000966
Bryop_Fan_12148-RA
Bryop_Ppa_Pp3c11_20550
Bryop_Ppa_Pp3c18_14850
Angio_Asa_6G03153
Angio_Vvi_201s0244g00130
Angio_Vvi_214s0068g01200
Angio_Cka_00278200
Angio_Stu_03G032550
Angio_Stu_06G024810
Angio_Rsi_KAF7135251
Angio_Cde_prefix12577
Angio_Cde_prefix20737
Angio_Afim_Af04G0678001
Angio_Cka_00138900
Angio_Ltu_03G014800
Angio_Atr_scaffold00016.130
Angio_NcoXP_031491104
Angio_Hpu_KAH9618935
Angio_Hpu_KAH9625099
Angio_Osa_LOC_Os11g32100
Lycop_Dco_18G070400
Gymno_Pgl_00069860
Gymno_Pta_31076
Gymno_Pta_33866
Gymno_Metgl_GWHPCBJF003152
Lycop_Smo_94010
Lycop_Smo_91237
Gymno_Tpl_29377942s0008
Lycop_Smo_75047
Fern_Cri_23G086600
Fern_Scu_s0003.g001696
Lycop_Sekr_SK04G01990
Bryop_Mpo_4g04910
Bryop_Mpo_4g04920
Gymno_Gbi_chr11.897
Gymno_Metgl_GWHPCBJF009140
Lycop_Dco_13G031600
Bryop_Sfa_03G053400
Bryop_Sma_03G049300
Bryop_Fan_21113-RA
Bryop_Cpl_011498
Bryop_Cpu_2G256400
Bryop_Ppa_Pp3c2_16410
Bryop_Cpu_6G195700
Bryop_Cpl_019259
Bryop_Fan_08113-RA
Angio_Ath_AT5G65640
Angio_Ath_AT5G10570
Angio_Gma_08G274200
Angio_Lus_10039631
Angio_Lus_10002160
Angio_Ghi_A11G209200
Angio_Asa_5G02240
Angio_Osa_LOC_Os03g04310
Angio_Aco_6G066900
Angio_Rsi_KAF7130761
Angio_Vvi_200s1312g00010
Angio_Vvi_217s0000g03550
Angio_Min_042493043
Angio_Cka_01163000
Angio_Cka_00639100
Angio_Ltu_19G077200
Angio_Cka_00238300
Angio_Ltu_17G031700
Gymno_Gmo_TnS000478175t02
Angio_Atr_scaffold00184.23
Angio_NcoXP_031483454
Angio_Zma_Zm00001d036382
Lycop_Smo_77288
Gymno_Metgl_GWHPCBJF009053
Gymno_Tpl_29379410s0015
Gymno_Tpl_206752s0001
Gymno_Metgl_GWHPCBJF009137
Gymno_Tpl_28189582s0001
Gymno_Pgl_00063264
Gymno_Pgl_00057659
Gymno_Pta_26334
Gymno_Pgl_00015434
Gymno_Gmo_TnS000976213t02
Gymno_Pta_38865
Gymno_Pta_42732
Gymno_Pgl_00048885
Gymno_Cpa_028119
Gymno_Gbi_chr1.2416
Lycop_Sekr_SK04G23640
Bryop_Mpo_4g15560
Bryop_Aag_utg000001l.28
Angio_Hpu_KAH9603561
Angio_Hpu_KAH9617479
Angio_Rsi_KAF7149456
Angio_Afim_Af03G0749001
Angio_Aco_7G303300
Angio_Min_042497304
Angio_Min_042497305
Angio_Ath_AT5G57150
Angio_Asa_4G00984
Angio_Asa_4G00985
Angio_Asa_4G00983
Angio_Aco_2G040100
Angio_Atr_scaffold00019.433
Gymno_Tpl_29381500s0001
Gymno_Gbi_chr3.2339
Angio_Afim_Af03G1027001
Angio_Aco_2G212000
Angio_Atr_scaffold00102.24
Gymno_Cpa_006623
Gymno_Gmo_TnS000963655t02
Fern_Cri_08G052100
Gymno_Cpa_006624
Gymno_Tpl_29381367s0006
Gymno_Metgl_GWHPCBJF027764
Gymno_Pta_40251
Gymno_Gmo_TnS000839827t05
Gymno_Pgl_00072430
91.8/100
35.8/23
75.9/97
82.3/44
91.2/59
18.2/70
61.2/95
79.3/55
87.8/97
98.9/100
69.8/37
85.1/44
12.1/57
91.9/75
96.8/66
94.3/98
95.7/99
98.6/99
99.7/100
91.1/96
86.9/98
41.7/92
81.7/99
49.3/87
99.2/95
91.1/87
90.9/87
22.1/86
13.4/70
43.3/77
96.9/96
84.9/91
89.7/98
8.0

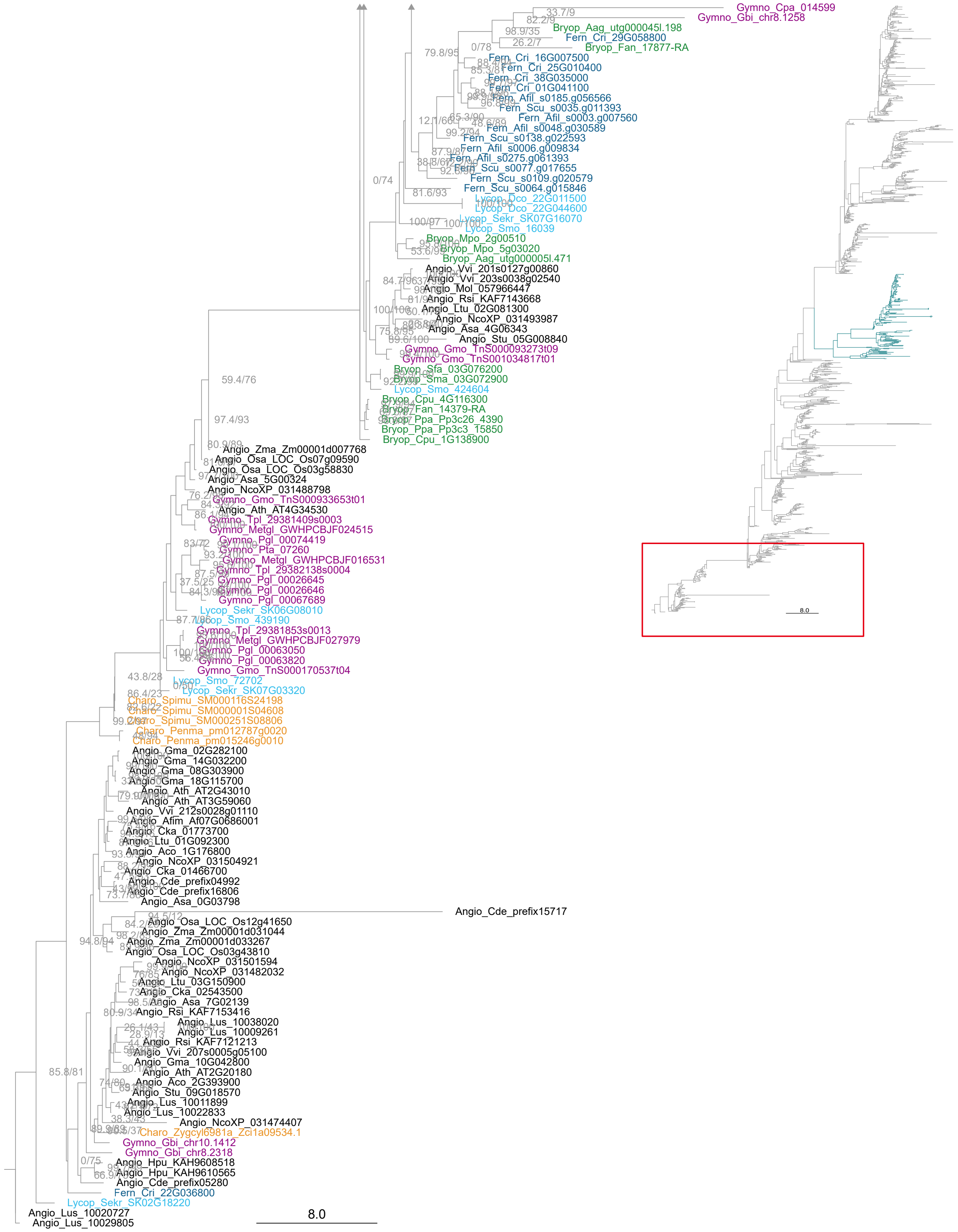

Supplementary Figure 2. Structure-guided phylogenetic analysis of BIM1. The phylogenetic tree was constructed using IQ-TREE v2.1.4 based on the best-fitting model (JTT+F+R10) with 1,000 bootstrap replicates. Green branches represent the BIM1 clade, and the colors of tip labels represent different plant lineages.

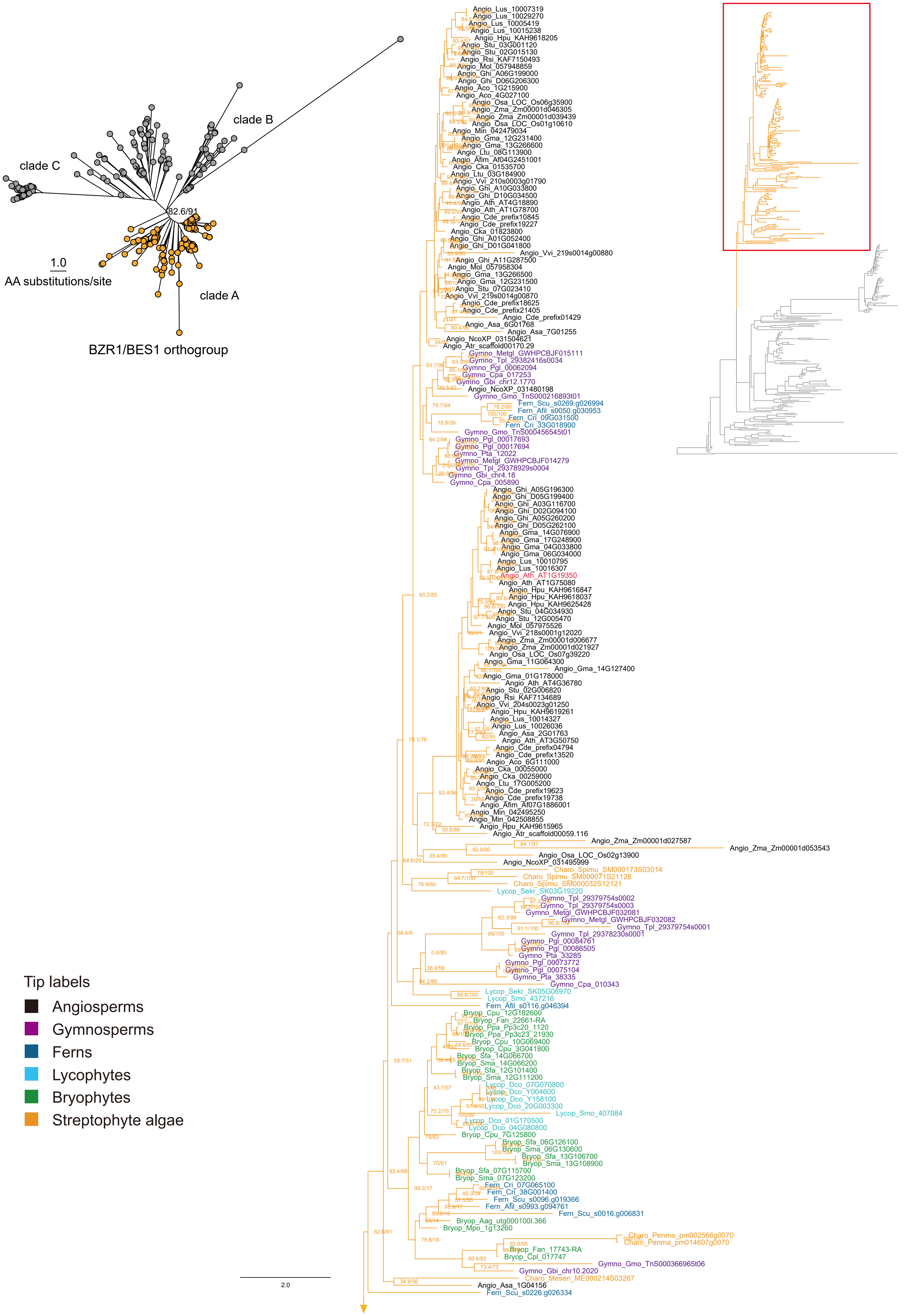
clade B
clade C
82.6/91
1.0
AA substitutions/site
clade A
BZR1/BES1 orthogroup
Angio_Lus_10007319
Angio_Lus_10029270
Angio_Lus_10005419
Angio_Lus_10015238
Angio_Hpu_KAH9618205
Angio_Stu_03G001120
Angio_Stu_02G015130
Angio_Rsi_KAF7150493
Angio_Mol_057948859
Angio_Ghi_A06G199000
Angio_Ghi_D06G206300
Angio_Aco_1G215900
Angio_Aco_4G027100
Angio_Osa_LOC_Os06g35900
Angio_Zma_Zm00001d046305
Angio_Zma_Zm00001d039439
Angio_Osa_LOC_Os01g10610
Angio_Min_042479034
Angio_Gma_12G231400
Angio_Gma_13G266600
Angio_Ltu_08G113900
Angio_Afim_Af04G2451001
Angio_Cka_01535700
Angio_Ltu_03G184900
Angio_Vvi_210s0003g01790
Angio_Ghi_A10G033800
Angio_Ghi_D10G034500
Angio_Ath_AT4G18890
Angio_Ath_AT1G78700
Angio_Cde_prefix10845
Angio_Cde_prefix19227
Angio_Cka_01823800
Angio_Ghi_A01G052400
Angio_Ghi_D01G041800
Angio_Vvi_219s0014g00880
Angio_Ghi_A11G287500
Angio_Mol_057958304
Angio_Gma_13G266500
Angio_Gma_12G231500
Angio_Stu_07G023410
Angio_Vvi_219s0014g00870
Angio_Cde_prefix18625
Angio_Cde_prefix21405
Angio_Cde_prefix01429
Angio_Asa_6G01768
Angio_Asa_7G01255
Angio_NcoXP_031504621
Angio_Atr_scaffold00170.29
Gymno_Metgl_GWHPCBJF015111
Gymno_Tpl_29382416s0034
Gymno_Pgl_00062094
Gymno_Cpa_017253
Gymno_Gbi_chr12.1770
Angio_NcoXP_031480198
Gymno_Gmo_TnS000216893t01
Fern_Scu_s0269.g026994
Fern_Afil_s0050.g030953
Fern_Cri_09G031500
Fern_Cri_33G018900
Gymno_Gmo_TnS000456545t01
Gymno_Pgl_00017693
Gymno_Pgl_00017694
Gymno_Pta_12022
Gymno_Metgl_GWHPCBJF014279
Gymno_Tpl_29378929s0004
Gymno_Gbi_chr4.18
Gymno_Cpa_005890
Angio_Ghi_A05G196300
Angio_Ghi_D05G199400
Angio_Ghi_A03G116700
Angio_Ghi_D02G094100
Angio_Ghi_A05G260200
Angio_Ghi_D05G262100
Angio_Gma_14G076900
Angio_Gma_17G248900
Angio_Gma_04G033800
Angio_Gma_06G034000
Angio_Lus_10010795
Angio_Lus_10016307
Angio_Ath_AT1G19350
Angio_Ath_AT1G75080
Angio_Hpu_KAH9616847
Angio_Hpu_KAH9618037
Angio_Hpu_KAH9625428
Angio_Stu_04G034930
Angio_Stu_12G005470
Angio_Mol_057975526
Angio_Vvi_218s0001g12020
Angio_Zma_Zm00001d006677
Angio_Zma_Zm00001d021927
Angio_Osa_LOC_Os07g39220
Angio_Gma_11G064300
Angio_Gma_14G127400
Angio_Gma_01G178000
Angio_Ath_AT4G36780
Angio_Stu_02G006820
Angio_Rsi_KAF7134689
Angio_Vvi_204s0023g01250
Angio_Hpu_KAH9619261
Angio_Lus_10014327
Angio_Lus_10026036
Angio_Asa_2G01763
Angio_Ath_AT3G50750
Angio_Cde_prefix04794
Angio_Cde_prefix13520
Angio_Aco_6G111000
Angio_Cka_00055000
Angio_Cka_00259000
Angio_Ltu_17G005200
Angio_Cde_prefix19623
Angio_Cde_prefix19738
Angio_Afim_Af07G1886001
Angio_Min_042495250
Angio_Min_042508855
Angio_Hpu_KAH9615965
Angio_Atr_scaffold00059.116
Angio_Zma_Zm00001d027587
Angio_Zma_Zm00001d053543
Angio_Osa_LOC_Os02g13900
Angio_NcoXP_031495999
Charo_Spimu_SM000173S03014
Charo_Spimu_SM000071S21128
Charo_Spimu_SM000032S12121
Lycop_Sekr_SK03G19220
Gymno_Tpl_29379754s0002
Gymno_Tpl_29379754s0003
Gymno_Metgl_GWHPCBJF032081
Gymno_Metgl_GWHPCBJF032082
Gymno_Tpl_29379754s0001
Gymno_Tpl_29378230s0001
Gymno_Pgl_00084761
Gymno_Pgl_00086505
Gymno_Pta_33285
Gymno_Pgl_00073772
Gymno_Pgl_00075104
Gymno_Pta_38335
Gymno_Cpa_010343
Lycop_Sekr_SK05G06970
Lycop_Smo_437216
Fern_Afil_s0116.g046394
Bryop_Cpu_12G182600
Bryop_Fan_22661-RA
Bryop_Ppa_Pp3c20_1120
Bryop_Ppa_Pp3c23_21930
Bryop_Cpu_10G069400
Bryop_Cpu_3G041800
Bryop_Sfa_14G066700
Bryop_Sma_14G066200
Bryop_Sfa_12G101400
Bryop_Sma_12G111200
Lycop_Dco_07G070800
Lycop_Dco_Y004600
Lycop_Dco_Y158100
Lycop_Dco_20G003300
Lycop_Smo_407084
Lycop_Dco_01G170500
Lycop_Dco_04G080800
Bryop_Cpu_7G125800
Bryop_Sfa_06G126100
Bryop_Sma_06G130600
Bryop_Sfa_13G106700
Bryop_Sma_13G108900
Bryop_Sfa_07G115700
Bryop_Sma_07G123200
Fern_Cri_07G065100
Fern_Cri_38G001400
Fern_Scu_s0096.g019366
Fern_Afil_s0993.g094761
Fern_Scu_s0016.g006831
Bryop_Aag_utg000100l.366
Bryop_Mpo_1g13260
Charo_Penma_pm002566g0070
Charo_Penma_pm014607g0070
Bryop_Fan_17743-RA
Bryop_Cpl_017747
Gymno_Gmo_TnS000366965t06
Gymno_Gbi_chr10.2020
Charo_Mesen_ME000214S03267
Angio_Asa_1G04156
Fern_Scu_s0226.g026334
2.0
Tip labels
Angiosperms
Gymnosperms
Ferns
Lycophytes
Bryophytes
Streptophyte algae

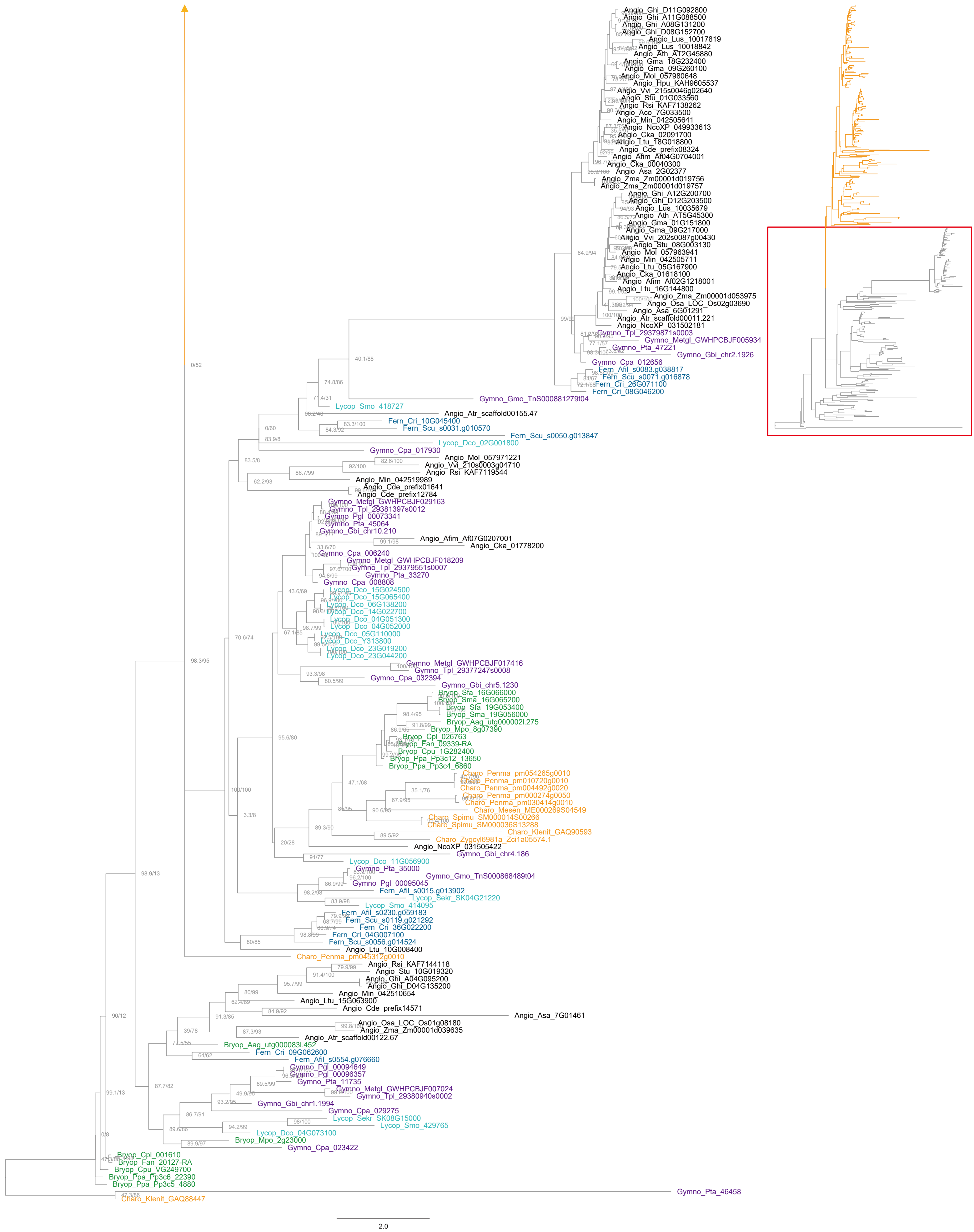

Supplementary Figure 3. Structure-guided phylogenetic analysis of BES1. The phylogenetic tree was constructed using IQ-TREE v2.1.4 based on the best-fitting model (JTT+F+R7) with 1,000 bootstrap replicates. Orange branches represent the BES1 clade, and the colors of tip labels represent different plant lineages.

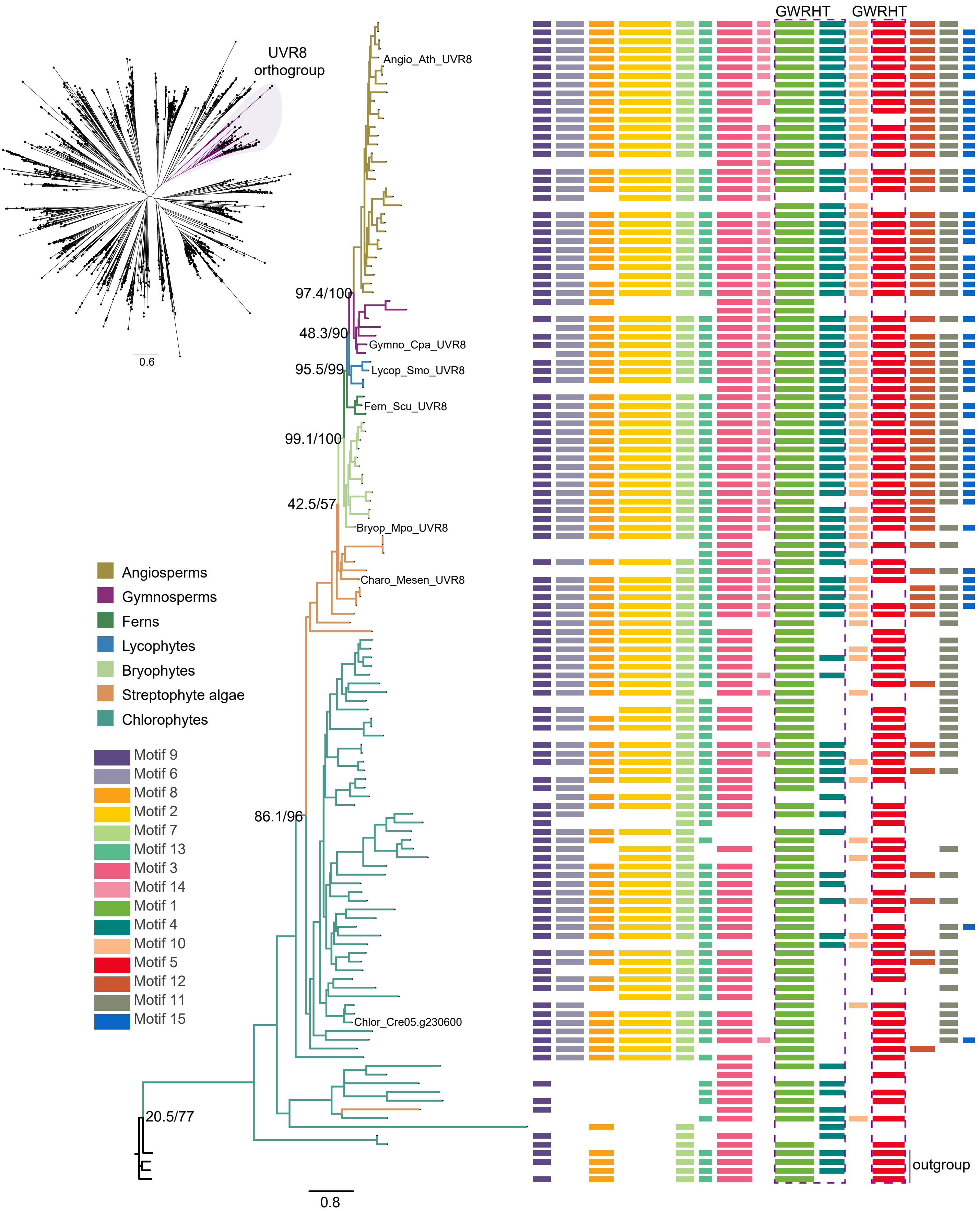

Supplementary Figure 4. Phylogenetic tree and domain architecture of *UVR8* orthologs. Maximum-likelihood phylogeny based on amino acid sequence alignments, with the LG+I+R7 model and 1,000 ultrafast bootstraps. The closest paralogs (black branches) were used to root the tree. Scale bar: amino acid substitutions per site. MEME-identified motifs are shown to the right as colored boxes; the dashed box marks the WGRHT-containing motif critical for UV-B perception. Motif sequences are listed in Supplementary Table 10.

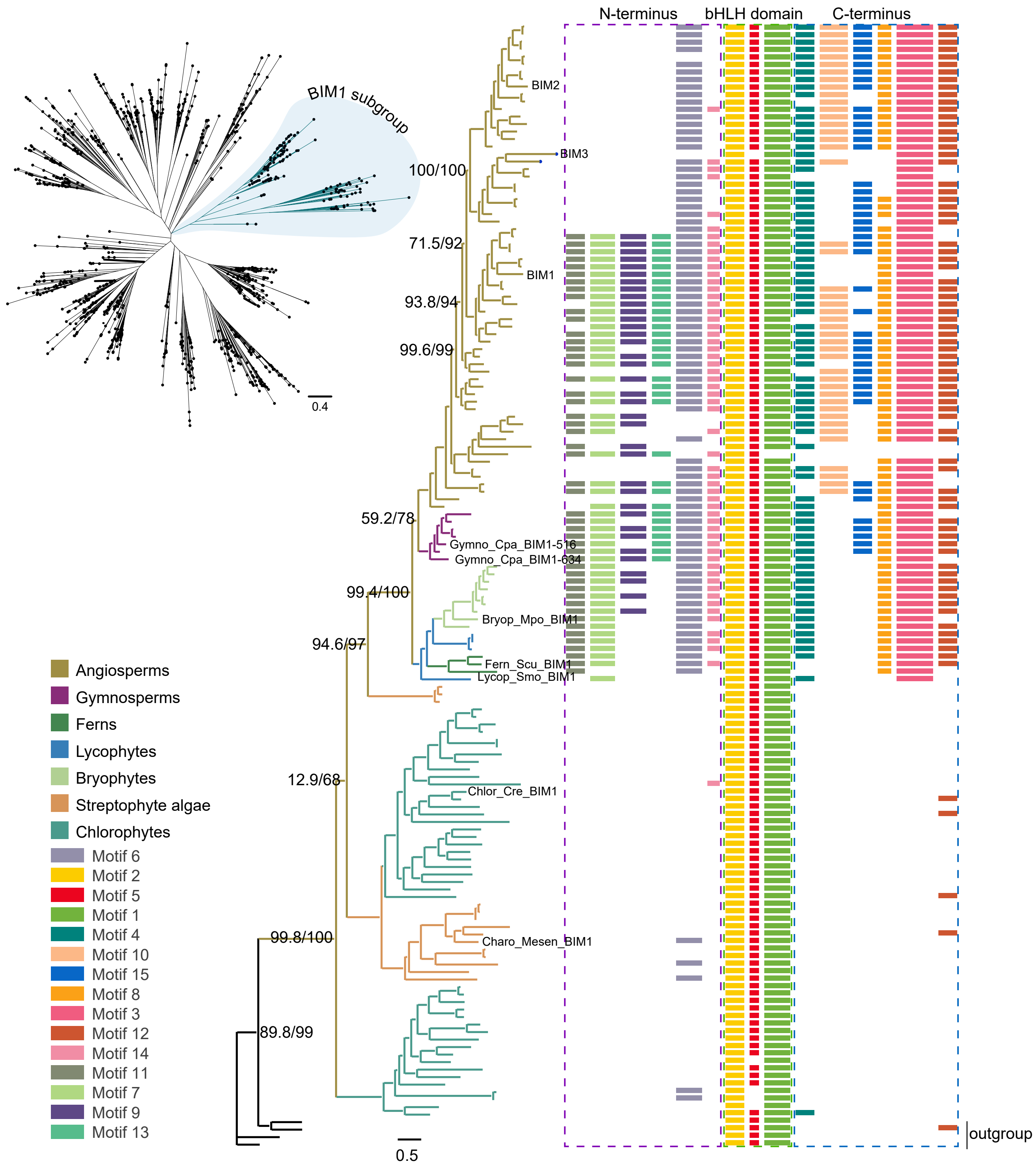

Supplementary Figure 5. Phylogenetic tree and domain architecture of *BIM1* orthologs. Maximum-likelihood phylogeny based on amino acid sequence alignments, with the JTT+F+R5 model and 1,000 ultrafast bootstraps. The closest paralogs (black branches) were used to root the tree. Scale bar: amino acid substitutions per site. MEME-identified motifs are shown to the right as colored boxes; the dashed box marks the N-terminal region, the bHLH domain, and the C-terminal region. Motif sequences are listed in Supplementary Table 10.

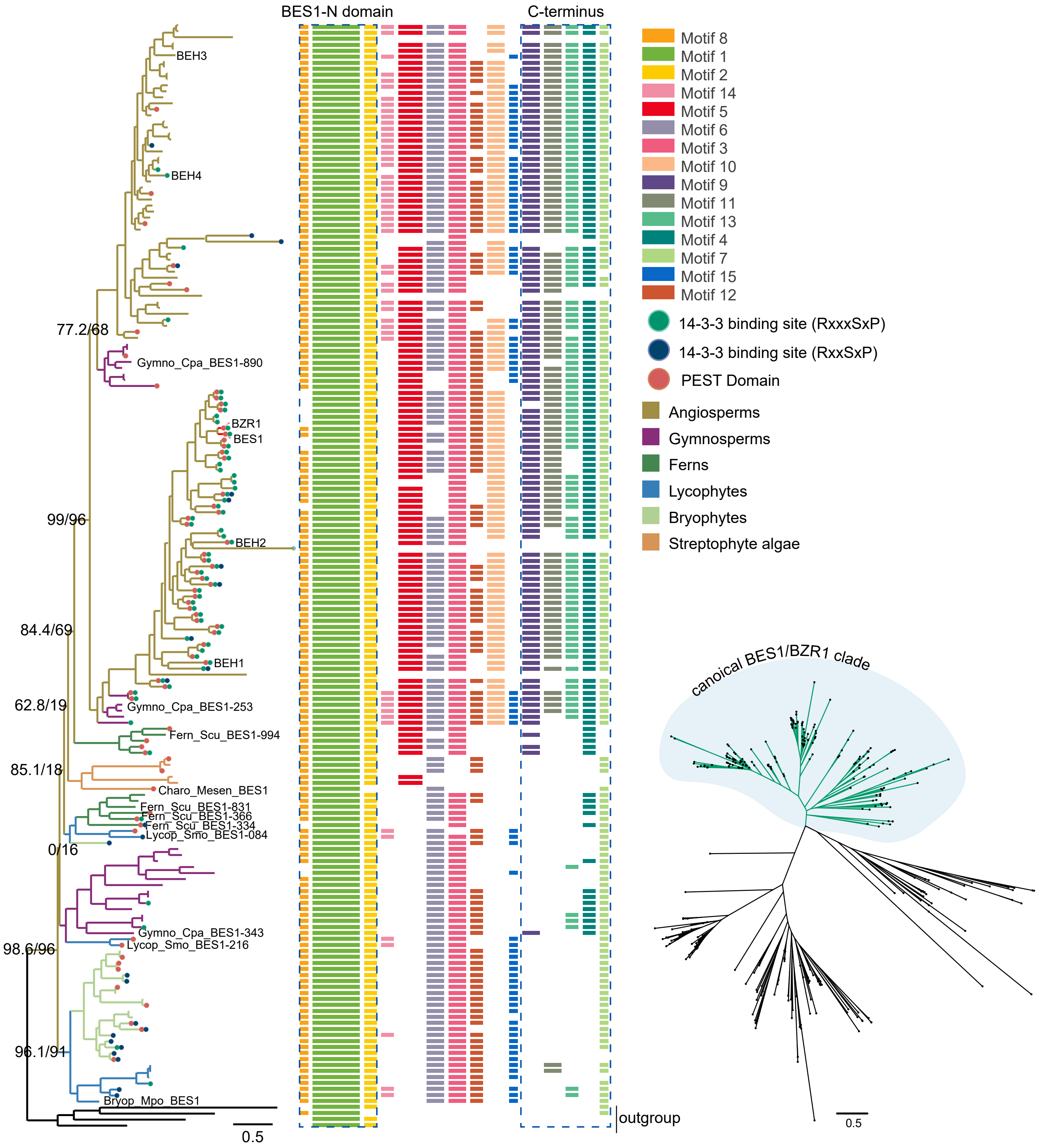

Supplementary Figure 6. Phylogenetic tree and domain architecture of *BES1* orthologs. Maximum-likelihood phylogeny based on amino acid sequence alignments, with the JTT+I+R7 model and 1,000 ultrafast bootstraps. The closest paralogs (black branches) were used to root the tree. Scale bar: amino acid substitutions per site. MEME-identified motifs are shown to the right as colored boxes; the dashed box marks the BES1 N-terminal domain and the C-terminal region. Motif sequences are listed in Supplementary Table 10.

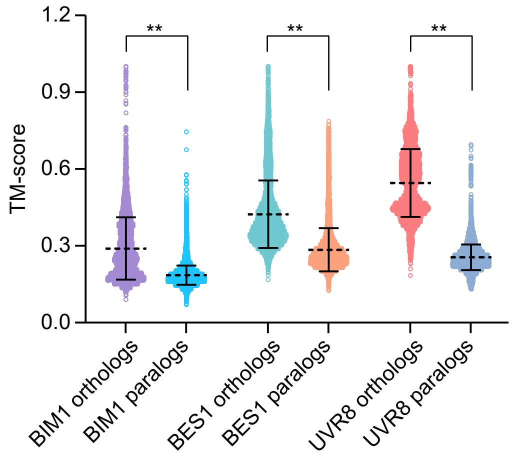

Supplementary Figure 7. Pairwise TM-scores between orthologs and paralogs. Pairwise protein structural alignments were performed using Foldseek (Van Kempen et al., 2024). *p* values were calculated using two-tailed Student's t-tests. $**p < 0.01$.

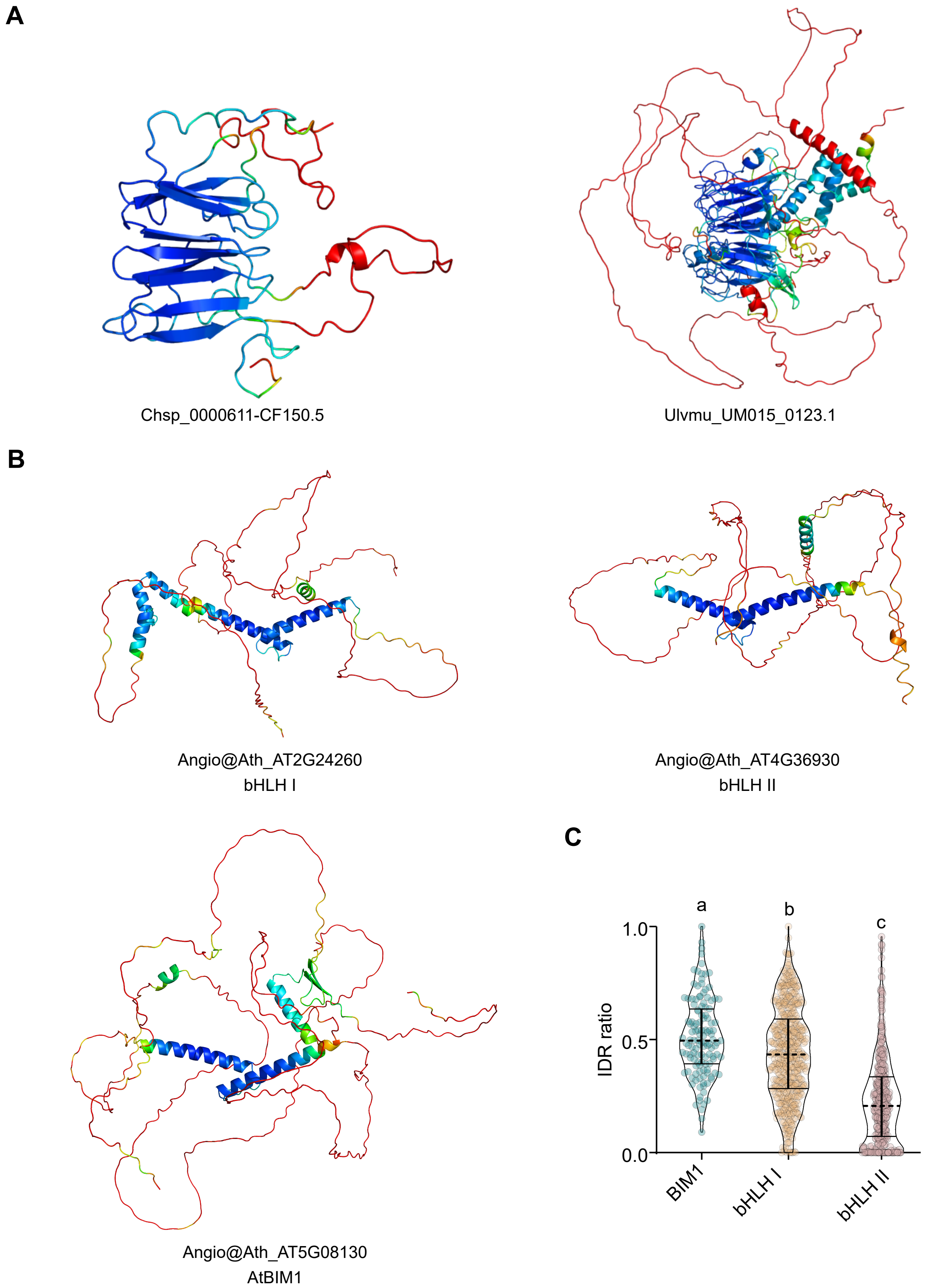

Supplementary Figure 8. Structural divergence between orthologs and paralogs of UVR8- and BIM1-like proteins. **A**, Example of structural divergence in UVR8 paralogs: Chsp_0000611-CF150.5 lacks full β-propeller architecture and Ulvmu_UM015_0123.1 carries an extra C-terminal domain beyond the canonical UVR8 core. **B**, Example of structural divergence in AtBIM1 and its paralogs. AtBIM1 harbors more intrinsically disordered regions than its paralogs. **C**, Ratio of intrinsically disordered region (IDR) length to total protein length for each sequence in the bHLH subfamily.

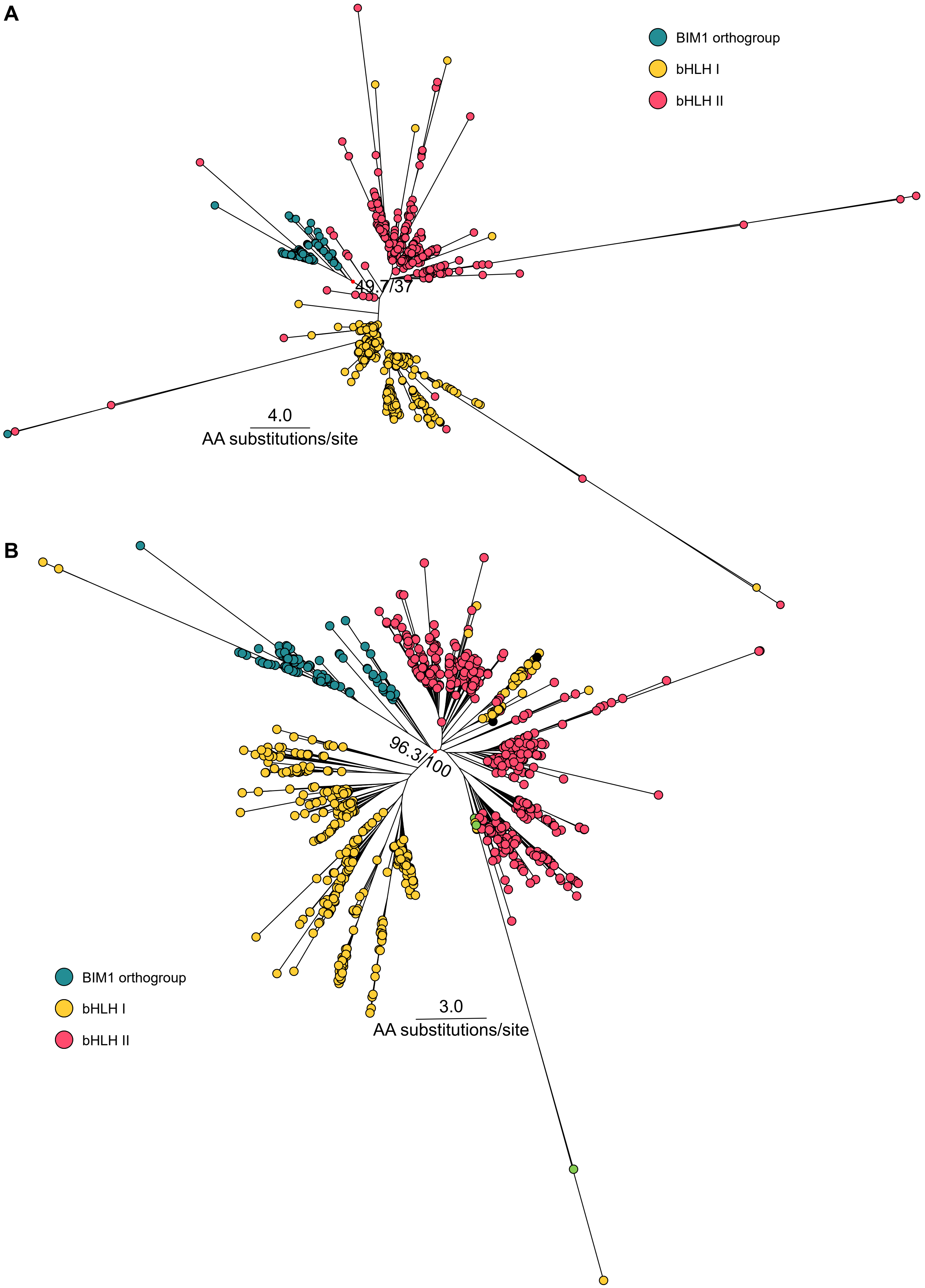

Supplementary Figure 9. Impact of IDRs on BIM1 phylogenetic reconstruction. A, Structure-guided unrooted tree of BIM1 constructed with a trimming threshold of 0.5 (-gt 0.5). **B,** Structure-guided unrooted tree of BIM1 constructed without trimming. Solid green circles at the tips denote the BIM1 clade, while red and yellow circles represent bHLH II and bHLH I, respectively.

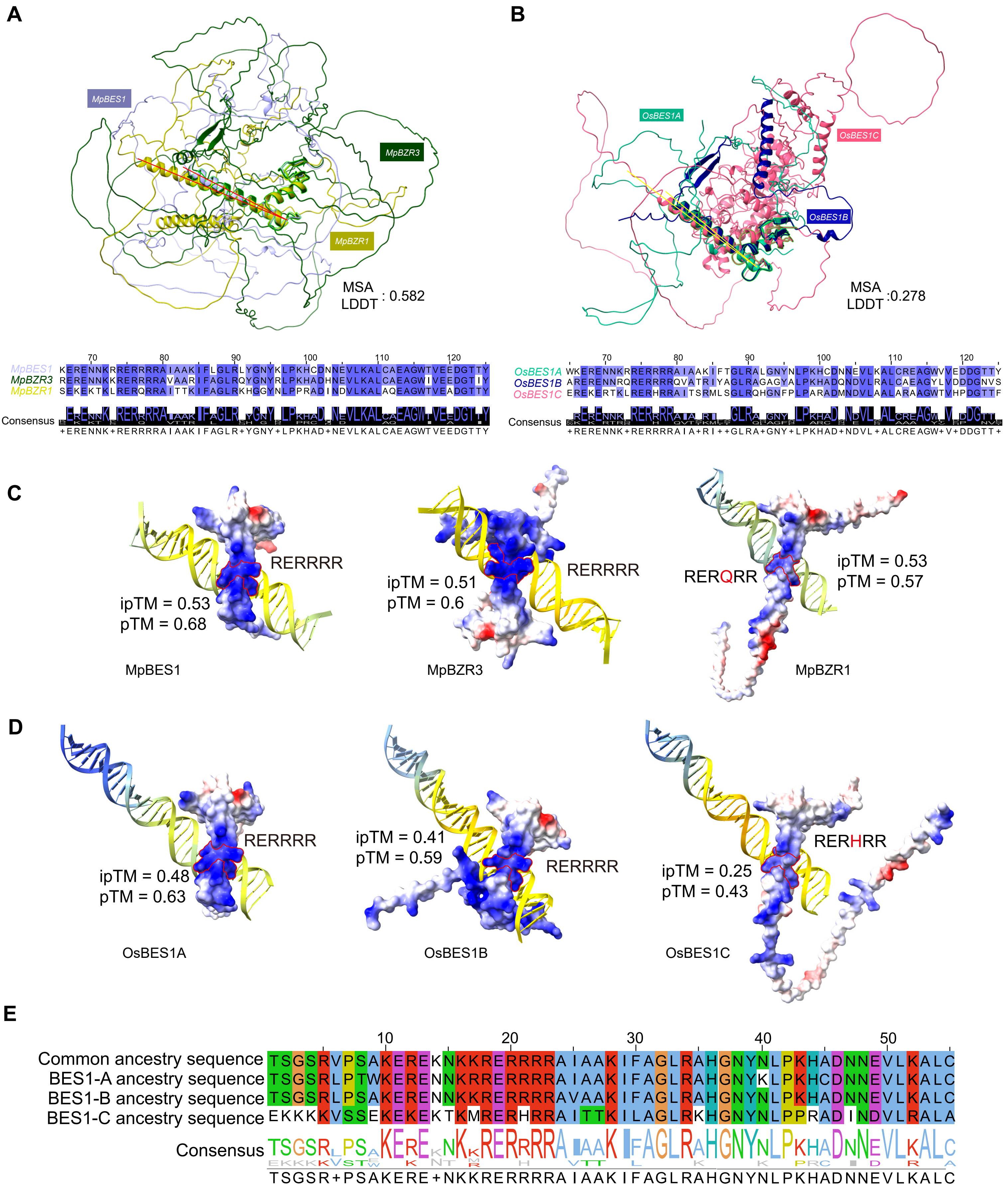

Supplementary Figure 10. Structural diversification of BES1 subfamilies in land plants. A,B, Structure alignment of BES1-A, BES1-B, and BES1-C subfamily members from *M. polymorpha* (A) and *O. sativa* (B). **C, D,** Electrostatic potential surfaces of molecular docking models showing the interaction between DNA and BES1 subfamily members from *M. polymorpha* (C) and *O. sativa* (D). The interface predicted template modeling (ipTM) and predicted template modeling (pTM) scores are indicated. In the electrostatic potential maps, deep blue indicates positively charged regions. **E**, Ancestral sequence reconstruction of the BES1-N domain.

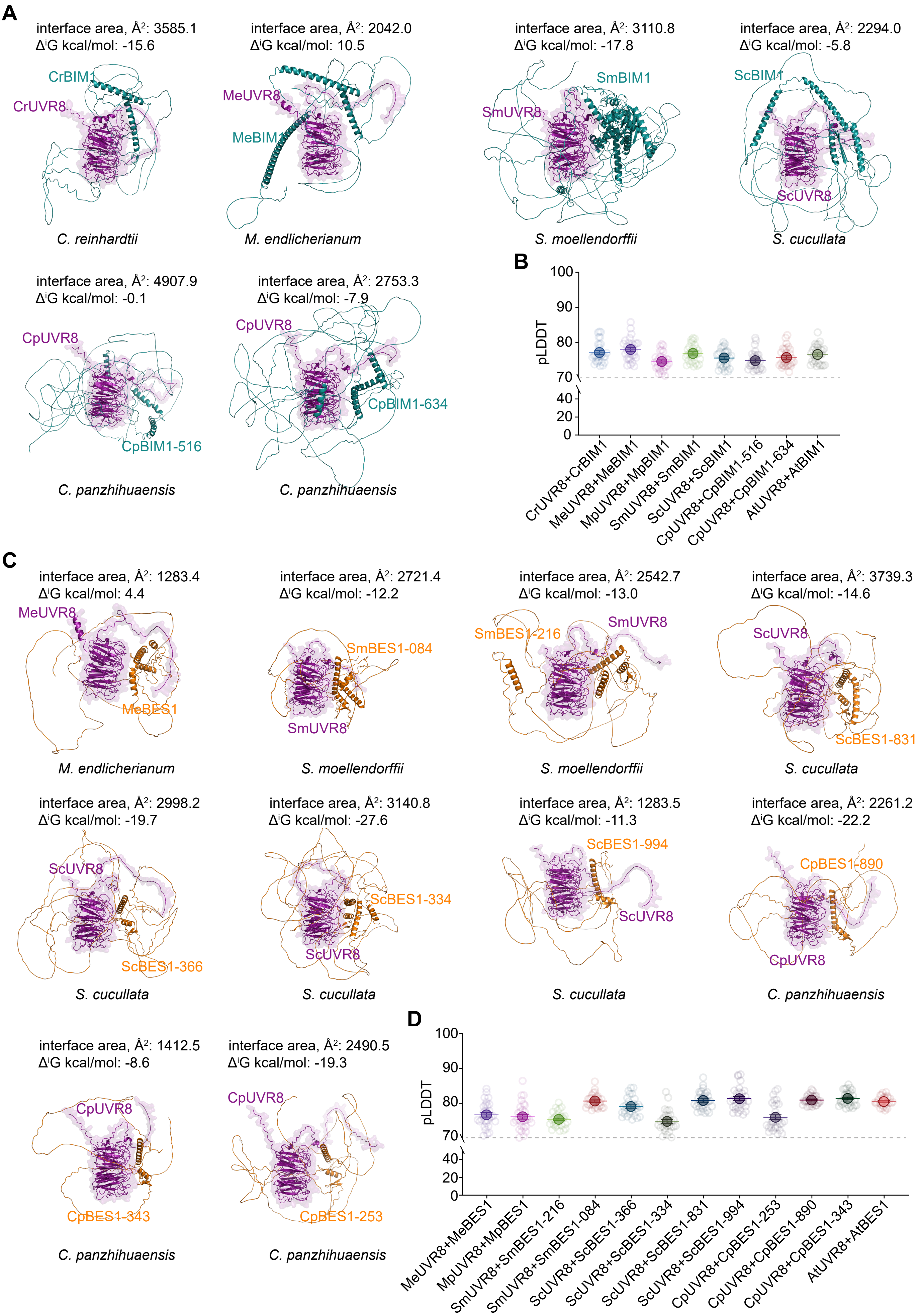
A
interface area, Å²: 3585.1
ΔⁱG kcal/mol: -15.6
CrBIM1
CrUVR8
C. reinhardtii
interface area, Å²: 2042.0
ΔⁱG kcal/mol: 10.5
MeUVR8
MeBIM1
M. endlicherianum
interface area, Å²: 3110.8
ΔⁱG kcal/mol: -17.8
SmBIM1
SmUVR8
S. moellendorffii
interface area, Å²: 2294.0
ΔⁱG kcal/mol: -5.8
ScBIM1
ScUVR8
S. cucullata
interface area, Å²: 4907.9
ΔⁱG kcal/mol: -0.1
CpUVR8
CpBIM1-516
C. panzhihuaensis
interface area, Å²: 2753.3
ΔⁱG kcal/mol: -7.9
CpUVR8
CpBIM1-634
C. panzhihuaensis
B
pLDDT
100
90
80
70
40
20
0
CrUVR8+CrBIM1
MeUVR8+MeBIM1
MpUVR8+MpBIM1
SmUVR8+SmBIM1
ScUVR8+ScBIM1
CpUVR8+CpBIM1-516
CpUVR8+CpBIM1-634
AtUVR8+AtBIM1
C
interface area, Å²: 1283.4
ΔⁱG kcal/mol: 4.4
MeUVR8
MeBES1
M. endlicherianum
interface area, Å²: 2721.4
ΔⁱG kcal/mol: -12.2
SmBES1-084
SmUVR8
S. moellendorffii
interface area, Å²: 2542.7
ΔⁱG kcal/mol: -13.0
SmBES1-216
SmUVR8
S. moellendorffii
interface area, Å²: 3739.3
ΔⁱG kcal/mol: -14.6
ScUVR8
ScBES1-831
S. cucullata
interface area, Å²: 2998.2
ΔⁱG kcal/mol: -19.7
ScUVR8
ScBES1-366
S. cucullata
interface area, Å²: 3140.8
ΔⁱG kcal/mol: -27.6
ScBES1-334
ScUVR8
S. cucullata
interface area, Å²: 1283.5
ΔⁱG kcal/mol: -11.3
ScBES1-994
ScUVR8
S. cucullata
interface area, Å²: 2261.2
ΔⁱG kcal/mol: -22.2
CpBES1-890
CpUVR8
C. panzhihuaensis
interface area, Å²: 1412.5
ΔⁱG kcal/mol: -8.6
CpUVR8
CpBES1-343
C. panzhihuaensis
interface area, Å²: 2490.5
ΔⁱG kcal/mol: -19.3
CpUVR8
CpBES1-253
C. panzhihuaensis
D
pLDDT
100
90
80
70
40
20
0
MeUVR8+MeBES1
MpUVR8+MpBES1
SmUVR8+SmBES1-216
SmUVR8+SmBES1-084
ScUVR8+ScBES1-366
ScUVR8+ScBES1-334
ScUVR8+ScBES1-831
ScUVR8+ScBES1-994
CpUVR8+CpBES1-253
CpUVR8+CpBES1-890
CpUVR8+CpBES1-343
AtUVR8+AtBES1

Supplementary Figure 11. Predicted dimeric structures of UVR8-BIM1 and UVR8-BES1. A,**C**, Examples of predicted structures for UVR8-BIM1 (A) and UVR8-BES1 (C) dimers in representative species. $\Delta^{i}G$ indicates the solvation free energy gain upon formation of the interface (in kcal/M). Lower $\Delta^{i}G$ and larger interface areas indicates higher likelihood of physical interaction. **B**,**D**, pLDDT scores of UVR8-BIM1 (B) and UVR8-BES1 (D) dimer structures in representative species. Each dot represents one prediction repeat. pLDDT $\geq$ 70 denotes confident structures.

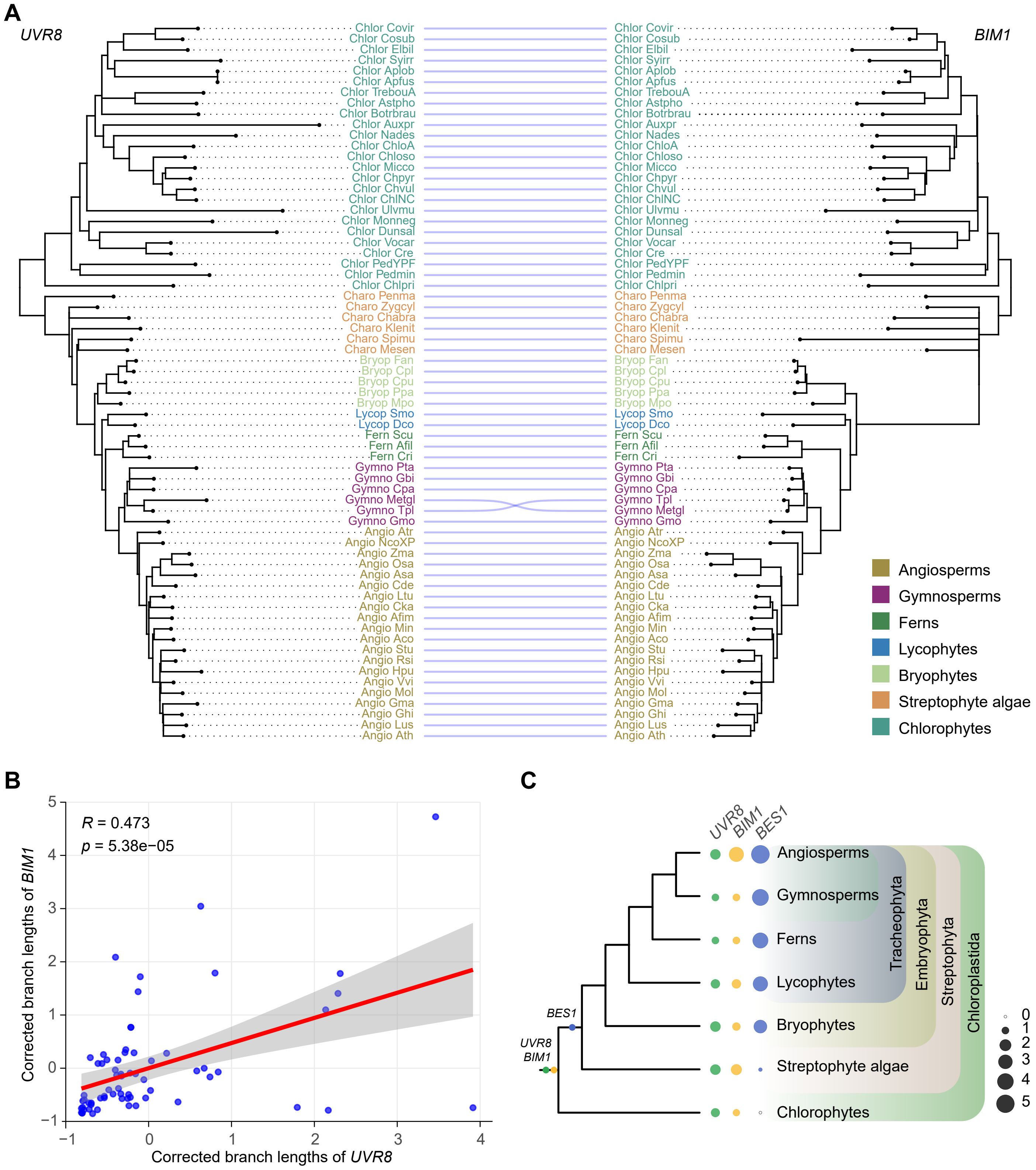

Supplementary Figure 12. Coordinated evolution of UVR8 and BIM1 in green plants. **A**, The mirror-tree of *UVR8* and *BIM1*. **B**, Evolutionary rate covariation (ERC) analysis between *UVR8* and *BIM1* was performed using the CovER function in PhyKIT. **C**, Copy number of *UVR8*, *BIM1*, and *BES1* orthologs in major green plant lineages.

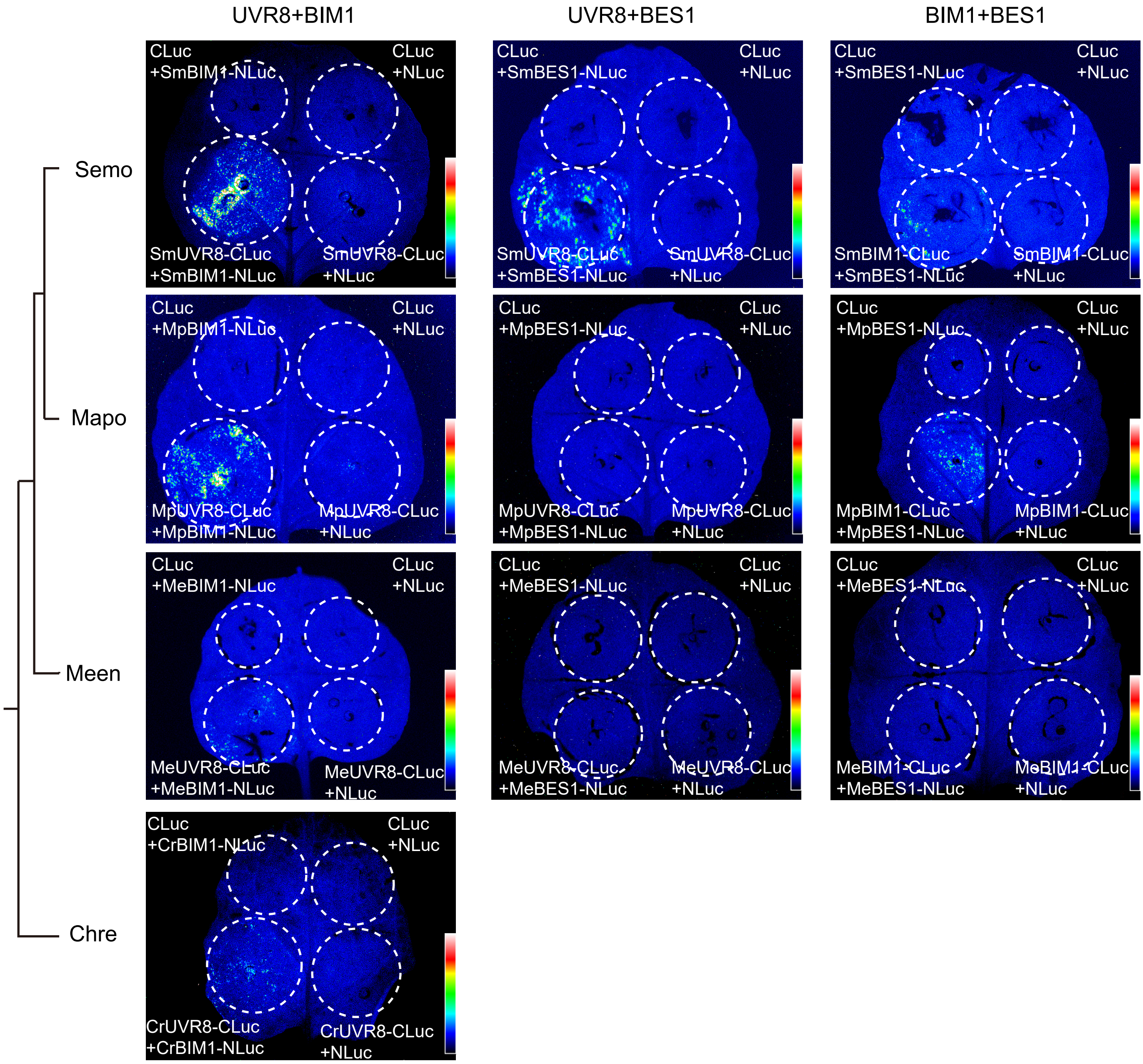

Supplementary Figure 13. Representative images of luciferase complementation in tobacco leaves. The pseudocolor scale indicates the luminescence intensity, with red and blue representing high and low signals, respectively. CLuc + NLuc-gene, CLuc-gene + NLuc, and CLuc + NLuc were used as negative controls.

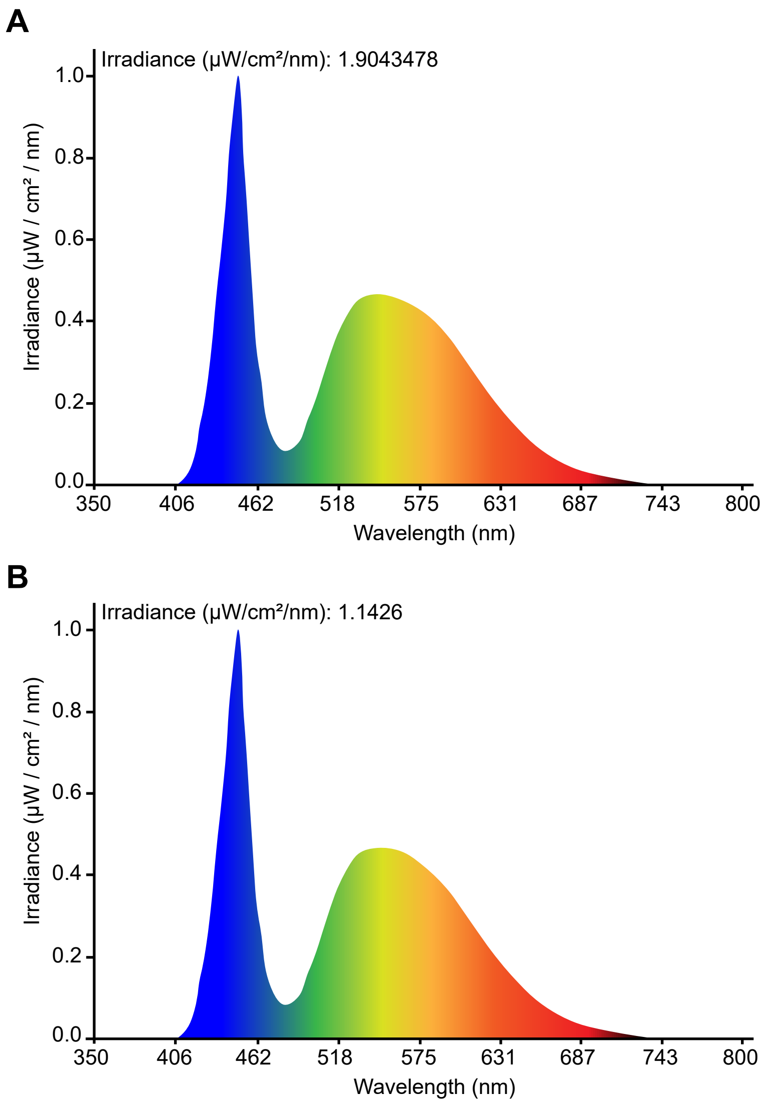

Supplementary Figure 14. Light spectra used for *M. endlicherianum* and *M. polymorpha* cultivation. A, B, Light spectra of the growth chambers used for *M. polymorpha* (A) and *M. endlicherianum* (B).

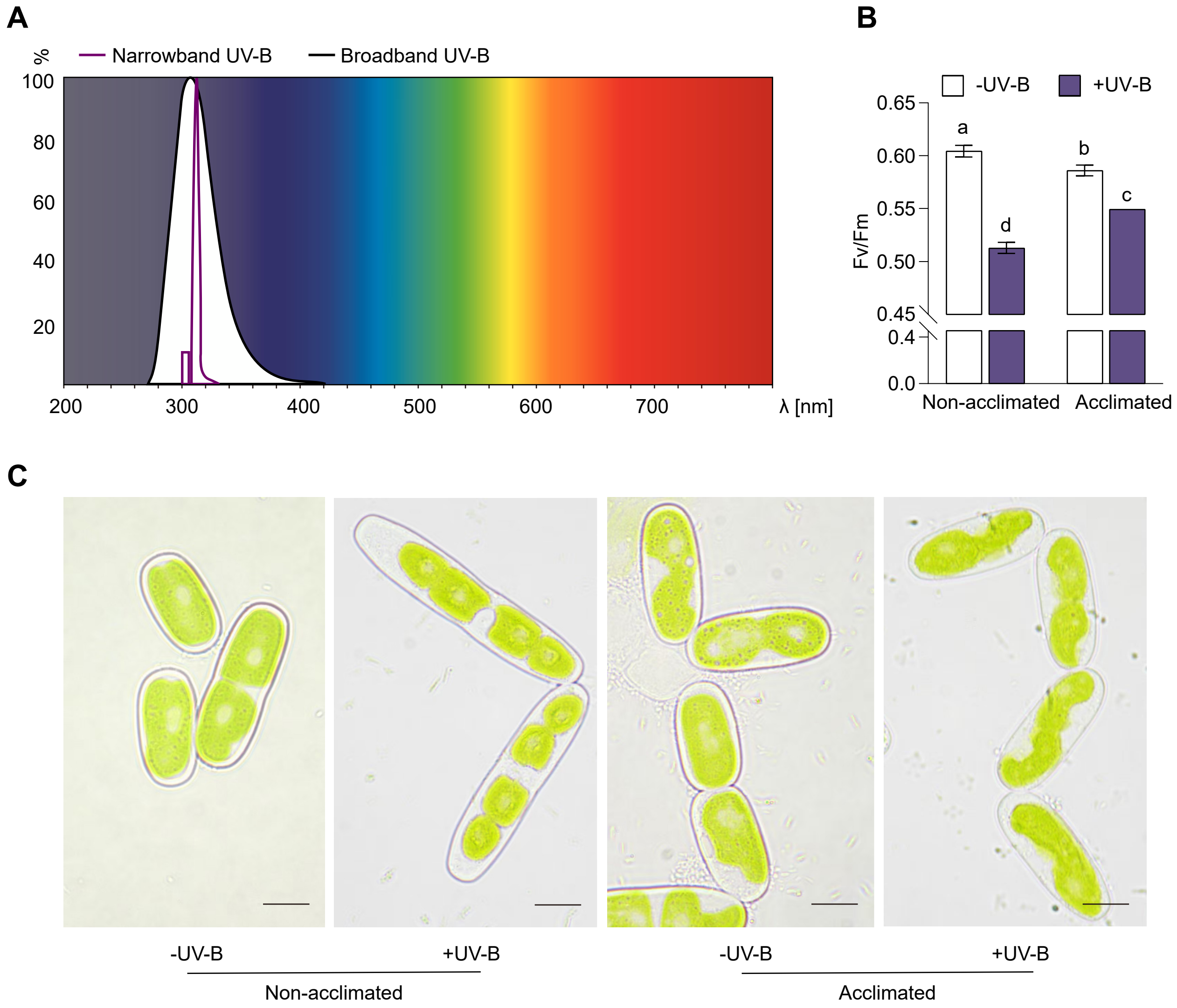

Supplementary Figure 15. Physiological and morphological responses of *M. endlicherianum* to narrowband and broadband UV-B. A, Spectral distribution of the narrowband and broadband UV-B light sources. **B,** Fv/Fm in *M. endlicherianum* under UV-B stress. Data were recorded after 3 h of broadband UV-B irradiation following 3 days of narrowband UV-B acclimation (or in non-acclimated controls). **C,** Representative images showing the cell morphology of *M. endlicherianum* under UV-B stress with or without prior narrowband UV-B acclimation. Scale bar, 100 nm.

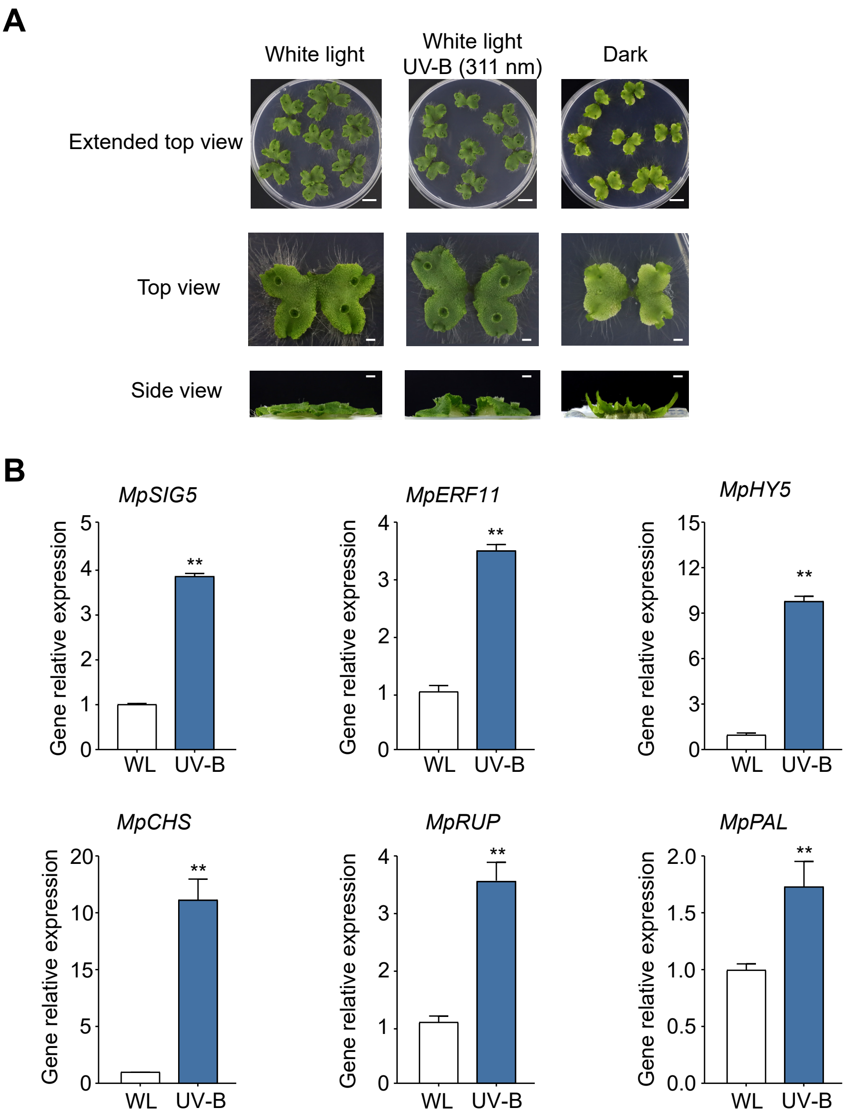

Supplementary Figure 16. UV-B-induced morphological and transcriptional responses in *M. polymorpha*. **A**, Morphology of 12-day-old *M. polymorpha* gemmalings (Tak-1, wild-type, male) after 7-day treatments under different light conditions. Scale bars: 1 cm (extended top view); 200 µm (top view and side view). **B**, Relative expression levels of UV-B-responsive genes under white light (WL) and WL + UV-B treatments in *M. polymorpha*. Data are presented as means ± SD (n = 3). ** denotes a statistically significant difference compared to control (Student's *t*-test, $p < 0.01$).

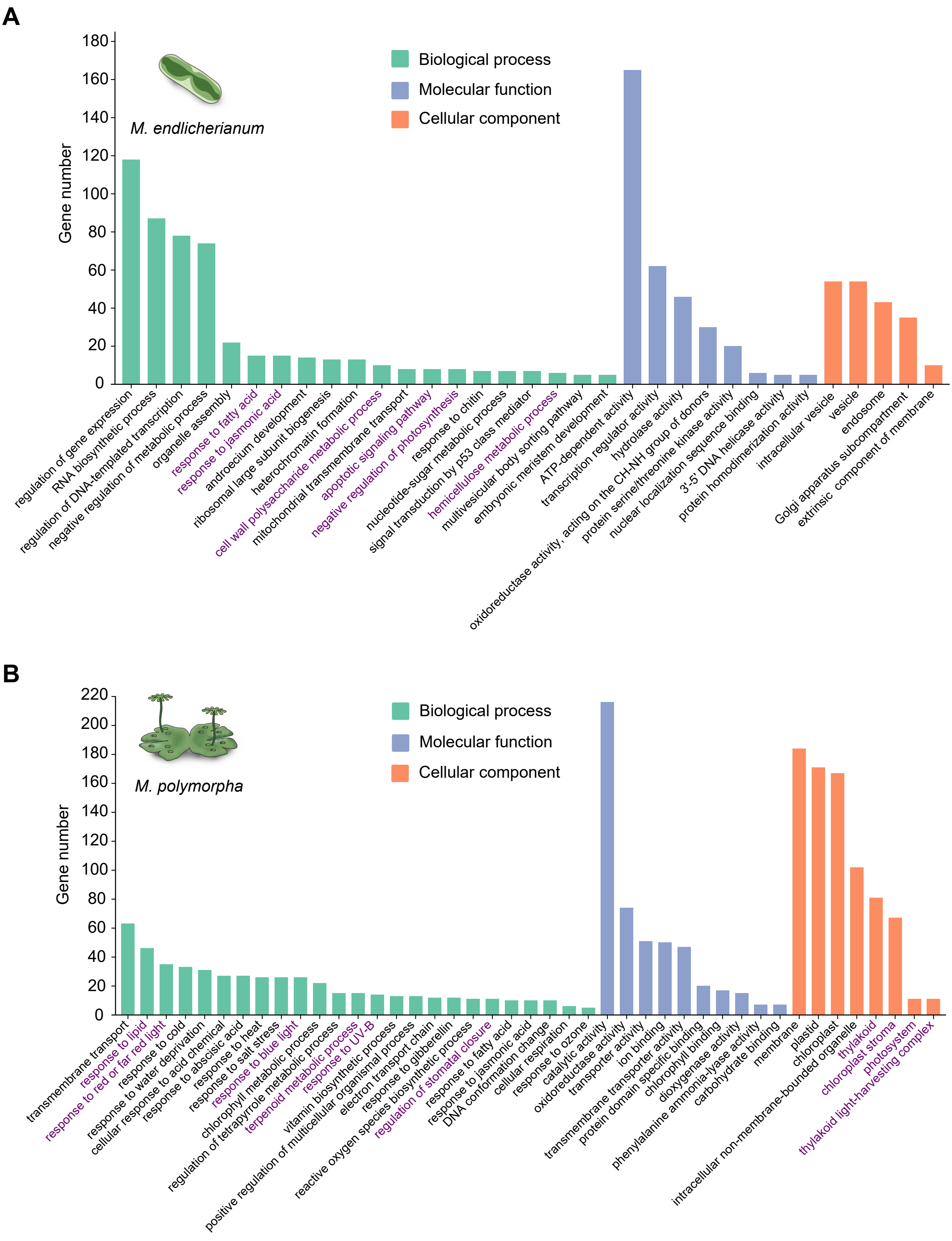

Supplementary Figure 17. GO enrichment analysis of UV-B-responsive DEGs in *M. endlicherianum* and *M. polymorpha*. **A**, GO enrichment of UV-B-responsive DEGs identified in *M. endlicherianum*. **B**, GO enrichment of UV-B-responsive DEGs identified in *M. polymorpha*.

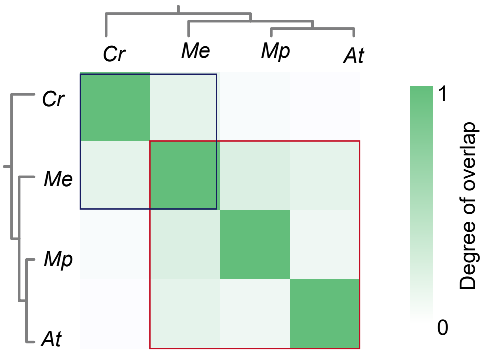

Supplementary Figure 18. Pairwise overlap of UV-B-responsive gene orthogroups across selected plant species. Heatmap color intensity reflects the degree of overlap in UV-B-responsive orthogroups (Green: high; white: low). Boxes highlight comparisons in which the proportion of shared orthogroups exceeds 60% (0.206), indicating relatively conserved UV-B-responsive transcriptional programs.

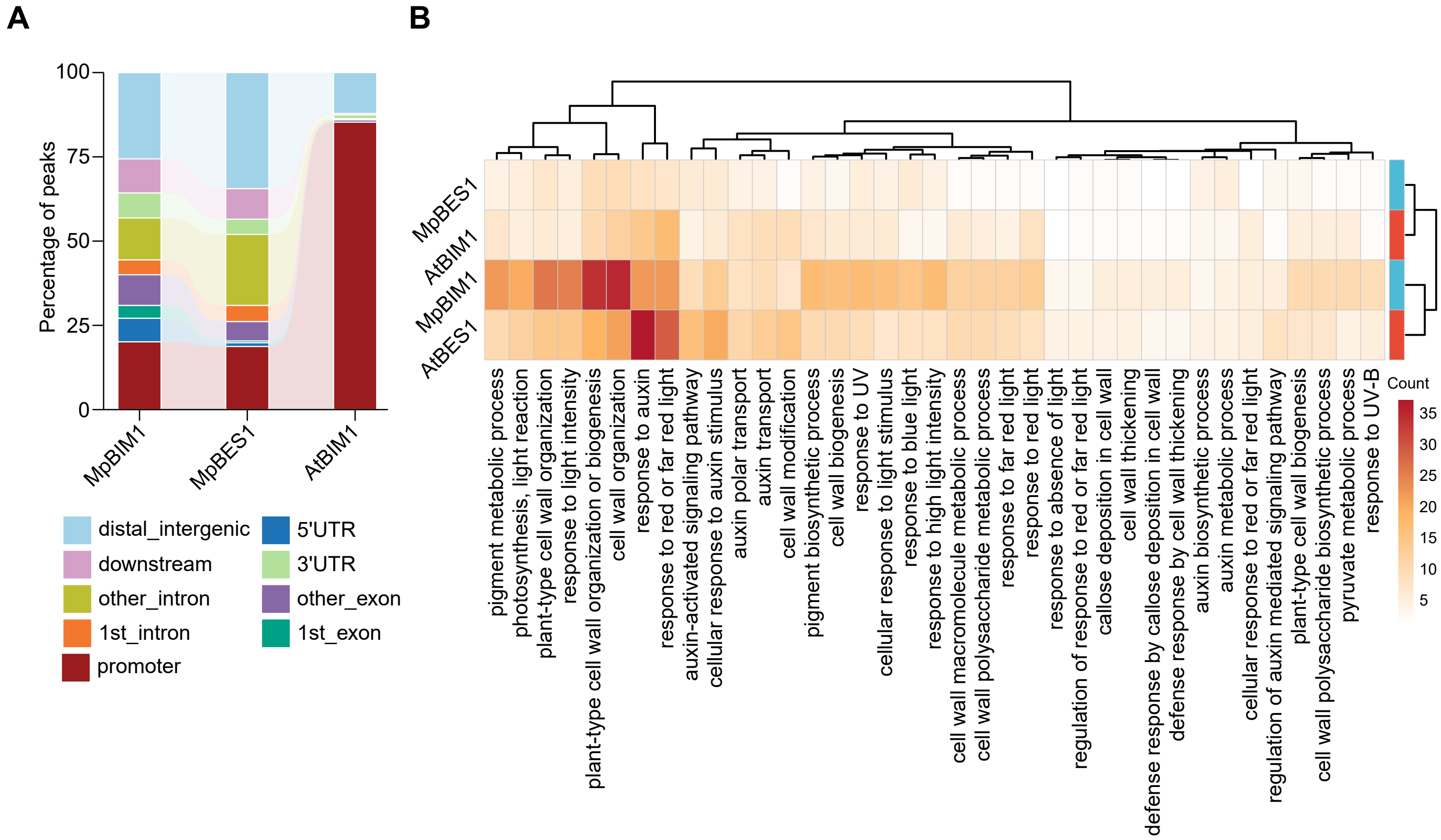

Supplementary Figure 19: Genome-wide binding sites distribution and functional annotation of BIM1 and BES1. A, Genomic distribution of MpBIM1, MpBES1, and AtBIM1 binding sites. **B,** GO enrichment analysis of putative target genes bound by BIM1 and BES1 in *M. polymorpha* and *A. thaliana*.

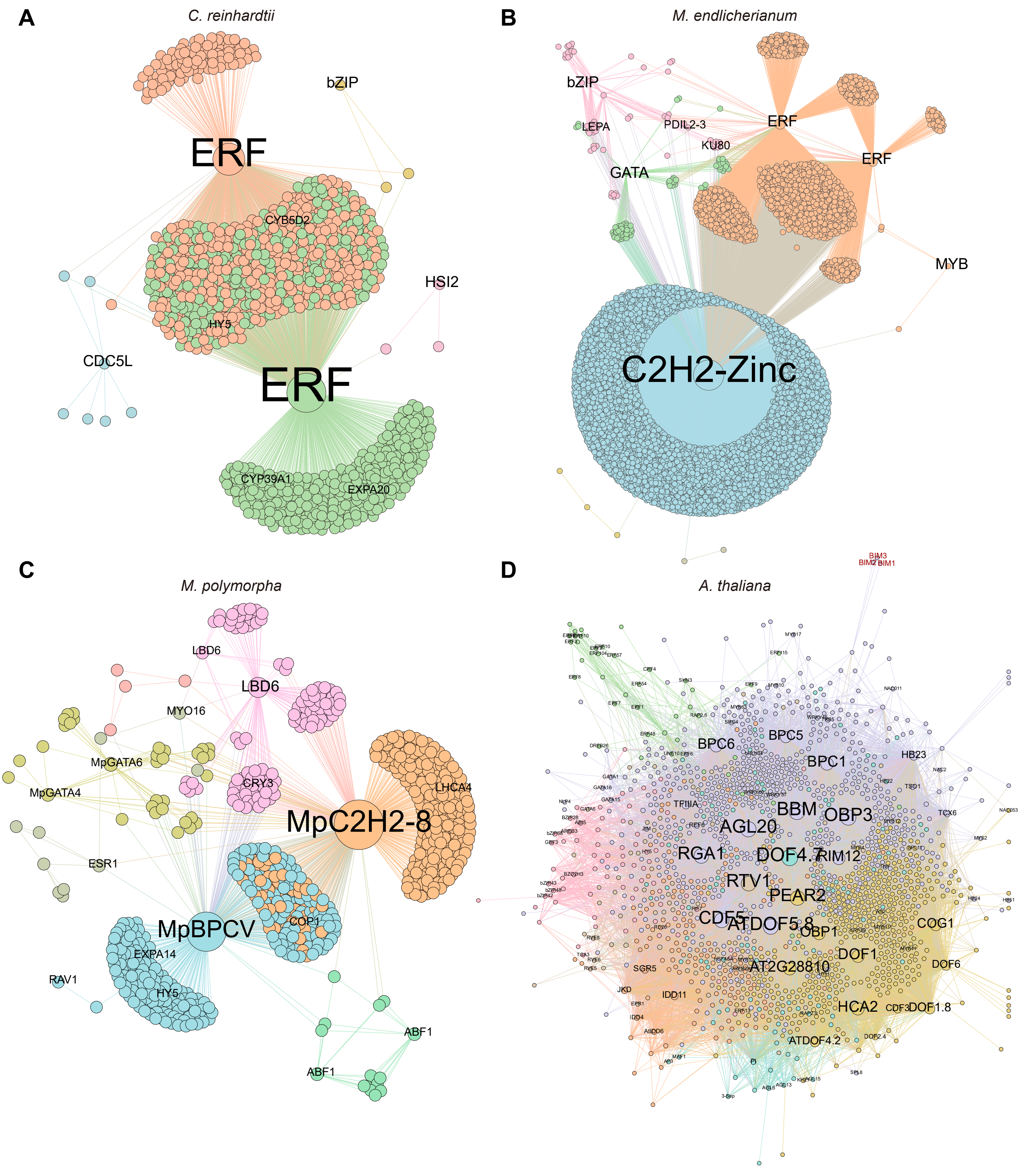

Supplementary Figure 20. Inferred UV-B gene regulatory networks in algae and land plants. A, *C. reinhardtii*. **B,** *M. endlicherianum*. **C,** *M. polymorpha*. **D,** *A. thaliana*.

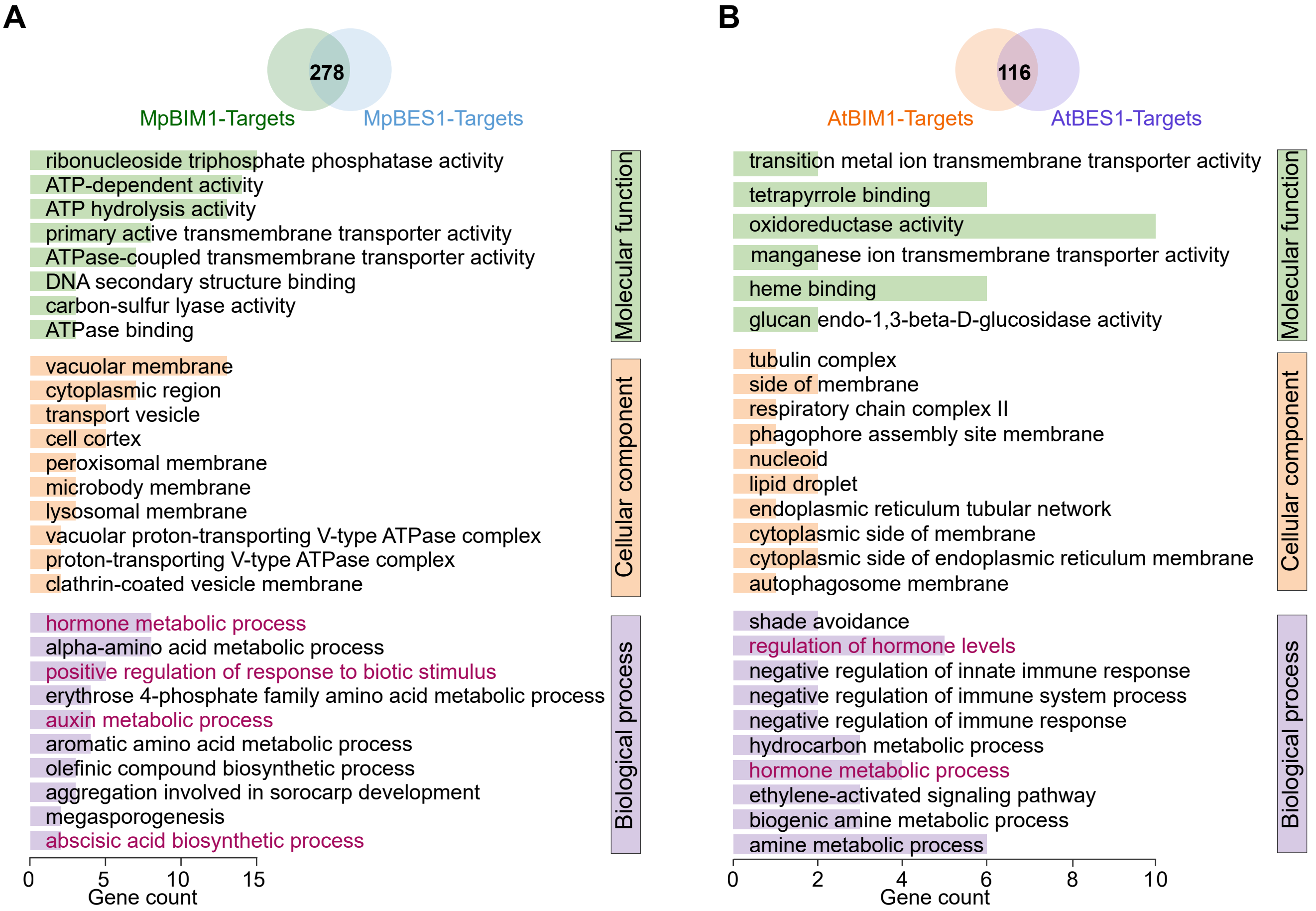

Supplementary Figure 21. Functional enrichment of BIM1/BES1 shared targets. A,B, GO enrichment analysis of the overlapping target genes shared by MpBIM1 and MpBES1 (A) and by AtBIM1 and AtBES1 (B).

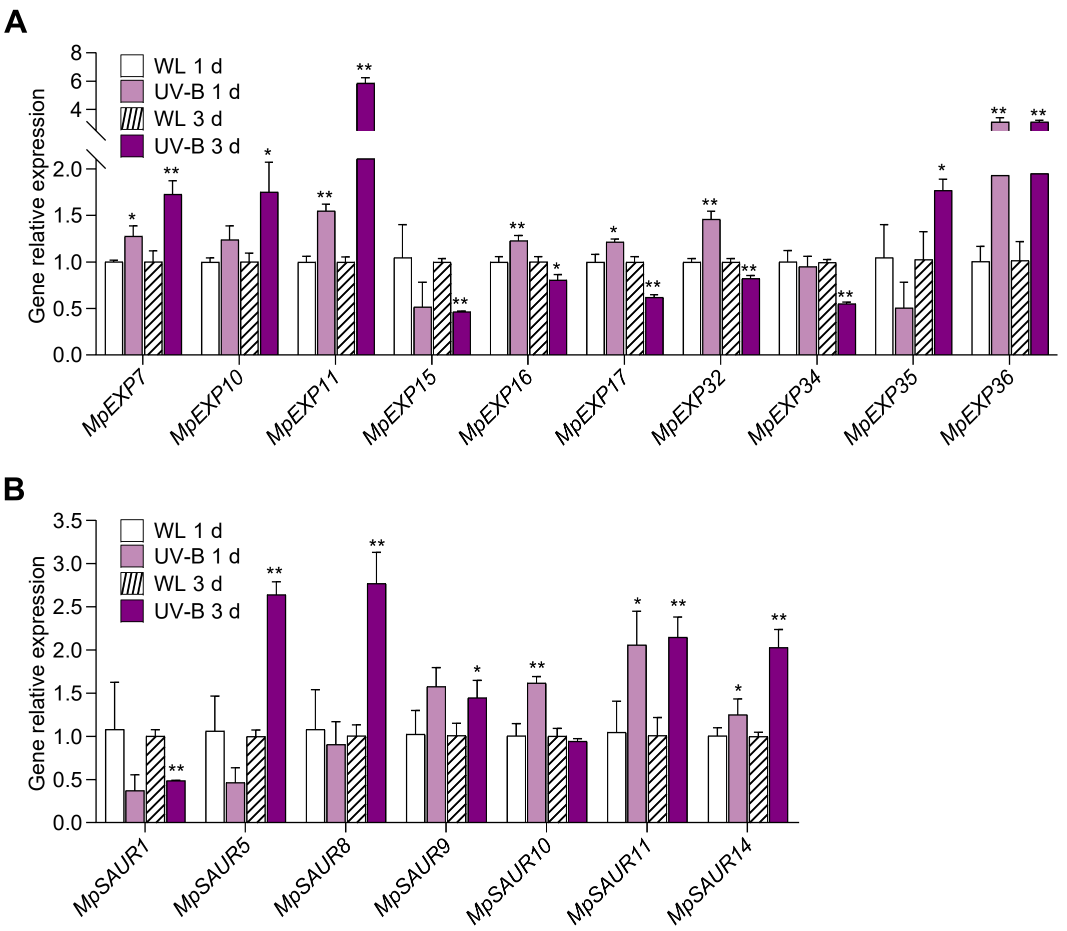

Supplementary Figure 22. Relative expression levels of *EXPANSIN* and *SAUR* gene families. A,**B**, Relative expression levels of *EXPANSIN* (A) and *SAUR* (B) genes under WL and WL + UV-B treatments in *M. polymorpha*. Data are presented as means ± SD (n = 3). *p* values were calculated using two-tailed Student's *t*-tests. *$p < 0.05$; **$p < 0.01$.
